# Iron-Mediated Nitrate Reduction at Ambient Temperature for Deaminative Sulfonylation and Fluorination of Anilines

**DOI:** 10.1021/jacs.4c17981

**Published:** 2025-05-09

**Authors:** Tim Schulte, Deepak Behera, Davide Carboni, Annika Höppner, Felix Waldbach, Javier Mateos, Ahmet Altun, Markus Leutzsch, Moritz L. Krebs, Tobias Ritter

**Affiliations:** 1 28314 Max-Planck-Institut für Kohlenforschung, Kaiser-Wilhelm-Platz 1, Mülheim an der Ruhr 45470, Germany; 2 Institute of Organic Chemistry, 9165 RWTH Aachen University, Landoltweg 1, Aachen 52074, Germany

## Abstract

Preparation of arylsulfonic acids and derivatives can be achieved under mild conditions from aryldiazonium salts, although conventional methods often require isolation or accumulation of these potentially hazardous intermediates. Herein, we present that iron nitrate reduction at 25 °C enables the *in situ* generation of diazonium salts, which allows direct deaminative chlorosulfonylation and fluorination from anilines via aryldiazonium salts as fleeting intermediates. Other sulfonic acid derivatives, such as sulfonamides, sulfonyl fluorides, and sulfonic acids, are readily accessible from this method.

## Graphical abstract

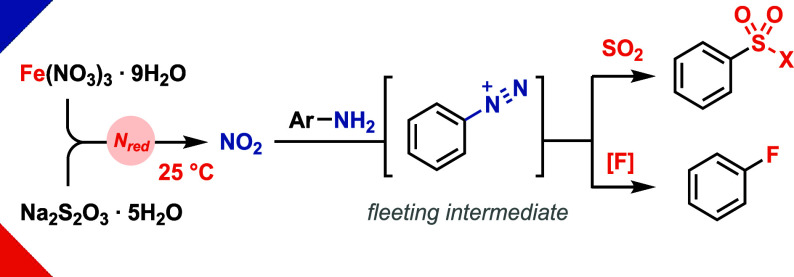

## Introduction

Biological nitrate reduction is pivotal for the geochemical nitrogen cycle and the metabolism in plants,^1,2^ and chemical nitrate reduction is a topic of current research.^3,4^ Sophisticated molybdenum- and iron-based catalysts that mimic biological nitrate reduction under ambient conditions have been developed and studied in remarkable detail^5,6^ but not yet been used for transformations in organic chemistry.^6,7^ Our group has recently reported the utilization of rate-limiting nitrate reduction for safer aryldiazonium chemistry,^8^ but the kinetic stability of nitrate prevented the development of transformations that did not tolerate the 85 °C required for nitrate reduction. Here, we report nitrate reduction at ambient temperature enabled by stoichiometric iron­(III). The facile nitrate reduction in the presence of iron allows the *in situ* generation of aryldiazonium salts at ambient temperature and thereby not only increases the safety profile but also enables the direct synthesis of sulfonyl chlorides from anilines and aminoheterocycles. Additionally, we demonstrate that nitrate reduction can be utilized for the direct generation of sulfonic acids from anilines with inexpensive reagents and that iron also expands nitrate-based deaminative halogenation by successful aryl fluoride synthesis.

The interconversion between the nitrogen species with formal oxidation states from + V (nitrate) to – III (ammonia) proceeds by a complex redox network to which nitrate reduction is crucial.^6,9^ In plants, molybdenum-based nitrate reductases catalyze the reduction of nitrate to nitrite using NADH or FADH_2_ cofactors.^10,11^ To mimic the biological process, Holm pioneered the use of molybdenum catalysts as “artificial enzymes”^12^ for nitrate reduction (Figure 1A).^5^ Fout reported an iron-based complex that catalyzes the reduction of nitrate to NO (Figure 1A).^6^ Our group has recently reported the combination of nitrate and nitrate ester reduction with the *in situ* generation of aryldiazonium salts to achieve deaminative halogenation of anilines and aminoheterocycles that requires a temperature of 85 °C.^8^ This temperature precludes the use of nitrate reduction for deamination reactions, in which the conversion of the diazonium salt must proceed at lower temperatures, such as the conversion of aryldiazoniums to sulfonyl chlorides reported by Meerwein.^13^ Here, we show how a simple iron­(III) salt in stoichiometric quantity can facilitate nitrate reduction and thereby enable direct sulfonyl chloride synthesis.

**Figure 1:**
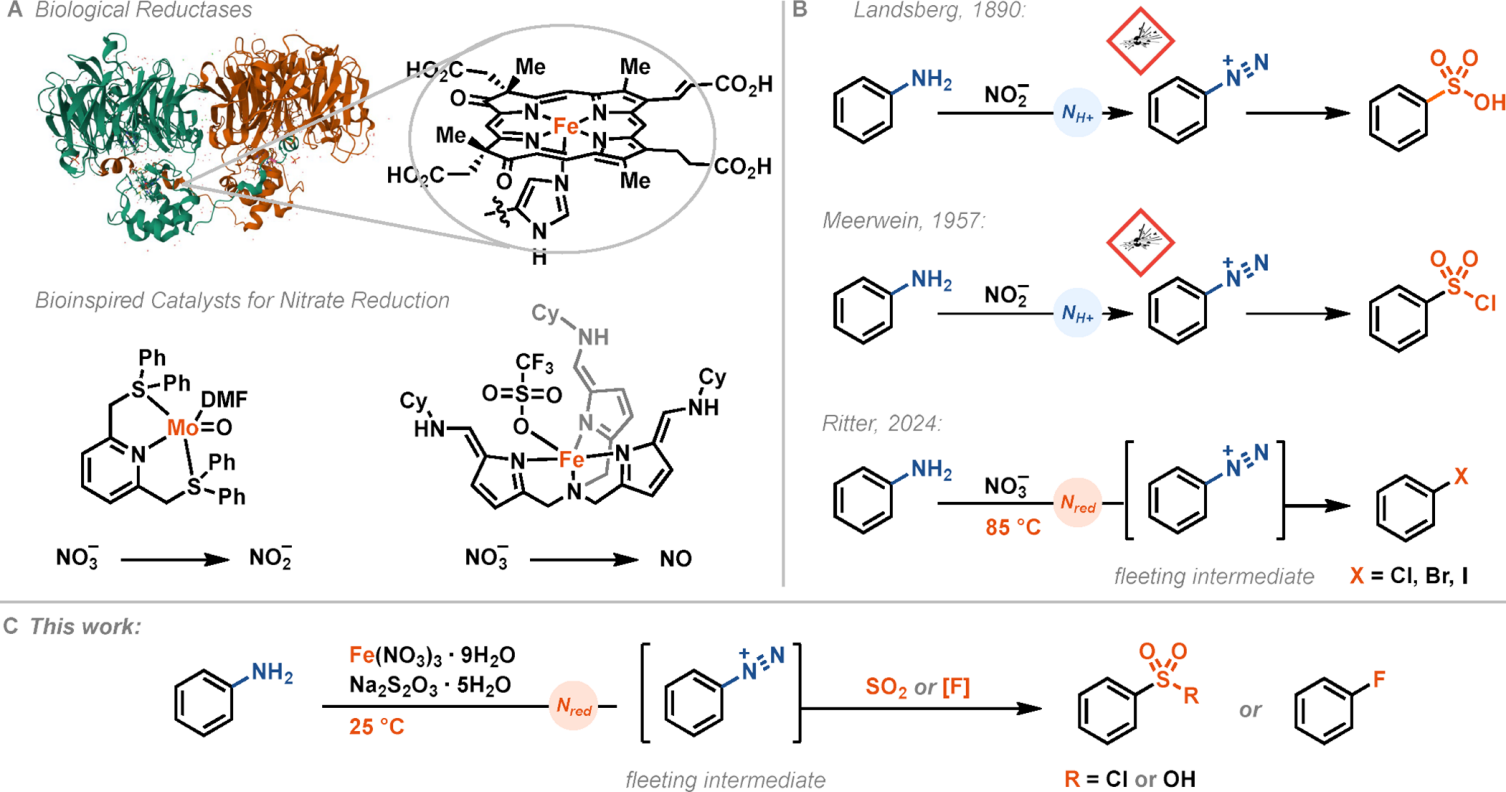
(A) Structure of cd1 nitrite reductase NirS (PDB = 6TSI) with structure of heme d1; structures of bioinspired catalysts for nitrate reduction. (B) Conversion of diazonium salts presented by Landsberg and Meerwein and deaminative halogenation at 85 °C by Ritter. (C) This work; direct deaminative sulfonylation and fluorination enabled by iron nitrate reduction at 25 °C.

Sulfonic acids and sulfonic acid derivatives are frequent motives found in therapeutics,^14^ dyes,^15^ and chemical reagents.^16^ Sulfonamides have found widespread applications as therapeutics due to their physiochemical, antibacterial, antifungal, and antitumor properties.^14,17^ Sulfonyl fluorides are extensively used in organic synthesis,^16^ due to the development of SuFEx click chemistry by Sharpless.^18^

Arylsulfonic acid derivatives are commonly prepared by nucleophilic substitution of sulfonyl chlorides,^19–21^ which requires prior preparation of the respective sulfonic acid,^22^ typically by electrophilic aromatic sulfonylation, often with harsh reagents, such as oleum. Oxidative conversion of thiols^23–26^ and transition metal-catalyzed methodologies have also been used.^27–29^ The conversion of aryldiazonium salts to sulfonic acids was first described by Landsberg in 1890 using copper hydroxide and sulfurous acid (Figure 1B).^30^ Meerwein demonstrated the conversion of aryldiazonium salts to sulfonyl chlorides in 1957 using copper­(I) chloride and HCl with SO_2_ (Figure 1B).^13^ Anilines are generally less expensive than thiols and aryl halides used in alternative preparation methods for sulfonic acids and sulfonyl chlorides.^23–29^ Modern adaptions of Meerwein’s transformation^13^ have been reported by Hogan with SOCl_2_ ^31^ and by Willis with DABSO in a one-step protocol.^32^ All previous methods for the formation of sulfonic acids or derivatives via aryldiazonium intermediates rely on nitrite-based reagents, therefore either isolate, accumulate, or risk the accumulation of potentially explosive aryldiazoniums.^33^ Concerns in diazonium safety for deaminative chlorosulfonylation have also been addressed with flow chemistry, in which the aryldiazonium salt is only generated in small quantities at a given time.^34^ Direct conversion of anilines to sulfonic acids has, to the best of our knowledge, never been reported.

## Results and Discussion

Combining Meerwein’s chlorosulfonylation conditions, namely, SO_2_, HCl, and CuCl,^13^ with our previously reported deaminative chlorination conditions, which involve the reduction of a nitrate ester at 85 °C,^8^ only lead to a ∼1:1 mixture of sulfonyl chloride **1** with the respective aryl chloride **2** (Figure 2A). We hypothesized that the reaction temperature leads to an insufficient SO_2_ concentration, so that chlorosulfonylation is suppressed by direct Sandmeyer chlorination. Gas dissolution in liquids is typically exothermic; according to Le Chatelier’s principle, higher temperature leads to gas release and lower solubility of gases in liquids.^35^ The lower solubility of SO_2_ in MeCN at elevated temperatures (84.6 g SO_2_ in 100 g MeCN at 25 °C vs 25.6 g of SO_2_ in 100 g MeCN at 50 °C)^36^ favors the formation of the aryl chloride **2** by conventional Sandmeyer chlorination rather than the formation of the sulfonyl chloride **1**. We found that iron nitrate (Fe­(NO_3_)_3_·9H_2_O) reacts with thiosulfate (Na_2_S_2_O_3_·5H_2_O) at 25 °C to form NO_2_ (Figure 2B). When Fe­(NO_3_)_3_·9H_2_O was mixed with Na_2_S_2_O_3_·5H_2_O at 25 °C in the absence of any solvent, nitrate (N^+V^) is reduced to NO_2_ (N^+IV^). The formed NO_2_ was detected in the headspace by gas-phase IR spectroscopy (Figure 2B and Figure S3) and by UV–vis spectroscopy (Figure S4). A similar reduction is not observed with other nitrate salts, such as KNO_3_ or TBANO_3_, which highlights the importance of iron in the nitrate reduction process. The reaction of iron nitrate with thiosulfate was analyzed by quantum chemical calculations (Figure 2C and Figure S14), which suggests that the thiosulfate anion undergoes single-electron oxidation by iron nitrate to form Fe­(II)-species and S_2_O_3_^**·**–^. The formed thiosulfate radical anion is able to initiate an oxylanion radical (·O^–^) transfer from nitrate to yield NO_2_ and a constitutional isomer (^−^OS–SO_3_^–^) of the symmetrical dithionite dianion (S_2_O_4_^2–^). We propose that S_2_O_4_^2–^ further reacts with water to form sulfite and thiosulfate as previously reported.^37^ By mass spectrometry analysis of the Fe­(NO_3_)_3_·9H_2_O and Na_2_S_2_O_3_·5H_2_O mixture after the formation of NO_2_, sulfate, sulfite, and Fe­(II) species were detected (Figures S5–S6). Nitrate reduction with iron nitrate at 25 °C allows lowering of the overall reaction temperature and therefore increases the amount of dissolved SO_2_. When the deaminative chlorosulfonylation was carried out with iron nitrate and thiosulfate at 25 °C, 67% of the desired sulfonyl chloride **1** was obtained, with only 6% of the concomitant aryl chloride **2** (Figure 2A). We propose that the deaminative chlorosulfonylation proceeds via aryldiazonium salts as fleeting intermediates (Figure 2C). NO_2_ formed via the nitrate reduction process can dimerize to form N_2_O_4_, which subsequently disproportionates to generate nitrosonium cation (NO^+^) and nitrate, as previously described.^8,38^ Diazonium salts are then generated by reaction of aniline with NO^+^ following the conventional diazotization mechanism.^39^ The aryldiazonium reacts with CuCl, SO_2_, and HCl to form the sulfonyl chloride via initial formation of an aryl radical under the release of dinitrogen.^13^ When the deaminative chlorosulfonylation was carried out with 2-(allyloxy)­aniline as radical trap, the respective radical cyclization product was detected, which is consistent with a radical mechanism operation (Figure S7). Additionally, control experiments were carried out in the absence of CuCl or HCl, which resulted in significantly lower yields of sulfonyl chloride when 4-aminobenzonitrile was used as substrate (21% yield without CuCl, 46% yield without HCl, Table S16), further supporting the mechanism postulated by Meerwein.^13^ Nitrate reduction can thus be used for the direct generation of aryl radicals from anilines.

**Figure 2:**
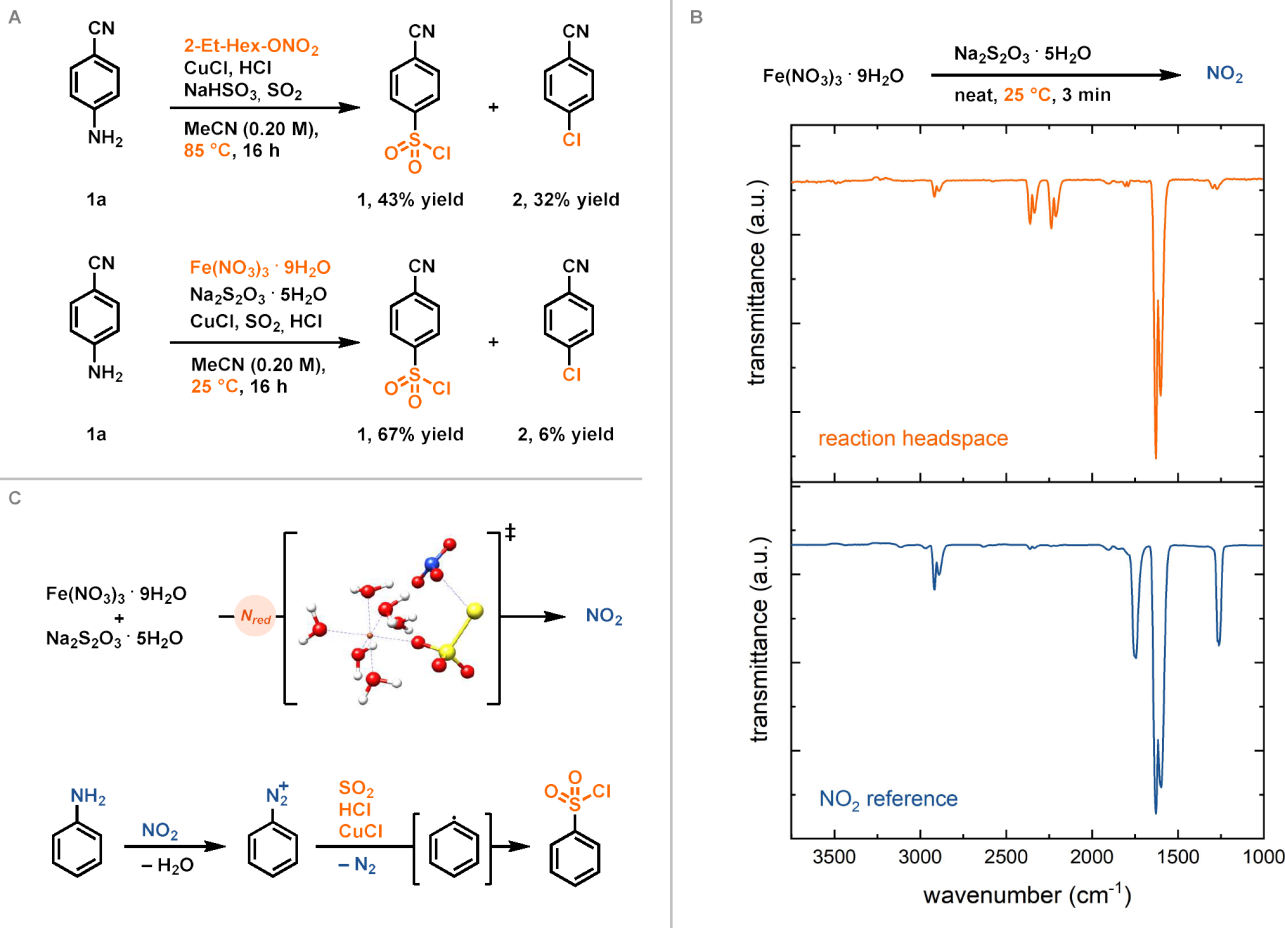
Mechanism of the deaminative chlorosulfonylation. (A) Deaminative chlorosulfonylation with nitrate ester at 85 °C vs with iron nitrate at 25 °C; yields determined by ^1^H NMR spectroscopy. (B) Nitrate reduction with thiosulfate at 23 °C, detection of NO_2_ by gas-phase IR spectroscopy. (C) Mechanistic overview for deaminative sulfonylation; transition state of nitrate reduction calculated at the M06-2X-D3(0)/ma-def2-TZVP level of theory.

Reaction optimization with 4-aminobenzonitrile **1a** (Tables S1–S5) revealed that a combination of 1.0 equiv iron nitrate with 1.0 equiv Na_2_S_2_O_3_·5H_2_O in the presence of 1.0 equiv CuCl and 2.0 equiv HCl in 1,4-dioxane with an excess of SO_2_ (10 equiv) in MeCN leads to the formation of sulfonyl chlorides in high yield. An excess of SO_2_ was crucial, due to competing direct chlorination by conventional Sandmeyer chlorination (Table S3). Stoichiometric iron nitrate was employed; reducing cofactors that may be considered^6^ are often more expensive than iron nitrate and could interfere with the reaction chemistry. The development of a similar reaction with iron as a catalyst and an inexpensive cofactor for reduction is an exciting future challenge. CuCl can be used as a catalyst (Table S4), also on a larger scale. However, stoichiometric CuCl delivered higher yields for a broader range of substrates. For the chlorosulfonylation, HCl in 1,4-dioxane can be substituted with TMSCl (Table S5). At 25 °C reaction temperature, reproducibility issues were encountered regarding the yield for the chlorosulfonylation to **1**. The deaminative chlorosulfonylation gives desired sulfonyl chloride **1** in 67% yield at 25 °C (Table S13). It was empirically found that increasing the temperature to 40 °C resolved the reproducibility issues and enabled slightly higher yields for a broader range of substrates while avoiding higher temperatures at which direct Sandmeyer chlorination becomes problematic.

The produced sulfonyl chlorides can be further converted to other sulfonic acid derivatives by the subsequent addition of nucleophiles (Figure 3). Purification of the sulfonyl chloride after deaminative chlorosulfonylation is not required. However, aqueous workup before addition of the nucleophile is necessary to achieve satisfactory yields (for **1a**, 81% with aqueous workup before amine addition, 11% without aqueous workup, Tables S3 and S15) presumably due to interactions between the nucleophile and iron or copper salts in the reaction mixture when no aqueous workup is carried out before nucleophile addition. For example, flutamide-derived aniline **3** was converted into secondary and tertiary sulfonamides **6** and **7** by the addition of primary and secondary amines in the absence of additional base, respectively (Figure 2). When ammonia is employed as a nucleophile, **3** was converted to the respective primary sulfonamide **5**, formally inserting a sulfonyl group into the C–N bond of **3**. A similar process was recently described by the Levin group utilizing isodiazene intermediates.^40^ With CsF, **3** was converted to the respective sulfonyl fluoride **8**, demonstrating that anilines can be used as direct precursors for the SuFEx reagents. When water is added, sulfonyl chloride **4** can be hydrolyzed to the respective sulfonic acid **9**.

**Figure 3:**
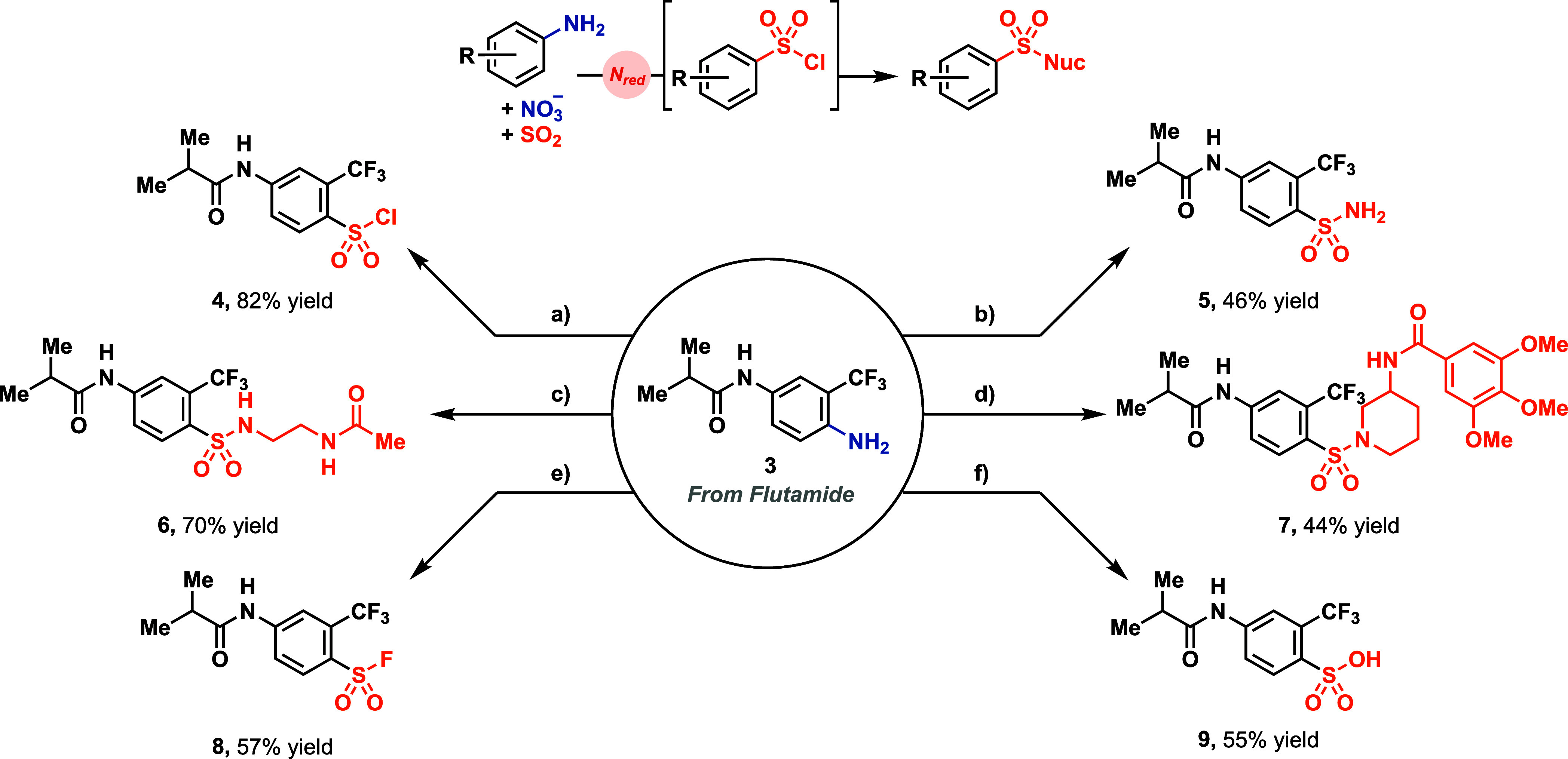
Different transformations for diversification after deaminative sulfonylation. Standard conditions (SC): 0.500 mmol reduced flutamide **1**, 0.500 mmol Na_2_S_2_O_3_·5H_2_O, 0.500 mmol Fe­(NO_3_)_3_·9H_2_O, 0.500 mmol CuCl, 2.5 mL MeCN, 1.0 mmol HCl (4.0 M in 1,4-dioxane), 5.00 mmol SO_2_ (3.00 M in MeCN) at 40 °C for 16 h; (a) SC; (b) SC then 2.00 mmol NH_3_ (0.50 M in 1,4-dioxane) at 23 °C for 1 h; (c) SC then 2.00 mmol *N*-acetylethylenediamine at 23 °C for 1 h; (d) SC then 0.500 mmol Troxipid and 0.500 mmol K_2_CO_3_ at 23 °C for 2 h; (e) SC then 1.00 mmol CsF at 23 °C for 1 h; (f) SC then 10 equiv H_2_O and 2.0 equiv K_2_CO_3_ at 85 °C for 16 h.

The iron nitrate reduction proceeds with electron-rich, electron-neutral, and electron-deficient anilines; also, amino heterocycles were converted into the respective sulfonyl chlorides. Due to the ease of purification, the products were isolated as the pyrrolidine-derived sulfonamides (Figure 4). Substrates for which the isolation or accumulation of the respective diazonium salt would be problematic, such as heterocycles (**13**, **29**, and **33**),^41^ sterically hindered anilines (**24**),^42^ or complex anilines (**32**, and **34**),^8^ were functionalized using the iron nitrate reduction strategy. Anilines of which the corresponding diazonium salts are explosive,^8^ such as nitroarenes (**16**), pyridines (**13**), or benzoic acid derivatives (**15**), can be functionalized, which demonstrates a safer access to the sulfonyl chlorides compared to the functionalization of isolated or accumulated diazonium salts.

**Figure 4:**
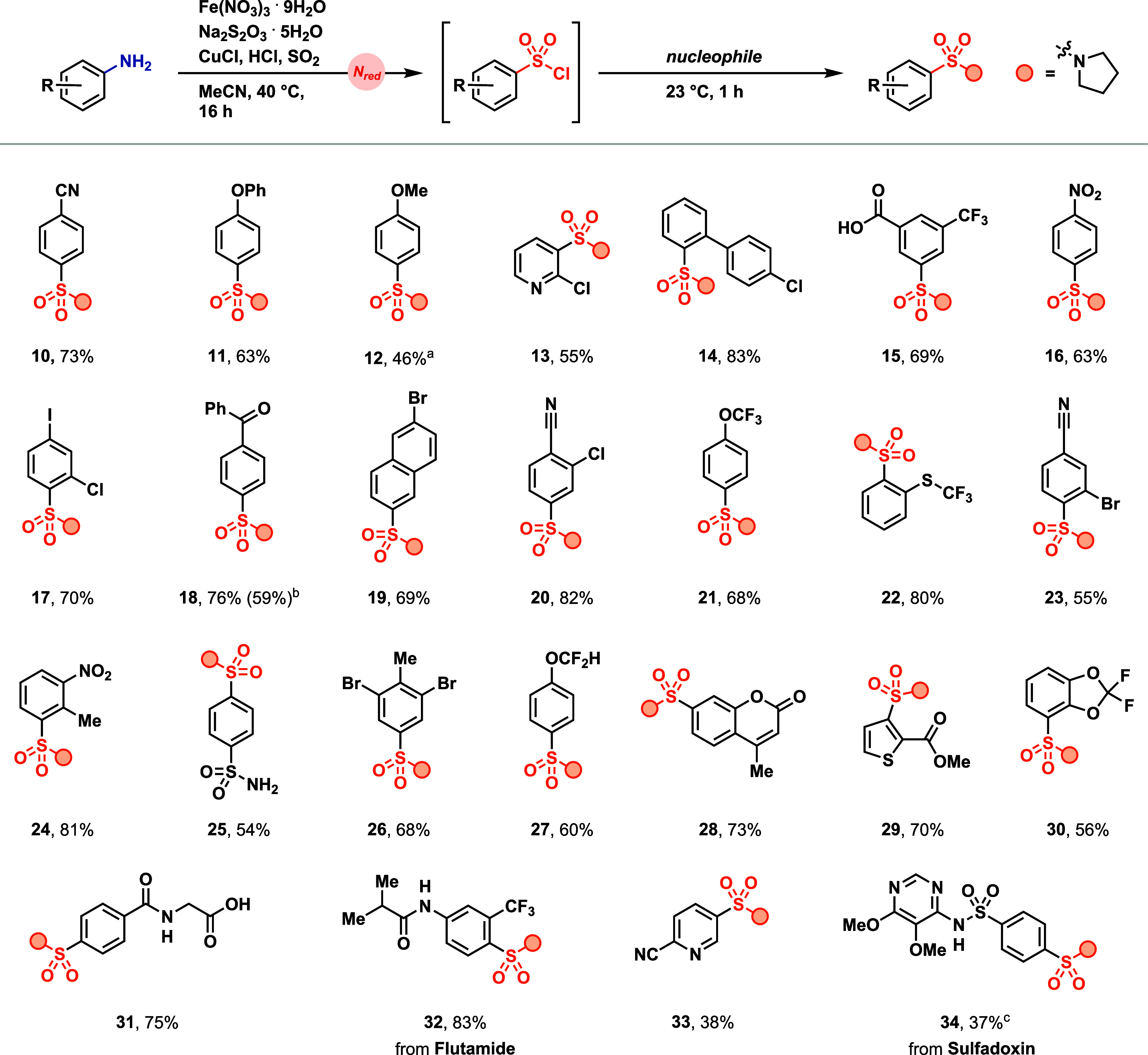
Aniline scope for deaminative chlorosulfonylation. Products isolated as sulfonamides with pyrrolidine as nucleophile. Reaction conditions = 0.500 mmol aniline/amino heterocycle, 0.500 mmol Na_2_S_2_O_3_·5H_2_O, 0.500 mmol Fe­(NO_3_)_3_·9H_2_O, 0.500 mmol CuCl, 2.5 mL MeCN, 1.0 mmol HCl (4.0 M in 1,4-dioxane), 5.00 mmol SO_2_ (3.00 M in MeCN) at 40 °C for 16 h; then 2.00 mmol pyrrolidine at 23 °C for 1 h. ^a^Isolated on a 0.1 mmol scale. ^b^Isolated on 10 mmol scale. ^c^Was isolated as sulfonyl chloride.

We observed that electron-deficient substrates generally result in facile sulfonyl chloride formation, while complex anilines that contain electron-donating groups typically give yields <30%. The deaminative chlorosulfonylation was scaled up to 10 mmol to give **18** in 59% yield (compared to 76% yield on a 0.5 mmol scale, Figure 4).

We previously demonstrated nitrate-based deaminative halogenation reactions,^8^ which remained limited to chlorination, bromination, and iodination. The discovery of the beneficial effect of iron for nitrate reduction allowed for preliminary data to now also include the remaining halide. Deaminative fluorination of anilines was achieved and demonstrated for several different aniline substrates (Figure 5), further underlining the utility of the iron nitrate-based protocol. The deaminative fluorination was carried out on sterically hindered anilines (**36**) and amino heterocycles (**39**) (Figure 5). We propose that the Fe-mediated nitrate reduction proceeds analogously to the deaminative sulfonylation reaction, producing NO_2_, which can generate an aryldiazonium intermediate. The resulting diazonium salt can subsequently react with SbF_6_^–^ by following a conventional Balz-Schiemann mechanism. Change to other polyfluorinated anions, such as BF_4_^–^ or PF_6_^–^, resulted in lower yields.

**Figure 5:**
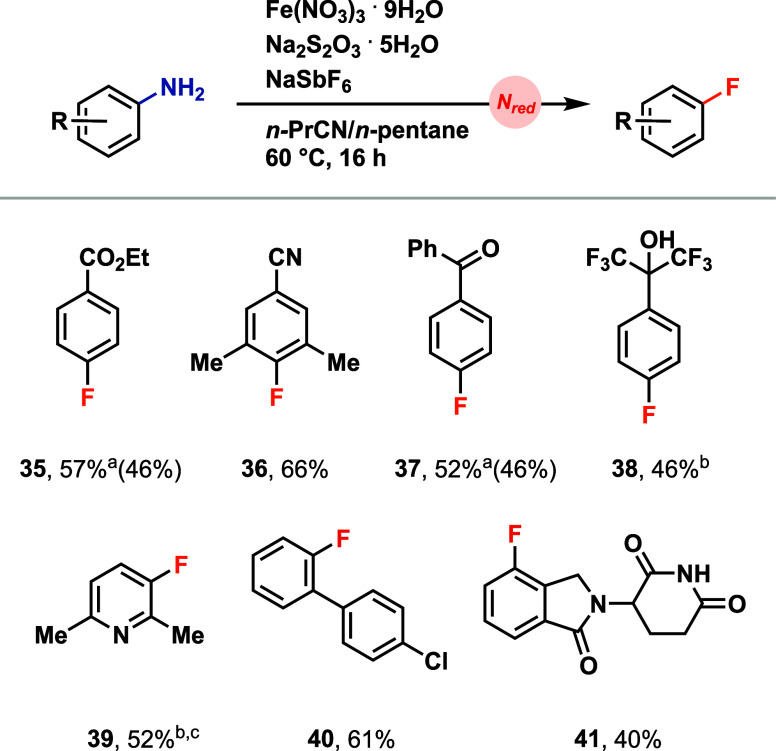
Aniline scope for the direct deaminative fluorination. Reaction conditions = 0.500 mmol aniline/amino heterocycle, 0.600 mmol Na_2_S_2_O_3_·5H_2_O, 1.50 mmol NaSbF_6_, 1.00 mmol Fe­(NO_3_)_3_·9H_2_O, 0.625 mL of *n*-PrCN, 0.625 mL of *n*-pentane, at 60 °C for 16 h (a) isolated on 0.1 mmol scale (b) yield determined by ^1^H NMR spectroscopy due to the volatility of compound (c) AgSbF_6_ used instead of NaSbF_6_.

When aqueous HCl was used instead of HCl in organic solvents, low yields of sulfonyl chloride were obtained (Table S14). With a large excess of water present, anilines are directly converted to respective sulfonic acids. While the utilization of KNO_3_ did not allow a facile deaminative chlorosulfonylation in the absence of iron, sulfonic acids can be obtained with KNO_3_. Further optimization of the conditions showed that a combination of inexpensive KNO_3_ (12.8€ per kg, Carl Roth), Na_2_S_2_O_5_ (9.0€ per kg, Carl Roth), and aqueous HCl results in a high-yielding deaminative sulfonic acid synthesis in refluxing MeCN (Tables S8–S11). We propose that nitrate is reduced by sulfur dioxide in the absence of iron at 85 °C to give NO_2_.^43^ SO_2_ is generated by thermal fragmentation of S_2_O_5_^2–^ into SO_2_ and SO_3_^2–^, whereas sulfite can further decompose into another equivalent of SO_2_ and water under acidic conditions.^44^ The generated NO_2_ can subsequently convert the aniline to the respective diazonium salt, as previously described.^8^ The reduction of KNO_3_ with SO_2_ proceeds at 85 °C.^8^ Since the concentration of SO_2_ at 85 °C is sufficient to give high yields of sulfonic acids, but only results in low yields of sulfonyl chlorides, we hypothesize that the sulfonic acid formation from aryldiazoniums does not proceed by initial formation of the sulfonyl chloride with SO_2_, followed by hydrolysis with water, but rather by direct conversion of the diazonium salt to the sulfonic acid. Control experiments in the absence of HCl (or any other chloride source) delivered the sulfonic acid **44** in 42% yield (Tables S17–S18), which further supports that the deaminative sulfonic acid formation can proceed without passing through a sulfonyl chloride intermediate. Due to the absence of any copper species for the deaminative sulfonic acid synthesis, Sandmeyer chlorination as a potential side reaction is not observed even in the presence of HCl. Based on quantum chemical calculations, we propose that the sulfonic acid formation from the diazonium salt proceeds via initial reduction of the diazonium salt by sulfite, formed from the previously described fragmentation of the metabisulfite.^42^ The sulfite radical anion can subsequently react with the diazenyl radical (Ar–N_2_·) to form the sulfonic acid in a single step under the extrusion of dinitrogen (Figure S13). When the deaminative sulfonic acid synthesis was carried out in the presence of radical traps, the respective radical addition adduct was observed (Figures S6–S7).

When the deaminative sulfonylation was monitored by ^1^H NMR spectroscopy, no formation of sulfonyl chloride or other intermediates was detected in significant concentrations apart from aniline starting material and sulfonic acid reaction product (Figure S8). A significant change of the ^1^H chemical shift of the aromatic signals of the starting material and product was observed, which could result from a change in the degree of protonation of the aniline. Analysis of the reaction mixture by ^17^O NMR spectroscopy before and after heating for 18 h showed the formation of sulfate, presumably as terminal reaction product from the nitrate reduction with SO_2_ (Figure S10). By ^14^N NMR spectroscopy, the formation of nitrite or other nitrogen species was not detected, which is consistent with the formation of NO_2_ as a product of the nitrate reduction process (Figure S11). Direct conversion of electron-rich, electron-neutral, and electron-deficient anilines and amino heterocycles to the respective sulfonic acids was achieved with the KNO_3_, Na_2_S_2_O_5_, and HCl-based protocol (Figure 6). Complex anilines were tolerated, of which the corresponding diazonium salts cannot be easily isolated or accumulated (**47** and **50**).^8^ Additionally, substrates that contain functional groups that are prone to oxidation and therefore do not tolerate nitrite,^45^ such as tertiary amines, were successfully functionalized with the KNO_3_-based procedure (**49**). The simple reaction set up of mixing all solid reagents followed by addition of all liquid reagents at 25 °C provides robust access to the sulfonic acids from anilines.

**Figure 6:**
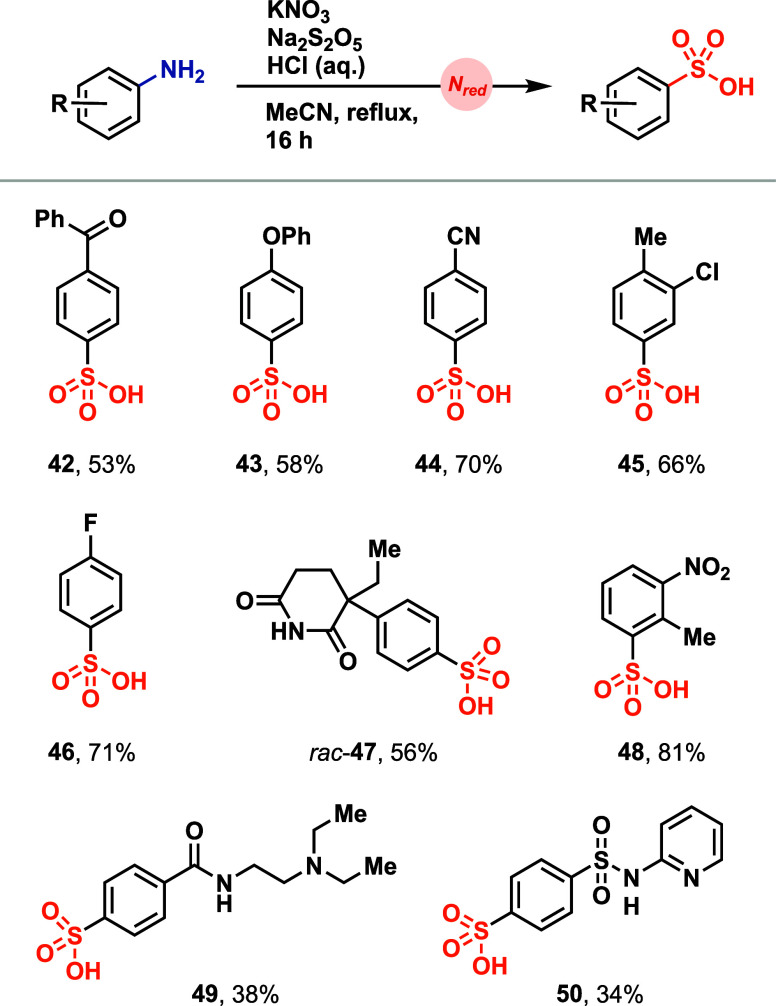
Aniline scope for direct deaminative sulfonic acid synthesis. Reaction conditions = 0.200 mmol aniline/amino heterocycle, 0.600 mmol KNO_3_, 0.500 mmol Na_2_S_2_O_5_, 0.400 mmol HCl (aq. 9.25%), and 1.0 mL of MeCN, at 85 °C for 16 h.

## Conclusions

In conclusion, we have demonstrated that inexpensive Fe­(NO_3_)_3_·9H_2_O (30.0€ per kg, Carl Roth) can generate NO_2_ through nitrate reduction with thiosulfate at room temperature (25 °C), which enables the use of the nitrate reduction strategy for the *in situ* generation of aryl diazonium salts below 85 °C. The iron nitrate reduction was utilized for the deaminative chlorosulfonylation of a variety of structurally and electronically diverse anilines and amino heterocycles, giving access to multiple sulfur-based functional groups directly from the corresponding aromatic amines, without accumulation of the respective diazonium salt. The deaminative fluorination was presented as an additional application of Fe-mediated nitrate reduction. We envision that the iron nitrate reduction will also enable other deaminative functionalization reactions that require close to ambient temperature. Additionally, we described a deaminative sulfonic acid synthesis directly from anilines as a previously unknown transformation.

## Supplementary Material



## Notes

Experimental and computational procedures, spectroscopic data, NMR spectra of all products (PDF)

## Abbreviations

SuFEx: Sulfur­(VI) fluoride exchange
MeCN: Acetonitrile
NCS: *N*-chlorosuccinimide
NFSI: *N*-fluorobenzenesulfonimide
NMR: Nuclear magnetic resonance spectroscopy
UV–vis: Ultraviolet–visible light
DABSO: 1,4-Diazabicyclo­[2.2.2]­octane bis­(sulfur dioxide) adduct
DABCO: 1,4-Diazabicyclo­[2.2.2]­octane
NADH: Nicotinamide adenine dinucleotide
FAD: Flavin adenine dinucleotide
TMEDA: Tetramethylethylenediamine
TBA: Tetrabutylammonium
