# Supplementary material for: Iron-Mediated Nitrate Reduction at Ambient Temperature for Deaminative Sulfonylation and Fluorination of Anilines
Source: J Am Chem Soc. 2025 May 9;147(20):16901–8. doi: 10.1021/jacs.4c17981 (PMC12100724; doi:10.1021/jacs.4c17981)
Supplement: Supplementary file 1 [file ja4c17981_si_001.pdf]

SUPPLEMENTARY MATERIALS

Iron-Mediated Nitrate Reduction at Ambient Temperature for Deaminative Sulfonylation and Fluorination of Anilines

Tim Schulte,[1,‡] Deepak Behera,[1,2,‡] Davide Carboni,[1] Annika Höppner,[1,2] Felix Waldbach,[1] Javier Mateos,[1] Ahmet Altun,[1] Markus Leutzsch,[1] Moritz L. Krebs,[1] Tobias Ritter*[1]

1 Max-Planck-Institut für Kohlenforschung; Mülheim an der Ruhr, D-45470, Germany.

2 Institute of Organic Chemistry, RWTH Aachen University; Aachen, 52074, Germany.

‡ The authors contributed equally.

*Corresponding author. Email: ritter@kofo.mpg.de

TABLE OF CONTENTS

MATERIALS AND METHODS 7

EXPERIMENTAL DATA 9

General Procedures for Deaminative Sulfonylations 9

General procedure for deaminative sulfochlorination 9

General procedure for deaminative sulfochlorination (isolation as sulfonamide) 10

General procedure for deaminative fluorination 12

General procedure for deaminative sulfonic acid synthesis 13

Optimization Tables 14

Optimization of the deaminative sulfochlorination 14

Optimization of the deaminative fluorination 18

Optimization of the deaminative sulfonic acid synthesis 20

Deaminative Sulfonylations with Subsequent Derivatization 23

Deaminative sulfochlorination for sulfonyl chloride **4** 23

Deaminative sulfoamidation for sulfonamide **5** 24

Deaminative sulfoamidation for sulfonamide **6** 25

Deaminative sulfoamidation for sulfonamide **7** 26

Deaminative sulfofluorination for sulfonyl fluoride **8** 28

Deaminative sulfonic acid synthesis for sulfonic acid **9** 29

Aniline Scope for Deaminative Sulfochlorination (isolation as sulfonamides) 30

4-(Pyrrolidin-1-ylsulfonyl)benzonitrile (**10**) 30

1-((4-Phenoxyphenyl)sulfonyl)pyrrolidine (**11**) 31

1-((4-Methoxyphenyl)sulfonyl)pyrrolidine (**12**) 32

2-Chloro-3-(pyrrolidin-1-ylsulfonyl)pyridine (**13**) 33

1-((4'-Chloro-[1,1'-biphenyl]-2-yl)sulfonyl)pyrrolidine (**14**) 34

3-(Pyrrolidin-1-ylsulfonyl)-5-(trifluoromethyl)benzoic acid (**15**) 35

4-(Pyrrolidin-1-ylsulfonyl)nitrobenzene (**16**) 36

1-((2-Chloro-4-iodophenyl)sulfonyl)pyrrolidine (**17**) 37

Phenyl(4-(pyrrolidin-1-ylsulfonyl)phenyl)methanone (**18**) 38

1-((6-Bromonaphthalen-2-yl)sulfonyl)pyrrolidine (**19**) 40

2-Chloro-4-(pyrrolidin-1-ylsulfonyl)benzonitrile (**20**) 41

4-(Pyrrolidin-1-ylsulfonyl)trifluoromethoxybenzene (**21**) 42

1-((2-((Trifluoromethyl)thio)phenyl)sulfonyl)pyrrolidine (**22**) 43

3-Bromo-4-(pyrrolidin-1-ylsulfonyl)benzonitrile (**23**) 44

1-((2-Methyl-3-nitrophenyl)sulfonyl)pyrrolidine (**24**) 45

4-(pyrrolidin-1-ylsulfonyl)benzenesulfonamide (**25**) 46

1-((3,5-Dibromo-4-methylphenyl)sulfonyl)pyrrolidine (**26**) 47
4-(Pyrrolidin-1-ylsulfonyl)difluoromethoxybenzene (**27**) 48
4-Methyl-7-(pyrrolidin-1-ylsulfonyl)-2*H*-chromen-2-one (**28**) 49
Methyl 3-(pyrrolidin-1-ylsulfonyl)thiophene-2-carboxylate (**29**) 50
1-((2,2-Difluorobenzo[d][1,3]dioxol-4-yl)sulfonyl)pyrrolidine (**30**) 51
Hippuric acid-derived sulfonamide **31** 52
Flutamide-derived sulfonamide **32** 53
5-(Pyrrolidin-1-ylsulfonyl)picolinonitrile (**33**) 54
Sulfadoxin-derived sulfonyl chloride **34a** 55

Aniline Scope for Deaminative Fluorination 56

Ethyl-4-fluorobenzoate (**35**) 56
4-Fluoro-3,5-dimethylbenzonitrile (**36**) 56
(4-Fluorophenyl)(phenyl)methanone (**37**) 57
1,1,1,3,3,3-Hexafluoro-2-(4-fluorophenyl)propan-2-ol (**38**) 58
3-Fluoro-2,6-dimethylpyridine (**39**) 59
4'-Chloro-2-fluoro-1,1'-biphenyl (**40**) 60
Lenalidomide-derived fluoroarene **41** 61

Aniline Scope for Sulfonic Acid Synthesis by Deaminative Sulfonylation 63

4-Benzoylbenzenesulfonic acid (**42**) 63
4-Phenoxybenzenesulfonic acid (**43**) 64
4-Cyanobenzenesulfonic acid (**44**) 65
3-Chloro-4-methylbenzenesulfonic acid (**45**) 66
4-Fluorobenzenesulfonic acid (**46**) 67
Aminoglutethimide-derived sulfonic acid *rac*-**47** 68
2-Methyl-3-nitrobenzenesulfonic acid (**48**) 69
Procainamide-derived sulfonic acid **49** 70
Sulfapyridine-derived sulfonic acid **50** 71

Mechanistic Experiments 72

NO_2 detection with gas phase IR spectroscopy 72
NO_2 detection with UV-Vis spectroscopy 73
Mass spectrometric analysis 74
Control experiments deaminative sulfochlorination 75
Control experiments deaminative sulfonic acid synthesis 80
Radical trapping experiments 83
Reaction monitoring with NMR spectroscopy 86

COMPUTATIONAL DATA 89

Nitrate reduction with thiosulfate 89
Sulfonic acid formation from aryldiazonium salt 93

SPECTROSCOPIC DATA.......... 96
^{1}H NMR spectrum of Flutamide-derived sulfonyl chloride **4** 96
^{13}C NMR spectrum of Flutamide-derived sulfonyl chloride **4** 97
^{19}F NMR spectrum of Flutamide-derived sulfonyl chloride **4** 98
^{1}H NMR spectrum of Flutamide-derived primary sulfonamide **5** 99
^{13}C NMR spectrum of Flutamide-derived primary sulfonamide **5** 100
^{19}F NMR spectrum of Flutamide-derived primary sulfonamide **5** 101
^{1}H NMR spectrum of Flutamide-derived secondary sulfonamide **6** 102
^{13}C NMR spectrum of Flutamide-derived secondary sulfonamide **6** 103
^{19}F NMR spectrum of Flutamide-derived secondary sulfonamide **6** 104
^{1}H NMR specrum of Flutamide-derived tertiary sulfonamide **7** 105
^{13}C NMR spectrum of Flutamide-derived tertiary sulfonamide **7** 106
^{19}F NMR spectrum of Flutamide-derived tertiary sulfonamide **7** 107
^{1}H NMR spectrum of Flutamide-derived sulfonyl fluoride **8** 108
^{13}C NMR spectrum of Flutamide-derived sulfonyl fluoride **8** 109
^{19}F NMR spectrum of Flutamide-derived sulfonyl fluoride **8** 110
^{1}H NMR spectrum of Flutamide-derived sulfonic acid **9** 111
^{13}C NMR spectrum of Flutamide-derived sulfonic acid **9** 112
^{19}F NMR spectrum of Flutamide-derived sulfonic acid **9** 113
^{1}H NMR spectrum of 4-(pyrrolidin-1-ylsulfonyl)benzonitrile (**10**) 114
^{13}C NMR spectrum of 4-(pyrrolidin-1-ylsulfonyl)benzonitrile (**10**) 115
^{1}H NMR spectrum of 1-((4-phenoxyphenyl)sulfonyl)pyrrolidine (**11**) 116
^{13}C NMR spectrum of 1-((4-phenoxyphenyl)sulfonyl)pyrrolidine (**11**) 117
^{1}H NMR spectrum of 1-((4-methoxyphenyl)sulfonyl)pyrrolidine (**12**) 118
^{13}C NMR spectrum of 1-((4-methoxyphenyl)sulfonyl)pyrrolidine (**12**) 119
^{1}H NMR spectrum of 2-chloro-3-(pyrrolidin-1-ylsulfonyl)pyridine (**13**) 120
^{13}C NMR spectrum of 2-chloro-3-(pyrrolidin-1-ylsulfonyl)pyridine (**13**) 121
^{1}H NMR spectrum of 1-((4'-chloro-[1,1'-biphenyl]-2-yl)sulfonyl)pyrrolidine (**14**) 122
^{13}C NMR spectrum of 1-((4'-chloro-[1,1'-biphenyl]-2-yl)sulfonyl)pyrrolidine (**14**) 123
^{1}H NMR spectrum of 3-(pyrrolidin-1-ylsulfonyl)-5-(trifluoromethyl)benzoic acid (**15**) 124

^{13}C NMR spectrum of 3-(pyrrolidin-1-ylsulfonyl)-5-(trifluoromethyl)benzoic acid (**15**) 125

^{19}F NMR spectrum of 3-(pyrrolidin-1-ylsulfonyl)-5-(trifluoromethyl)benzoic acid (**15**) 126

^{1}H NMR spectrum of 4-(pyrrolidin-1-ylsulfonyl)nitrobenzene (**16**) 127

^{13}C NMR spectrum of 4-(pyrrolidin-1-ylsulfonyl)nitrobenzene (**16**) 128

^{1}H NMR spectrum of 1-((2-chloro-4-iodophenyl)sulfonyl)pyrrolidine (**17**)..................................... 129

^{13}C NMR spectrum of 1-((2-chloro-4-iodophenyl)sulfonyl)pyrrolidine (**17**) 130

^{1}H NMR spectrum of phenyl(4-(pyrrolidin-1-ylsulfonyl)phenyl)methanone (**18**) 131

^{13}C NMR spectrum of phenyl(4-(pyrrolidin-1-ylsulfonyl)phenyl)methanone (**18**)........................... 132

^{1}H NMR spectrum of 1-((6-bromonaphthalen-2-yl)sulfonyl)pyrrolidine (**19**) 133

^{13}C NMR spectrum of 1-((6-bromonaphthalen-2-yl)sulfonyl)pyrrolidine (**19**)................................ 134

^{1}H NMR spectrum of 2-chloro-4-(pyrrolidin-1-ylsulfonyl)benzonitrile (**20**) 135

^{13}C NMR spectrum of 2-chloro-4-(pyrrolidin-1-ylsulfonyl)benzonitrile (**20**).................................. 136

^{1}H NMR spectrum of 4-(pyrrolidin-1-ylsulfonyl)trifluoromethoxybenzene (**21**) 137

^{13}C NMR spectrum of 4-(pyrrolidin-1-ylsulfonyl)trifluoromethoxybenzene (**21**) 138

^{19}F NMR spectrum of 4-(pyrrolidin-1-ylsulfonyl)trifluoromethoxybenzene (**21**) 139

^{1}H NMR spectrum of 1-((2-((trifluoromethyl)thio)phenyl)sulfonyl)pyrrolidine (**22**) 140

^{13}C NMR spectrum of 1-((2-((trifluoromethyl)thio)phenyl)sulfonyl)pyrrolidine (**22**)........................ 141

^{19}F NMR spectrum of 1-((2-((trifluoromethyl)thio)phenyl)sulfonyl)pyrrolidine (**22**) 142

^{1}H NMR spectrum of 3-bromo-4-(pyrrolidin-1-ylsulfonyl)benzonitrile (**23**).................................... 143

^{13}C NMR spectrum of 3-bromo-4-(pyrrolidin-1-ylsulfonyl)benzonitrile (**23**) 144

^{1}H NMR spectrum of 1-((2-methyl-3-nitrophenyl)sulfonyl)pyrrolidine (**24**).................................... 145

^{13}C NMR spectrum of 1-((2-methyl-3-nitrophenyl)sulfonyl)pyrrolidine (**24**) 146

^{1}H NMR spectrum of 4-(pyrrolidin-1-ylsulfonyl)benzenesulfonamide (**25**).................................... 147

^{13}C NMR spectrum of 4-(pyrrolidin-1-ylsulfonyl)benzenesulfonamide (**25**) 148

^{1}H NMR spectrum of 1-((3,5-dibromo-4-methylphenyl)sulfonyl)pyrrolidine (**26**)............................ 149

^{13}C NMR spectrum of 1-((3,5-dibromo-4-methylphenyl)sulfonyl)pyrrolidine (**26**) 150

^{1}H NMR spectrum of 4-(pyrrolidin-1-ylsulfonyl)difluoromethoxybenzene (**27**).............................. 151

^{13}C NMR spectrum of 4-(pyrrolidin-1-ylsulfonyl)difluoromethoxybenzene (**27**) 152

^{19}F NMR spectrum of 4-(pyrrolidin-1-ylsulfonyl)difluoromethoxybenzene (**27**).............................. 153

^{1}H NMR spectrum of 4-methyl-7-(pyrrolidin-1-ylsulfonyl)-2*H*-chromen-2-one (**28**) 154

^{13}C NMR spectrum of 4-methyl-7-(pyrrolidin-1-ylsulfonyl)-2*H*-chromen-2-one (**28**) 155

^{1}H NMR spectrum of methyl 3-(pyrrolidin-1-ylsulfonyl)thiophene-2-carboxylate (**29**) 156

^{13}C NMR spectrum of methyl 3-(pyrrolidin-1-ylsulfonyl)thiophene-2-carboxylate (**29**) 157

^{1}H NMR spectrum of 1-((2,2-difluorobenzo[d][1,3]dioxol-4-yl)sulfonyl)pyrrolidine (**30**) 158

^{13}C NMR spectrum of 1-((2,2-difluorobenzo[d][1,3]dioxol-4-yl)sulfonyl)pyrrolidine (**30**)................ 159

^{19}F NMR spectrum of 1-((2,2-difluorobenzo[d][1,3]dioxol-4-yl)sulfonyl)pyrrolidine (**30**) 160

^{1}H NMR spectrum of hippuric acid-derived sulfonamide **31** 161

^{13}C NMR spectrum of hippuric acid-derived sulfonamide **31** 162

^{1}H NMR spectrum of Flutamide-derived sulfonamide **32**... 163

^{13}C NMR spectrum of Flutamide-derived sulfonamide **32** 164

^{19}F NMR spectrum of Flutamide-derived sulfonamide **32**.. 165

^{1}H NMR spectrum of 5-(pyrrolidin-1-ylsulfonyl)picolinonitrile (**33**) 166

^{13}C NMR spectrum of 5-(pyrrolidin-1-ylsulfonyl)picolinonitrile (**33**).. 167

^{1}H NMR spectrum of Sulfadoxin-derived sulfonyl chloride (**34a**).. 168

^{13}C NMR spectrum of Sulfadoxin-derived sulfonyl chloride (**34a**) 169

^{1}H NMR spectrum of ethyl-4-fluorobenzoate (**35**)... 170

^{13}C NMR spectrum of ethyl-4-fluorobenzoate (**35**) 171

^{19}F NMR spectrum of ethyl-4-fluorobenzoate (**35**).. 172

^{1}H NMR spectrum of 4-fluoro-3,5-dimethylbenzonitrile (**36**)... 173

^{13}C NMR spectrum of 4-fluoro-3,5-dimethylbenzonitrile (**36**).. 174

^{19}F NMR spectrum of 4-fluoro-3,5-dimethylbenzonitrile (**36**) 175

^{1}H NMR spectrum of (4-fluorophenyl)(phenyl)methanone (**37**) 176

^{13}C NMR spectrum of (4-fluorophenyl)(phenyl)methanone (**37**)... 177

^{19}F NMR spectrum of (4-fluorophenyl)(phenyl)methanone (**37**) 178

^{1}H NMR spectrum of 4'-chloro-2-fluoro-1,1'-biphenyl (**40**).. 179

^{13}C NMR spectrum of 4'-chloro-2-fluoro-1,1'-biphenyl (**40**)... 180

^{19}F NMR spectrum of 4'-chloro-2-fluoro-1,1'-biphenyl (**40**)... 181

^{1}H NMR spectrum of Lenalidomide-derived fluoroarene **41** 182

^{13}C NMR spectrum of Lenalidomide-derived fluoroarene **41**.. 183

^{19}F NMR spectrum of Lenalidomide-derived fluoroarene **41** 184

^{1}H NMR spectrum of 4-benzoylbenzenesulfonic acid (**42**) 185

^{13}C NMR spectrum of 4-benzoylbenzenesulfonic acid (**42**) 186

^{1}H NMR spectrum of 4-phenoxybenzenesulfonic acid (**43**) 187

^{13}C NMR spectrum of 4-phenoxybenzenesulfonic acid (**43**) 188

^{1}H NMR spectrum of 4-cyanobenzenesulfonic acid (**44**) 189

^{13}C NMR spectrum of 4-cyanobenzenesulfonic acid (**44**) 190

^{1}H NMR spectrum of 3-chloro-4-methylbenzenesulfonic acid (**45**) 191

^{13}C NMR spectrum of 3-chloro-4-methylbenzenesulfonic acid (**45**) 192

^{1}H NMR spectrum of 4-fluorobenzenesulfonic acid (**46**) 193

^{13}C NMR spectrum of 4-fluorobenzenesulfonic acid (**46**) 194

^{19}F NMR spectrum of 4-fluorobenzenesulfonic acid (**46**) 195

^{1}H NMR spectrum of Aminoglutethimide-derived sulfonic acid *rac*-**47** 196

^{13}C NMR spectrum of Aminoglutethimide-derived sulfonic acid *rac*-**47** 197

^{1}H NMR spectrum of 2-methyl-3-nitrobenzenesulfonic acid (**48**) 198

^{13}C NMR spectrum of 2-methyl-3-nitrobenzenesulfonic acid (**48**) 199

^{1}H NMR spectrum of Procainamide-derived sulfonic acid **49** 200

^{13}C NMR spectrum of Procainamide-derived sulfonic acid **49** 201

^{1}H NMR spectrum of Sulfapyridine-derived sulfonic acid **50** 202

^{13}C NMR spectrum of Sulfapyridine-derived sulfonic acid **50** 203

REFERENCES 204

MATERIALS AND METHODS

All air- and moisture-insensitive reactions were carried out under ambient atmosphere and monitored by thin-layer chromatography (TLC). High-resolution mass spectra were obtained using *Q Exactive Plus* from *Thermo*. Concentration under reduced pressure was performed by rotary evaporation at 25–40°C at an appropriate pressure. Purified compounds were further dried under high vacuum (0.010–0.005 mbar). Yields refer to purified and spectroscopically pure compounds, unless otherwise stated.

Solvents

Acetonitrile was purchased from Fisher Scientific. Anhydrous solvents were obtained from Phoenix Solvent Drying Systems. All deuterated solvents were purchased from Euriso-Top.

Chromatography

Thin layer chromatography (TLC) was performed using EMD TLC plates pre-coated with 250 µm thickness silica gel 60 F254 plates and visualized by fluorescence quenching under 254 nm UV light or permanganate stain. Flash chromatography was performed using silica gel (40–63 µm particle size) purchased from Geduran®.

NMR Spectroscopy

NMR spectra were recorded on a Bruker AVANCE III HD 500 spectrometer operating at 500 MHz, 471 MHz, and 126 MHz, for ^{1}H, ^{19}F, and ^{13}C acquisitions, respectively; a Bruker AVANCE NEO 600 spectrometer equipped with a cryogenically cooled cryoBBO probe operating at 600 MHz and 61 MHz for ^{1}H and ^{15}N acquisitions, respectively; or on a Bruker AVANCE III HD 400 spectrometer operating at 400 MHz, 100 MHz, 29 MHz, and 54 MHz for ^{1}H, ^{13}C, ^{14}N, and ^{17}O acquisitions, respectively. ^{1}H and ^{13}C chemical shifts are reported in ppm with the solvent residual peak as the internal standard. For ^{1}H NMR: $CDCl_3$, δ 7.26; DMSO-d_6, δ 2.50; CD_3CN, δ 1.94; For ^{13}C NMR: $CDCl_3$, δ 77.2; DMSO-d_6, δ 39.5; CD_3CN, δ 1.3. Data is reported as follows: s = singlet, d = doublet, t = triplet, q = quartet, p = pentet, m = multiplet, br = broad; coupling constants in Hz; integration (*56*). ^{14}N and ^{15}N shifts are reported relative to $MeNO_2$ (δ = 0.0 ppm). ^{17}O shifts are reported relative to D_2O (δ = 0.0 ppm). NMR shifts of heteronuclei were referenced using their respective Ξ value using the *xiref* au program in Bruker Topspin following IUPAC recommendations.

IR Spectroscopy

The gas phase was analyzed by IR spectroscopy using a Thermo Scientific Nicolet Avatar 370 FT-IR spectrometer, with 42 scans per spectrum and a spectral resolution of 2 cm^{-1}. Before each measurement, a background spectrum was recorded, which was subtracted from the actual measurement.

Computational details

All calculations were performed using the ORCA program package, version 6.0.[1-2] Geometry optimizations, transition-state location, and enthalpy computations, along with free energy computations at standard conditions (298.15 K and 1 atm), were performed using the M06-2X-D3(0) density functional[3] and the ma-def2-TZVP basis set,[4–5] i.e., at the M06-2X-D3(0)/ma-def2-TZVP level. Electronic energies were refined with the ma-def2-QZVP basis set, corresponding to the M06-2X-D3(0)/ma-def2-QZVP//M06-2X-D3(0)/ma-def2-TZVP level. The effect of the solvent (acetonitrile) on both geometries and energies was incorporated implicitly using the Solvation Model based on Density (SMD) scheme.[6]

Reagents and starting materials

All reagents were used as received from commercial suppliers. KNO_3, $Na_2S_2O_5$, and pyrrolidine were obtained from *Sigma-Aldrich*; $Na_2S_2O_3$ $5H_2O$ and HCl (4M in 1,4-dioxane) were obtained from *Thermo Scientific*; $Fe(NO_3)_3$ $9H_2O$ was purchased from *Alfa Aesar*; CuCl was obtained from *TCI*, HCl (37% aq.) was obtained from *J.T. Baker*; SO_2 (gas) was obtained from *Air Liquide*, and MeCN was purchased from *Fisher Scientific*. All other reagents and substrates were used as received from commercial suppliers, such as *Sigma-Aldrich*, *TCI*, *Alfa Aesar, BLDPharm, or ChemImpex*.

Safety statement

The procedures reported in this work are intended for use only by individuals with proper training in experimental chemistry. All hazardous materials (solid, liquid, or gaseous) should be handled using the standard work procedures described in references such as Chapter 4 of "Prudent Practices in the Laboratory".[7] All chemical waste should be disposed of in accordance with local regulations. For general guidelines for the management of chemical waste, see Chapter 8 of "Prudent Practices in the Laboratory".[7] Reaction set-up, and chemical-specific hazards are highlighted in bold with "**Caution:**" notes in the procedures reported in these supplementary materials. It is important to note that the absence of a caution note does not imply that no significant hazards are associated with the chemicals involved in that procedure.

During the course of this study no explosions or violent decompositions occurred. A summary of the possible risks and hazards is described below:

Caution: When performing reactions in pressurized systems (such as closed vials, pressure tubes, and autoclaves), a blast shield must be used to minimize personal damage in case of an accident.

EXPERIMENTAL DATA

General Procedures for Deaminative Sulfonylations

General procedure for deaminative sulfochlorination

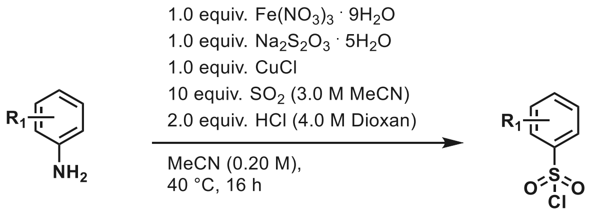

Caution: When performing reactions in pressurized vessels (such as closed vials, pressure tubes, and autoclaves), a blast shield must be used to minimize personal damage in case of an accident. See safety statement on page S8.

0.5 mmol Scale: Under an ambient atmosphere, to a 20-mL borosilicate vial equipped with a Teflon-coated magnetic stir bar were added the (hetero)aromatic amine (if solid, 0.500 mmol, 1.00 equiv.), $Na_2S_2O_3$ $5H_2O$ (124 mg, 0.500 mmol, 1.00 equiv.) and CuCl (49.5 mg, 0.500 mmol, 1.00 equiv.). Then, acetonitrile (2.5 mL), (hetero)aromatic amine (if liquid, 0.500 mmol, 1.00 equiv.), HCl (4.0 M in 1,4-dioxane, 0.25 mL, 1.0 mmol, 2.0 equiv.), and SO_2 (3.0 M in MeCN, 1.7 mL, 5.0 mmol, 10 equiv.) were added, the vial was quickly sealed with a septum cap and the mixture stirred for 5 min at 23 °C. Subsequently, iron(III) nitrate nonahydrate (202 mg, 0.500 mmol, 1.00 equiv.) was added to the suspension and the vial was quickly resealed with a septum cap. The mixture was then stirred at 40 °C for 18 h. After cooling to 23 °C, the resulting mixture was concentrated by rotary evaporation under reduced pressure. The residue was diluted with water (5 mL) and extracted with DCM (3 × 5 mL). The organic layers were combined, the solvent was removed under reduced pressure, and the residue dissolved in $CDCl_3$ (2.0 mL). Dibromomethane was added as internal standard (34.9 µL, 86.9 mg, 0.500 mmol, 1.00 equiv.). The mixture was filtered and a 0.5 mL aliquot of the solution used to record a ^{1}H-NMR spectrum. The integration of the CH_2Br_2 signal (s, 5.0 ppm) was compared to the integration of a signal corresponding to an aromatic proton of the respective sulfonyl chloride (between 6.0–8.0 ppm) to determine the yield.

General procedure for deaminative sulfochlorination (isolation as sulfonamide)

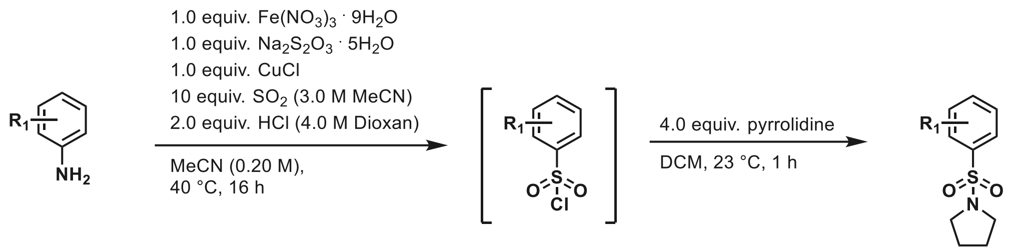

Caution: When performing reactions in pressurized vessels (such as closed vials, pressure tubes, and autoclaves), a blast shield must be used to minimize personal damage in case of an accident. See safety statement on page S8.

0.5 mmol Scale: Under an ambient atmosphere, to a 20-mL borosilicate vial equipped with a Teflon-coated magnetic stir bar were added the (hetero)aromatic amine (if solid, 0.500 mmol, 1.00 equiv.), $Na_2S_2O_3$ $5H_2O$ (124 mg, 0.500 mmol, 1.00 equiv.) and CuCl (49.5 mg, 0.500 mmol, 1.00 equiv.). Then, acetonitrile (2.5 mL), (hetero)aromatic amine (if liquid, 0.500 mmol, 1.00 equiv.), HCl (4.0 M in 1,4-dioxane, 0.25 mL, 1.0 mmol, 2.0 equiv.), and SO_2 (3.0 M in MeCN, 1.7 mL, 5.0 mmol, 10.0 equiv.) were added, the vial was quickly sealed with a septum cap and the mixture stirred for 5 min at 23 °C. Subsequently, iron(III) nitrate nonahydrate (202 mg, 0.500 mmol, 1.00 equiv.) was added to the suspension and the vial was quickly resealed with a septum cap. The mixture was then stirred at 40 °C for 18 h. After cooling to 23 °C, the resulting mixture was concentrated by rotary evaporation under reduced pressure. The residue was diluted with water (5 mL) and extracted with DCM (3 × 5 mL). The organic layers were combined in a 20-mL borosilicate vial and a Teflon-coated magnetic stir bar was added, followed by pyrrolidine (165 µL, 142 mg, 2.00 mmol, 4.00 equiv.). The vessel was sealed with a septum cap and the mixture stirred for 1 h at 23 °C. Subsequently water (5 mL) was added, the aqueous phase decanted and further extracted with EtOAc (3 x 5 mL). The combined organic layers were dried over $MgSO_4$, filtered, and the solvent evaporated under reduced pressure. To obtain analytically pure samples of the sulfonamides, the residue was purified by chromatography on silica gel.

10 mmol Scale: Under an ambient atmosphere, to a 350-mL Ace pressure tube equipped with a Teflon-coated magnetic stir bar were added the (hetero)aromatic amine (if solid, 10.0 mmol, 1.00 equiv.), $Na_2S_2O_3$ $5H_2O$ (2.48 g, 10.0 mmol, 1.00 equiv.) and CuCl (990 mg, 10.0 mmol, 1.00 equiv.). Then, acetonitrile (50 mL), (hetero)aromatic amine (if liquid, 10.0 mmol, 1.00 equiv.), HCl (4.0 M in 1,4-dioxane, 5.0 mL, 20 mmol, 2.0 equiv.), and SO_2 (3.0 M in MeCN, 32 mL, 0.10 mol, 10 equiv.) were added, the vessel was quickly sealed and the mixture stirred for 5 min at 23 °C. Subsequently, iron(III) nitrate nonahydrate (4.04 g, 10.0 mmol, 1.00 equiv.) was added to the suspension and the pressure tube was quickly resealed. A blast shield was placed in front of the set up and the mixture was then stirred at 40 °C for 18 h. After cooling to 23 °C, the resulting mixture was transferred to a 100 mL round-bottomed flask and concentrated by rotary evaporation under reduced pressure. The residue was diluted with water (50 mL) and extracted with EtOAc (3 × 75 mL). The organic layers were concentrated in a 250 mL round-bottomed flask by rotary evaporation under

reduced pressure. DCM (50 mL) was and a Teflon-coated magnetic stir bar were added, followed by pyrrolidine (3.31 mL, 2.84 g, 40.0 mmol, 4.00 equiv.). The pressure tube was sealed and the mixture stirred for 1 h at 23 °C. Subsequently, the mixture was transferred to a 250 mL separatory funnel and water (50 mL) was added, the aqueous phase decanted and further extracted with DCM (3 x 50 mL). The combined organic layers were dried over $MgSO_4$, filtered, and the solvent evaporated by rotary evaporation under reduced pressure. To obtain analytically pure samples of the sulfonamides, the residue was purified by chromatography on silica gel.

General procedure for deaminative fluorination

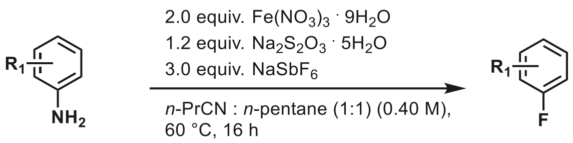

Caution: When performing reactions in pressurized vessels (such as closed vials, pressure tubes, and autoclaves), a blast shield must be used to minimize personal damage in case of an accident. See safety statement on page S8.

0.5 mmol Scale: Under an ambient atmosphere, to a 4-mL borosilicate vial equipped with a teflon-coated magnetic stir bar were added the (hetero) aromatic amine (if solid, 0.500 mmol, 1.00 equiv.), $Na_2S_2O_3$ $5H_2O$ (149 mg, 0.600 mmol, 1.20 equiv.), $NaSbF_6$ (388 mg, 1.50 mmol, 3.00 equiv.) and iron (III) nitrate nonahydrate (404 mg, 1.00 mmol, 2.00 equiv.). Then, butyronitrile (*n*-PrCN) (0.625 mL), *n*-pentane (0.625 mL) (0.4 M overall conc.) and (hetero) aromatic amine (if liquid, 0.500 mmol, 1.00 equiv.) were added, the vial was quickly sealed with a septum cap and the reaction mixture stirred at 60 °C for 16 h. After cooling to 23 °C, the resulting mixture was passed through a pad of celite (~2.5 g) by eluting with ethyl acetate (50 ml). Then, the solvent was evaporated by rotary evaporation under reduced pressure. In order to obtain analytically pure samples of fluoroarenes, the residue was purified by chromatography on silica gel.

General procedure for deaminative sulfonic acid synthesis

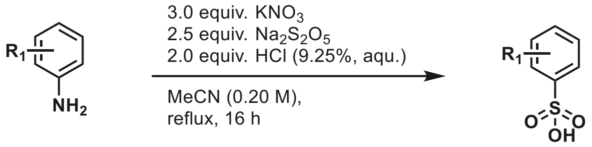

Caution: When performing reactions in pressurized vessels (such as closed vials, pressure tubes, and autoclaves), a blast shield must be used to minimize personal damage in case of an accident. See safety statement on page S8.

0.2 mmol Scale: Under an ambient atmosphere, to a 4-mL borosilicate vial equipped with a Teflon-coated magnetic stir bar were added the (hetero)aromatic amine (if solid, 0.200 mmol, 1.00 equiv.), KNO_3 (60.7 mg, 0.600 mmol, 2.00 equiv.), and $Na_2S_2O_5$ (95.1 mg, 0.500 mmol, 2.50 equiv.). Then, acetonitrile (1.0 mL), (hetero)aromatic amine (if liquid, 0.200 mmol, 1.00 equiv.), and HCl (9.25%, aqueous, 131 µL, 4.00 mmol, 2.00 equiv.) were added, the vial was quickly sealed with a septum cap and the mixture stirred at 85 °C (oil bath temperature) for 18 h. After cooling to 23 °C, the resulting mixture was concentrated by rotary evaporation under reduced pressure. To obtain analytically pure samples of the sulfonic acids, the residue was purified by chromatography on silica gel or on C18 columns.

Optimization Tables

Optimization of the deaminative sulfochlorination

Caution: When performing reactions in pressurized vessels (such as closed vials, pressure tubes, and autoclaves), a blast shield must be used to minimize personal damage in case of an accident. See safety statement on page S8.

General optimization procedure – deaminative sulfochlorination: Under an ambient atmosphere, to a 4-mL borosilicate vial equipped with a Teflon-coated magnetic stir bar were added 4-aminobenzonitrile (11.8 mg, 0.100 mmol, 1.00 equiv.), $Na_2S_2O_3$ $5H_2O$ (24.8 mg, 0.100 mmol, 1.00 equiv.) and CuCl (9.9 mg, 0.10 mmol, 1.0 equiv.). Then, acetonitrile (MeCN, 0.5 mL), HCl (4.0 M in 1,4-dioxane, 50 µL, 0.20 mmol, 2.0 equiv.), and SO_2 (3.0 M in MeCN, 0.33 mL, 1.0 mmol, 10 equiv.) were added, the vial was quickly sealed with a septum cap and the mixture stirred for 5 min at 23 °C. Subsequently, iron(III) nitrate nonahydrate (40.0 mg, 0.100 mmol, 1.00 equiv.) was added to the suspension and the vial was quickly resealed with a septum cap. The mixture was then stirred at 40 °C for 18 h. After cooling to 23 °C, the resulting mixture was concentrated by rotary evaporation under reduced pressure. The residue was diluted with water (1 mL) and extracted with DCM (3 × 1 mL). The organic layers were combined, the solvent was removed under reduced pressure, and the residue dissolved in $CDCl_3$ (0.6 mL). Dibromomethane was added as internal standard (7.0 µL, 17 mg, 0.10 mmol, 1.0 equiv.). The mixture was filtered and a ^{1}H-NMR spectrum recorded. The integration of the CH_2Br_2 signal (s, 5.0 ppm) was compared to the integration of a signal corresponding to aromatic protons of **1** (d, 8.2 ppm) to determine the yield.

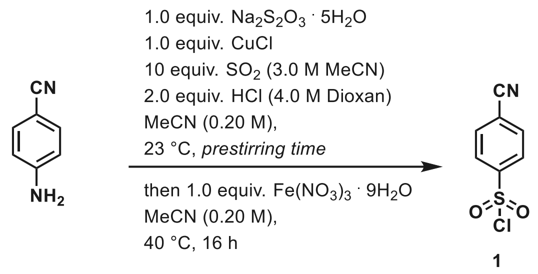

Table S1. Reaction outcome with different prestirring times before iron nitrate addition.

Entry	Prestirring time	Yield **1*** (%)
1	2 min	65
**2**	**5 min**	**75**
3	10 min	53
4	30 min	3

*.The yield was determined by ^{1}H NMR spectroscopy at 500 MHz and 298 K by dissolving the residue of the reaction mixture in 0.6 mL of $CDCl_3$, and using CH_2Br_2 as internal standard (7.0 µL, 17 mg, 0.10 mmol, 1.0 equiv.). The integration of the CH_2Br_2 signal at 4.96 ppm (s, 2H) was compared to the signal of **1** at 8.20 ppm (d, J = 8.7 Hz, 2H).

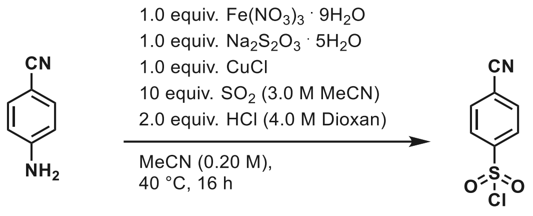

1

Table S2. Reaction outcome with modifications of the standard protocol.

Entry	Change from standard protocol	Yield **1*** (%)
1	2 min prestirring before iron nitrate addition with **open vial**	10
**2**	2 min prestirring before iron nitrate addition with **open vial**, then vial filled with Ar	8
3	First all solids weighted into the reaction vial (including iron nitrate), then all liquids added	6
4	All solids weighted into reaction vial apart from $Na_2S_2O_3$. $5H_2O$, the all liquids, then 2 min prestirring, then addition of $Na_2S_2O_3$. $5H_2O$	4
5	No prestirring before iron nitrate addition	44

*. The yield was determined by ^{1}H NMR spectroscopy at 500 MHz and 298 K by dissolving the residue of the reaction mixture in 0.6 mL of $CDCl_3$, and using CH_2Br_2 as internal standard (7.0 µL, 17 mg, 0.10 mmol, 1.0 equiv.). The integration of the CH_2Br_2 signal at 4.96 ppm (s, 2H) was compared to the signal of **1** at 8.20 ppm (d, *J* = 8.7 Hz, 2H).

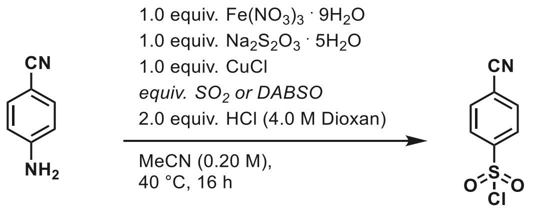

1

Table S3. Reaction outcome with different equivalents of SO_2 or DABSO.

Entry	SO_2 or DABSO	Yield **1*** (%)
1	5 equiv. SO_2	54
**2**	**10 equiv. SO_2**	**81**
3	15 equiv. SO_2	74
4	0.5 equiv. DABSO	< 5%
5	1 equiv. DABSO	< 5%
6	2 equiv. DABSO	< 5%
7	5 equiv. DABSO	< 5%
8	10 equiv. DABSO	< 5%

*. The yield was determined by ^{1}H NMR spectroscopy at 500 MHz and 298 K by dissolving the residue of the reaction mixture in 0.6 mL of $CDCl_3$, and using CH_2Br_2 as internal standard (7.0 µL, 17 mg, 0.10 mmol, 1.0 equiv.). The integration of the CH_2Br_2 signal at 4.96 ppm (s, 2H) was compared to the signal of **1** at 8.20 ppm (d, *J* = 8.7 Hz, 2H).

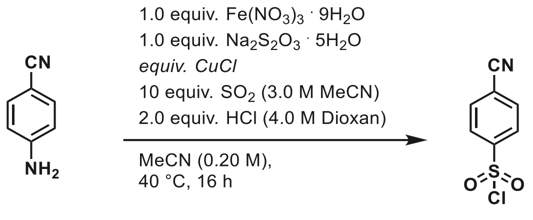

Table S4. Reaction outcome with different equivalents of CuCl.

Entry	CuCl equivalents	Yield **1*** (%)
1	0 equiv.	32%
2	0.05 equiv.	66%
3	0.2 equiv.	79%
4	0.3 equiv.	81%
5	0.5 equiv.	81%
6	1 equiv.	81%

*. The yield was determined by ^{1}H NMR spectroscopy at 500 MHz and 298 K by dissolving the residue of the reaction mixture in 0.6 mL of $CDCl_3$, and using CH_2Br_2 as internal standard (7.0 µL, 17 mg, 0.10 mmol, 1.0 equiv.). The integration of the CH_2Br_2 signal at 4.96 ppm (s, 2H) was compared to the signal of **1** at 8.20 ppm (d, *J* = 8.7 Hz, 2H).

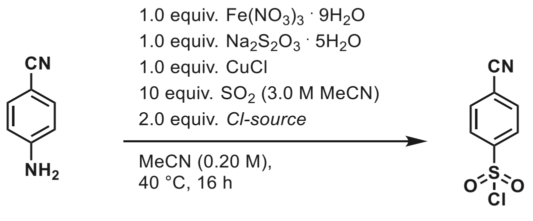

Table S5. Reaction outcome with different chloride sources

Entry	Cl-source	Yield **1*** (%)
1	TBACl	< 5%
2	TMACl	< 5%
3	LiCl	< 5%
4	$MgCl_2$	< 5%
**5**	**TMSCl**	**76%**

*. The yield was determined by ^{1}H NMR spectroscopy at 500 MHz and 298 K by dissolving the residue of the reaction mixture in 0.6 mL of $CDCl_3$, and using CH_2Br_2 as internal standard (7.0 µL, 17 mg, 0.10 mmol, 1.0 equiv.). The integration of the CH_2Br_2 signal at 4.96 ppm (s, 2H) was compared to the signal of **1** at 8.20 ppm (d, *J* = 8.7 Hz, 2H).

Optimization of the deaminative fluorination

Caution: When performing reactions in pressurized vessels (such as closed vials, pressure tubes, and autoclaves), a blast shield must be used to minimize personal damage in case of an accident. See safety statement on page S8.

General optimization procedure – deaminative fluorination: Under an ambient atmosphere, to a 4-mL borosilicate vial equipped with a magnetic stir bar were added benzocaine (16.5 mg, 0.100 mmol, 1.00 equiv.), $Na_2S_2O_3$ $5H_2O$ (29.8 mg, 0.120 mmol, 1.20 equiv.), iron (III) nitrate nonahydrate (80.8 mg, 0.200 mmol, 2.00 equiv.), fluoride source (0.120 mmol, 1.20 equiv.), and the solvent (0.25 mL, c = 0.40 M). The vessel was sealed with a septum cap and heated at 85 ºC for 16 h in an aluminum block on a heating plate while the reaction was stirred. After cooling to 23 °C, the resulting mixture was passed through pad of celite by eluting with ethyl acetate (10 ml). The yield was determined by ^{1}H-NMR spectroscopy by dissolving the crude mixture in 0.5 mL of $CDCl_3$, and using CH_2Br_2 as internal standard (7.0 µL, 17 mg, 0.10 mmol, 1.0 equiv.). The integration of the CH_2Br_2 signal at 4.96 ppm (s, 2H) was compared to the signal of **35** at 7.14 – 7.05 (m, 2H).

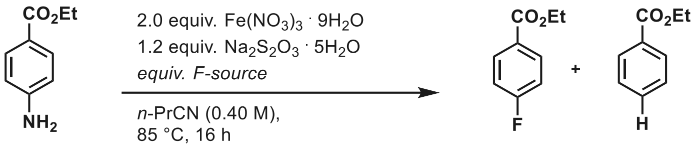

35a 35 35b

Table S6. Reaction outcome with different fluoride sources

Entry	F-source	Yield **35*** (%)	Yield **35b*** (%)
1	2 equiv. $LiBF_4$	16%	< 5%
2	2 equiv. $NaBF_4$	8%	< 5%
3	2 equiv. $TBABF_4$	6%	17%
4	2 equiv. NH_4BF_4	12%	< 5%
5	2 equiv. $LiPF_6$	20%	< 5%
6	2 equiv. KPF_6	17%	< 5%
7	2 equiv. $TBAPF_6$	13%	10%
8	2 equiv. NH_4PF_6	21%	< 5%
9	2 equiv. $NaSbF_6$	37%	< 5%
**10**	**3 equiv. $NaSbF_6$**	**42 %**	**< 5%**
11	2 equiv. $TBASbF_6$	5%	< 5%

*.The yield was determined by ^{1}H NMR spectroscopy at 500 MHz and 298 K by dissolving the residue of the reaction mixture in 0.6 mL of $CDCl_3$, and using CH_2Br_2 as internal standard (7.0 µL, 17 mg, 0.10 mmol, 1.0 equiv.). The integration of the CH_2Br_2 signal at 4.96 ppm (s, 2H) was compared to the signal of **35** at 7.14 – 7.05 (m, 2H) and signal of **35b** at 7.51 (m, 1H).

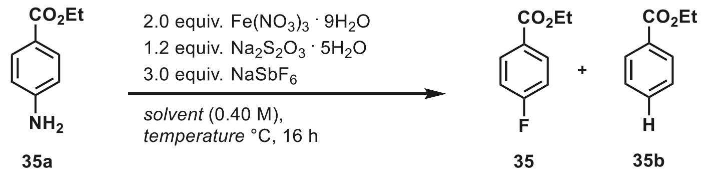

Table S7. Reaction outcome with different solvent

Entry	Solvent	Temperature (ºC)	Yield **35*** (%)	Yield **35b*** (%)
1	*n*-PrCN	85 ºC	42%	< 5%
2	MeCN	85 ºC	27%	< 5%
**3**	***n*-PrCN : pentane (1:1)**	**60 ºC**	**59%**	**< 5%**
4	*n*-PrCN : toluene (1:1)	60 ºC	42%	< 5%
5	*n*-PrCN : benzene (1:1)	60 ºC	44%	< 5%
6	*n*-PrCN : heptane (1:1)	60 ºC	52%	< 5%
7	THF	85 ºC	-	51%
8	NMP	85 ºC	-	55%

*. The yield was determined by ^{1}H NMR spectroscopy at 500 MHz and 298 K by dissolving the residue of the reaction mixture in 0.6 mL of $CDCl_3$, and using CH_2Br_2 as internal standard (7.0 µL, 17 mg, 0.10 mmol, 1.0 equiv.). The integration of the CH_2Br_2 signal at 4.96 ppm (s, 2H) was compared to the signal of **35** at 7.14 – 7.05 (m, 2H) and signal of **35b** at 7.51 (m, 1H).

Optimization of the deaminative sulfonic acid synthesis

Caution: When performing reactions in pressurized vessels (such as closed vials, pressure tubes, and autoclaves), a blast shield must be used to minimize personal damage in case of an accident. See safety statement on page S8.

General optimization procedure – deaminative sulfonic acid synthesis: Under an ambient atmosphere, to a 4-mL borosilicate vial equipped with a Teflon-coated magnetic stir bar were added the 4-aminobenzonitrile (11.8 mg, 0.100 mmol, 1.00 equiv.), KNO_3 (30.0 mg, 0.300 mmol, 3.00 equiv.), and $Na_2S_2O_5$ (48.0 mg, 0.250 mmol, 2.50 equiv.). Then, acetonitrile (0.5 mL) and HCl (9.25%, aqueous, 66.0 µL, 2.00 mmol, 2.00 equiv.) were added, the vial was quickly sealed with a septum cap and the mixture stirred at 85 °C (oil bath temperature) for 18 h. After cooling to 23 °C, the resulting mixture was concentrated by rotary evaporation under reduced pressure. The residue was dissolved in DMSO-d_6 (0.6 mL) and dibromomethane was added as internal standard (7.0 µL, 17 mg, 0.10 mmol, 1.0 equiv.). The mixture was filtered and a ^{1}H-NMR spectrum recorded. The integration of the CH_2Br_2 signal (s, 5.0 ppm) was compared to the integration of a signal corresponding to aromatic protons of sulfonic acid **44** (d, 7.78 ppm) to determine the yield.

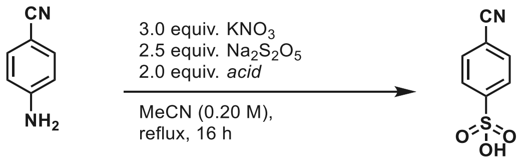

44

Table S8. Reaction outcome with different acids in the presence of $Na_2S_2O_5$.

Entry	Acid	Yield **44*** (%)
1	none	<5
**2**	**HCl (aq. 37%)**	**70**
3	HNO_3 (aq. 67%)	71
4	H_2SO_4 (95–97%)	56
5	$HBF_4{\cdot}Et_2O$	16
6	HPF_6 (aq. 55%)	25

*. The yield was determined by ^{1}H NMR spectroscopy at 500 MHz and 298 K by dissolving the residue of the reaction mixture in 0.6 mL of DMSO-d_6, and using CH_2Br_2 as internal standard (7.0 µL, 17 mg, 0.10 mmol, 1.0 equiv.). The integration of the CH_2Br_2 signal at 4.96 ppm (s, 2H) was compared to the signal of **44** at 7.78 ppm (d, J = 8.3 Hz, 2H).

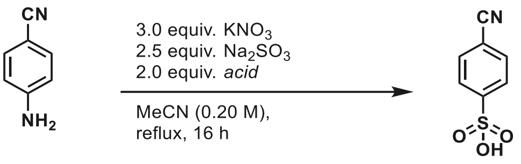

44

Table S9. Reaction outcome with different acids in the presence of Na_2SO_3 (instead of $Na_2S_2O_5$).

Entry	Acid	Yield **44*** (%)
1	none	<5
**2**	HCl (aq. 37%)	21
3	HNO_3 (aq. 67%)	27
4	H_2SO_4 (95–97%)	40
5	$HBF_4 \cdot Et_2O$	23
6	HPF_6 (aq. 55%)	<5%

*. The yield was determined by ^1H NMR spectroscopy at 500 MHz and 298 K by dissolving the residue of the reaction mixture in 0.6 mL of DMSO-d_6, and using CH_2Br_2 as internal standard (7.0 µL, 17 mg, 0.10 mmol, 1.0 equiv.). The integration of the CH_2Br_2 signal at 4.96 ppm (s, 2H) was compared to the signal of **44** at 7.78 ppm (d, J = 8.3 Hz, 2H).

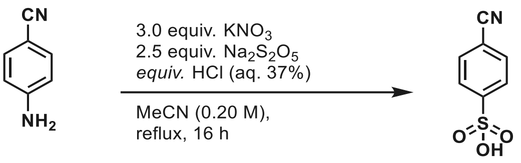

44

Table S10. Reaction outcome with different equivalents of HCl in the presence of $Na_2S_2O_5$.

Entry	Equivalents HCl	Yield **44*** (%)
1	No acid	<5
2	1.0 equiv.	28
**3**	**2.0 equiv**	**68**
4	5.0 equiv.	60

*. The yield was determined by ^1H NMR spectroscopy at 500 MHz and 298 K by dissolving the residue of the reaction mixture in 0.6 mL of DMSO-d_6, and using CH_2Br_2 as internal standard (7.0 µL, 17 mg, 0.10 mmol, 1.0 equiv.). The integration of the CH_2Br_2 signal at 4.96 ppm (s, 2H) was compared to the signal of **44** at 7.78 ppm (d, J = 8.3 Hz, 2H).

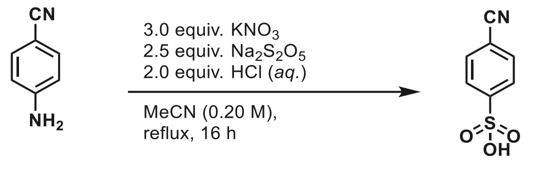

Table S11. Reaction outcome with different HCl concentrations in the presence of $Na_2S_2O_5$.

Entry	HCl concentration	Yield **44*** (%)
1	37%	69
2	18.5 %	77
**3**	**9.25%**	**95**

*. The yield was determined by ^{1}H NMR spectroscopy at 500 MHz and 298 K by dissolving the residue of the reaction mixture in 0.6 mL of DMSO-d_6, and using CH_2Br_2 as internal standard (7.0 µL, 17 mg, 0.10 mmol, 1.0 equiv.). The integration of the CH_2Br_2 signal at 4.96 ppm (s, 2H) was compared to the signal of **44** at 7.78 ppm (d, J = 8.3 Hz, 2H).

Deaminative Sulfonylations with Subsequent Derivatization

Deaminative sulfochlorination for sulfonyl chloride 4

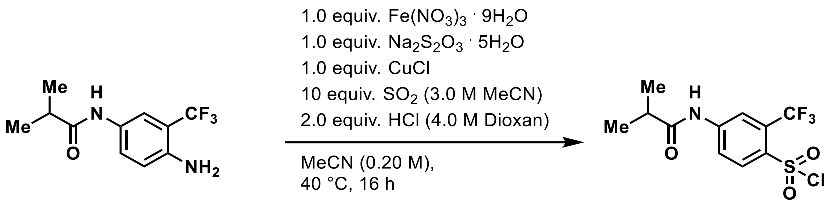

3 **4**, 82%

Caution: A blast shield must be used to minimize personal damage in case of an accident. See safety statement on page S8.

Under an ambient atmosphere, to a 20-mL borosilicate vial equipped with a Teflon-coated magnetic stir bar were added reduced Flutamide **3** (123 mg, 0.500 mmol, 1.00 equiv.), $Na_2S_2O_3$ $5H_2O$ (124 mg, 0.500 mmol, 1.00 equiv.) and CuCl (49.5 mg, 0.500 mmol, 1.0 equiv.). Then, acetonitrile (2.5 mL), HCl (4.0 M in 1,4-dioxane, 250 µL, 1.00 mmol, 2.0 equiv.), and SO_2 (3.0 M in MeCN, 1.7 mL, 5.0 mmol, 10 equiv.) were added, the vial was quickly sealed with a septum cap and the mixture stirred for 5 min at 23 °C. Subsequently, iron(III) nitrate nonahydrate (202 mg, 0.500 mmol, 1.00 equiv.) was added to the suspension and the vial was quickly resealed with a septum cap. The mixture was then stirred at 40 °C for 18 h. After cooling to 23 °C, the resulting mixture was concentrated by rotary evaporation under reduced pressure. The residue was diluted with water (5 mL) and extracted with EtOAc (3 × 5 mL). The organic layers were combined in a 20-mL borosilicate vial and concentrated by rotary evaporation under reduced pressure. The residue was purified by column chromatography on silica with ethyl acetate in pentane (gradient, 0%–50% over 50 column volumes) as eluent, to give the desired sulfonyl chloride **4** as a yellow solid (0.13 g, 0.41 mmol, 82%).

$\mathbf{R}_{f}$ = 0.61 (EtOAc in cyclohexane = 50%, v/v (UV)).

NMR Spectroscopy:

^{1}H NMR (500 MHz, $CDCl_3$, 23°C, δ): 8.28 (d, *J* = 8.6 Hz, 1H), 8.21–8.00 (m, 2H), 7.72 (s, 1H), 2.60 (p, *J* = 6.9 Hz, 1H), 1.29 (d, *J* = 6.9 Hz, 6H).

^{13}C NMR (125 MHz, $CDCl_3$, 23°C, δ): 176.0, 144.5, 136.3, 133.6, 129.6 (q, *J* = 34.5 Hz), 121.9 (q, *J* = 274.8 Hz), 121.5, 118.9 (q, *J* = 6.6 Hz), 37.1, 19.5.

^{19}F NMR (565 MHz, $CDCl_3$, 23°C, δ): −57.74.

HRMS-ESI (m/z) calc'd for $C_{11}H_{10}N_1O_3S_1F_3Cl_1$ $[M-H]^-$, 328.0028; found, 328.0029; deviation: −0.6 ppm.

Deaminative sulfoamidation for sulfonamide 5

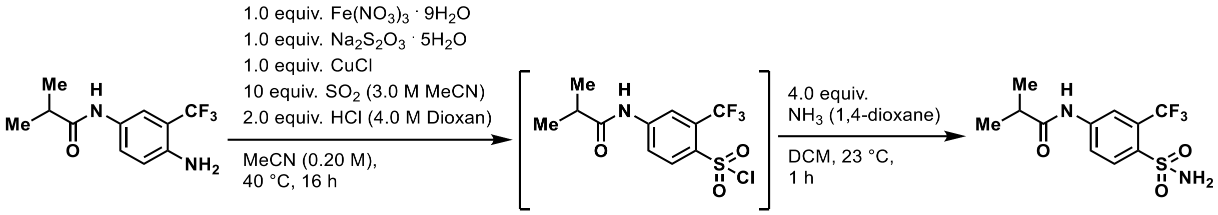

3 **4** **5**, 46%

Caution: A blast shield must be used to minimize personal damage in case of an accident. See safety statement on page S8.

Under an ambient atmosphere, to a 20-mL borosilicate vial equipped with a Teflon-coated magnetic stir bar were added reduced Flutamide **3** (123 mg, 0.500 mmol, 1.00 equiv.), $Na_2S_2O_3$ $5H_2O$ (124 mg, 0.500 mmol, 1.00 equiv.) and CuCl (49.5 mg, 0.500 mmol, 1.0 equiv.). Then, acetonitrile (2.5 mL), HCl (4.0 M in 1,4-dioxane, 250 µL, 1.00 mmol, 2.0 equiv.), and SO_2 (3.0 M in MeCN, 1.7 mL, 5.0 mmol, 10 equiv.) were added, the vial was quickly sealed with a septum cap and the mixture stirred for 5 min at 23 °C. Subsequently, iron(III) nitrate nonahydrate (202 mg, 0.500 mmol, 1.00 equiv.) was added to the suspension and the vial was quickly resealed with a septum cap. The mixture was then stirred at 40 °C for 18 h. After cooling to 23 °C, the resulting mixture was concentrated by rotary evaporation under reduced pressure. The residue was diluted with water (5 mL) and extracted with EtOAc (3 × 5 mL). The organic layers were combined in a 20-mL borosilicate vial and concentrated by rotary evaporation under reduced pressure. Then, DCM (5 mL) was added, followed by a Teflon-coated magnetic stir bar and NH_3 (0.5 M in 1,4-dioxane, 4.00 mL, 171 mg, 2.00 mmol, 4.00 equiv.). The vessel was sealed with a septum cap and the mixture stirred for 1 h at 23 °C. Subsequently, HCl solution (10% aqueous, 5 mL) was added, and the mixture extracted with EtOAc (3 × 5 mL). The combined organic layers were dried over $MgSO_4$, filtered, and the solvent evaporated under reduced pressure. The residue was purified by column chromatography on silica with ethyl acetate in pentane (gradient, 0%–100% over 50 column volumes) as eluent, to give the desired primary sulfonamide **5** as a white solid (70 mg, 0.23 mmol, 46%).

R_f = 0.45 (MeOH in DCM = 5%, v/v (UV)).

NMR Spectroscopy:

^{1}H NMR (500 MHz, MeCN-d_3, 23°C, δ): 8.71 (s, 1H), 8.19 (d, J = 2.2 Hz, 1H), 8.12 (dd, J = 8.8, 0.7 Hz, 1H), 7.97 (ddd, J = 8.8, 2.3, 0.5 Hz, 1H), 5.75 (s, 2H), 2.59 (p, J = 6.9 Hz, 1H), 1.18 (d, J = 6.9 Hz, 6H).

^{13}C NMR (125 MHz, MeCN-d_3, 23°C, δ): 177.5, 144.1, 135.9, 132.6, 128.2 (q, J = 32.8 Hz), 124.1 (q, J = 273.1 Hz), 122.4, 119.1 (q, J = 6.7 Hz), 36.9, 19.6.

^{19}F NMR (565 MHz, MeCN-d_3, 23°C, δ): −58.64.

HRMS-EI (m/z) calc'd for $C_{11}H_{13}N_2O_3S_1F_3$ $[M]^+$, 310.0594; found, 310.0594; deviation: −0.3 ppm.

Deaminative sulfoamidation for sulfonamide 6

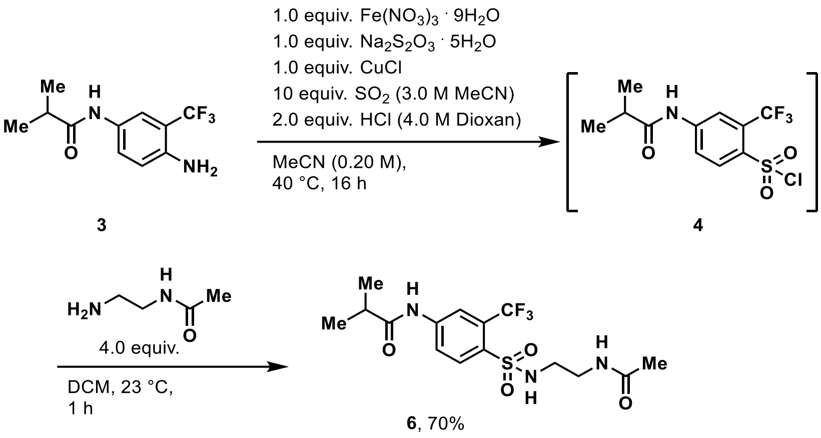

Caution: A blast shield must be used to minimize personal damage in case of an accident. See safety statement on page S8.

Under an ambient atmosphere, to a 20-mL borosilicate vial equipped with a Teflon-coated magnetic stir bar were added reduced Flutamide **3** (123 mg, 0.500 mmol, 1.00 equiv.), $Na_2S_2O_3$ $5H_2O$ (124 mg, 0.500 mmol, 1.00 equiv.) and CuCl (49.5 mg, 0.500 mmol, 1.0 equiv.). Then, acetonitrile (2.5 mL), HCl (4.0 M in 1,4-dioxane, 250 µL, 1.00 mmol, 2.0 equiv.), and SO_2 (3.0 M in MeCN, 1.7 mL, 5.0 mmol, 10 equiv.) were added, the vial was quickly sealed with a septum cap and the mixture stirred for 5 min at 23 °C. Subsequently, iron(III) nitrate nonahydrate (202 mg, 0.500 mmol, 1.00 equiv.) was added to the suspension and the vial was quickly resealed with a septum cap. The mixture was then stirred at 40 °C for 18 h. After cooling to 23 °C, the resulting mixture was concentrated by rotary evaporation under reduced pressure. The residue was diluted with water (5 mL) and extracted with EtOAc (3 × 5 mL). The organic layers were combined in a 20-mL borosilicate vial and concentrated by rotary evaporation under reduced pressure. Then, DCM (5 mL) was added, followed by a Teflon-coated magnetic stir bar and N-acetylethylenediamine (0.213 mL, 227 mg, 2.00 mmol, 4.00 equiv.). The vessel was sealed with a septum cap and the mixture stirred for 1 h at 23 °C. Subsequently, water (5 mL) was added, and the mixture extracted with EtOAc (3 × 5 mL). The combined organic layers were dried over $MgSO_4$, filtered, and the solvent evaporated under reduced pressure. The residue was purified by column chromatography on silica with ethyl acetate in pentane (gradient, 0%–100% over 30 column volumes, followed by 100% over 20 column volumes) as eluent, to give the desired secondary sulfonamide **6** as a white solid (139 mg, 0.35 mmol, 70%).

R_f = 0.53 (MeOH in DCM = 10%, v/v (UV)).

NMR Spectroscopy:

^{1}H NMR (500 MHz, MeCN-d_3, 23°C, δ): 8.75 (s, 1H), 8.21 (d, J = 2.2 Hz, 1H), 8.04 (dd, J = 8.8, 0.7 Hz, 1H), 8.01–7.93 (m, 1H), 6.50 (s, 1H), 5.96 (t, J = 5.8 Hz, 1H), 3.17 (q, J = 6.0 Hz, 2H), 2.98 (q, J = 5.8 Hz, 2H), 2.59 (hept, J = 6.9 Hz, 1H), 1.80 (s, 3H), 1.17 (d, J = 6.9 Hz, 6H).

^{13}C NMR (125 MHz, MeCN-d_3, 23°C, δ): 177.5, 171.8, 144.2, 133.7, 133.1, 128.7 (q, J = 32.7 Hz), 124.0 (q, J = 273.1 Hz), 122.2, 119.5 (q, J = 6.8 Hz), 44.2, 39.8, 36.9, 22.9, 19.6.

¹⁹F NMR (471 MHz, MeCN-d_3, 23°C, δ): −58.62.

HRMS-ESI (m/z) calc'd for $C_{15}H_{20}N_3O_4S_1F_3Na_1$ $[M+Na]^+$, 418.1019; found, 418.1015; deviation: +0.8 ppm.

Deaminative sulfoamidation for sulfonamide 7

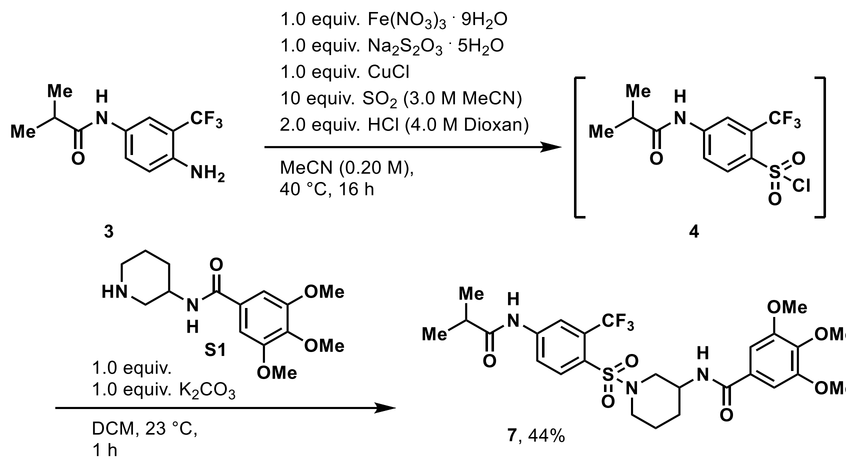

Caution: A blast shield must be used to minimize personal damage in case of an accident. See safety statement on page S8.

Under an ambient atmosphere, to a 20-mL borosilicate vial equipped with a Teflon-coated magnetic stir bar were added reduced Flutamide **3** (123 mg, 0.500 mmol, 1.00 equiv.), $Na_2S_2O_3$ $5H_2O$ (124 mg, 0.500 mmol, 1.00 equiv.) and CuCl (49.5 mg, 0.500 mmol, 1.0 equiv.). Then, acetonitrile (2.5 mL), HCl (4.0 M in 1,4-dioxane, 250 µL, 1.00 mmol, 2.0 equiv.), and SO_2 (3.0 M in MeCN, 1.7 mL, 5.0 mmol, 10 equiv.) were added, the vial was quickly sealed with a septum cap and the mixture stirred for 5 min at 23 °C. Subsequently, iron(III) nitrate nonahydrate (202 mg, 0.500 mmol, 1.00 equiv.) was added to the suspension and the vial was quickly resealed with a septum cap. The mixture was then stirred at 40 °C for 18 h. After cooling to 23 °C, the resulting mixture was concentrated by rotary evaporation under reduced pressure. The residue was diluted with water (5 mL) and extracted with EtOAc (3 × 5 mL). The organic layers were combined in a 20-mL borosilicate vial and concentrated by rotary evaporation under reduced pressure. Then, DCM (5 mL) was added, followed by a Teflon-coated magnetic stir bar, K_2CO_3 (69.1 mg, 0.50 mmol, 1.00 equiv), and Troxipid **S1** (147 mg, 0.50 mmol, 1.00 equiv.). The vessel was sealed with a septum cap and the mixture stirred for 1 h at 23 °C. Subsequently, water (5 mL) was added, and the mixture extracted with EtOAc (3 × 5 mL). The combined organic layers were dried over $MgSO_4$, filtered, and the solvent evaporated under reduced pressure. The residue was purified by column chromatography on silica with ethyl acetate in pentane (gradient, 0%–100% over 50 column volumes) as eluent, to give the desired tertiary sulfonamide **7** as a white solid (129 mg, 0.22 mmol, 44%).

R_f = 0.55 (MeOH in DCM = 10%, v/v (UV)).

NMR Spectroscopy:

¹H NMR (500 MHz, $CDCl_3$, 23°C, δ): 8.20–7.97 (m, 3H), 7.88 (s, 1H), 7.15–7.14 (m, 3H), 4.27 (s,

1H), 3.94 (s, 6H), 3.91 (s, 3H), 3.84 (d, *J* = 13.4 Hz, 1H), 3.62 (d, *J* = 13.6 Hz, 1H), 3.21 (dd, *J* = 13.6, 2.5 Hz, 1H), 3.00 (ddd, *J* = 13.5, 11.4, 2.7 Hz, 1H), 2.59 (p, *J* = 6.9 Hz, 1H), 2.14 (d, *J* = 13.3 Hz, 1H), 1.89 (q, *J* = 12.1 Hz, 1H), 1.76–1.73 (m, 1H), 1.28 (d, *J* = 6.8 Hz, 6H).

^{13}C NMR (125 MHz, $CDCl_3$, 23°C, δ): 176.2, 166.8, 153.4, 142.7, 140.9, 133.7, 131.7, 129.6, 129.0 (q, *J* = 33.3 Hz), 122.4 (q, *J* = 274.4 Hz), 121.6, 119.3 (d, *J* = 6.9 Hz), 104.5, 61.1, 56.5, 49.8, 46.6, 44.5, 36.9, 28.2, 21.6, 19.6 (d, *J* = 5.8 Hz).

^{19}F NMR (565 MHz, $CDCl_3$, 23°C, δ): −58.44.

HRMS-ESI (m/z) calc’d for $C_{26}H_{32}N_3O_7S_1F_3Na_1$ $[M+Na]^+$, 610.1805; found, 610.1808; deviation: −0.5 ppm.

Deaminative sulfofluorination for sulfonyl fluoride 8

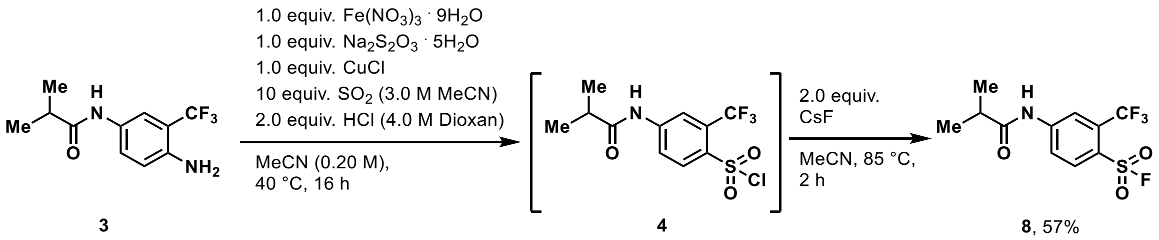

Caution: A blast shield must be used to minimize personal damage in case of an accident. See safety statement on page S8.

Under an ambient atmosphere, to a 20-mL borosilicate vial equipped with a Teflon-coated magnetic stir bar were added reduced Flutamide **3** (123 mg, 0.500 mmol, 1.00 equiv.), $Na_2S_2O_3$ $5H_2O$ (124 mg, 0.500 mmol, 1.00 equiv.) and CuCl (49.5 mg, 0.500 mmol, 1.0 equiv.). Then, acetonitrile (2.5 mL), HCl (4.0 M in 1,4-dioxane, 250 µL, 1.00 mmol, 2.0 equiv.), and SO_2 (3.0 M in MeCN, 1.7 mL, 5.0 mmol, 10 equiv.) were added, the vial was quickly sealed with a septum cap and the mixture stirred for 5 min at 23 °C. Subsequently, iron(III) nitrate nonahydrate (202 mg, 0.500 mmol, 1.00 equiv.) was added to the suspension and the vial was quickly resealed with a septum cap. The mixture was then stirred at 40 °C for 18 h. After cooling to 23 °C, the resulting mixture was concentrated by rotary evaporation under reduced pressure. The residue was diluted with water (5 mL) and extracted with EtOAc (3 × 5 mL). The organic layers were combined in a 20-mL borosilicate vial and concentrated by rotary evaporation under reduced pressure. Then, MeCN (5 mL) was added, followed by a Teflon-coated magnetic stir bar and CsF (152 mg, 1.00 mmol, 2.00 equiv.). The vessel was sealed with a septum cap and the mixture stirred for 2 h at 85 °C. Subsequently, sat. aq. $NaHCO_3$ solution (5 mL) was added, and the mixture extracted with EtOAc (3 × 5 mL). The combined organic layers were dried over $MgSO_4$, filtered, and the solvent evaporated under reduced pressure. The residue was purified by column chromatography on silica with ethyl acetate in pentane (gradient, 0%–30% over 50 column volumes) as eluent, to give the desired sulfonyl fluoride **8** as a faint yellow solid (89 mg, 0.28 mmol, 57%).

R*f* = 0.60 (EtOAc in cyclohexane = 50%, v/v (UV)).

NMR Spectroscopy:

^{1}H NMR (500 MHz, $CDCl_3$, 23°C, δ): 8.24 (d, *J* = 8.8 Hz, 1H), 8.13 (d, *J* = 2.2 Hz, 1H), 8.11–7.98 (m, 1H), 7.65 (s, 1H), 2.59 (hept, *J* = 6.9 Hz, 1H), 1.29 (d, *J* = 6.8 Hz, 6H).

^{13}C NMR (125 MHz, $CDCl_3$, 23°C, δ): 176.1, 144.8, 134.6 (d, *J* = 1.2 Hz), 131.0 (q, *J* = 34.6 Hz), 124.9 (d, *J* = 27.5 Hz), 121.7 (q, *J* = 274.5 Hz), 121.3, 119.2 (q, *J* = 6.2 Hz), 37.1, 19.5.

^{19}F NMR (565 MHz, $CDCl_3$, 23°C, δ): 66.46 (q, *J* = 12.2 Hz), −59.28 (d, *J* = 12.1 Hz).

HRMS-EI (m/z) calc'd for $C_{11}H_{11}N_1O_3S_1F_4$ $[M]^+$, 313.0390; found, 313.0390; deviation: +0.1 ppm.

Deaminative sulfonic acid synthesis for sulfonic acid 9

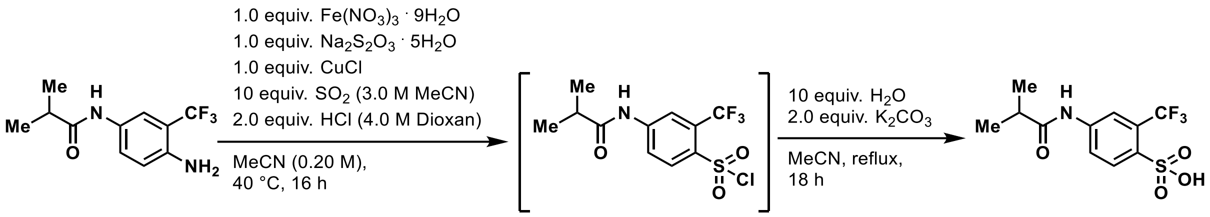

3 **4** **9**, 55%

Caution: A blast shield must be used to minimize personal damage in case of an accident. See safety statement on page S8.

Under an ambient atmosphere, to a 20-mL borosilicate vial equipped with a Teflon-coated magnetic stir bar were added reduced Flutamide **3** (123 mg, 0.500 mmol, 1.00 equiv.), $Na_2S_2O_3$ $5H_2O$ (124 mg, 0.500 mmol, 1.00 equiv.) and CuCl (49.5 mg, 0.500 mmol, 1.0 equiv.). Then, acetonitrile (2.5 mL), HCl (4.0 M in 1,4-dioxane, 250 µL, 1.00 mmol, 2.0 equiv.), and SO_2 (3.0 M in MeCN, 1.7 mL, 5.0 mmol, 10 equiv.) were added, the vial was quickly sealed with a septum cap and the mixture stirred for 5 min at 23 °C. Subsequently, iron(III) nitrate nonahydrate (202 mg, 0.500 mmol, 1.00 equiv.) was added to the suspension and the vial was quickly resealed with a septum cap. The mixture was then stirred at 40 °C for 18 h. After cooling to 23 °C, the resulting mixture was concentrated by rotary evaporation under reduced pressure. The residue was diluted with water (5 mL) and extracted with EtOAc (3 × 5 mL). The organic layers were combined in a 20-mL borosilicate vial and concentrated by rotary evaporation under reduced pressure. Then, MeCN (5 mL) was added, followed by a Teflon-coated magnetic stir bar, K_2CO_3 (138 mg, 1.00 mmol, 2.00 equiv.), and deionized H_2O (0.90 mL, 90.1 mg, 5.00 mmol, 10.0 equiv.). The vessel was sealed with a septum cap and the mixture stirred for 18 h at 85 °C. Subsequently, the solvent was evaporated and the residue purified by reversed phase column chromatography on C18 with MeOH in H_2O + 0.1% formic acid (gradient, 0%–100% over 50 column volumes) as eluent, to give the desired sulfonic acid **9** as a white solid (86 mg, 0.28 mmol, 55%).

R_f = 0.29 (MeOH in DCM = 20%, v/v (UV)).

NMR Spectroscopy:

^{1}H NMR (500 MHz, DMSO-d_6, 23°C, δ): 10.18 (s, 1H), 8.06 (d, J = 2.2 Hz, 1H), 7.98 (d, J = 8.6 Hz, 1H), 7.79 (dd, J = 8.7, 2.2 Hz, 1H), 2.69–2.55 (m, 1H), 1.08 (d, J = 6.8 Hz, 6H).

^{13}C NMR (125 MHz, DMSO-d_6, 23°C, δ): 176.0, 140.6, 140.2, 130.8, 126.2 (q, J = 31.7 Hz), 123.5 (q, J = 274.5 Hz), 121.1, 116.9 (q, J = 6.6 Hz), 35.1, 19.5.

^{19}F NMR (565 MHz, DMSO-d_6, 23°C, δ): −56.58.

HRMS-EI (m/z) calc'd for $C_{11}H_{11}N_1O_4S_1F_3$ $[M]^+$, 310.0366; found, 310.0369; deviation: −0.9 ppm.

Aniline Scope for Deaminative Sulfochlorination (isolation as sulfonamides)

4-(Pyrrolidin-1-ylsulfonyl)benzonitrile (10)

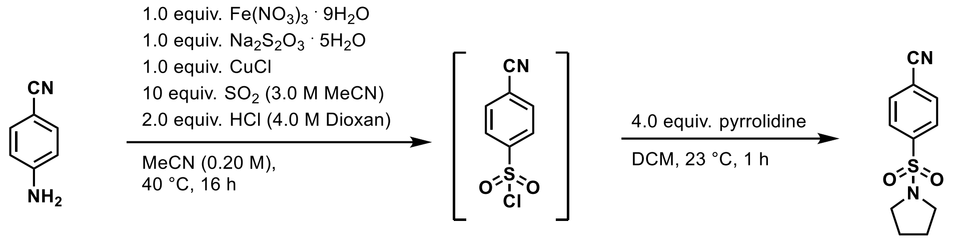

1a **1** **10**, 73%

Caution: A blast shield must be used to minimize personal damage in case of an accident. See safety statement on page S8.

Under an ambient atmosphere, to a 20-mL borosilicate vial equipped with a Teflon-coated magnetic stir bar were added 4-aminobenzonitrile (59.1 mg, 0.500 mmol, 1.00 equiv.), $Na_2S_2O_3$ $5H_2O$ (124 mg, 0.500 mmol, 1.00 equiv.) and CuCl (49.5 mg, 0.500 mmol, 1.0 equiv.). Then, acetonitrile (2.5 mL), HCl (4.0 M in 1,4-dioxane, 250 µL, 1.00 mmol, 2.0 equiv.), and SO_2 (3.0 M in MeCN, 1.7 mL, 5.0 mmol, 10 equiv.) were added, the vial was quickly sealed with a septum cap and the mixture stirred for 5 min at 23 °C. Subsequently, iron(III) nitrate nonahydrate (202 mg, 0.500 mmol, 1.00 equiv.) was added to the suspension and the vial was quickly resealed with a septum cap. The mixture was then stirred at 40 °C for 18 h. After cooling to 23 °C, the resulting mixture was concentrated by rotary evaporation under reduced pressure. The residue was diluted with water (5 mL) and extracted with DCM (3 × 5 mL). The organic layers were combined in a 20-mL borosilicate vial and a Teflon-coated magnetic stir bar was added, followed by pyrrolidine (165 µL, 142 mg, 2.00 mmol, 4.00 equiv.). The vessel was sealed with a septum cap and the mixture stirred for 1 h at 23 °C. Subsequently water (5 mL) was added, the aqueous phase decanted and further extracted with DCM (3 × 5 mL). The combined organic layers were dried over $MgSO_4$, filtered, and the solvent evaporated under reduced pressure. The residue was purified by column chromatography on silica with ethyl acetate in cyclohexane (gradient, 0%–50% over 30 column volumes) as eluent, to give the desired sulfonamide **10** as a colorless solid (86 mg, 0.36 mmol, 73%).

R*f* = 0.21 (EtOAc in cyclohexane = 20%, v/v (UV)).

NMR Spectroscopy:

> **^{1}H NMR** (500 MHz, $CDCl_3$, 23°C, δ): 8.04–7.89 (m, 2H), 7.87–7.77 (m, 2H), 3.45–3.00 (m, 4H), 1.92–1.73 (m, 4H).
>
> **^{13}C NMR** (125 MHz, $CDCl_3$, 23°C, δ): 141.8, 133.0, 128.1, 117.5, 116.5, 48.1, 25.5.

HRMS-EI (m/z) calc'd for $C_{11}H_{12}N_2O_2S_1$ $[M]^+$, 236.0614; found, 236.0614; deviation: +0.1 ppm.

1-((4-Phenoxyphenyl)sulfonyl)pyrrolidine (11)

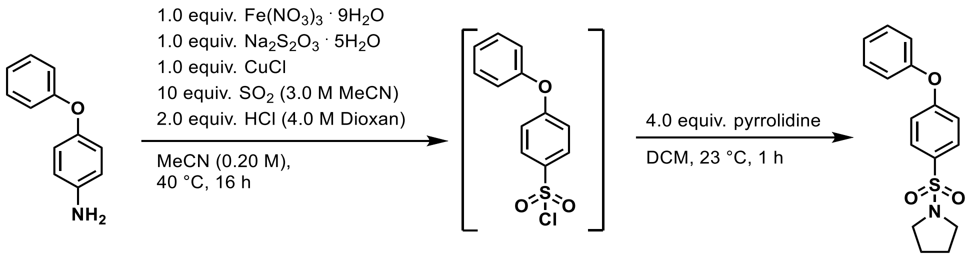

11a **11**, 63%

Caution: A blast shield must be used to minimize personal damage in case of an accident. See safety statement on page S8.

Under an ambient atmosphere, to a 20-mL borosilicate vial equipped with a Teflon-coated magnetic stir bar were added 4-phenoxyaniline (92.3 mg, 0.500 mmol, 1.00 equiv.), $Na_2S_2O_3$ $5H_2O$ (124 mg, 0.500 mmol, 1.00 equiv.) and CuCl (49.5 mg, 0.500 mmol, 1.0 equiv.). Then, acetonitrile (2.5 mL), HCl (4.0 M in 1,4-dioxane, 250 µL, 1.00 mmol, 2.0 equiv.), and SO_2 (3.0 M in MeCN, 1.7 mL, 5.0 mmol, 10 equiv.) were added, the vial was quickly sealed with a septum cap and the mixture stirred for 5 min at 23 °C. Subsequently, iron(III) nitrate nonahydrate (202 mg, 0.500 mmol, 1.00 equiv.) was added to the suspension and the vial was quickly resealed with a septum cap. The mixture was then stirred at 40 °C for 18 h. After cooling to 23 °C, the resulting mixture was concentrated by rotary evaporation under reduced pressure. The residue was diluted with water (5 mL) and extracted with DCM (3 × 5 mL). The organic layers were combined in a 20-mL borosilicate vial and a Teflon-coated magnetic stir bar was added, followed by pyrrolidine (165 µL, 142 mg, 2.00 mmol, 4.00 equiv.). The vessel was sealed with a septum cap and the mixture stirred for 1 h at 23 °C. Subsequently water (5 mL) was added, the aqueous phase decanted and further extracted with DCM (3 × 5 mL). The combined organic layers were dried over $MgSO_4$, filtered, and the solvent evaporated under reduced pressure. The residue was purified by column chromatography on silica with ethyl acetate in cyclohexane (gradient, 0%–50% over 30 column volumes) as eluent, to give the desired sulfonamide **11** as a colorless solid (95 mg, 0.31 mmol, 63%).

R*f* = 0.42 (EtOAc in cylohexane = 20%, v/v (UV)).

NMR Spectroscopy:

^{1}**H NMR** (500 MHz, $CDCl_3$, 23°C, δ): 8.05–7.64 (m, 2H), 7.41 (dd, *J* = 8.6, 7.4 Hz, 2H), 7.25–7.19 (m, 1H), 7.14–7.00 (m, 4H), 3.24 (t, *J* = 6.8 Hz, 4H), 2.31– .64 (m, 4H).

^{13}**C NMR** (125 MHz, $CDCl_3$, 23°C, δ): 161.6, 155.3, 130.9, 130.3, 129.8, 125.1, 120.4, 117.7, 48.1, 25.4.

HRMS-EI (m/z) calc'd for $C_{16}H_{17}N_1O_3S_1$ $[M]^+$, 303.0924; found, 303.0928; deviation: −1.3 ppm.

1-((4-Methoxyphenyl)sulfonyl)pyrrolidine (12)

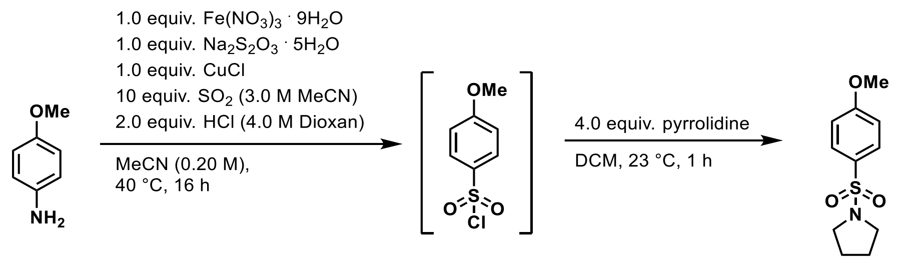

Caution: A blast shield must be used to minimize personal damage in case of an accident. See safety statement on page S8.

Under an ambient atmosphere, to a 4-mL borosilicate vial equipped with a Teflon-coated magnetic stir bar were added 4-methoxyaniline (12.3 mg, 0.100 mmol, 1.00 equiv.), $Na_2S_2O_3$ $5H_2O$ (24.8 mg, 0.100 mmol, 1.00 equiv.) and CuCl (9.90 mg, 0.100 mmol, 1.0 equiv.). Then, acetonitrile (0.5 mL), HCl (4.0 M in 1,4-dioxane, 50 µL, 0.10 mmol, 2.0 equiv.), and SO_2 (3.0M in MeCN, 0.323 mL, 1.00 mmol, 10.0 equiv.) were added, the vial was quickly sealed with a septum cap and the mixture stirred for 5 min at 23 °C. Subsequently, iron(III) nitrate nonahydrate (40.4 mg, 0.100 mmol, 1.00 equiv.) was added to the suspension and the vial was quickly resealed with a septum cap. The mixture was then stirred at 40 °C for 18 h. After cooling to 23 °C, the resulting mixture was concentrated by rotary evaporation under reduced pressure. The residue was diluted with water (2 mL) and extracted with DCM (3 × 2 mL). The organic layers were combined in a 4-mL borosilicate vial and a Teflon-coated magnetic stir bar was added, followed by pyrrolidine (33.1 µL, 28.4 mg, 0.400 mmol, 4.00 equiv.). The vessel was sealed with a septum cap and the mixture stirred for 1 h at 23 °C. Subsequently water (5 mL) was added, the aqueous phase decanted and further extracted with DCM (3 × 5 mL). The combined organic layers were dried over $MgSO_4$, filtered, and the solvent evaporated under reduced pressure. The residue was purified by column chromatography on silica with ethyl acetate in cyclohexane (gradient, 0%–50% over 30 column volumes) as eluent, to give the desired sulfonamide **12** as a colorless solid (11.2 mg, 0.46 mmol, 46%).

R_f = 0.22 (EtOAc in pentane = 20%, v/v (UV)).

NMR Spectroscopy:

^{1}H NMR (500 MHz, $CDCl_3$, 23°C, δ): 7.79 (d, J = 8.9 Hz, 2H), 7.01 (d, J = 8.9 Hz, 2H), 3.90 (s, 3H), 3.47–2.91 (m, 4H), 2.26–1.51 (m, 4H).

^{13}C NMR (125 MHz, $CDCl_3$, 23°C, δ): 130.0, 129.8, 128.8, 114.3, 55.7, 48.0, 25.3.

HRMS-EI (m/z) calc'd for $C_{11}H_{15}N_1O_3S_1$ $[M]^+$, 241.0767; found, 241.0765; deviation: −0.77 ppm.

2-Chloro-3-(pyrrolidin-1-ylsulfonyl)pyridine (13)

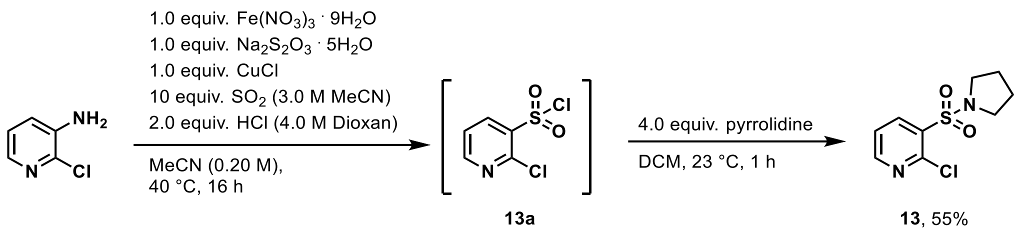

Caution: A blast shield must be used to minimize personal damage in case of an accident. See safety statement on page S8.

Under an ambient atmosphere, to a 20-mL borosilicate vial equipped with a Teflon-coated magnetic stir bar were added 3-amino-2-chloropyridine (64.3 mg, 0.500 mmol, 1.00 equiv.), $Na_2S_2O_3$ $5H_2O$ (124 mg, 0.500 mmol, 1.00 equiv.) and CuCl (49.5 mg, 0.500 mmol, 1.0 equiv.). Then, acetonitrile (2.5 mL), HCl (4.0 M in 1,4-dioxane, 250 µL, 1.00 mmol, 2.0 equiv.), and SO_2 (3.0 M in MeCN, 1.7 mL, 5.0 mmol, 10 equiv.) were added, the vial was quickly sealed with a septum cap and the mixture stirred for 5 min at 23 °C. Subsequently, iron(III) nitrate nonahydrate (202 mg, 0.500 mmol, 1.00 equiv.) was added to the suspension and the vial was quickly resealed with a septum cap. The mixture was then stirred at 40 °C for 18 h. After cooling to 23 °C, the resulting mixture was concentrated by rotary evaporation under reduced pressure. The residue was diluted with water (5 mL) and extracted with DCM (3 × 5 mL). The organic layers were combined in a 20-mL borosilicate vial and a Teflon-coated magnetic stir bar was added, followed by pyrrolidine (165 µL, 142 mg, 2.00 mmol, 4.00 equiv.). The vessel was sealed with a septum cap and the mixture stirred for 1 h at 23 °C. Subsequently water (5 mL) was added, the aqueous phase decanted and further extracted with DCM (3 × 5 mL). The combined organic layers were dried over $MgSO_4$, filtered, and the solvent evaporated under reduced pressure. The residue was purified by column chromatography on silica with ethyl acetate in pentane (15%, v/v) as eluent, to give the desired sulfonamide **13** as a colorless oil (68 mg, 0.28 mmol, 55%).

R*f* = 0.25 (EtOAc in pentane = 40%, v/v (UV)).

NMR Spectroscopy:

^{1}H NMR (500 MHz, $CDCl_3$, 23°C, δ): 8.55 (dd, *J* = 4.7, 1.9 Hz, 1H), 8.42 (dd, *J* = 7.8, 1.9 Hz, 1H), 7.41 (dd, *J* = 7.8, 4.7 Hz, 1H), 3.45 (t, *J* = 6.7 Hz, 4H), 2.41–1.63 (m, 4H).

^{13}C NMR (125 MHz, $CDCl_3$, 23°C, δ): 152.3, 148.5, 141.0, 134.7, 122.5, 48.1, 25.8.

HRMS-ESI (m/z) calc'd for $C_9H_{11}N_2O_2S_1Cl_1Na_1$ $[M+Na]^+$, 269.0122; found, 269.0122; deviation: +0.2 ppm.

1-((4'-Chloro-[1,1'-biphenyl]-2-yl)sulfonyl)pyrrolidine (14)

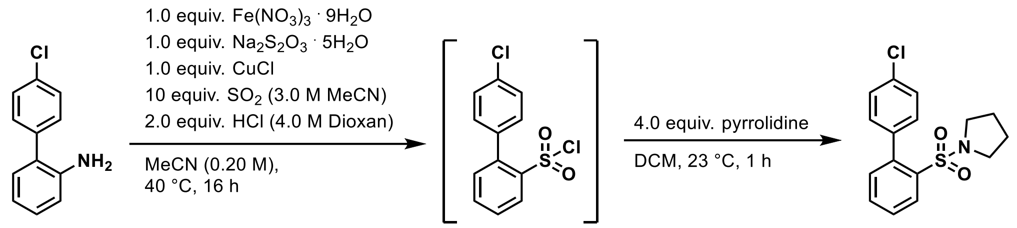

14a **14**, 83%

Caution: A blast shield must be used to minimize personal damage in case of an accident. See safety statement on page S8.

Under an ambient atmosphere, to a 20-mL borosilicate vial equipped with a Teflon-coated magnetic stir bar were added 4-chloro-(1-biphenyl)-2-amine (102 mg, 0.500 mmol, 1.00 equiv.), $Na_2S_2O_3$ $5H_2O$ (124 mg, 0.500 mmol, 1.00 equiv.) and CuCl (49.5 mg, 0.500 mmol, 1.0 equiv.). Then, acetonitrile (2.5 mL), HCl (4.0 M in 1,4-dioxane, 250 µL, 1.00 mmol, 2.0 equiv.), and SO_2 (3.0 M in MeCN, 1.7 mL, 5.0 mmol, 10 equiv.) were added, the vial was quickly sealed with a septum cap and the mixture stirred for 5 min at 23 °C. Subsequently, iron(III) nitrate nonahydrate (202 mg, 0.500 mmol, 1.00 equiv.) was added to the suspension and the vial was quickly resealed with a septum cap. The mixture was then stirred at 40 °C for 18 h. After cooling to 23 °C, the resulting mixture was concentrated by rotary evaporation under reduced pressure. The residue was diluted with water (5 mL) and extracted with DCM (3 × 5 mL). The organic layers were combined in a 20-mL borosilicate vial and a Teflon-coated magnetic stir bar was added, followed by pyrrolidine (165 µL, 142 mg, 2.00 mmol, 4.00 equiv.). The vessel was sealed with a septum cap and the mixture stirred for 1 h at 23 °C. Subsequently water (5 mL) was added, the aqueous phase decanted and further extracted with DCM (3 × 5 mL). The combined organic layers were dried over $MgSO_4$, filtered, and the solvent evaporated under reduced pressure. The residue was purified by column chromatography on silica with ethyl acetate in cyclohexane (gradient, 0%–50% over 30 column volumes) as eluent, to give the desired sulfonamide **14** as a colorless solid (0.13 mg, 0.42 mmol, 83%).

R_f = 0.49 (EtOAc in cyclohexane = 20%, v/v (UV)).

NMR Spectroscopy:

^{1}H NMR (500 MHz, $CDCl_3$, 23°C, δ): 8.14 (dd, J = 7.9, 1.4 Hz, 1H), 7.59 (td, J = 7.5, 1.4 Hz, 1H), 7.52 (td, J = 7.7, 1.4 Hz, 1H), 7.46–7.35 (m, 4H), 7.30 (dd, J = 7.7, 1.5 Hz, 1H), 3.30–2.42 (m, 4H), 2.22–1.54 (m, 4H).

^{13}C NMR (125 MHz, $CDCl_3$, 23°C, δ): 140.4, 138.4, 138.3, 134.0, 132.8, 132.4, 131.0, 130.1, 128.0, 127.8, 46.9, 25.8.

HRMS-EI (m/z) calc'd for $C_{16}H_{16}N_2O_2S_1Cl_1$ $[M]^+$, 321.0585; found, 321.0588; deviation: −1.2 ppm.

3-(Pyrrolidin-1-ylsulfonyl)-5-(trifluoromethyl)benzoic acid (15)

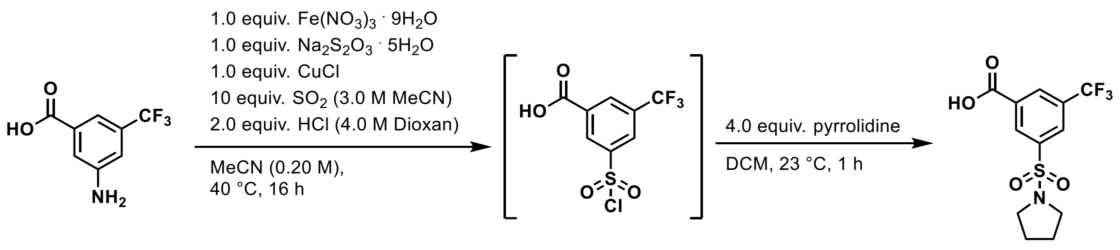

Caution: A blast shield must be used to minimize personal damage in case of an accident. See safety statement on page S8.

Under an ambient atmosphere, to a 20-mL borosilicate vial equipped with a Teflon-coated magnetic stir bar were added 3-amino-5-(trifluormethyl)-benzoic acid (103 mg, 0.500 mmol, 1.00 equiv.), $Na_2S_2O_3$ $5H_2O$ (124 mg, 0.500 mmol, 1.00 equiv.) and CuCl (49.5 mg, 0.500 mmol, 1.0 equiv.). Then, acetonitrile (2.5 mL), HCl (4.0 M in 1,4-dioxane, 250 µL, 1.00 mmol, 2.0 equiv.), and SO_2 (3.0 M in MeCN, 1.7 mL, 5.0 mmol, 10 equiv.) were added, the vial was quickly sealed with a septum cap and the mixture stirred for 5 min at 23 °C. Subsequently, iron(III) nitrate nonahydrate (202 mg, 0.500 mmol, 1.00 equiv.) was added to the suspension and the vial was quickly resealed with a septum cap. The mixture was then stirred at 40 °C for 18 h. After cooling to 23 °C, the resulting mixture was concentrated by rotary evaporation under reduced pressure. The residue was diluted with water (5 mL) and extracted with DCM (3 × 5 mL). The organic layers were combined in a 20-mL borosilicate vial and a Teflon-coated magnetic stir bar was added, followed by pyrrolidine (165 µL, 142 mg, 2.00 mmol, 4.00 equiv.). The vessel was sealed with a septum cap and the mixture stirred for 1 h at 23 °C. Subsequently water (5 mL) was added, the aqueous phase decanted and further extracted with DCM (3 × 5 mL). The combined organic layers were dried over $MgSO_4$, filtered, and the solvent evaporated under reduced pressure. The residue was purified by column chromatography on silica with ethyl acetate in pentane (38% + 2% AcOH) as eluent, to give the desired sulfonamide **15** as an off-white solid (0.11 mg, 0.35 mmol, 69%).

R_f = 0.74 (EtOAc in pentane = 28% + 2% AcOH, v/v (UV)).

NMR Spectroscopy:

^{1}H NMR (500 MHz, DMSO-d_6, 23°C, δ): 8.47 (s, 1H), 8.44 (s, 1H), 8.26 (s, 1H), 3.42–3.10 (m, 4H), 2.10–1.58 (m, 4H).

^{13}C NMR (125 MHz, DMSO-d_6, 23°C, δ): 165.3, 139.0, 134.5, 131.7, 131.1 (q, J = 33.2 Hz), 130.1 (q, J = 3.8 Hz), 127.7 (q, J = 3.7 Hz), 123.4 (q, J = 273.1 Hz), 48.4, 25.2.

^{19}F NMR (565 MHz, DMSO-d_6, 23°C, δ): −61.46.

HRMS-ESI (m/z) calc'd for $C_{12}H_{12}N_1O_4S_1F_3Na_1$ $[M+Na]^+$, 346.0331; found, 346.0332; deviation: −0.1 ppm.

4-(Pyrrolidin-1-ylsulfonyl)nitrobenzene (16)

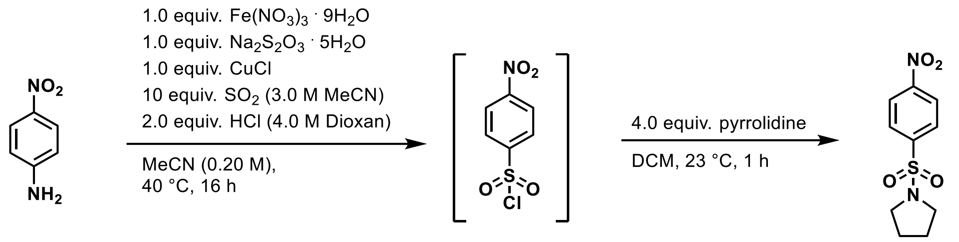

Caution: A blast shield must be used to minimize personal damage in case of an accident. See safety statement on page S8.

Under an ambient atmosphere, to a 20-mL borosilicate vial equipped with a Teflon-coated magnetic stir bar were added 4-nitroaniline (69.1 mg, 0.500 mmol, 1.00 equiv.), $Na_2S_2O_3$ $5H_2O$ (124 mg, 0.500 mmol, 1.00 equiv.) and CuCl (49.5 mg, 0.500 mmol, 1.0 equiv.). Then, acetonitrile (2.5 mL), HCl (4.0 M in 1,4-dioxane, 250 µL, 1.00 mmol, 2.0 equiv.), and SO_2 (3.0 M in MeCN, 1.7 mL, 5.0 mmol, 10 equiv.) were added, the vial was quickly sealed with a septum cap and the mixture stirred for 5 min at 23 °C. Subsequently, iron(III) nitrate nonahydrate (202 mg, 0.500 mmol, 1.00 equiv.) was added to the suspension and the vial was quickly resealed with a septum cap. The mixture was then stirred at 40 °C for 18 h. After cooling to 23 °C, the resulting mixture was concentrated by rotary evaporation under reduced pressure. The residue was diluted with water (5 mL) and extracted with DCM (3 × 5 mL). The organic layers were combined in a 20-mL borosilicate vial and a Teflon-coated magnetic stir bar was added, followed by pyrrolidine (165 µL, 142 mg, 2.00 mmol, 4.00 equiv.). The vessel was sealed with a septum cap and the mixture stirred for 1 h at 23 °C. Subsequently water (5 mL) was added, the aqueous phase decanted and further extracted with DCM (3 × 5 mL). The combined organic layers were dried over $MgSO_4$, filtered, and the solvent evaporated under reduced pressure. The residue was purified by column chromatography on silica with ethyl acetate in pentane (12%, v/v) as eluent, to give the desired sulfonamide **16** as a yellow solid (81 mg, 0.32 mmol, 63%).

R*f* = 0.37 (EtOAc in pentane = 12%, v/v (UV)).

NMR Spectroscopy:

^{1}H NMR (500 MHz, $CDCl_3$, 23°C, δ): 8.39 (d, *J* = 8.8 Hz, 2H), 8.03 (d, *J* = 8.8 Hz, 2H), 3.65–3.02 (m, 4H), 2.25–1.59 (m, 4H).

^{13}C NMR (125 MHz, $CDCl_3$, 23°C, δ): 150.1, 143.2, 128.5, 124.3, 48.1, 25.4.

HRMS-EI (m/z) calc'd for $C_{10}H_{12}N_2O_4S_1$ $[M]^+$, 256.0512; found, 256.0512; deviation: +0.3 ppm.

1-((2-Chloro-4-iodophenyl)sulfonyl)pyrrolidine (17)

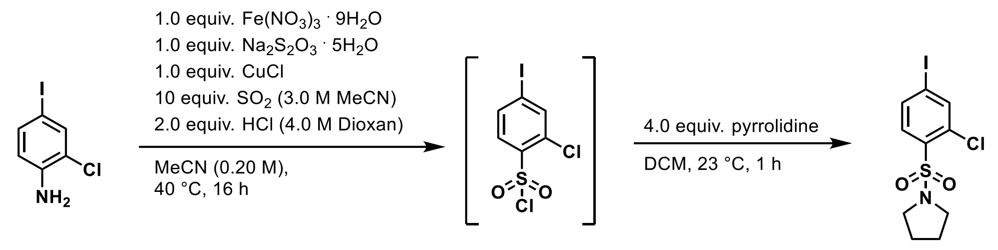

Caution: A blast shield must be used to minimize personal damage in case of an accident. See safety statement on page S8.

Under an ambient atmosphere, to a 20-mL borosilicate vial equipped with a Teflon-coated magnetic stir bar were added 2-chloro-4-iodaniline (127 mg, 0.500 mmol, 1.00 equiv.), $Na_2S_2O_3$ $5H_2O$ (124 mg, 0.500 mmol, 1.00 equiv.) and CuCl (49.5 mg, 0.500 mmol, 1.0 equiv.). Then, acetonitrile (2.5 mL), HCl (4.0 M in 1,4-dioxane, 250 µL, 1.00 mmol, 2.0 equiv.), and SO_2 (3.0 M in MeCN, 1.7 mL, 5.0 mmol, 10 equiv.) were added, the vial was quickly sealed with a septum cap and the mixture stirred for 5 min at 23 °C. Subsequently, iron(III) nitrate nonahydrate (202 mg, 0.500 mmol, 1.00 equiv.) was added to the suspension and the vial was quickly resealed with a septum cap. The mixture was then stirred at 40 °C for 18 h. After cooling to 23 °C, the resulting mixture was concentrated by rotary evaporation under reduced pressure. The residue was diluted with water (5 mL) and extracted with DCM (3 × 5 mL). The organic layers were combined in a 20-mL borosilicate vial and a Teflon-coated magnetic stir bar was added, followed by pyrrolidine (165 µL, 142 mg, 2.00 mmol, 4.00 equiv.). The vessel was sealed with a septum cap and the mixture stirred for 1 h at 23 °C. Subsequently water (5 mL) was added, the aqueous phase decanted and further extracted with DCM (3 × 5 mL). The combined organic layers were dried over $MgSO_4$, filtered, and the solvent evaporated under reduced pressure. The residue was purified by column chromatography on silica with ethyl acetate in cyclohexane (gradient, 0%–50% over 30 column volumes) as eluent, to give the desired sulfonamide **17** as a colorless solid (0.130 g, 0.35 mmol, 70%).

R_f = 0.52 (EtOAc in cyclohexane = 20%, v/v (UV)).

NMR Spectroscopy:

^{1}**H NMR** (500 MHz, $CDCl_3$, 23°C, δ): 7.89 (d, *J* = 1.6 Hz, 1H), 7.76 (d, *J* = 8.3 Hz, 1H), 7.73 (dd, *J* = 8.3, 1.6 Hz, 1H), 3.44–3.30 (m, 4H), 2.10–1.82 (m, 4H).

^{13}**C NMR** (125 MHz, $CDCl_3$, 23°C, δ): 140.4, 137.1, 136.3, 133.1, 133.0, 99.5, 47.9, 25.9.

HRMS-EI (m/z) calc'd for $C_{10}H_{11}N_1O_2S_1Cl_1I_1$ $[M]^+$, 370.9238; found, 370.9242; deviation: −1.0 ppm.

Phenyl(4-(pyrrolidin-1-ylsulfonyl)phenyl)methanone (18)

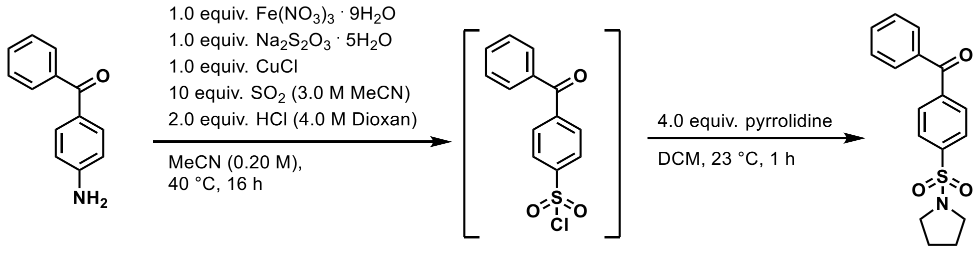

18a

18, 76%

Caution: A blast shield must be used to minimize personal damage in case of an accident. See safety statement on page S8.

0.5 mmol Scale: Under an ambient atmosphere, to a 20-mL borosilicate vial equipped with a Teflon-coated magnetic stir bar were added 4-aminobenzophenone (98.6 mg, 0.500 mmol, 1.00 equiv.), $Na_2S_2O_3$ $5H_2O$ (124 mg, 0.500 mmol, 1.00 equiv.) and CuCl (49.5 mg, 0.500 mmol, 1.0 equiv.). Then, acetonitrile (2.5 mL), HCl (4.0 M in 1,4-dioxane, 250 µL, 1.00 mmol, 2.0 equiv.), and SO_2 (3.0 M in MeCN, 1.7 mL, 5.0 mmol, 10 equiv.) were added, the vial was quickly sealed with a septum cap and the mixture stirred for 5 min at 23 °C. Subsequently, iron(III) nitrate nonahydrate (202 mg, 0.500 mmol, 1.00 equiv.) was added to the suspension and the vial was quickly resealed with a septum cap. The mixture was then stirred at 40 °C for 18 h. After cooling to 23 °C, the resulting mixture was concentrated by rotary evaporation under reduced pressure. The residue was diluted with water (5 mL) and extracted with DCM (3 × 5 mL). The organic layers were combined in a 20-mL borosilicate vial and a Teflon-coated magnetic stir bar was added, followed by pyrrolidine (165 µL, 142 mg, 2.00 mmol, 4.00 equiv.). The vessel was sealed with a septum cap and the mixture stirred for 1 h at 23 °C. Subsequently water (5 mL) was added, the aqueous phase decanted and further extracted with DCM (3 × 5 mL). The combined organic layers were dried over $MgSO_4$, filtered, and the solvent evaporated under reduced pressure. The residue was purified by column chromatography on silica with ethyl acetate in cyclohexane (gradient, 0%–50% over 30 column volumes) as eluent, to give the desired sulfonamide **18** as a colorless solid (0.12 g, 0.38 mmol, 76%).

10 mmol Scale: Under an ambient atmosphere, to a 350-mL Ace pressure tube equipped with a Teflon-coated magnetic stir bar were added the 4-aminobenzophenone (1.97 g, 10.0 mmol, 1.00 equiv.), $Na_2S_2O_3$ $5H_2O$ (2.48 g, 10.0 mmol, 1.00 equiv.) and CuCl (990 mg, 10.0 mmol, 1.00 equiv.). Then, acetonitrile (50 mL), HCl (4.0 M in 1,4-dioxane, 5.0 mL, 20 mmol, 2.0 equiv.), and SO_2 (3.00 M in MeCN, 32.3 mL, 100 mmol, 10.0 equiv.) were added, the vessel was quickly sealed and the mixture stirred for 5 min at 23 °C. Subsequently, iron(III) nitrate nonahydrate (4.04 g, 10.0 mmol, 1.00 equiv.) was added to the suspension and the pressure tube was quickly resealed. A blast shield was placed in front of the set up and the mixture was then stirred at 40 °C for 18 h. After cooling to 23 °C, the resulting mixture was transferred to a 100 mL round-bottomed flask and concentrated by rotary evaporation under reduced pressure. The residue was diluted with water (50 mL) and extracted with EtOAc (3 × 75 mL). The organic layers were concentrated in a 250 mL round-bottomed flask by rotary evaporation under reduced pressure. DCM (50 mL) and a Teflon-coated

magnetic stir bar were added, followed by pyrrolidine (3.31 mL, 2.84 g, 40.0 mmol, 4.00 equiv.). The pressure tube was sealed and the mixture stirred for 1 h at 23 °C. Subsequently, the mixture was transferred to a 250 mL separatory funnel and water (50 mL) was added, the aqueous phase decanted and further extracted with DCM (3 x 50 mL). The combined organic layers were dried over $MgSO_4$, filtered, and the solvent evaporated by rotary evaporation under reduced pressure. The residue was purified by column chromatography on silica with ethyl acetate in pentane (gradient, 0% for 10 column volumes, then 0%–50% over 50 column volumes) as eluent, to give the desired sulfonamide **18** as a faint red solid (1.85 g, 5.87 mmol, 59%).

10 mmol Scale and catalytic CuCl: Under an ambient atmosphere, to a 350-mL Ace pressure tube equipped with a Teflon-coated magnetic stir bar were added the 4-aminobenzophenone (1.97 g, 10.0 mmol, 1.00 equiv.), $Na_2S_2O_3$ $5H_2O$ (2.48 g, 10.0 mmol, 1.00 equiv.) and CuCl (197 mg, 2.00 mmol, 20 mol%). Then, acetonitrile (50 mL), HCl (4.0 M in 1,4-dioxane, 5.0 mL, 20 mmol, 2.0 equiv.), and SO_2 (3.0 M in MeCN, 32 mL, 0.10 mol, 10 equiv.) were added, the vessel was quickly sealed and the mixture stirred for 5 min at 23 °C. Subsequently, iron(III) nitrate nonahydrate (4.04 g, 10.0 mmol, 1.00 equiv.) was added to the suspension and the pressure tube was quickly resealed. A blast shield was placed in front of the set up and the mixture was then stirred at 40 °C for 18 h. After cooling to 23 °C, the resulting mixture was transferred to a 100 mL round-bottomed flask and concentrated by rotary evaporation under reduced pressure. The residue was diluted with water (50 mL) and extracted with EtOAc (3 × 75 mL). The organic layers were concentrated in a 250 mL round-bottomed flask by rotary evaporation under reduced pressure. DCM (50 mL) and a Teflon-coated magnetic stir bar were added, followed by pyrrolidine (3.31 mL, 2.84 g, 40.0 mmol, 4.00 equiv.). The pressure tube was sealed and the mixture stirred for 1 h at 23 °C. Subsequently, the mixture was transferred to a 250 mL separatory funnel and water (50 mL) was added, the aqueous phase decanted and further extracted with DCM (3 x 50 mL). The combined organic layers were dried over $MgSO_4$, filtered, and the solvent evaporated by rotary evaporation under reduced pressure. Subsequently, $CDCl_3$ (5 mL) and 1,3,5-trimethoxybenzene (1.68 g, 10.0 mmol, 1.00 equiv.) as internal standard were added. A 0.5 mL aliquot was taken and the **yield determined to be 68 %** by ^{1}H NMR spectroscopy at 500 MHz and 298 K by comparing the integration of the 1,3,5-trimethoxybenzene signal at 6.06 ppm (s, 3H) to the signal of **18** at 7.62 ppm (t, 7.6 Hz, 1H).

R_f = 0.28 (EtOAc in cyclohexane = 20%, v/v (UV)).

NMR Spectroscopy:

> **^{1}H NMR** (500 MHz, $CDCl_3$, 23°C, δ): 8.00–7.93 (m, 2H), 7.93–7.88 (m, 2H), 7.84–7.76 (m, 2H), 7.64 (t, *J* = 7.5 Hz, 1H), 7.56–7.48 (m, 2H), 3.52–3.03 (m, 4H), 2.12–1.66 (m, 4H).
>
> **^{13}C NMR** (125 MHz, $CDCl_3$, 23°C, δ): 195.6, 141.3, 140.5, 136.7, 133.4, 130.5, 130.3, 128.8, 127.5, 48.1, 25.5.

HRMS-EI (m/z) calc'd for $C_{17}H_{17}N_1O_3S_1$ $[M]^+$, 315.0924; found, 315.0929; deviation: −1.6 ppm.

1-((6-Bromonaphthalen-2-yl)sulfonyl)pyrrolidine (19)

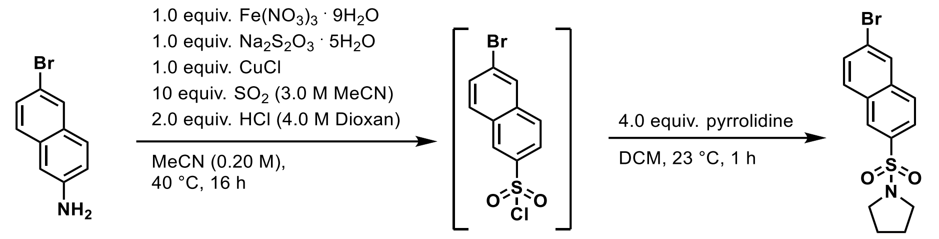

19a **19**, 69%

Caution: A blast shield must be used to minimize personal damage in case of an accident. See safety statement on page S8.

Under an ambient atmosphere, to a 20-mL borosilicate vial equipped with a Teflon-coated magnetic stir bar were added 6-bromnaphthalene-2-amine (111 mg, 0.500 mmol, 1.00 equiv.), $Na_2S_2O_3$ $5H_2O$ (124 mg, 0.500 mmol, 1.00 equiv.) and CuCl (49.5 mg, 0.500 mmol, 1.0 equiv.). Then, acetonitrile (2.5 mL), HCl (4.0 M in 1,4-dioxane, 250 µL, 1.00 mmol, 2.0 equiv.), and SO_2 (3.0 M in MeCN, 1.7 mL, 5.0 mmol, 10 equiv.) were added, the vial was quickly sealed with a septum cap and the mixture stirred for 5 min at 23 °C. Subsequently, iron(III) nitrate nonahydrate (202 mg, 0.500 mmol, 1.00 equiv.) was added to the suspension and the vial was quickly resealed with a septum cap. The mixture was then stirred at 40 °C for 18 h. After cooling to 23 °C, the resulting mixture was concentrated by rotary evaporation under reduced pressure. The residue was diluted with water (5 mL) and extracted with DCM (3 × 5 mL). The organic layers were combined in a 20-mL borosilicate vial and a Teflon-coated magnetic stir bar was added, followed by pyrrolidine (165 µL, 142 mg, 2.00 mmol, 4.00 equiv.). The vessel was sealed with a septum cap and the mixture stirred for 1 h at 23 °C. Subsequently water (5 mL) was added, the aqueous phase decanted and further extracted with DCM (3 × 5 mL). The combined organic layers were dried over $MgSO_4$, filtered, and the solvent evaporated under reduced pressure. The residue was purified by column chromatography on silica with ethyl acetate in cyclohexane (20%, v/v) as eluent, to give the desired sulfonamide **19** as a brown solid (69 mg, 0.35 mmol, 69%).

R_f = 0.33 (EtOAc in pentane = 20%, v/v (UV)).

NMR Spectroscopy:

^{1}H NMR (500 MHz, $CDCl_3$, 23°C, δ): 8.38 (d, J = 1.7 Hz, 1H), 8.10 (s, 1H), 8.00–7.79 (m, 3H), 7.69 (dd, J = 8.8, 2.0 Hz, 1H), 3.52–3.01 (m, 4H), 2.52–1.58 (m, 4H).

^{13}C NMR (125 MHz, $CDCl_3$, 23°C, δ): 135.7, 134.7, 131.0, 130.7, 130.7, 130.1, 128.6, 128.3, 124.1, 123.1, 48.0, 25.3.

HRMS-EI (m/z) calc'd for $C_{14}H_{14}N_1O_2S_1Br_1$ $[M]^+$, 338.9923; found, 338.9925; deviation: −0.6 ppm.

2-Chloro-4-(pyrrolidin-1-ylsulfonyl)benzonitrile (20)

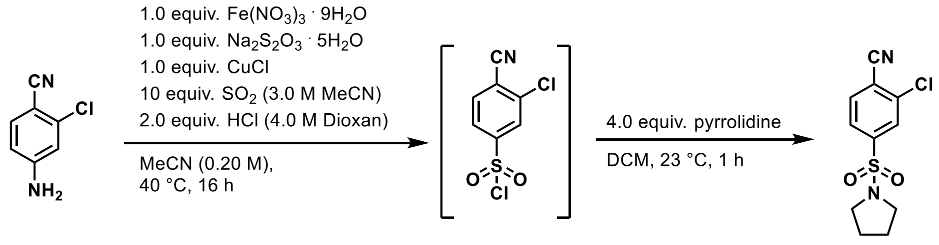

Caution: A blast shield must be used to minimize personal damage in case of an accident. See safety statement on page S8.

Under an ambient atmosphere, to a 20-mL borosilicate vial equipped with a Teflon-coated magnetic stir bar were added 4-amino-2-chlorobenzonitrile (76.3 mg, 0.500 mmol, 1.00 equiv.), $Na_2S_2O_3$ $5H_2O$ (124 mg, 0.500 mmol, 1.00 equiv.) and CuCl (49.5 mg, 0.500 mmol, 1.0 equiv.). Then, acetonitrile (2.5 mL), HCl (4.0 M in 1,4-dioxane, 250 µL, 1.00 mmol, 2.0 equiv.), and SO_2 (3.0 M in MeCN, 1.7 mL, 5.0 mmol, 10 equiv.) were added, the vial was quickly sealed with a septum cap and the mixture stirred for 5 min at 23 °C. Subsequently, iron(III) nitrate nonahydrate (202 mg, 0.500 mmol, 1.00 equiv.) was added to the suspension and the vial was quickly resealed with a septum cap. The mixture was then stirred at 40 °C for 18 h. After cooling to 23 °C, the resulting mixture was concentrated by rotary evaporation under reduced pressure. The residue was diluted with water (5 mL) and extracted with DCM (3 × 5 mL). The organic layers were combined in a 20-mL borosilicate vial and a Teflon-coated magnetic stir bar was added, followed by pyrrolidine (165 µL, 142 mg, 2.00 mmol, 4.00 equiv.). The vessel was sealed with a septum cap and the mixture stirred for 1 h at 23 °C. Subsequently water (5 mL) was added, the aqueous phase decanted and further extracted with DCM (3 × 5 mL). The combined organic layers were dried over $MgSO_4$, filtered, and the solvent evaporated under reduced pressure. The residue was purified by column chromatography on silica with ethyl acetate in pentane (12%, v/v) as eluent, to give the desired sulfonamide **20** as an off-white solid (87 mg, 0.41 mmol, 82%).

R_f = 0.34 (EtOAc in pentane = 30%, v/v (UV)).

NMR Spectroscopy:

^{1}H NMR (500 MHz, $CDCl_3$, 23°C, δ): 7.98 (d, J = 1.6 Hz, 1H), 7.94–7.80 (m, 2H), 3.58–3.06 (m, 4H), 2.65–1.68 (m, 4H).

^{13}C NMR (125 MHz, $CDCl_3$, 23°C, δ): 143.0, 138.1, 134.7, 128.5, 125.7, 117.0, 114.8, 48.1, 25.4.

HRMS-EI (m/z) calc'd for $C_{11}H_{11}N_2O_2S_1Cl_1$ $[M]^+$, 270.0224; found, 270.0226; deviation: −0.8 ppm.

4-(Pyrrolidin-1-ylsulfonyl)trifluoromethoxybenzene (21)

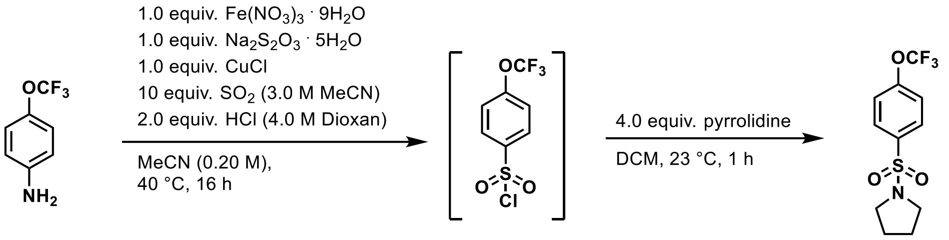

21a **21**, 68%

Caution: A blast shield must be used to minimize personal damage in case of an accident. See safety statement on page S8.

Under an ambient atmosphere, to a 20-mL borosilicate vial equipped with a Teflon-coated magnetic stir bar were added 4-trifluoromethoxyaniline (88.6 mg, 0.500 mmol, 1.00 equiv.), $Na_2S_2O_3$ $5H_2O$ (124 mg, 0.500 mmol, 1.00 equiv.) and CuCl (49.5 mg, 0.500 mmol, 1.0 equiv.). Then, acetonitrile (2.5 mL), HCl (4.0 M in 1,4-dioxane, 250 µL, 1.00 mmol, 2.0 equiv.), and SO_2 (3.0 M in MeCN, 1.7 mL, 5.0 mmol, 10 equiv.) were added, the vial was quickly sealed with a septum cap and the mixture stirred for 5 min at 23 °C. Subsequently, iron(III) nitrate nonahydrate (202 mg, 0.500 mmol, 1.00 equiv.) was added to the suspension and the vial was quickly resealed with a septum cap. The mixture was then stirred at 40 °C for 18 h. After cooling to 23 °C, the resulting mixture was concentrated by rotary evaporation under reduced pressure. The residue was diluted with water (5 mL) and extracted with DCM (3 × 5 mL). The organic layers were combined in a 20-mL borosilicate vial and a Teflon-coated magnetic stir bar was added, followed by pyrrolidine (165 µL, 142 mg, 2.00 mmol, 4.00 equiv.). The vessel was sealed with a septum cap and the mixture stirred for 1 h at 23 °C. Subsequently water (5 mL) was added, the aqueous phase decanted and further extracted with DCM (3 × 5 mL). The combined organic layers were dried over $MgSO_4$, filtered, and the solvent evaporated under reduced pressure. The residue was purified by column chromatography on silica with ethyl acetate in cyclohexane (gradient, 0%–50% over 30 column volumes) as eluent, to give the desired sulfonamide **21** as a colorless solid (99 mg, 0.34 mmol, 68%).

R_f = 0.38 (EtOAc in cyclohexane = 20%, v/v (UV)).

NMR Spectroscopy:

^{1}H NMR (500 MHz, $CDCl_3$, 23°C, δ): 7.96–7.79 (m, 2H), 7.35 (dd, J = 8.9, 1.0 Hz, 2H), 3.26 (t, J = 6.8 Hz, 4H), 2.27–1.69 (m, 4H).

^{13}C NMR (125 MHz, $CDCl_3$, 23°C, δ): 152.2 (d, J = 1.8 Hz), 135.8, 129.7, 121.0 (d, J = 1.4 Hz), 120.4 (q, J = 259.3 Hz), 48.1, 25.4.

^{19}F NMR (565 MHz, $CDCl_3$, 23°C, δ): −57.69

HRMS-EI (m/z) calc'd for $C_{11}H_{12}N_1O_3S_1F_3$ $[M]^+$, 295.0485; found, 295.0489; deviation: −1.5 ppm.

1-((2-((Trifluoromethyl)thio)phenyl)sulfonyl)pyrrolidine (22)

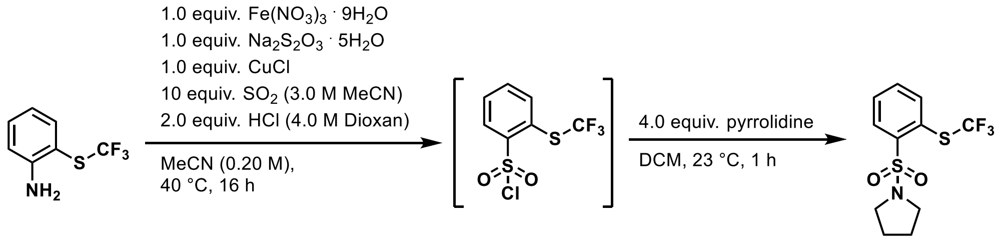

22a **22**, 80%

Caution: A blast shield must be used to minimize personal damage in case of an accident. See safety statement on page S8.

Under an ambient atmosphere, to a 20-mL borosilicate vial equipped with a Teflon-coated magnetic stir bar were added 2-(trifluoromethylthio)aniline (96.6 mg, 0.500 mmol, 1.00 equiv.), $Na_2S_2O_3$ $5H_2O$ (124 mg, 0.500 mmol, 1.00 equiv.) and CuCl (49.5 mg, 0.500 mmol, 1.0 equiv.). Then, acetonitrile (2.5 mL), HCl (4.0 M in 1,4-dioxane, 250 µL, 1.00 mmol, 2.0 equiv.), and SO_2 (3.0 M in MeCN, 1.7 mL, 5.0 mmol, 10 equiv.) were added, the vial was quickly sealed with a septum cap and the mixture stirred for 5 min at 23 °C. Subsequently, iron(III) nitrate nonahydrate (202 mg, 0.500 mmol, 1.00 equiv.) was added to the suspension and the vial was quickly resealed with a septum cap. The mixture was then stirred at 40 °C for 18 h. After cooling to 23 °C, the resulting mixture was concentrated by rotary evaporation under reduced pressure. The residue was diluted with water (5 mL) and extracted with DCM (3 × 5 mL). The organic layers were combined in a 20-mL borosilicate vial and a Teflon-coated magnetic stir bar was added, followed by pyrrolidine (165 µL, 142 mg, 2.00 mmol, 4.00 equiv.). The vessel was sealed with a septum cap and the mixture stirred for 1 h at 23 °C. Subsequently water (5 mL) was added, the aqueous phase decanted and further extracted with DCM (3 × 5 mL). The combined organic layers were dried over $MgSO_4$, filtered, and the solvent evaporated under reduced pressure. The residue was purified by column chromatography on silica with ethyl acetate in pentane (12% v/v) as eluent, to give the desired sulfonamide **22** as a yellow oil (98 mg, 0.40 mmol, 80%).

R_f = 0.47 (EtOAc in pentane = 20%, v/v (UV)).

NMR Spectroscopy:

^{1}H NMR (500 MHz, $CDCl_3$, 23°C, δ): 8.15 (dd, *J* = 7.7, 1.7 Hz, 1H), 7.84 (dt, *J* = 7.7, 1.2 Hz, 1H), 7.58 (dtd, *J* = 22.1, 7.6, 1.6 Hz, 2H), 3.65–3.23 (m, 4H), 2.40–1.69 (m, 4H).

^{13}C NMR (125 MHz, $CDCl_3$, 23°C, δ): 141.9, 136.1, 132.9, 131.4, 129.9, 129.4 (q, *J* = 309.1 Hz), 125.1 (q, *J* = 2.3 Hz), 47.6, 25.7.

^{19}F NMR (565 MHz, $CDCl_3$, 23°C, δ): −40.73

HRMS-EI (m/z) calc'd for $C_{11}H_{12}N_1O_2S_2F_3$ $[M]^+$, 311.0256; found, 311.0258; deviation: −0.6 ppm.

3-Bromo-4-(pyrrolidin-1-ylsulfonyl)benzonitrile (23)

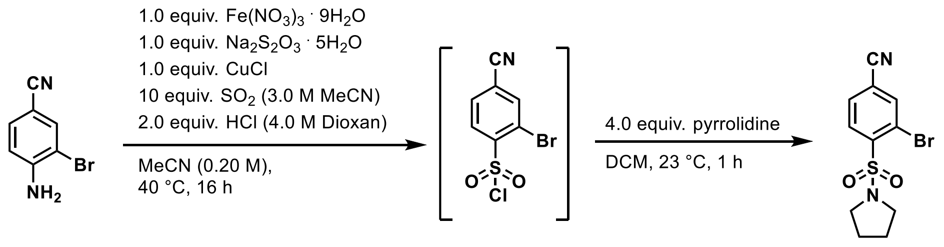

23a **23**, 55%

Caution: A blast shield must be used to minimize personal damage in case of an accident. See safety statement on page S8.

Under an ambient atmosphere, to a 20-mL borosilicate vial equipped with a Teflon-coated magnetic stir bar were added 4-amino-3-bromobenzonitrile (98.5 mg, 0.500 mmol, 1.00 equiv.), $Na_2S_2O_3$ $5H_2O$ (124 mg, 0.500 mmol, 1.00 equiv.) and CuCl (49.5 mg, 0.500 mmol, 1.0 equiv.). Then, acetonitrile (2.5 mL), HCl (4.0 M in 1,4-dioxane, 250 µL, 1.00 mmol, 2.0 equiv.), and SO_2 (3.0 M in MeCN, 1.7 mL, 5.0 mmol, 10 equiv.) were added, the vial was quickly sealed with a septum cap and the mixture stirred for 5 min at 23 °C. Subsequently, iron(III) nitrate nonahydrate (202 mg, 0.500 mmol, 1.00 equiv.) was added to the suspension and the vial was quickly resealed with a septum cap. The mixture was then stirred at 40 °C for 18 h. After cooling to 23 °C, the resulting mixture was concentrated by rotary evaporation under reduced pressure. The residue was diluted with water (5 mL) and extracted with DCM (3 × 5 mL). The organic layers were combined in a 20-mL borosilicate vial and a Teflon-coated magnetic stir bar was added, followed by pyrrolidine (165 µL, 142 mg, 2.00 mmol, 4.00 equiv.). The vessel was sealed with a septum cap and the mixture stirred for 1 h at 23 °C. Subsequently water (5 mL) was added, the aqueous phase decanted and further extracted with DCM (3 × 5 mL). The combined organic layers were dried over $MgSO_4$, filtered, and the solvent evaporated under reduced pressure. The residue was purified by column chromatography on silica with ethyl acetate in pentane (14%, v/v) as eluent, to give the desired sulfonamide **23** as an off-white solid (87 mg, 0.28 mmol, 55%).

R_f = 0.40 (EtOAc in pentane = 30%, v/v (UV)).

NMR Spectroscopy:

^{1}H NMR (500 MHz, $CDCl_3$, 23°C, δ): 8.21 (d, *J* = 8.2 Hz, 1H), 8.03 (d, *J* = 1.7 Hz, 1H), 7.75 (dd, *J* = 8.1, 1.7 Hz, 1H), 3.47–3.31 (m, 4H), 2.43–1.70 (m, 4H).

^{13}C NMR (125 MHz, $CDCl_3$, 23°C, δ): 143.4, 138.6, 132.4, 131.0, 121.1, 117.0, 116.1, 48.1, 25.8.

HRMS-ESI (m/z) calc'd for $C_{11}H_{11}N_2O_2S_1Br_1Na_1$ $[M+Na]^+$, 336.9617; found, 336.9615; deviation: +0.7 ppm.

1-((2-Methyl-3-nitrophenyl)sulfonyl)pyrrolidine (24)

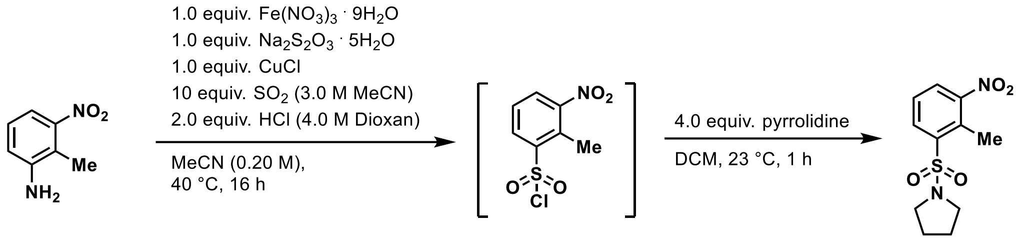

24a **24**, 81%

Caution: A blast shield must be used to minimize personal damage in case of an accident. See safety statement on page S8.

Under an ambient atmosphere, to a 20-mL borosilicate vial equipped with a Teflon-coated magnetic stir bar were added 2-methyl-3-nitroaniline (76.1 mg, 0.500 mmol, 1.00 equiv.), $Na_2S_2O_3$ $5H_2O$ (124 mg, 0.500 mmol, 1.00 equiv.) and CuCl (49.5 mg, 0.500 mmol, 1.0 equiv.). Then, acetonitrile (2.5 mL), HCl (4.0 M in 1,4-dioxane, 250 µL, 1.00 mmol, 2.0 equiv.), and SO_2 (3.0 M in MeCN, 1.7 mL, 5.0 mmol, 10 equiv.) were added, the vial was quickly sealed with a septum cap and the mixture stirred for 5 min at 23 °C. Subsequently, iron(III) nitrate nonahydrate (202 mg, 0.500 mmol, 1.00 equiv.) was added to the suspension and the vial was quickly resealed with a septum cap. The mixture was then stirred at 40 °C for 18 h. After cooling to 23 °C, the resulting mixture was concentrated by rotary evaporation under reduced pressure. The residue was diluted with water (5 mL) and extracted with DCM (3 × 5 mL). The organic layers were combined in a 20-mL borosilicate vial and a Teflon-coated magnetic stir bar was added, followed by pyrrolidine (165 µL, 142 mg, 2.00 mmol, 4.00 equiv.). The vessel was sealed with a septum cap and the mixture stirred for 1 h at 23 °C. Subsequently water (5 mL) was added, the aqueous phase decanted and further extracted with DCM (3 × 5 mL). The combined organic layers were dried over $MgSO_4$, filtered, and the solvent evaporated under reduced pressure. The residue was purified by column chromatography on silica with ethyl acetate in pentane (gradient, 0%–50% over 30 column volumes) as eluent, to give the desired sulfonamide **24** as a colorless solid (0.11 g, 0.41 mmol, 81%).

R_f = 0.28 (EtOAc in pentane = 20%, v/v (UV)).

NMR Spectroscopy:

^{1}H NMR (500 MHz, $CDCl_3$, 23°C, δ): 8.17 (ddd, *J* = 8.0, 1.4, 0.5 Hz, 1H), 7.86 (ddd, *J* = 8.1, 1.4, 0.5 Hz, 1H), 7.46 (td, *J* = 8.0, 0.6 Hz, 1H), 3.35 (t, *J* = 6.8 Hz, 4H), 2.71 (s, 3H), 2.24–1.82 (m, 4H).

^{13}C NMR (125 MHz, $CDCl_3$, 23°C, δ): 152.8, 140.8, 133.0, 132.2, 127.6, 126.8, 47.7, 25.9, 15.5.

HRMS-EI (m/z) calc'd for $C_{11}H_{14}N_2O_4S_1$ $[M]^+$, 270.0669; found, 270.0669; deviation: −0.4 ppm.

4-(pyrrolidin-1-ylsulfonyl)benzenesulfonamide (25)

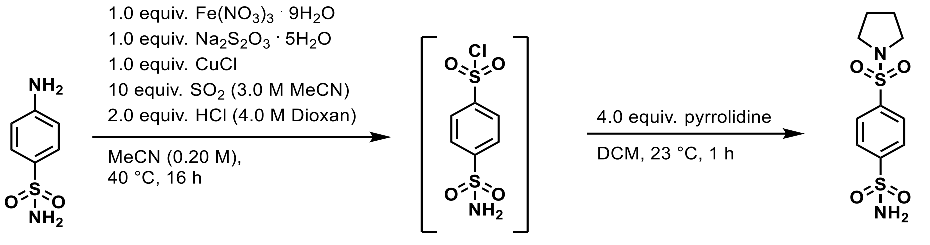

Caution: A blast shield must be used to minimize personal damage in case of an accident. See safety statement on page S8.

Under an ambient atmosphere, to a 20-mL borosilicate vial equipped with a Teflon-coated magnetic stir bar were added sulfanilamide (59.1 mg, 0.500 mmol, 1.00 equiv.), $Na_2S_2O_3$ $5H_2O$ (124 mg, 0.500 mmol, 1.00 equiv.) and CuCl (49.5 mg, 0.500 mmol, 1.0 equiv.). Then, acetonitrile (2.5 mL), HCl (4.0 M in 1,4-dioxane, 250 µL, 1.00 mmol, 2.0 equiv.), and SO_2 (3.0 M in MeCN, 1.7 mL, 5.0 mmol, 10 equiv.) were added, the vial was quickly sealed with a septum cap and the mixture stirred for 5 min at 23 °C. Subsequently, iron(III) nitrate nonahydrate (202 mg, 0.500 mmol, 1.00 equiv.) was added to the suspension and the vial was quickly resealed with a septum cap. The mixture was then stirred at 40 °C for 18 h. After cooling to 23 °C, the resulting mixture was concentrated by rotary evaporation under reduced pressure. The residue was diluted with water (5 mL) and extracted with DCM (3 × 5 mL). The organic layers were combined in a 20-mL borosilicate vial and a Teflon-coated magnetic stir bar was added, followed by pyrrolidine (165 µL, 142 mg, 2.00 mmol, 4.00 equiv.). The vessel was sealed with a septum cap and the mixture stirred for 1 h at 23 °C. Subsequently water (5 mL) was added, the aqueous phase decanted and further extracted with DCM (3 × 5 mL). The combined organic layers were dried over $MgSO_4$, filtered, and the solvent evaporated under reduced pressure. The residue was purified by column chromatography on silica with ethyl acetate in pentane (40%, v/v) as eluent, to give the desired sulfonamide **25** as a colorless solid (78 mg, 0.27 mmol, 54%).

R*f* = 0.64 (EtOAc in pentane = 70%, v/v (UV)).

NMR Spectroscopy:

^{1}H NMR (500 MHz, DMSO-d_6, 23°C, δ): 8.04 (q, J = 8.6 Hz, 4H), 3.25–2.94 (m, 4H), 2.12–1.46 (m, 4H).

^{13}C NMR (125 MHz, DMSO-d_6, 23°C, δ): 148.2, 139.7, 128.6, 127.2, 48.4, 25.3.

HRMS-ESI (m/z) calc'd for $C_{10}H_{14}N_2O_4S_2Na_1$ [M+Na]$^+$, 313.0287; found, 313.0289; deviation: −0.5 ppm.

1-((3,5-Dibromo-4-methylphenyl)sulfonyl)pyrrolidine (26)

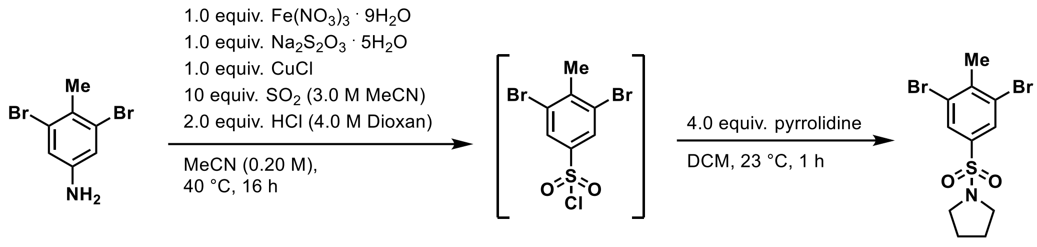

26a

26, 68%

Caution: A blast shield must be used to minimize personal damage in case of an accident. See safety statement on page S8.

Under an ambient atmosphere, to a 20-mL borosilicate vial equipped with a Teflon-coated magnetic stir bar were added 3,5-dibromo-4-methylaniline (133 mg, 0.500 mmol, 1.00 equiv.), $Na_2S_2O_3$ $5H_2O$ (124 mg, 0.500 mmol, 1.00 equiv.) and CuCl (49.5 mg, 0.500 mmol, 1.0 equiv.). Then, acetonitrile (2.5 mL), HCl (4.0 M in 1,4-dioxane, 250 µL, 1.00 mmol, 2.0 equiv.), and SO_2 (3.0 M in MeCN, 1.7 mL, 5.0 mmol, 10 equiv.) were added, the vial was quickly sealed with a septum cap and the mixture stirred for 5 min at 23 °C. Subsequently, iron(III) nitrate nonahydrate (202 mg, 0.500 mmol, 1.00 equiv.) was added to the suspension and the vial was quickly resealed with a septum cap. The mixture was then stirred at 40 °C for 18 h. After cooling to 23 °C, the resulting mixture was concentrated by rotary evaporation under reduced pressure. The residue was diluted with water (5 mL) and extracted with DCM (3 × 5 mL). The organic layers were combined in a 20-mL borosilicate vial and a Teflon-coated magnetic stir bar was added, followed by pyrrolidine (165 µL, 142 mg, 2.00 mmol, 4.00 equiv.). The vessel was sealed with a septum cap and the mixture stirred for 1 h at 23 °C. Subsequently water (5 mL) was added, the aqueous phase decanted and further extracted with DCM (3 × 5 mL). The combined organic layers were dried over $MgSO_4$, filtered, and the solvent evaporated under reduced pressure. The residue was purified by column chromatography on silica with ethyl acetate in pentane (gradient, 0%–50% over 30 column volumes) as eluent, to give the desired sulfonamide **26** as a colorless solid (0.13 g, 0.34 mmol, 68%).

$\mathbf{R}_f$ = 0.51 (EtOAc in pentane = 20%, v/v (UV)).

NMR Spectroscopy:

^{1}H NMR (500 MHz, $CDCl_3$, 23°C, δ): 7.98 (s, 2H), 3.37–3.17 (m, 4H), 2.66 (s, 3H), 2.39–1.71 (m, 4H).

^{13}C NMR (125 MHz, $CDCl_3$, 23°C, δ): 142.7, 137.2, 130.4, 126.0, 48.2, 25.5, 24.2.

HRMS-EI (m/z) calc'd for $C_{11}H_{13}N_1O_2S_1Br_2$ $[M]^+$, 380.9029; found, 380.9034; deviation: −1.4 ppm.

4-(Pyrrolidin-1-ylsulfonyl)difluoromethoxybenzene (27)

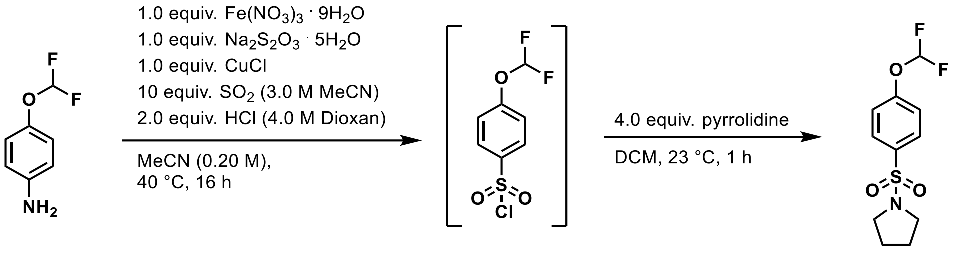

Caution: A blast shield must be used to minimize personal damage in case of an accident. See safety statement on page S8.

Under an ambient atmosphere, to a 20-mL borosilicate vial equipped with a Teflon-coated magnetic stir bar were added 4-difluoromethoxyaniline (79.6 mg, 0.500 mmol, 1.00 equiv.), $Na_2S_2O_3$ $5H_2O$ (124 mg, 0.500 mmol, 1.00 equiv.) and CuCl (49.5 mg, 0.500 mmol, 1.0 equiv.). Then, acetonitrile (2.5 mL), HCl (4.0 M in 1,4-dioxane, 250 µL, 1.00 mmol, 2.0 equiv.), and SO_2 (3.0 M in MeCN, 1.7 mL, 5.0 mmol, 10 equiv.) were added, the vial was quickly sealed with a septum cap and the mixture stirred for 5 min at 23 °C. Subsequently, iron(III) nitrate nonahydrate (202 mg, 0.500 mmol, 1.00 equiv.) was added to the suspension and the vial was quickly resealed with a septum cap. The mixture was then stirred at 40 °C for 18 h. After cooling to 23 °C, the resulting mixture was concentrated by rotary evaporation under reduced pressure. The residue was diluted with water (5 mL) and extracted with DCM (3 × 5 mL). The organic layers were combined in a 20-mL borosilicate vial and a Teflon-coated magnetic stir bar was added, followed by pyrrolidine (165 µL, 142 mg, 2.00 mmol, 4.00 equiv.). The vessel was sealed with a septum cap and the mixture stirred for 1 h at 23 °C. Subsequently water (5 mL) was added, the aqueous phase decanted and further extracted with DCM (3 × 5 mL). The combined organic layers were dried over $MgSO_4$, filtered, and the solvent evaporated under reduced pressure. The residue was purified by column chromatography on silica with ethyl acetate in cyclohexane (gradient, 0%–50% over 30 column volumes) as eluent, to give the desired sulfonamide **27** as a colorless solid (82 mg, 0.30 mmol, 60%).

R*f* = 0.26 (EtOAc in cyclohexane = 20%, v/v (UV)).

NMR Spectroscopy:

^{1}H NMR (500 MHz, $CDCl_3$, 23°C, δ): 7.85 (d, *J* = 8.8 Hz, 2H), 7.42–7.16 (m, 2H), 6.60 (t, *J* = 72.7 Hz, 1H), 3.24 (t, *J* = 6.8 Hz, 4H), 2.15–1.69 (m, 4H).

^{13}C NMR (125 MHz, $CDCl_3$, 23°C, δ): 154.2 (t, *J* = 2.9 Hz), 134.1, 129.7, 119.5, 115.4 (t, *J* = 262.4 Hz), 48.1, 25.4.

^{19}F NMR (565 MHz, $CDCl_3$, 23°C, δ): −81.98 (d, *J* = 72.0 Hz).

HRMS-EI (m/z) calc’d for $C_{11}H_{13}N_1O_3S_1F_2$ $[M]^+$, 277.0579; found, 277.0579; deviation: −0.1 ppm.

4-Methyl-7-(pyrrolidin-1-ylsulfonyl)-2*H*-chromen-2-one (28)

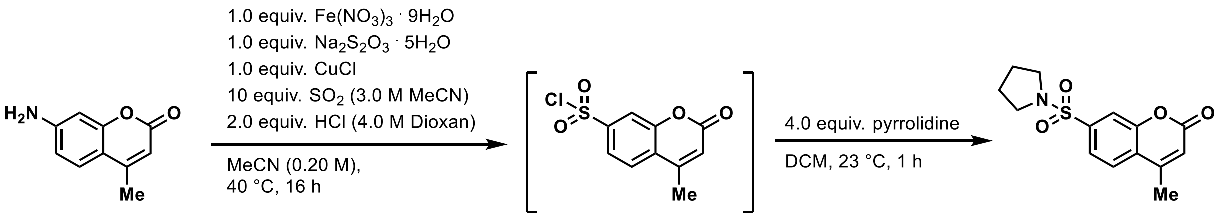

28a **28**, 73%

Caution: A blast shield must be used to minimize personal damage in case of an accident. See safety statement on page S8.

Under an ambient atmosphere, to a 20-mL borosilicate vial equipped with a Teflon-coated magnetic stir bar were added 7-amino-4-methylcumarin (87.6 mg, 0.500 mmol, 1.00 equiv.), $Na_2S_2O_3$ $5H_2O$ (124 mg, 0.500 mmol, 1.00 equiv.) and CuCl (49.5 mg, 0.500 mmol, 1.0 equiv.). Then, acetonitrile (2.5 mL), HCl (4.0 M in 1,4-dioxane, 250 µL, 1.00 mmol, 2.0 equiv.), and SO_2 (3.0 M in MeCN, 1.7 mL, 5.0 mmol, 10 equiv.) were added, the vial was quickly sealed with a septum cap and the mixture stirred for 5 min at 23 °C. Subsequently, iron(III) nitrate nonahydrate (202 mg, 0.500 mmol, 1.00 equiv.) was added to the suspension and the vial was quickly resealed with a septum cap. The mixture was then stirred at 40 °C for 18 h. After cooling to 23 °C, the resulting mixture was concentrated by rotary evaporation under reduced pressure. The residue was diluted with water (5 mL) and extracted with DCM (3 × 5 mL). The organic layers were combined in a 20-mL borosilicate vial and a Teflon-coated magnetic stir bar was added, followed by pyrrolidine (165 µL, 142 mg, 2.00 mmol, 4.00 equiv.). The vessel was sealed with a septum cap and the mixture stirred for 1 h at 23 °C. Subsequently water (5 mL) was added, the aqueous phase decanted and further extracted with DCM (3 × 5 mL). The combined organic layers were dried over $MgSO_4$, filtered, and the solvent evaporated under reduced pressure. The residue was purified by column chromatography on silica with ethyl acetate in cyclohexane (gradient, 0%–100% over 30 column volumes) as eluent, to give the desired sulfonamide **28** as a colorless solid (107 mg, 0.37 mmol, 73%).

R*f* = 0.27 (EtOAc in cyclohexane = 50%, v/v (UV)).

NMR Spectroscopy:

^{1}H NMR (500 MHz, $CDCl_3$, 23°C, δ): 7.95–7.64 (m, 3H), 6.42 (d, *J* = 1.4 Hz, 1H), 3.41– .22 (m, 4H), 2.48 (d, *J* = 1.3 Hz, 3H), 2.16–1.72 (m, 4H).

^{13}C NMR (125 MHz, $CDCl_3$, 23°C, δ): 159.7, 153.5, 151.3, 140.4, 125.7, 123.2, 122.8, 117.5, 116.3, 48.2, 25.5, 18.9.

HRMS-EI (m/z) calc'd for $C_{14}H_{15}N_1O_4S_1$ $[M]^+$, 293.0716; found, 293.0714; deviation: +0.9 ppm.

Methyl 3-(pyrrolidin-1-ylsulfonyl)thiophene-2-carboxylate (29)

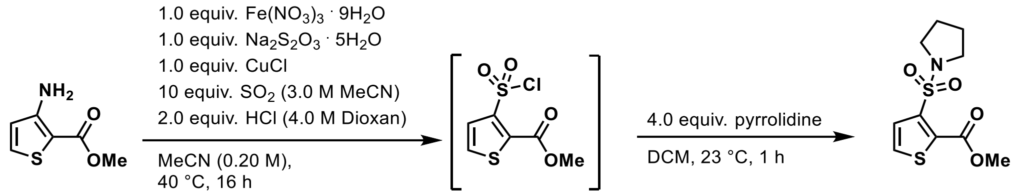

Caution: A blast shield must be used to minimize personal damage in case of an accident. See safety statement on page S8.

Under an ambient atmosphere, to a 20-mL borosilicate vial equipped with a Teflon-coated magnetic stir bar were added methyl 3-amino-2-thiophenecarboxylate (78.6 mg, 0.500 mmol, 1.00 equiv.), $Na_2S_2O_3$ $5H_2O$ (124 mg, 0.500 mmol, 1.00 equiv.) and CuCl (49.5 mg, 0.500 mmol, 1.0 equiv.). Then, acetonitrile (2.5 mL), HCl (4.0 M in 1,4-dioxane, 250 µL, 1.00 mmol, 2.0 equiv.), and SO_2 (3.0 M in MeCN, 1.7 mL, 5.0 mmol, 10 equiv.) were added, the vial was quickly sealed with a septum cap and the mixture stirred for 5 min at 23 °C. Subsequently, iron(III) nitrate nonahydrate (202 mg, 0.500 mmol, 1.00 equiv.) was added to the suspension and the vial was quickly resealed with a septum cap. The mixture was then stirred at 40 °C for 18 h. After cooling to 23 °C, the resulting mixture was concentrated by rotary evaporation under reduced pressure. The residue was diluted with water (5 mL) and extracted with DCM (3 × 5 mL). The organic layers were combined in a 20-mL borosilicate vial and a Teflon-coated magnetic stir bar was added, followed by pyrrolidine (165 µL, 142 mg, 2.00 mmol, 4.00 equiv.). The vessel was sealed with a septum cap and the mixture stirred for 1 h at 23 °C. Subsequently water (5 mL) was added, the aqueous phase decanted and further extracted with DCM (3 × 5 mL). The combined organic layers were dried over $MgSO_4$, filtered, and the solvent evaporated under reduced pressure. The residue was purified by column chromatography on silica with ethyl acetate in pentane (13%, v/v) as eluent, to give the desired sulfonamide **29** as a colorless oil (96 mg, 0.35 mmol, 70%).

R*f* = 0.34 (EtOAc in pentane = 40%, v/v (UV)).

NMR Spectroscopy:

> **^{1}H NMR** (500 MHz, $CDCl_3$, 23°C, δ): 7.49 (d, *J* = 5.2 Hz, 1H), 7.45 (d, *J* = 5.3 Hz, 1H), 3.91 (s, 3H), 3.62–3.34 (m, 4H), 2.39–1.58 (m, 4H).
>
> **^{13}C NMR** (125 MHz, $CDCl_3$, 23°C, δ): 160.3, 141.4, 133.6, 131.1, 129.0, 53.0, 48.0, 25.7.

HRMS-ESI (m/z) calc'd for $C_{10}H_{13}N_1O_4S_2Na_1$ $[M+Na]^+$, 298.0178; found, 298.0175; deviation: +1.1 ppm.

1-((2,2-Difluorobenzo[d][1,3]dioxol-4-yl)sulfonyl)pyrrolidine (30)

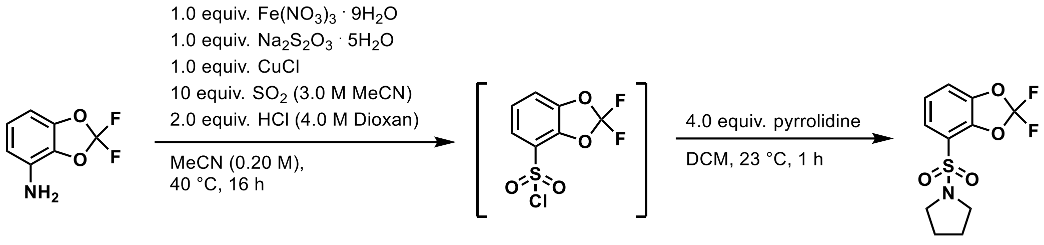

30a **30**, 56%

Caution: A blast shield must be used to minimize personal damage in case of an accident. See safety statement on page S8.

Under an ambient atmosphere, to a 20-mL borosilicate vial equipped with a Teflon-coated magnetic stir bar were added 2,2-difluorobenzo[d][1,3]dioxol-5-amine (86.6 mg, 0.500 mmol, 1.00 equiv.), $Na_2S_2O_3$ $5H_2O$ (124 mg, 0.500 mmol, 1.00 equiv.) and CuCl (49.5 mg, 0.500 mmol, 1.0 equiv.). Then, acetonitrile (2.5 mL), HCl (4.0 M in 1,4-dioxane, 250 µL, 1.00 mmol, 2.0 equiv.), and SO_2 (3.0 M in MeCN, 1.7 mL, 5.0 mmol, 10 equiv.) were added, the vial was quickly sealed with a septum cap and the mixture stirred for 5 min at 23 °C. Subsequently, iron(III) nitrate nonahydrate (202 mg, 0.500 mmol, 1.00 equiv.) was added to the suspension and the vial was quickly resealed with a septum cap. The mixture was then stirred at 40 °C for 18 h. After cooling to 23 °C, the resulting mixture was concentrated by rotary evaporation under reduced pressure. The residue was diluted with water (5 mL) and extracted with DCM (3 × 5 mL). The organic layers were combined in a 20-mL borosilicate vial and a Teflon-coated magnetic stir bar was added, followed by pyrrolidine (165 µL, 142 mg, 2.00 mmol, 4.00 equiv.). The vessel was sealed with a septum cap and the mixture stirred for 1 h at 23 °C. Subsequently water (5 mL) was added, the aqueous phase decanted and further extracted with DCM (3 × 5 mL). The combined organic layers were dried over $MgSO_4$, filtered, and the solvent evaporated under reduced pressure. The residue was purified by column chromatography on silica with ethyl acetate in pentane (gradient, 0%–50% over 30 column volumes) as eluent, to give the desired sulfonamide **30** as a colorless solid (81 mg, 0.28 mmol, 56%).

R_f = 0.36 (EtOAc in pentane = 20%, v/v (UV)).

NMR Spectroscopy:

^{1}H NMR (500 MHz, $CDCl_3$, 23°C, δ): 7.65 (dd, J = 8.3, 1.8 Hz, 1H), 7.55 (dd, J = 1.8, 0.4 Hz, 1H), 7.20 (dd, J = 8.3, 0.4 Hz, 1H), 3.25 (t, J = 6.8 Hz, 4H), 2.02–1.75 (m, 4H).

^{13}C NMR (125 MHz, $CDCl_3$, 23°C, δ): 146.7, 144.0, 133.5, 131.8, 130.1, 124.4, 109.6 (d, J = 88.0 Hz), 48.1, 25.4.

^{19}F NMR (565 MHz, $CDCl_3$, 23°C, δ): −49.31

HRMS-EI (m/z) calc'd for $C_{11}H_{11}N_1O_4S_1F_2$ $[M]^+$, 291.0371; found, 291.0372; deviation: −0.1 ppm.

Hippuric acid-derived sulfonamide 31

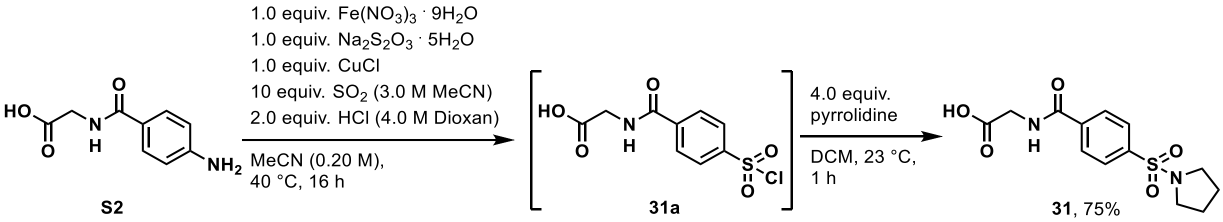

Caution: A blast shield must be used to minimize personal damage in case of an accident. See safety statement on page S8.

Under an ambient atmosphere, to a 20-mL borosilicate vial equipped with a Teflon-coated magnetic stir bar were added reduced *p*-aminohippuric acid **S2** (97.1 mg, 0.500 mmol, 1.00 equiv.), $Na_2S_2O_3$ $5H_2O$ (124 mg, 0.500 mmol, 1.00 equiv.) and CuCl (49.5 mg, 0.500 mmol, 1.0 equiv.). Then, acetonitrile (2.5 mL), HCl (4.0 M in 1,4-dioxane, 250 µL, 1.00 mmol, 2.0 equiv.), and SO_2 (3.0 M in MeCN, 1.7 mL, 5.0 mmol, 10 equiv.) were added, the vial was quickly sealed with a septum cap and the mixture stirred for 5 min at 23 °C. Subsequently, iron(III) nitrate nonahydrate (202 mg, 0.500 mmol, 1.00 equiv.) was added to the suspension and the vial was quickly resealed with a septum cap. The mixture was then stirred at 40 °C for 18 h. After cooling to 23 °C, the resulting mixture was concentrated by rotary evaporation under reduced pressure. The residue was diluted with water (5 mL) and extracted with DCM (3 × 5 mL). The organic layers were combined in a 20-mL borosilicate vial and a Teflon-coated magnetic stir bar was added, followed by pyrrolidine (165 µL, 142 mg, 2.00 mmol, 4.00 equiv.). The vessel was sealed with a septum cap and the mixture stirred for 1 h at 23 °C. Subsequently water (5 mL) was added, the aqueous phase decanted and further extracted with DCM (3 × 5 mL). The combined organic layers were dried over $MgSO_4$, filtered, and the solvent evaporated under reduced pressure. The residue was purified by column chromatography on silica with MeOH in DCM (10% + 2% AcOH, v/v) as eluent, to give the desired sulfonamide **33** as a light-yellow solid (0.12 g, 0.38 mmol, 75%).

R*f* = 0.61 (MeOH in DCM = 10% + 2% AcOH, v/v (UV)).

NMR Spectroscopy:

^{1}H NMR (500 MHz, DMSO-*d*$_6$, 23°C, δ): 9.05 (t, *J* = 5.2 Hz, 1H), 8.09 (d, *J* = 8.1 Hz, 2H), 7.92 (d, *J* = 8.0 Hz, 2H), 3.95 (s, 2H), 3.32–2.95 (m, 4H), 1.92 (s, 1H), 1.64 (q, *J* = 3.4 Hz, 4H).

^{13}C NMR (125 MHz, DMSO-*d*$_6$, 23°C, δ): 171.7, 165.7, 139.0, 138.2, 128.3, 127.9, 48.3, 42.1, 25.2.

HRMS-ESI (m/z) calc'd for $C_{13}H_{15}N_2O_5S_1$ $[M-H]^-$, 311.0707; found, 311.0710; deviation: −0.7 ppm.

Flutamide-derived sulfonamide 32

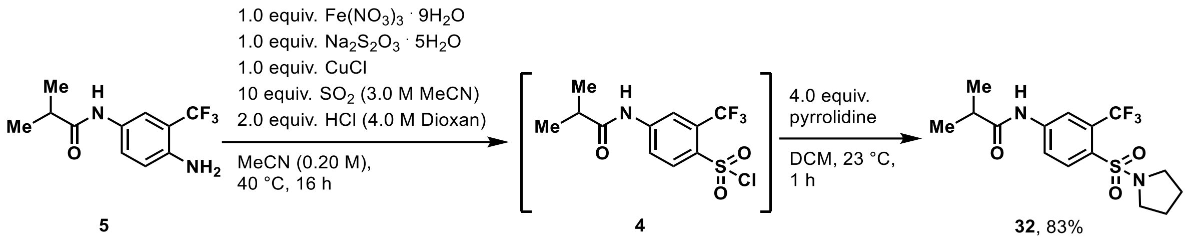

Caution: A blast shield must be used to minimize personal damage in case of an accident. See safety statement on page S8.

Under an ambient atmosphere, to a 20-mL borosilicate vial equipped with a Teflon-coated magnetic stir bar were added reduced Flutamide **5** (123 mg, 0.500 mmol, 1.00 equiv.), $Na_2S_2O_3$ $5H_2O$ (124 mg, 0.500 mmol, 1.00 equiv.) and CuCl (49.5 mg, 0.500 mmol, 1.0 equiv.). Then, acetonitrile (2.5 mL), HCl (4.0 M in 1,4-dioxane, 250 µL, 1.00 mmol, 2.0 equiv.), and SO_2 (3.0 M in MeCN, 1.7 mL, 5.0 mmol, 10 equiv.) were added, the vial was quickly sealed with a septum cap and the mixture stirred for 5 min at 23 °C. Subsequently, iron(III) nitrate nonahydrate (202 mg, 0.500 mmol, 1.00 equiv.) was added to the suspension and the vial was quickly resealed with a septum cap. The mixture was then stirred at 40 °C for 18 h. After cooling to 23 °C, the resulting mixture was concentrated by rotary evaporation under reduced pressure. The residue was diluted with water (5 mL) and extracted with DCM (3 × 5 mL). The organic layers were combined in a 20-mL borosilicate vial and a Teflon-coated magnetic stir bar was added, followed by pyrrolidine (165 µL, 142 mg, 2.00 mmol, 4.00 equiv.). The vessel was sealed with a septum cap and the mixture stirred for 1 h at 23 °C. Subsequently water (5 mL) was added, the aqueous phase decanted and further extracted with DCM (3 × 5 mL). The combined organic layers were dried over $MgSO_4$, filtered, and the solvent evaporated under reduced pressure. The residue was purified by column chromatography on silica with ethyl acetate in pentane (25%, v/v) as eluent, to give the desired sulfonamide **32** as a light-yellow oil (0.12 g, 0.42 mmol, 83%).

R_f = 0.28 (EtOAc in pentane = 40%, v/v (UV)).

NMR Spectroscopy:

^{1}H NMR (500 MHz, $CDCl_3$, 23°C, δ): 8.31 (s, 1H), 8.13–7.96 (m, 3H), 3.44–3.20 (m, 4H), 2.61 (p, *J* = 6.8 Hz, 1H), 2.10–1.75 (m, 4H), 1.24 (d, *J* = 6.8 Hz, 6H).

^{13}C NMR (125 MHz, $CDCl_3$, 23°C, δ): 176.6, 142.4, 132.9, 131.8, 128.81 (q, *J* = 33.3 Hz), 122.3 (q, *J* = 274.3 Hz), 121.6, 119.1 (q, *J* = 6.8 Hz), 47.7, 36.5, 25.6, 19.4.

^{19}F NMR (565 MHz, $CDCl_3$, 23°C, δ): −58.10

HRMS-ESI (m/z) calc'd for $C_{15}H_{19}N_2O_3S_1F_3Na_1$ $[M+Na]^+$, 387.0961; found, 387.0959; deviation: +0.3 ppm.

5-(Pyrrolidin-1-ylsulfonyl)picolinonitrile (33)

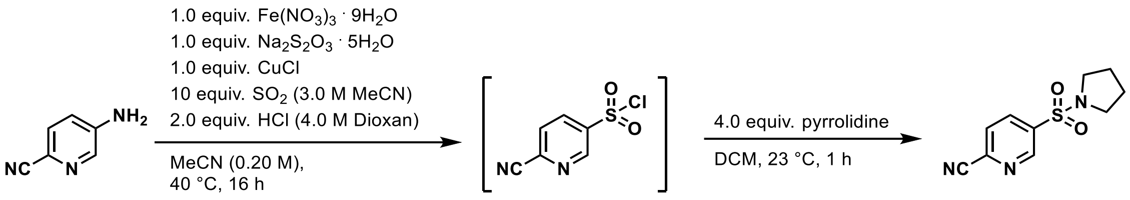

33a **33**, 38%

Caution: A blast shield must be used to minimize personal damage in case of an accident. See safety statement on page S8.

Under an ambient atmosphere, to a 20-mL borosilicate vial equipped with a Teflon-coated magnetic stir bar were added 5-aminopicolinonitrile (59.6 mg, 0.500 mmol, 1.00 equiv.), $Na_2S_2O_3$ $5H_2O$ (124 mg, 0.500 mmol, 1.00 equiv.) and CuCl (49.5 mg, 0.500 mmol, 1.0 equiv.). Then, acetonitrile (2.5 mL), HCl (4.0 M in 1,4-dioxane, 250 µL, 1.00 mmol, 2.0 equiv.), and SO_2 (3.0 M in MeCN, 1.7 mL, 5.0 mmol, 10 equiv.) were added, the vial was quickly sealed with a septum cap and the mixture stirred for 5 min at 23 °C. Subsequently, iron(III) nitrate nonahydrate (202 mg, 0.500 mmol, 1.00 equiv.) was added to the suspension and the vial was quickly resealed with a septum cap. The mixture was then stirred at 40 °C for 18 h. After cooling to 23 °C, the resulting mixture was concentrated by rotary evaporation under reduced pressure. The residue was diluted with water (5 mL) and extracted with DCM (3 × 5 mL). The organic layers were combined in a 20-mL borosilicate vial and a Teflon-coated magnetic stir bar was added, followed by pyrrolidine (165 µL, 142 mg, 2.00 mmol, 4.00 equiv.). The vessel was sealed with a septum cap and the mixture stirred for 1 h at 23 °C. Subsequently water (5 mL) was added, the aqueous phase decanted and further extracted with DCM (3 × 5 mL). The combined organic layers were dried over $MgSO_4$, filtered, and the solvent evaporated under reduced pressure. The residue was purified by column chromatography on silica with ethyl acetate in pentane (25%, v/v) as eluent, to give the desired sulfonamide **35** as an off-white solid (45 mg, 0.19 mmol, 38%).

R*f* = 0.50 (EtOAc in pentane = 40%, v/v (UV)).

NMR Spectroscopy:

> **^{1}H NMR** (500 MHz, DMSO-d_6, 23°C, δ): 9.13 (d, J = 2.3 Hz, 1H), 8.48 (dd, J = 8.2, 2.3 Hz, 1H), 8.31 (d, J = 8.1 Hz, 1H), 3.25–2.51 (m, 4H), 1.70 (q, J = 3.4 Hz, 4H).

> **^{13}C NMR** (125 MHz, DMSO-d_6, 23°C, δ): 149.1, 137.6, 136.5, 136.2, 130.3, 117.1, 48.4, 25.3.

HRMS-EI (m/z) calc'd for $C_{10}H_{11}N_3O_2S_1$ $[M]^+$, 237.0567; found, 237.0565; deviation: +0.6 ppm.

Sulfadoxin-derived sulfonyl chloride 34a

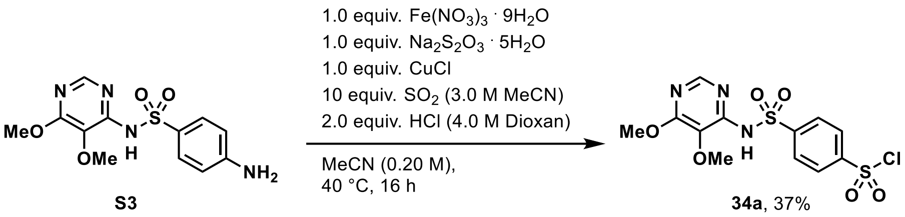

Caution: A blast shield must be used to minimize personal damage in case of an accident. See safety statement on page S8.

Under an ambient atmosphere, to a 20-mL borosilicate vial equipped with a Teflon-coated magnetic stir bar were added Sulfadoxine **S3** (155.2 mg, 0.500 mmol, 1.00 equiv.), $Na_2S_2O_3$ $5H_2O$ (124 mg, 0.500 mmol, 1.00 equiv.) and CuCl (49.5 mg, 0.500 mmol, 1.0 equiv.). Then, acetonitrile (2.5 mL), HCl (4.0 M in 1,4-dioxane, 250 µL, 1.00 mmol, 2.0 equiv.), and SO_2 (3.0 M in MeCN, 1.7 mL, 5.0 mmol, 10 equiv.) were added, the vial was quickly sealed with a septum cap and the mixture stirred for 5 min at 23 °C. Subsequently, iron(III) nitrate nonahydrate (202 mg, 0.500 mmol, 1.00 equiv.) was added to the suspension and the vial was quickly resealed with a septum cap. The mixture was then stirred at 40 °C for 18 h. After cooling to 23 °C, the resulting mixture was concentrated by rotary evaporation under reduced pressure. The residue was diluted with water (5 mL) and extracted with DCM (3 × 5 mL). The combined organic layers were dried over $MgSO_4$, filtered, and the solvent evaporated under reduced pressure. The residue was purified by column chromatography on silica with ethyl acetate in pentane (30%, v/v) as eluent, to give the desired sulfonyl chloride **34** as a colorless solid (73 mg, 0.19 mmol, 37%).

R*f* = 0.25 (EtOAc in pentane = 50%, v/v (UV)).

NMR Spectroscopy:

^{1}H NMR (500 MHz, DMSO-d_6, 23°C, δ): 8.11 (s, 1H), 7.96 (d, *J* = 8.3 Hz, 2H), 7.77 (d, *J* = 8.5 Hz, 2H), 3.90 (s, 3H), 3.70 (s, 3H).

^{13}C NMR (125 MHz, DMSO-d_6, 23°C, δ): 162.2, 152.6, 150.9, 150.7, 141.1, 127.9, 127.7, 126.4, 60.7, 54.6.

HRMS-ESI (m/z) calc'd for $C_{12}H_{11}N_3O_6S_2Cl_1$ $[M-H]^-$, 391.9783; found, 391.9789; deviation: +1.4 ppm.

Aniline Scope for Deaminative Fluorination

Ethyl-4-fluorobenzoate (35)

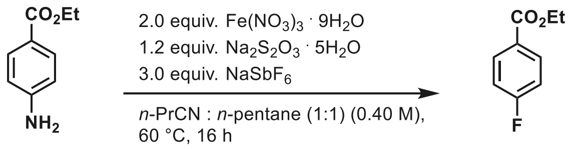

35, 57%

Caution: A blast shield must be used to minimize personal damage in case of an accident. See safety statement on page S8.

Under an ambient atmosphere, to a 4-mL borosilicate vial equipped with a teflon-coated magnetic stir bar were added ethyl-4-aminobenzoate (16.5 mg, 0.100 mmol, 1.00 equiv.), $Na_2S_2O_3$ $5H_2O$ (29.8 mg, 0.120 mmol, 1.20 equiv.), $NaSbF_6$ (77.6 mg, 0.300 mmol, 3.00 equiv.) and iron (III) nitrate nonahydrate (80.8 mg, 0.200 mmol, 2.00 equiv.). Then, butyronitrile (*n*-PrCN) (0.125 mL), *n*-pentane (0.125 mL) (0.4 M overall conc.) were added, the vial was quickly sealed with a septum cap and the reaction mixture stirred at 60 °C for 16 h. After cooling to 23 °C, the resulting mixture was passed through a pad of celite (~1.0 g) by eluting with ethyl acetate (10 ml). Then, the solvent was removed under reduced pressure. The residue was purified by column chromatography on silica with ethyl acetate in pentane (1%, v/v) as eluent, to give the desired fluoroarene **35** as a colorless liquid (9.6 mg, 0.057 mmol, 57%).

R_f = 0.53 (EtOAc in pentane = 5%, v/v (UV)).

NMR Spectroscopy:

^{1}H NMR (500 MHz, $CDCl_3$, 23°C, δ): 8.14 – 7.96 (m, 2H), 7.14 – 7.05 (m, 2H), 4.36 (q, *J* = 7.2 Hz, 2H), 1.38 (t, *J* = 7.1 Hz, 3H).

^{13}C NMR (125 MHz, $CDCl_3$, 23°C, δ): 165.7 (d, *J* = 251 Hz), 165.6, 132.0 (d, *J* = 8.7 Hz), 126.7 (d, *J* = 2.5 Hz), 115.4 (d, *J* = 21.2 Hz), 61.1, 14.3.

^{19}F NMR (471 MHz, $CDCl_3$, 23°C, δ): −106.1 (m).

HRMS-EI (m/z) calc'd for $C_9H_9O_2F_1$ $[M]^+$, 168.0581; found, 168.0581; deviation: −0.2 ppm.

4-Fluoro-3,5-dimethylbenzonitrile (36)

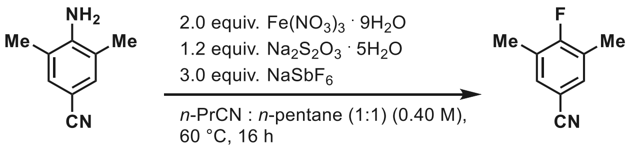

36, 66%

Caution: A blast shield must be used to minimize personal damage in case of an accident. See safety statement on page S8.

Under an ambient atmosphere, to a 4-mL borosilicate vial equipped with a teflon-coated magnetic stir bar were added 4-amino-3,5-dimethylbenzonitrile (73.1 mg, 0.500 mmol, 1.00 equiv.), $Na_2S_2O_3$ $5H_2O$

(149 mg, 0.600 mmol, 1.20 equiv.), $NaSbF_6$ (388 mg, 1.50 mmol, 3.00 equiv.) and iron (III) nitrate nonahydrate (404 mg, 1.00 mmol, 2.00 equiv.). Then, butyronitrile (*n*-PrCN) (0.625 mL), *n*-pentane (0.625 mL) (0.4 M overall conc.) were added, the vial was quickly sealed with a septum cap and the reaction mixture stirred at 60 °C for 16 h. After cooling to 23 °C, the resulting mixture was passed through a pad of celite (~2.5 g) by eluting with ethyl acetate (50 ml). Then, the solvent was removed under reduced pressure. The residue was purified by column chromatography on silica with ethyl acetate in pentane (1%, v/v) as eluent, to give the desired fluoroarene **36** as a colorless solid (49 mg, 0.33 mmol, 66%).

$\mathbf{R_f}$ = 0.43 (EtOAc in pentane = 5%, v/v (UV)).

NMR Spectroscopy:

^{1}H NMR (500 MHz, $CDCl_3$, 23°C, δ): 7.30 (d, *J* = 6.6 Hz, 1H), 2.26 (d, *J* = 2.6 Hz, 3H).

^{13}C NMR (125 MHz, $CDCl_3$, 23°C, δ): 162.3 (d, *J* = 252 Hz), 132.9 (d, *J* = 7.5 Hz), 126.3 (d, *J* = 18.7 Hz), 118.5, 107.50 (d, *J* = 3.7 Hz), 14.4 (d, *J* = 3.7 Hz).

^{19}F NMR (471 MHz, $CDCl_3$, 23°C, δ): −111.5 (m).

HRMS-EI (m/z) calc'd for $C_9H_8N_1F_1$ $[M]^+$, 149.0635; found, 149.0636; deviation: −0.2 ppm.

(4-Fluorophenyl)(phenyl)methanone (37)

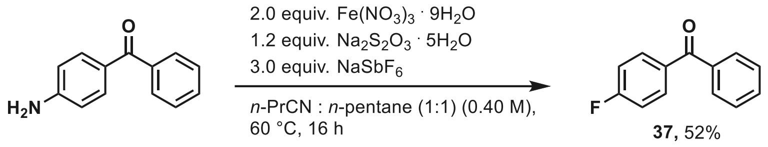

Caution: A blast shield must be used to minimize personal damage in case of an accident. See safety statement on page S8.

Under an ambient atmosphere, to a 4-mL borosilicate vial equipped with a teflon-coated magnetic stir bar were added 4-aminobenzophenone (19.7 mg, 0.100 mmol, 1.00 equiv.), $Na_2S_2O_3$ $5H_2O$ (29.8 mg, 0.120 mmol, 1.20 equiv.), $NaSbF_6$ (77.6 mg, 0.300 mmol, 3.00 equiv.) and iron (III) nitrate nonahydrate (80.8 mg, 0.200 mmol, 2.00 equiv.). Then, butyronitrile (*n*-PrCN) (0.125 mL), *n*-pentane (0.125 mL) (0.4 M overall conc.) were added, the vial was quickly sealed with a septum cap and the reaction mixture stirred at 60 °C for 16 h. After cooling to 23 °C, the resulting mixture was passed through a pad of celite (~1.0 g) by eluting with ethyl acetate (10 ml). Then, the solvent was removed under reduced pressure. The residue was purified by column chromatography on silica with ethyl acetate in pentane (1%, v/v) as eluent, to give the desired fluoroarene **37** as a colorless liquid (10.2 mg, 0.052 mmol, 52%).

$\mathbf{R_f}$ = 0.35 (EtOAc in pentane = 5%, v/v (UV)).

NMR Spectroscopy:

^{1}H NMR (500 MHz, $CDCl_3$, 23°C, δ): 7.90 – 7.84 (m, 2H), 7.81 – 7.77 (m, 2H), 7.64 – 7.60 (m, 1H), 7.51 (dd, *J* = 8.4, 7.0 Hz, 2H), 7.21 – 7.15 (m, 2H).

^{13}C NMR (125 MHz, $CDCl_3$, 23°C, δ): 195.3, 166.4 (d, *J* = 252 Hz), 137.5, 133.8 (d, *J* = 3.7 Hz), 132.7 (d, *J* = 10 Hz), 132.5, 129.9, 128.4, 115.5 (d, *J* = 22.5 Hz).

^{19}F NMR (471 MHz, $CDCl_3$, 23°C, δ): −106.0 (m).

HRMS-EI (m/z) calc'd for $C_{13}H_9O_1F_1$ $[M]^+$, 200.0632; found, 200.0635; deviation: −1.4 ppm.

1,1,1,3,3,3-Hexafluoro-2-(4-fluorophenyl)propan-2-ol (38)

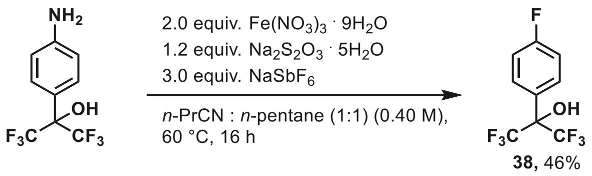

Caution: A blast shield must be used to minimize personal damage in case of an accident. See safety statement on page S8.

Under an ambient atmosphere, to a 4-mL borosilicate vial equipped with a teflon-coated magnetic stir bar were added 2-(4-aminophenyl)-1,1,1,3,3,3-hexafluor-2-propanol (130 mg, 0.500 mmol, 1.00 equiv.), $Na_2S_2O_3$ $5H_2O$ (149 mg, 0.600 mmol, 1.20 equiv.), $NaSbF_6$ (388 mg, 1.50 mmol, 3.00 equiv.) and iron (III) nitrate nonahydrate (404 mg, 1.00 mmol, 2.00 equiv.). Then, butyronitrile (*n*-PrCN) (0.625 mL), *n*-pentane (0.625 mL) (0.4 M overall conc.) were added, the vial was quickly sealed with a septum cap and the reaction mixture stirred at 60 °C for 16 h. After cooling to 23 °C, the resulting mixture was passed through a pad of celite (~2.5 g) by eluting with CH_2Cl_2 (50 ml). Then, the solvent was removed under reduced pressure. The yield was determined by both ^{1}H NMR spectroscopy at 500 MHz dissolving the residue of the reaction mixture in 0.5 mL of $CDCl_3$, and using CH_2Br_2 as internal standard (34.9 µL, 86.9 mg, 0.500 mmol, 1.00 equiv.) for ^{1}H NMR. The integration of the CH_2Br_2 signal at 4.94 ppm (s, 2H) was compared to the signal of **38** at 7.13 ppm (t, 2H).

R_f = 0.44 (EtOAc in pentane = 10%, v/v (UV)).

HRMS-EI(m/z) calc'd for $C_9H_5O_1F_7$ $[M]^+$, 262.0223; found, 269.0223; deviation: −0.1 ppm.

Note: Due to the volatility of the product the compound was not isolated. Signal at 5.30 ppm corresponds to CH_2Cl_2; signals at 2.34 ppm, 1.68 ppm and 1.08 ppm correspond to *n*-PrCN.

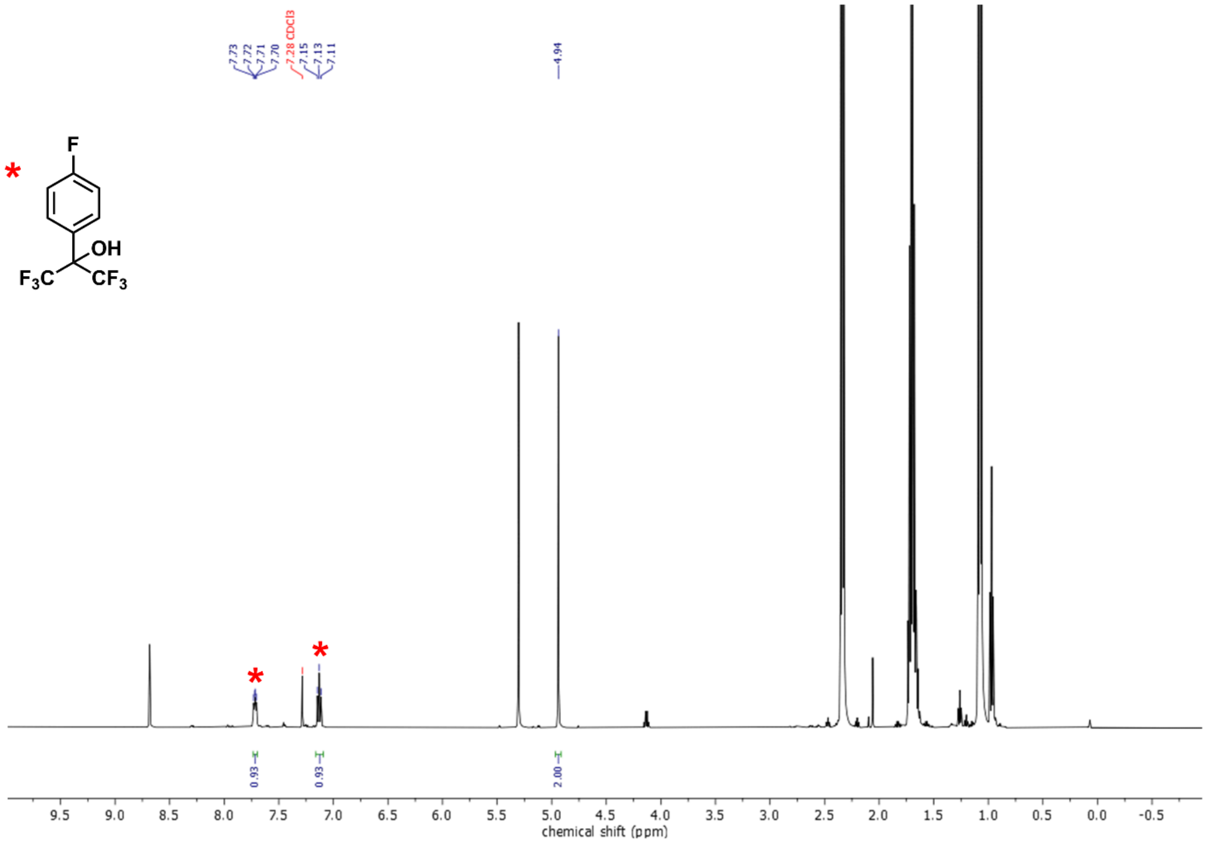

Fig S1. ^{1}H NMR spectra at 500 MHz NMR field and 298 K recorded in $CDCl_3$ solution after the reaction using CH_2Br_2 as internal standard (34.9 µL, 86.9 mg, 0.500 mmol, 1.00 equiv.). The integration of the CH_2Br_2 signal at 4.94 ppm (s, 2H) was compared to the signal of **38** at 7.13 ppm (t, 2H). Red stars: fluoroarene **38**.

3-Fluoro-2,6-dimethylpyridine (39)

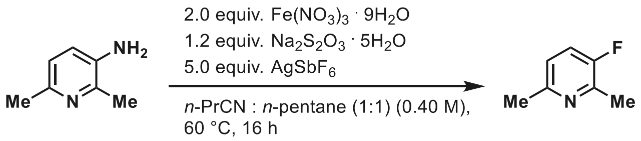

39, 51%

Caution: A blast shield must be used to minimize personal damage in case of an accident. See safety statement on page S8.

Under an ambient atmosphere, to a 4-mL borosilicate vial equipped with a teflon-coated magnetic stir bar were added 3-amino-2,6-dimethylpyridine (61.1 mg, 0.500 mmol, 1.00 equiv.), $Na_2S_2O_3$ $5H_2O$ (149 mg, 0.600 mmol, 1.20 equiv.), $AgSbF_6$ (859 mg, 2.50 mmol, 5.00 equiv.) and iron (III) nitrate nonahydrate (404 mg, 1.00 mmol, 2.00 equiv.) respectively. Then, butyronitrile (*n*-PrCN) (0.625 mL), *n*-pentane (0.625 mL) (0.4 M overall conc.) were added, the vial was quickly sealed with a septum cap and the reaction mixture stirred at 60 °C for 16 h. After cooling to 23 °C, the resulting mixture was passed through a pad of celite (~2.5 g) by eluting with CH_2Cl_2 (50 ml). Then, the solvent was removed under reduced pressure. The yield was determined by both ^{1}H NMR spectroscopy at 500 MHz dissolving the residue of the reaction mixture in 0.5 mL of $CDCl_3$, and using CH_2Br_2 as internal

standard (34.9 µL, 86.9 mg, 0.50 mmol, 1.0 equiv.) for ^1H NMR. The integration of the CH_2Br_2 signal at 4.93 ppm (s, 2H) was compared to the signal of **39** at 7.65 (dd, J = 8.8, 4.5 Hz, 1H).

R*f* = 0.62 (CH_2Cl_2 in MeOH = 10%, v/v (UV)).

HRMS-EI(m/z) calc'd for $C_7H_8N_1F_1$ $[M]^+$, 125.0635; found, 125.0636; deviation: −1.1 ppm.

Note: Due to the volatility of the product the compound was not isolated. Signal at 5.29 ppm corresponds to CH_2Cl_2; signals at 2.42 ppm, 1.71 ppm and 1.06 ppm correspond to *n*-PrCN.

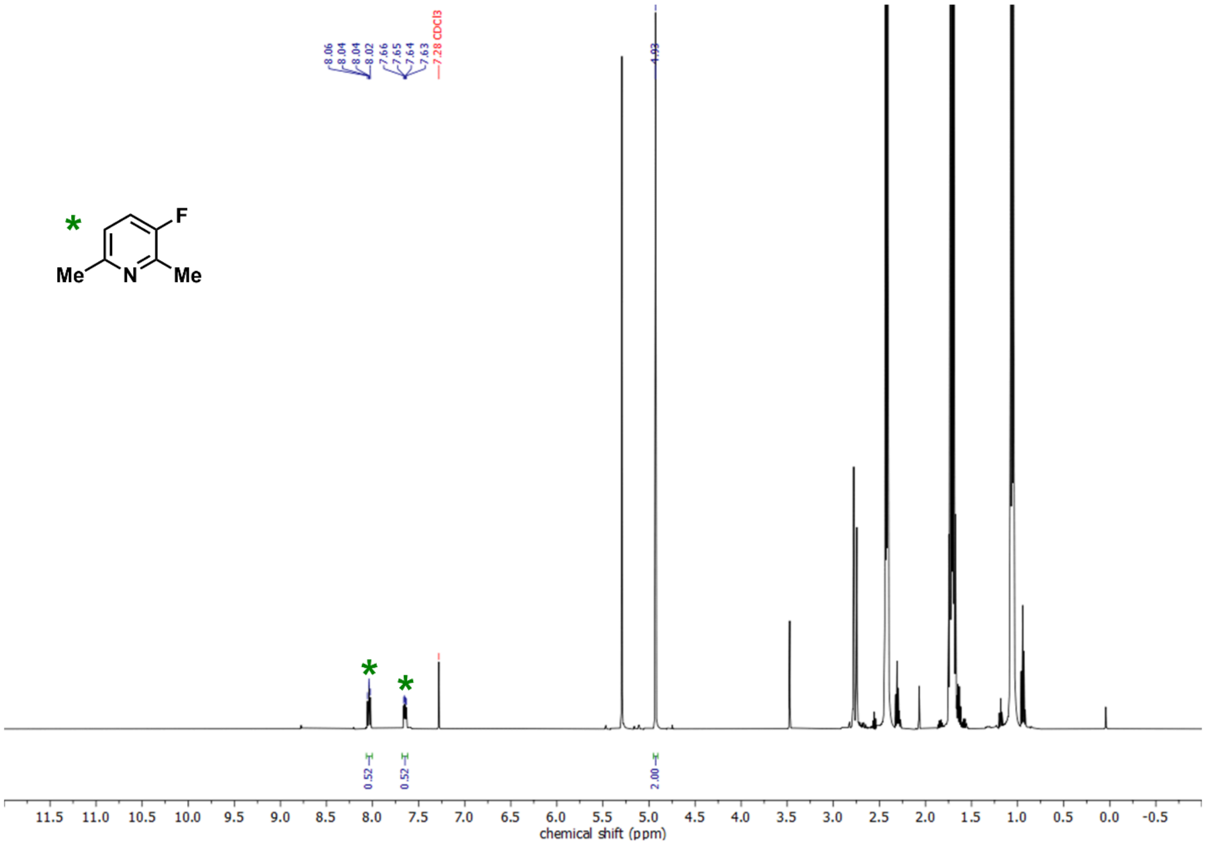

Fig S2. ^1H NMR spectra at 500 MHz NMR field and 298 K recorded in $CDCl_3$ solution after the reaction using CH_2Br_2 as internal standard (34.9 µL, 86.9 mg, 0.500 mmol, 1.00 equiv.). The integration of the CH_2Br_2 signal at 4.93 ppm (s, 2H) was compared to the signal of **39** at 7.65 (dd, J = 8.8, 4.5 Hz, 1H). Green stars: fluoroarene **39**.

4'-Chloro-2-fluoro-1,1'-biphenyl (40)

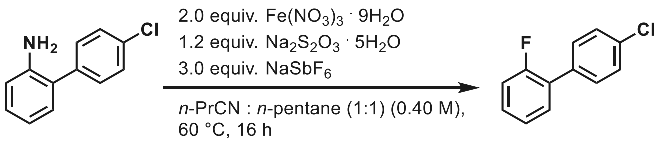

40, 61%

Caution: A blast shield must be used to minimize personal damage in case of an accident. See safety statement on page S8.

Under an ambient atmosphere, to a 4-mL borosilicate vial equipped with a teflon-coated magnetic stir

bar were added 4'-chloro-(1,1'-biphenyl)-2-amine (102 mg, 0.500 mmol, 1.00 equiv.), $Na_2S_2O_3$ $5H_2O$ (149 mg, 0.600 mmol, 1.20 equiv.), $NaSbF_6$ (388 mg, 1.50 mmol, 3.00 equiv.) and iron (III) nitrate nonahydrate (404 mg, 1.00 mmol, 2.00 equiv.). Then, butyronitrile (*n*-PrCN) (0.625 mL), *n*-pentane (0.625 mL) (0.4 M overall conc.) were added, the vial was quickly sealed with a septum cap and the reaction mixture stirred at 60 °C for 16 h. After cooling to 23 °C, the resulting mixture was passed through a pad of celite (~2.5 g) by eluting with ethyl acetate (50 ml). Then, the solvent was removed under reduced pressure. The residue was purified by column chromatography on silica with pentane as eluent, to give the desired fluoroarene **40** as a colorless liquid (63 mg, 0.305 mmol, 61%).

R_f = 0.67 (in pentane (UV)).

NMR Spectroscopy:

^{1}H NMR (600 MHz, $CDCl_3$, 23°C, δ): 7.52 – 7.50 (m, 2H), 7.47 – 7.41 (m, 2H), 7.43 (td, J = 7.9, 1.8 Hz, 1H), 7.35 (dddd, J = 8.2, 7.4, 5.0, 1.8 Hz, 1H), 7.23 (td, J = 7.5, 1.2 Hz, 1H), 7.18 (ddd, J = 10.8, 8.2, 1.2 Hz, 1H).

^{13}C NMR (151 MHz, $CDCl_3$, 23°C, δ): 159.8 (d, J = 248 Hz), 134.3 (d, J = 0.8 Hz), 133.9, 130.6 (d, J = 3.3 Hz), 130.4 (d, J = 3.1 Hz), 129.5 (d, J = 8.3 Hz), 128.8, 128.0 (d, J = 13.3 Hz), 124.6 (d, J = 3.7 Hz), 116.3 (d, J = 22.7 Hz).

^{19}F NMR (565 MHz, $CDCl_3$, 23°C, δ): −117.8 (m).

HRMS-EI (m/z) calc'd for $C_{12}H_8Cl_1F_1$ $[M]^+$, 206.0293; found, 206.0293; deviation: −0.1 ppm.

Lenalidomide-derived fluoroarene 41

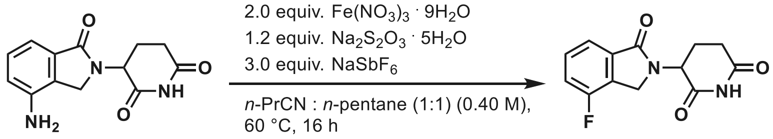

41, 40%

Caution: A blast shield must be used to minimize personal damage in case of an accident. See safety statement on page S8.

Under an ambient atmosphere, to a 4-mL borosilicate vial equipped with a teflon-coated magnetic stir bar were added Lenalidomide (130 mg, 0.500 mmol, 1.00 equiv.), $Na_2S_2O_3$ $5H_2O$ (149 mg, 0.600 mmol, 1.20 equiv.), $NaSbF_6$ (388 mg, 1.50 mmol, 3.00 equiv.) and iron (III) nitrate nonahydrate (404 mg, 1.00 mmol, 2.00 equiv.). Then, butyronitrile (*n*-PrCN) (1.25 mL) (c = 0.4 M) were added, the vial was quickly sealed with a septum cap and the reaction mixture stirred at 60 °C for 16 h. After cooling to 23 °C, the resulting mixture was passed through a pad of celite (~2.5 g) by eluting with ethyl acetate (50 ml). Then, the solvent was removed under reduced pressure. The residue was purified by column chromatography on silica with ethyl acetate in pentane (60%, v/v) as eluent, to give the desired fluoroarene **41** as a colorless liquid (52 mg, 0.198 mmol, 40%).

R_f = 0.37 (EtOAc in pentane = 80%, v/v (UV)).

NMR Spectroscopy:

^{1}H NMR (500 MHz, DMSO-d_6, 23°C, δ): 11.01 (s, 1H), 7.64 – 7.55 (m, 2H), 7.50 (ddd, *J* = 9.0, 7.3, 1.8 Hz, 1H), 5.13 (dd, *J* = 13.3, 5.1 Hz, 1H), 4.58 (d, *J* = 17.3 Hz, 1H), 4.40 (d, *J* = 17.4 Hz, 1H), 2.92 (ddd, *J* = 17.4, 13.6, 5.4 Hz, 1H), 2.64 – 2.57 (m, 1H), 2.43 (td, J = 13.3, 4.5 Hz, 1H), 2.01 (dtd, *J* = 13.5, 5.7, 2.4 Hz, 1H).

^{13}C NMR (151 MHz, DMSO-d_6, 23°C, δ): 172.8, 170.8, 167.0 (d, *J* = 2.3 Hz), 157.9, 156.2, 134.9 (d, *J* = 4.8 Hz), 130.7 (d, *J* = 6.5 Hz), 127.9, 127.8, 119.5 (d, *J* = 3.3 Hz), 118.5, 118.4, 51.8, 44.2, 31.2, 22.3.

^{19}F NMR (565 MHz, DMSO-d_6, 23°C, δ): − 120.1 (dd, *J* = 9.2, 4.1 Hz).

HRMS-ESI (m/z) calc'd for $C_{13}H_{11}F_1N_2Na_1O_3$ $[M+Na]^+$, 285.0646; found, 285.0650; deviation: −1.7 ppm.

Aniline Scope for Sulfonic Acid Synthesis by Deaminative Sulfonylation

Note: Due to the acidity of the formed aryl sulfonic acids, it cannot be excluded that minor contaminations of the respective sulfonate salts are formed during the purification process.

4-Benzoylbenzenesulfonic acid (42)

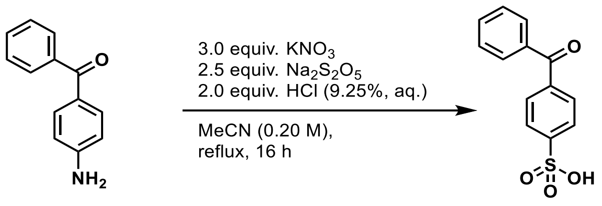

42, 53%

Caution: A blast shield must be used to minimize personal damage in case of an accident. See safety statement on page S8.

Under an ambient atmosphere, to a 4-mL borosilicate vial equipped with a Teflon-coated magnetic stir bar were added 4-aminobenzophenone (39.4 mg, 0.200 mmol, 1.00 equiv.), KNO_3 (60.7 mg, 0.600 mmol, 2.00 equiv.), and $Na_2S_2O_5$ (95.1 mg, 0.500 mmol, 2.50 equiv.). Then, acetonitrile (1.0 mL) and HCl (9.25%, aqueous, 131 µL, 4.00 mmol, 2.00 equiv.) were added, the vial was quickly sealed with a septum cap and the mixture stirred at 85 °C (oil bath temperature) for 18 h. After cooling to 23 °C, the resulting mixture was concentrated by rotary evaporation under reduced pressure. The residue was purified by column chromatography on silica with MeOH in DCM (0%–20% over 30 column volume followed by 20% for 20 column volume) as eluent to give the desired sulfonic acid **42** as a colorless solid (28 mg, 0.11 mmol, 53%).

$\mathbf{R}_f$ = 0.41 (MeOH in DCM = 20%, v/v (UV)).

NMR Spectroscopy:

^{1}H NMR (600 MHz, DMSO-d_6, 23°C, δ): 7.81–7.76 (m, 2H), 7.76–7.65 (m, 5H), 7.61–7.52 (m, 2H).

^{13}C NMR (125 MHz, DMSO-d_6, 23°C, δ): 195.9, 152.3, 137.5, 137.3, 133.2, 130.1, 129.9, 129.1, 126.2.

HRMS-ESI (m/z) calc’d for $C_{13}H_9O_4S_1$ $[M-H]^-$, 261.0227; found, 261.0228; deviation: −0.3 ppm.

4-Phenoxybenzenesulfonic acid (43)

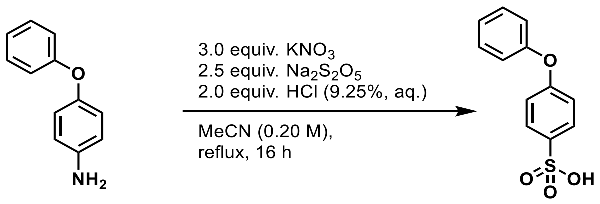

43, 58%

Caution: A blast shield must be used to minimize personal damage in case of an accident. See safety statement on page S8.

Under an ambient atmosphere, to a 4-mL borosilicate vial equipped with a Teflon-coated magnetic stir bar were added 4-phenoxyaniline (37.1 mg, 0.200 mmol, 1.00 equiv.), KNO_3 (60.7 mg, 0.600 mmol, 2.00 equiv.), and $Na_2S_2O_5$ (95.1 mg, 0.500 mmol, 2.50 equiv.). Then, acetonitrile (1.0 mL) and HCl (9.25%, aqueous, 131 µL, 4.00 mmol, 2.00 equiv.) were added, the vial was quickly sealed with a septum cap and the mixture stirred at 85 °C (oil bath temperature) for 18 h. After cooling to 23 °C, the resulting mixture was concentrated by rotary evaporation under reduced pressure. The residue was purified by column chromatography on silica with MeOH in DCM (5%, v/v) as eluent to give the desired sulfonic acid **43** as a colorless solid (29 mg, 0.12 mmol, 58%).

R_f = 0.56 (MeOH in DCM = 20%, v/v (UV)).

NMR Spectroscopy:

^{1}H NMR (600 MHz, DMSO-d_6, 23°C, δ): 7.70–7.54 (m, 2H), 7.40 (dd, J = 8.6, 7.4 Hz, 2H), 7.15 (t, J = 7.4 Hz, 1H), 7.02 (dd, J = 8.7, 1.1 Hz, 2H), 6.93 (d, J = 8.6 Hz, 2H).

^{13}C NMR (125 MHz, DMSO-d_6, 23°C, δ): 156.7, 156.4, 143.6, 130.1, 127.5, 123.6, 118.8, 117.4.

HRMS-ESI (m/z) calc'd for $C_{12}H_9O_4S_1$ $[M-H]^-$, 249.0227; found, 249.0229; deviation: −0.6 ppm.

4-Cyanobenzenesulfonic acid (44)

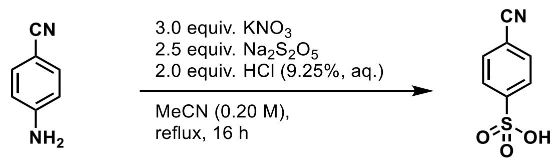

44, 70%

Caution: A blast shield must be used to minimize personal damage in case of an accident. See safety statement on page S8.

Under an ambient atmosphere, to a 4-mL borosilicate vial equipped with a Teflon-coated magnetic stir bar were added 4-aminobenzonitrile (23.6 mg, 0.200 mmol, 1.00 equiv.), KNO_3 (60.7 mg, 0.600 mmol, 2.00 equiv.), and $Na_2S_2O_5$ (95.1 mg, 0.500 mmol, 2.50 equiv.). Then, acetonitrile (1.0 mL) and HCl (9.25%, aqueous, 131 µL, 4.00 mmol, 2.00 equiv.) were added, the vial was quickly sealed with a septum cap and the mixture stirred at 85 °C (oil bath temperature) for 18 h. After cooling to 23 °C, the resulting mixture was concentrated by rotary evaporation under reduced pressure. The residue was purified first by column chromatography on silica with MeOH in DCM (gradient, 0%–20% over 50 column volumes) as eluent and then by reversed phase column chromatography on a C18 column with MeOH in water (gradient, 0%–100% over 30 column volumes) as eluent to give the desired sulfonic acid **44** as a colorless solid (26 mg, 0.14 mmol, 70%).

R_f = 0.27 (MeOH in DCM = 20%, v/v (UV)).

NMR Spectroscopy:

^{1}H NMR (600 MHz, DMSO-d_6, 23°C, δ): 7.96–7.78 (m, 2H), 7.78–7.67 (m, 2H).

^{13}C NMR (125 MHz, DMSO-d_6, 23°C, δ): 152.6, 132.1, 126.4, 118.7, 111.1.

HRMS-ESI (m/z) calc'd for $C_7H_4N_1O_4S_1$ $[M-H]^-$, 181.9917; found, 181.9918; deviation: −0.5 ppm.

3-Chloro-4-methylbenzenesulfonic acid (45)

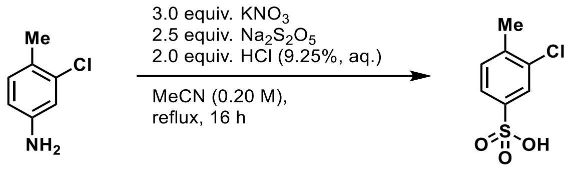

45, 66%

Caution: A blast shield must be used to minimize personal damage in case of an accident. See safety statement on page S8.

Under an ambient atmosphere, to a 4-mL borosilicate vial equipped with a Teflon-coated magnetic stir bar were added 4-chloro-3-methylaniline (28.3 mg, 0.200 mmol, 1.00 equiv.), KNO_3 (60.7 mg, 0.600 mmol, 2.00 equiv.), and $Na_2S_2O_5$ (95.1 mg, 0.500 mmol, 2.50 equiv.). Then, acetonitrile (1.0 mL) and HCl (9.25%, aqueous, 131 µL, 4.00 mmol, 2.00 equiv.) were added, the vial was quickly sealed with a septum cap and the mixture stirred at 85 °C (oil bath temperature) for 18 h. After cooling to 23 °C, the resulting mixture was concentrated by rotary evaporation under reduced pressure. The residue was purified by column chromatography on silica with MeOH in DCM (0%–20% over 30 column volume followed by 20% for 20 column volume) as eluent to give the desired sulfonic acid **45** as a yellow oil (27 mg, 0.13 mmol, 66%).

R_f = 0.67 (MeOH in DCM = 20%, v/v (UV)).

NMR Spectroscopy:

^{1}H NMR (600 MHz, CD_3OD, 23°C, δ): 7.74 (s, 1H), 7.61 (d, *J* = 7.7 Hz, 1H), 7.41 (d, *J* = 6.6 Hz, 1H), 2.40 (s, 3H).

^{13}C NMR (125 MHz, CD_3OD, 23°C, δ): 143.2, 135.9, 135.9, 128.5, 128.1, 124.6, 18.6.

HRMS-ESI (m/z) calc'd for $C_7H_6O_3S_1Cl_1$ $[M-H]^-$, 204.9732; found, 204.9732; deviation: −0.2 ppm.

4-Fluorobenzenesulfonic acid (46)

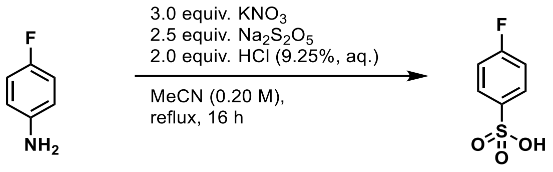

46, 71%

Caution: A blast shield must be used to minimize personal damage in case of an accident. See safety statement on page S8.

Under an ambient atmosphere, to a 4-mL borosilicate vial equipped with a Teflon-coated magnetic stir bar were added 4-fluoroaniline (18.9 µL, 22.2 mg, 0.200 mmol, 1.00 equiv.), KNO_3 (60.7 mg, 0.600 mmol, 2.00 equiv.), and $Na_2S_2O_5$ (95.1 mg, 0.500 mmol, 2.50 equiv.). Then, acetonitrile (1.0 mL) and HCl (9.25%, aqueous, 131 µL, 4.00 mmol, 2.00 equiv.) were added, the vial was quickly sealed with a septum cap and the mixture stirred at 85 °C (oil bath temperature) for 18 h. After cooling to 23 °C, the resulting mixture was concentrated by rotary evaporation under reduced pressure. The residue was purified by column chromatography on silica with MeOH in DCM (0%–20% over 50 column volume followed by 20% for 20 column volume) as eluent, followed by trituration with pentane (10 mL) to give the desired sulfonic acid **46** as a yellow oil (25 mg, 0.14 mmol, 71%).

R_f = 0.58 (MeOH in DCM = 20%, v/v (UV)).

NMR Spectroscopy:

^{1}H NMR (600 MHz, CD_3OD, 23°C, δ): 7.84 (t, *J* = 6.6 Hz, 2H), 7.15 (t, *J* = 7.8 Hz, 2H).

^{13}C NMR (125 MHz, CD_3OD, 23°C, δ): 165.1 (d, *J* = 248.5 Hz), 142.8, 132.0 (d, *J* = 9.8 Hz), 129.6, 117.8 (d, *J* = 23.2 Hz), 116.1 (d, *J* = 22.2 Hz).

HRMS-ESI (m/z) calc'd for $C_6H_4O_3S_1F_1$ $[M-H]^-$, 174.9871; found, 174.9873; deviation: −1.1 ppm.

Aminoglutethimide-derived sulfonic acid *rac*-47

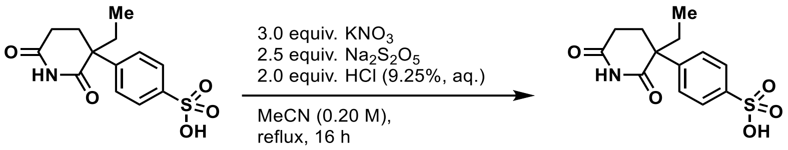

rac-**S4** *rac*-**47**, 56%

Caution: A blast shield must be used to minimize personal damage in case of an accident. See safety statement on page S8.

Under an ambient atmosphere, to a 4-mL borosilicate vial equipped with a Teflon-coated magnetic stir bar were added racemic Aminoglutethimide *rac*-**S4** (46.5 mg, 0.200 mmol, 1.00 equiv.), KNO_3 (60.7 mg, 0.600 mmol, 2.00 equiv.), and $Na_2S_2O_5$ (95.1 mg, 0.500 mmol, 2.50 equiv.). Then, acetonitrile (1.0 mL) and HCl (9.25%, aqueous, 131 µL, 4.00 mmol, 2.00 equiv.) were added, the vial was quickly sealed with a septum cap and the mixture stirred at 85 °C (oil bath temperature) for 18 h. After cooling to 23 °C, the resulting mixture was concentrated by rotary evaporation under reduced pressure. The residue was purified by reversed phase HPLC on a 150 mm Eclipse Plus C18 (21.2 mm *5 µm) with solvent A: MeCN and Solvent B: H_2O + 0.1% TFA (gradient: 10%–20% solvent A over 5 min, to 70% over 3 min, product elution at 4 min) to give the desired sulfonic acid *rac*-**47** as a colorless solid (33 mg, 0.11 mmol, 56%).

R_f = 0.34 (MeOH in DCM = 20%, v/v (UV)).

NMR Spectroscopy:

> **^{1}H NMR** (500 MHz, CD_3OD, 23°C, δ): 7.86 (d, *J* = 8.6 Hz, 2H), 7.45 (d, *J* = 8.5 Hz, 2H), 3.37 (s, 1H), 2.84–2.40 (m, 2H), 2.44–2.20 (m, 2H), 1.99 (ddq, *J* = 46.5, 14.6, 7.4 Hz, 2H), 0.89 (t, *J* = 7.4 Hz, 3H).

> **^{13}C NMR** (125 MHz, CD_3OD, 23°C, δ): 177.3, 175.0, 145.3, 143.6, 127.7, 127.6, 52.3, 33.7, 30.2, 28.1, 9.3.

HRMS-ESI (m/z) calc'd for $C_{13}H_{14}N_1O_5S_1$ $[M-H]^-$, 296.0598; found, 296.0601; deviation: −0.9 ppm.

2-Methyl-3-nitrobenzenesulfonic acid (48)

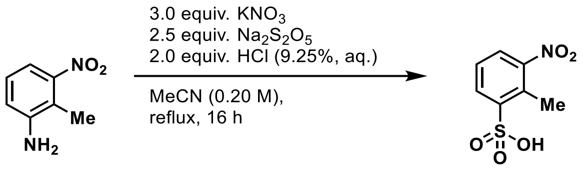

48, 81%

Caution: A blast shield must be used to minimize personal damage in case of an accident. See safety statement on page S8.

Under an ambient atmosphere, to a 4-mL borosilicate vial equipped with a Teflon-coated magnetic stir bar were added 2-methyl-3-nitroaniline (30.4 mg, 0.200 mmol, 1.00 equiv.), KNO_3 (60.7 mg, 0.600 mmol, 2.00 equiv.), and $Na_2S_2O_5$ (95.1 mg, 0.500 mmol, 2.50 equiv.). Then, acetonitrile (1.0 mL) and HCl (9.25%, aqueous, 131 µL, 4.00 mmol, 2.00 equiv.) were added, the vial was quickly sealed with a septum cap and the mixture stirred at 85 °C (oil bath temperature) for 18 h. After cooling to 23 °C, the resulting mixture was concentrated by rotary evaporation under reduced pressure. The residue was purified by column chromatography on silica with MeOH in DCM (0%–20% over 50 column volume followed by 20% for 20 column volume) as eluent, followed by trituration with pentane (10 mL) to give the desired sulfonic acid **48** as a yellow solid (35 mg, 0.16 mmol, 81%).

R_f = 0.71 (MeOH in DCM = 20%, v/v (UV)).

NMR Spectroscopy:

^{1}H NMR (600 MHz, CD_3OD-d_4, 23°C, δ): 8.21 (d, J = 7.4 Hz, 1H), 7.78 (d, J = 7.7 Hz, 1H), 7.43 (s, 1H), 2.72 (s, 3H).

^{13}C NMR (125 MHz, CD_3OD, 23°C, δ): 153.6, 147.4, 131.7, 131.3, 127.4, 126.2, 15.7.

HRMS-ESI (m/z) calc'd for $C_7H_6O_5N_1S_1$ $[M-H]^-$, 215.9972; found, 215.9972; deviation: +0.1 ppm.

Procainamide-derived sulfonic acid 49

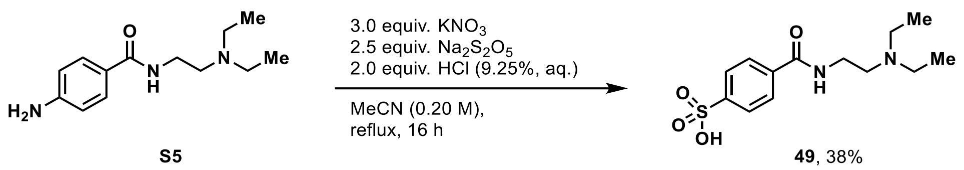

Caution: A blast shield must be used to minimize personal damage in case of an accident. See safety statement on page S8.

Under an ambient atmosphere, to a 4-mL borosilicate vial equipped with a Teflon-coated magnetic stir bar were added Procainamide-hydrochloride **S5** (54.4 mg, 0.200 mmol, 1.00 equiv.), KNO_3 (60.7 mg, 0.600 mmol, 2.00 equiv.), and $Na_2S_2O_5$ (95.1 mg, 0.500 mmol, 2.50 equiv.). Then, acetonitrile (1.0 mL) and HCl (9.25%, aqueous, 131 µL, 4.00 mmol, 2.00 equiv.) were added, the vial was quickly sealed with a septum cap and the mixture stirred at 85 °C (oil bath temperature) for 18 h. After cooling to 23 °C, the resulting mixture was concentrated by rotary evaporation under reduced pressure. The residue was purified by reversed phase HPLC on a 150 mm Eclipse Plus C18 (21.2 mm*5 µm) with solvent A: MeCN and solvent B: 20 mM NH_4HCO_3 buffer pH = 9 (gradient: 8%–8% solvent A over 7 min, to 50% over 0.5 min, product elution at 4 min) to give the desired sulfonic acid **49** as a colorless solid (23 mg, 77 µmol, 38%).

R_f = 0.30 (MeOH in DCM = 50%, v/v (UV)).

NMR Spectroscopy:

^{1}H NMR (500 MHz, D_2O, 23°C, δ): 7.89 (q, J = 0.6 Hz, 4H), 3.80 (t, J = 6.2 Hz, 2H), 3.44 (t, J = 6.3 Hz, 2H), 3.40–3.02 (m, 4H), 1.33 (td, J = 7.4, 0.6 Hz, 6H).

^{13}C NMR (125 MHz, D_2O, 23°C, δ): 170.4, 145.8, 135.6, 128.0, 126.0, 50.8, 48.0, 35.1, 8.2.

HRMS-ESI (m/z) calc'd for $C_{13}H_{19}N_2O_4S_1$ $[M-H]^-$, 299.1071; found, 299.1072; deviation: −0.4 ppm.

Sulfapyridine-derived sulfonic acid 50

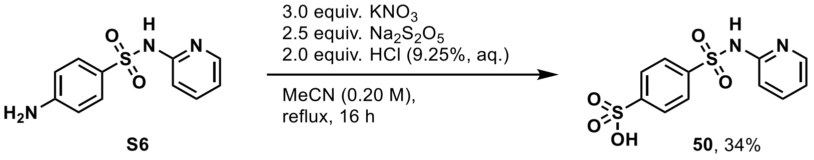

Caution: A blast shield must be used to minimize personal damage in case of an accident. See safety statement on page S8.

Under an ambient atmosphere, to a 4-mL borosilicate vial equipped with a Teflon-coated magnetic stir bar were added Sulfapyridine **S6** (49.9 mg, 0.200 mmol, 1.00 equiv.), KNO_3 (60.7 mg, 0.600 mmol, 2.00 equiv.), and $Na_2S_2O_5$ (95.1 mg, 0.500 mmol, 2.50 equiv.). Then, acetonitrile (1.0 mL) and HCl (9.25%, aqueous, 131 µL, 4.00 mmol, 2.00 equiv.) were added, the vial was quickly sealed with a septum cap and the mixture stirred at 85 °C (oil bath temperature) for 18 h. After cooling to 23 °C, the resulting mixture was concentrated by rotary evaporation under reduced pressure. The residue was purified by reversed phase column chromatography on C18 with MeOH in H_2O + 0.1% formic acid (gradient, 0%–100% over 100 column volumes) as eluent to give the desired sulfonic acid **50** as a colorless solid (21 mg, 68 µmol, 34%).

R_f = 0.27 (MeOH in DCM = 30%, v/v (UV)).

NMR Spectroscopy:

^{1}H NMR (500 MHz, DMSO-d_6, 23°C, δ): 8.00 (dd, J = 5.6, 1.9 Hz, 1H), 7.83 (d, J = 8.4 Hz, 2H), 7.78–7.63 (m, 3H), 7.16 (d, J = 8.7 Hz, 1H), 6.87 (t, J = 6.4 Hz, 1H).

^{13}C NMR (125 MHz, DMSO-d_6, 23°C, δ): 153.7, 151.9, 143.1, 142.4, 141.4, 126.8, 126.5, 115.8, 114.6.

HRMS-ESI (m/z) calc'd for $C_{11}H_9N_2O_5S_2$ $[M-H]^-$, 312.9958; found, 312.9962; deviation: −1.2 ppm.

Mechanistic Experiments

NO_2 detection with gas phase IR spectroscopy

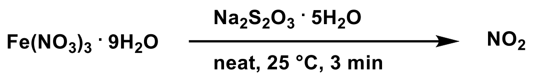

Under an ambient atmosphere, to a 4-mL borosilicate vial equipped with a Teflon-coated magnetic stir bar were added the $Na_2S_2O_3 \cdot 5H_2O$ (49.6 mg, 0.200 mmol, 1.00 equiv.) and $Fe(NO_3)_3 \cdot 9H_2O$ (80.8 mg, 0.200 mmol, 1.00 equiv.). The vial was sealed with a septum cap and the mixture left at 25 °C for 3 min until the formation of a brown gas in the headspace of the vial was observed. The headspace was taken up with a 10 mL plastic syringe by piercing the septum with a needle. Subsequently, the headspace was directly injected into the IR spectrometer and an IR spectrum was recorded. Reference spectra were measured by directly injecting gaseous samples into the IR spectrometer.

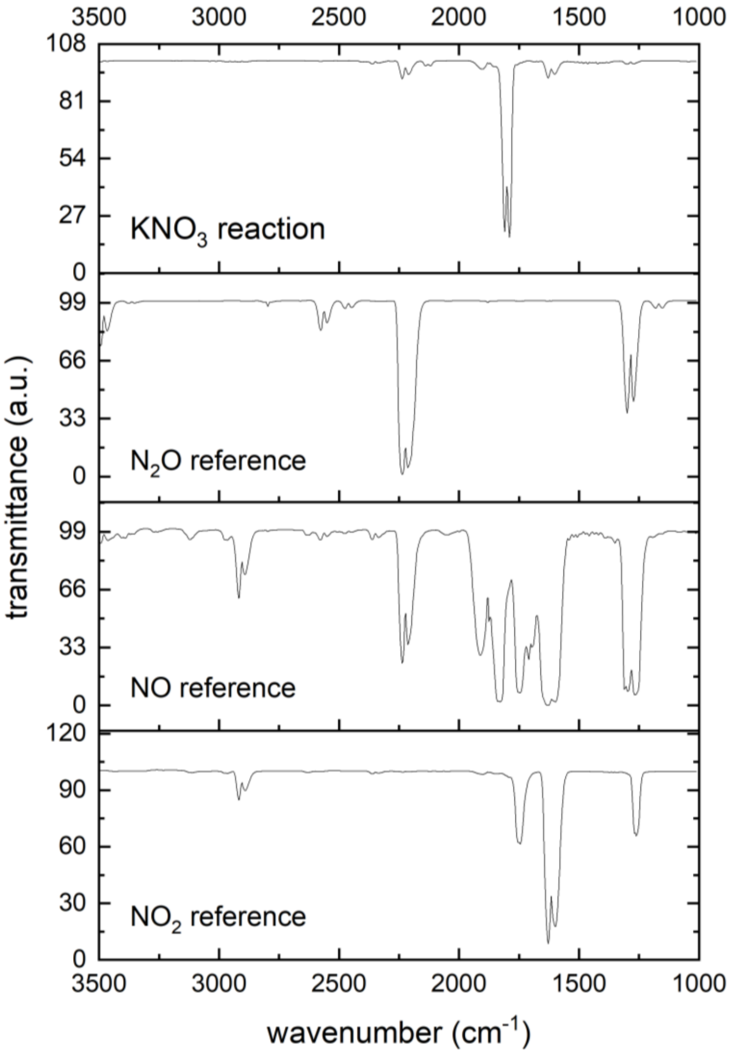

Fig S3. Stacked gas phase IR spectra at 23 °C of reaction mixture headspace (top) with reference spectra of N_2O, NO, and NO_2.

NO_2 detection with UV-Vis spectroscopy

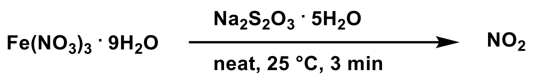

Under an ambient atmosphere, to a 4-mL borosilicate vial equipped with a Teflon-coated magnetic stir bar were added the $Na_2S_2O_3{\cdot}5H_2O$ (49.6 mg, 0.200 mmol, 1.00 equiv.) and $Fe(NO_3)_3{\cdot}9H_2O$ (80.8 mg, 0.200 mmol, 1.00 equiv.). The vial was sealed with a septum cap and the mixture left at 25 °C for 3 min until the formation of a brown gas in the headspace of the vial was observed. The headspace was taken up with a 10 mL plastic syringe by piercing the septum with a needle. The headspace was then bubbled through a cuvette filled with MeCN (2 mL). Subsequently a UV-Vis spectrum was recorded. The reference spectrum of NO_2 was recorded by bubbling NO_2 through a cuvette containing MeCN.

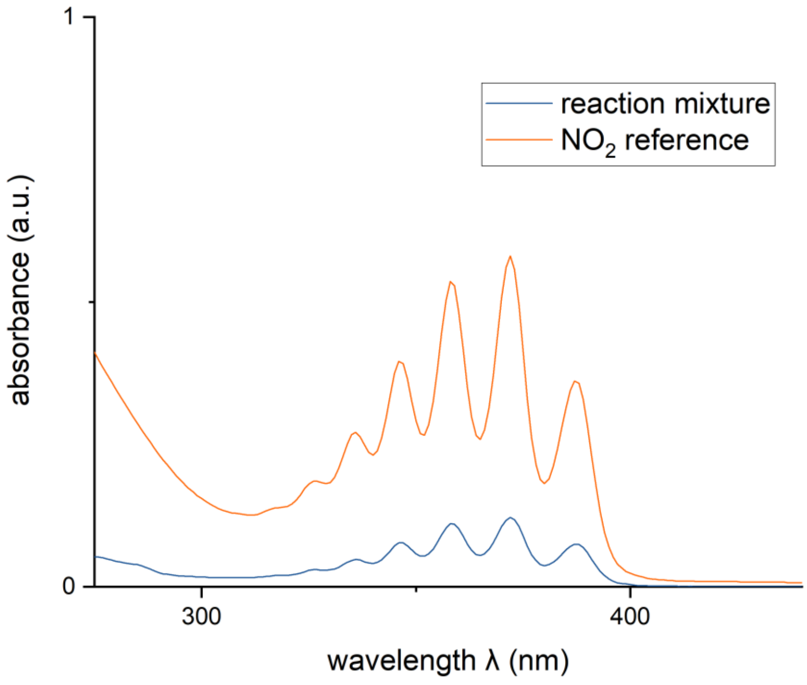

Fig S4. Stacked UV-Vis spectra at 23 °C in MeCN of reaction mixture headspace with reference spectrum of NO_2.

Mass spectrometric analysis

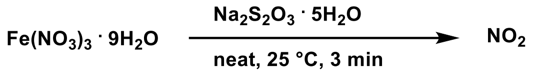

Under an ambient atmosphere, to a 4-mL borosilicate vial equipped with a Teflon-coated magnetic stir bar were added the $Na_2S_2O_3 \cdot 5H_2O$ (49.6 mg, 0.200 mmol, 1.00 equiv.) and $Fe(NO_3)_3 \cdot 9H_2O$ (80.8 mg, 0.200 mmol, 1.00 equiv.). The vial was sealed with a septum cap and the mixture left at 25 °C for 3 min until the formation of a brown gas in the headspace of the vial was observed. Any formed gases were vented by flushing the vial with Ar for 5 min. The residual solids were suspended in H_2O. The suspension was filtered and an ESI-MS measurement of the solution was carried out.

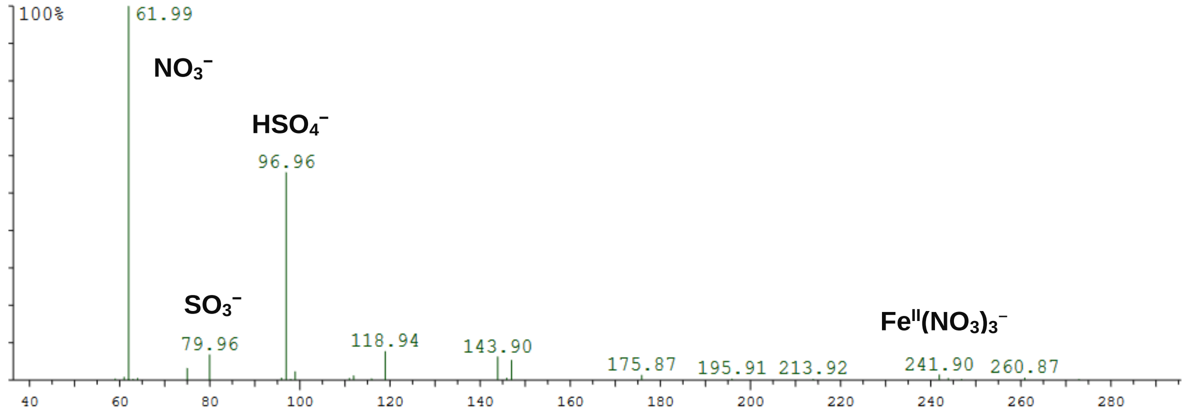

Fig S5. ESI-MS spectrum of H_2O solution after NO_2 formation. **Negative ionization** mode.

HRMS-ESI of sulfite (m/z) calc'd for O_3S_1 $[M]^-$, 79.9574; found, 79.9574; deviation: −0.9 ppm.

HRMS-ESI of bisulfate (m/z) calc'd for $H_1O_4S_1$ $[M]^-$, 96.9601; found, 96.9601; deviation: −0.3 ppm.

HRMS-ESI of $Fe^{II}(NO_3)_3^-$ (m/z) calc'd for $Fe_1N_3O_9$ $[M]^-$, 241.8989; found, 241.8993; deviation: −1.5 ppm.

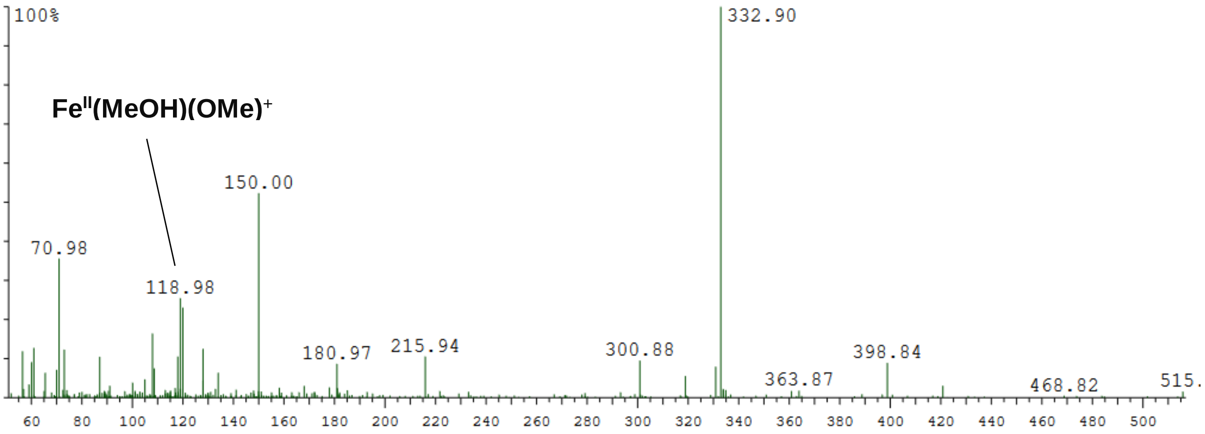

Fig S6. ESI-MS spectrum of H_2O solution after NO_2 formation. **Positive ionization** mode.

HRMS-ESI of $Fe^{II}(MeOH)(OMe)^+$ (m/z) calc'd for $C_2H_7Fe_1O_2$ $[M]^+$, 118.9790; found, 118.9792; deviation: −1.3 ppm.

Note: Methanol was used as solvent for the ESI-MS measurement.

Control experiments deaminative sulfochlorination

Reactions with 2-ethylhexyl nitrate

Under an ambient atmosphere, to a 4-mL borosilicate vial equipped with a Teflon-coated magnetic stir bar were added 4-aminobenzonitrile (11.8 mg, 0.100 mmol, 1.00 equiv.), $NaHSO_3$ (26.0 mg, 0.250 mmol, 2.50 equiv.) and CuCl (9.9 mg, 0.10 mmol, 1.0 equiv.). Then, acetonitrile (MeCN, 0.5 mL), 2-ethylhexyl nitrate (30.3 µL, 35.1 mg, 0.20 mmol, 2.0 equiv.), HCl (2.00 M in Et_2O, 0.1 mL, 0.20 mmol, 2.0 equiv.), and SO_2 (3.00 M in MeCN) were added, the vial was quickly sealed with a septum cap and the mixture stirred for 5 min at 23 °C. After cooling to 23 °C, the resulting mixture was concentrated by rotary evaporation under reduced pressure. The residue was diluted with water (1 mL) and extracted with DCM (3 × 1 mL). The organic layers were combined, the solvent was removed under reduced pressure, and the residue dissolved in $CDCl_3$ (0.6 mL). Dibromomethane was added as internal standard (7.0 µL, 17 mg, 0.10 mmol, 1.0 equiv.). The mixture was filtered and a ^{1}H-NMR spectrum recorded. The integration of the CH_2Br_2 signal (s, 5.0 ppm) was compared to the integration of a signal corresponding to aromatic protons of **1** (d, 8.2 ppm) and to the integration of a signal corresponding to aromatic protons of **2** (d, 7.5 ppm) to determine the yield.

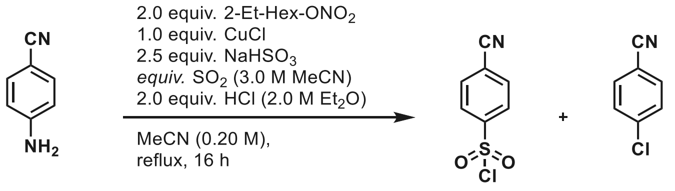

1 2

Table S12. Reaction outcome with 2-ethylhexyl nitrate and different equivalents of SO_2.

Entry	Equivalents SO_2	Yield **1*** (%)	Yield **2*** (%)
1	2.0	43	32
2	5.0	43	16

*·The yield was determined by ^{1}H NMR spectroscopy at 500 MHz and 298 K by dissolving the residue of the reaction mixture in 0.6 mL of $CDCl_3$, and using CH_2Br_2 as internal standard (7.0 µL, 17 mg, 0.10 mmol, 1.0 equiv.). The integration of the CH_2Br_2 signal at 4.96 ppm (s, 2H) was compared to the signal of **1** at 8.2 ppm (d, J = 8.7 Hz, 2H) and to the signal of **2** at 7.5 ppm (d, J = 8.6 Hz, 2H).

Reaction at different temperatures

Under an ambient atmosphere, to a 4-mL borosilicate vial equipped with a Teflon-coated magnetic stir bar were added 4-aminobenzonitrile (11.8 mg, 0.100 mmol, 1.00 equiv.), $Na_2S_2O_3$ $5H_2O$ (24.8 mg, 0.100 mmol, 1.00 equiv.) and CuCl (9.9 mg, 0.10 mmol, 1.0 equiv.). Then, acetonitrile (MeCN, 0.5 mL), HCl (4.0 M in 1,4-dioxane, 50 µL, 0.20 mmol, 2.0 equiv.), and SO_2 (3.0 M in MeCN, 333 µL, 1.00 mmol, 10.0 equiv.) were added, the vial was quickly sealed with a septum cap and the mixture stirred for 5 min at 23 °C. Subsequently, iron(III) nitrate nonahydrate (40.0 mg, 0.100 mmol, 1.00 equiv.) was added to the suspension and the vial was quickly resealed with a septum cap. The mixture was then stirred at different temperatures for 18 h. After cooling to 23 °C, the resulting mixture was concentrated by rotary evaporation under reduced pressure. The residue was diluted with water (1 mL) and extracted with DCM (3 × 1 mL). The organic layers were combined, the solvent was removed under reduced pressure, and the residue dissolved in $CDCl_3$ (0.6 mL). Dibromomethane was added as internal standard (7.0 µL, 17 mg, 0.10 mmol, 1.0 equiv.). The mixture was filtered and a ^{1}H-NMR spectrum recorded. The integration of the CH_2Br_2 signal (s, 5.0 ppm) was compared to the integration of a signal corresponding to aromatic protons of **1** (d, 8.2 ppm) to determine the yield.

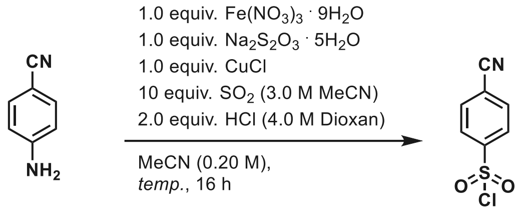

1

Table S13. Reaction outcome at different temperatures.

Entry	Temperature	Yield **1*** (%)
1	25 °C	67
**2**	**30 °C**	**77**
**3**	**40 °C**	**77**

*.The yield was determined by ^{1}H NMR spectroscopy at 500 MHz and 298 K by dissolving the residue of the reaction mixture in 0.6 mL of $CDCl_3$, and using CH_2Br_2 as internal standard (7.0 µL, 17 mg, 0.10 mmol, 1.0 equiv.). The integration of the CH_2Br_2 signal at 4.96 ppm (s, 2H) was compared to the signal of **1** at 8.20 ppm (d, J = 8.7 Hz, 2H).

Reaction with aqueous HCl

Under an ambient atmosphere, to a 4-mL borosilicate vial equipped with a Teflon-coated magnetic stir bar were added 4-aminobenzonitrile (11.8 mg, 0.100 mmol, 1.00 equiv.), $Na_2S_2O_3$ $5H_2O$ (49.6 mg, 0.200 mmol, 2.00 equiv.), and CuCl (9.9 mg, 0.10 mmol, 1.0 equiv.). Then, acetonitrile (MeCN, 0.5 mL), HCl (aq. 37%), and SO_2 (3.0 M in MeCN) were added, the vial was quickly sealed with a septum cap and the mixture stirred for 5 min at 23 °C. Subsequently, iron(III) nitrate nonahydrate (40.0 mg, 0.100 mmol, 1.00 equiv.) was added to the suspension and the vial was quickly resealed with a septum cap. The mixture was then stirred at 23 °C for 18 h. The resulting mixture was concentrated by rotary evaporation under reduced pressure. The residue was diluted with water (1 mL) and extracted with DCM (3 × 1 mL). The organic layers were combined, the solvent was removed under reduced pressure, and the residue dissolved in $CDCl_3$ (0.6 mL). Dibromomethane was added as internal standard (7.0 µL, 17 mg, 0.10 mmol, 1.0 equiv.). The mixture was filtered and a ^{1}H-NMR spectrum recorded. The integration of the CH_2Br_2 signal (s, 5.0 ppm) was compared to the integration of a signal corresponding to aromatic protons of **1** (d, 8.2 ppm) to determine the yield.

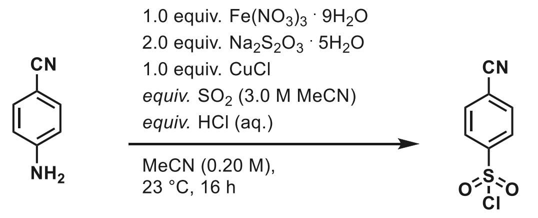

1

Table S14. Reaction outcome with aqueous HCl.

Entry	Equivalents SO_2	Equivalents HCl	Additional change	Yield **1*** (%)
1	2.0	4.0	No CuCl	<5
2	5.0	5.0	-	5

*. The yield was determined by ^{1}H NMR spectroscopy at 500 MHz and 298 K by dissolving the residue of the reaction mixture in 0.6 mL of $CDCl_3$, and using CH_2Br_2 as internal standard (7.0 µL, 17 mg, 0.10 mmol, 1.0 equiv.). The integration of the CH_2Br_2 signal at 4.96 ppm (s, 2H) was compared to the signal of **1** at 8.20 ppm (d, J = 8.7 Hz, 2H).

One-pot procedures for deaminative sulfoamidation

Under an ambient atmosphere, to a 4-mL borosilicate vial equipped with a Teflon-coated magnetic stir bar were added 4-aminobenzonitrile (11.8 mg, 0.100 mmol, 1.00 equiv.), $Na_2S_2O_3$ $5H_2O$ (49.6 mg, 0.200 mmol, 2.00 equiv.), and CuCl (9.9 mg, 0.10 mmol, 1.0 equiv.). Then, acetonitrile (MeCN, 0.5 mL), HCl (4.0 M in 1,4-dioxane, 50 µL, 0.20 mmol, 2.0 equiv.), and SO_2 (3.0 M in MeCN, 0.33 mL, 1.0 mmol, 10 equiv.) were added, the vial was quickly sealed with a septum cap and the mixture stirred for 5 min at 23 °C. Subsequently, iron(III) nitrate nonahydrate (40.0 mg, 0.100 mmol, 1.00 equiv.) was added to the suspension and the vial was quickly resealed with a septum cap. The mixture was then stirred at 40 °C for 18 h. After cooling to 23 °C, pyrrolidine was added as stated in Table S11 and the mixture stirred for 1 h at 23 °C. The resulting mixture was concentrated by rotary evaporation under reduced pressure. The residue was diluted with water (1 mL) and extracted with DCM (3 × 1 mL). The organic layers were combined, the solvent was removed under reduced pressure, and the residue dissolved in $CDCl_3$ (0.6 mL). Dibromomethane was added as internal standard (7.0 µL, 17 mg, 0.10 mmol, 1.0 equiv.). The mixture was filtered and a ^{1}H-NMR spectrum recorded. The integration of the CH_2Br_2 signal (s, 5.0 ppm) was compared to the integration of a signal corresponding to aromatic protons of **10** (m, 8.0 ppm) to determine the yield.

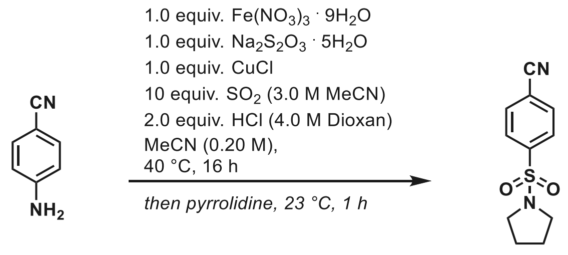

10

Table S15. Reaction outcome with different one-pot procedures without workup before amine addition.

Entry	Amine addition protocol	Yield **10*** (%)
1	10 equiv. pyrrolidine	11
2	6.0 equiv. TMEDA, 1 min stirring, then 6.0 equiv. pyrrolidine	<5
3	6.0 equiv. NaOAc, 2 min stirring, then 6.0 equiv. pyrrolidine	10
4	5.0 equiv Na_2SO_4, 2 min stirring, then 10 equiv. pyrrolidine	15
5	10 equiv. pyrrolidine added dropwise at 0 °C	12
6	Crude mixture diluted with 2 mL MeCN, filtered, then 10 equiv. pyrrolidine	34

*.The yield was determined by ^{1}H NMR spectroscopy at 500 MHz and 298 K by dissolving the residue of the reaction mixture in 0.6 mL of $CDCl_3$, and using CH_2Br_2 as internal standard (7.0 µL, 17 mg, 0.10 mmol, 1.0 equiv.). The integration of the CH_2Br_2 signal at 4.96 ppm (s, 2H) was compared to the signal of **10** at 8.00 ppm (m, 2H).

Control experiments

Under an ambient atmosphere, to a 20-mL borosilicate vial equipped with a Teflon-coated magnetic stir bar were added the 4-aminobenzonitrile (59.1 mg, 0.500 mmol, 1.00 equiv.), $Na_2S_2O_3$ $5H_2O$ (124 mg, 0.500 mmol, 1.00 equiv.) and CuCl (49.5 mg, 0.500 mmol, 1.0 equiv.). Then, acetonitrile (2.5 mL), HCl (4.0 M in 1,4-dioxane, 0.25 mL, 1.0 mmol, 2.0 equiv.), and SO_2 (3.0 M in MeCN, 1.7 mL, 5.0 mmol, 10 equiv.) were added, the vial was quickly sealed with a septum cap and the mixture stirred for 5 min at 23 °C. Subsequently, iron(III) nitrate nonahydrate (202 mg, 0.500 mmol, 1.00 equiv.) was added to the suspension and the vial was quickly resealed with a septum cap. The mixture was then stirred at 40 °C for 18 h. After cooling to 23 °C, the resulting mixture was concentrated by rotary evaporation under reduced pressure. The residue was diluted with water (5 mL) and extracted with DCM (3 × 5 mL).The organic layers were combined, the solvent was removed under reduced pressure, and the residue dissolved in $CDCl_3$ (2.5 mL). Dibromomethane was added as internal standard (35 µL, 87 mg, 0.50 mmol, 1.0 equiv.). The mixture was filtered and a ^{1}H-NMR spectrum recorded of an 0.5 mL aliquot. The integration of the CH_2Br_2 signal (s, 5.0 ppm) was compared to the integration of a signal corresponding to aromatic protons of **1** (d, 8.2 ppm) to determine the yield.

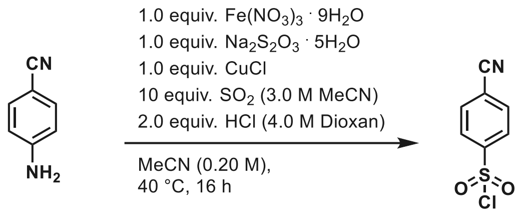

1

Table S16. Reaction outcome for different control experiments.

Entry	Change	Yield **1*** (%)
1	No CuCl	21
2	No HCl	46

*. The yield was determined by ^{1}H NMR spectroscopy at 500 MHz and 298 K by dissolving the residue of the reaction mixture in 2.5 mL of $CDCl_3$, and using CH_2Br_2 as internal standard (35 µL, 87 mg, 0.50 mmol, 1.0 equiv.). The integration of the CH_2Br_2 signal at 4.96 ppm (s, 2H) was compared to the signal of **1** at 8.2 ppm (d, J = 8.2 Hz, 2H).

Control experiments deaminative sulfonic acid synthesis

Reaction with SO_2

Under an ambient atmosphere, to a 4-mL borosilicate vial equipped with a Teflon-coated magnetic stir bar were added the 4-aminobenzonitrile (11.8 mg, 0.100 mmol, 1.00 equiv.) and KNO_3 (20.0 mg, 0.200 mmol, 2.00 equiv.). Then, acetonitrile (0.5 mL) and SO_2 (3.0 M stock solution in MeCN, equivalents in Table S17) were added, the vial was quickly sealed with a septum cap and the mixture stirred at 85 °C (oil bath temperature) for 18 h. After cooling to 23 °C, the resulting mixture was concentrated by rotary evaporation under reduced pressure. The residue was dissolved in DMSO d_6 (0.6 mL) and dibromomethane was added as internal standard (7.0 µL, 17 mg, 0.10 mmol, 1.0 equiv.). The mixture was filtered and a ^{1}H-NMR spectrum recorded. The integration of the CH_2Br_2 signal (s, 5.0 ppm) was compared to the integration of a signal corresponding to aromatic protons of sulfonic acid **44** (d, 7.78 ppm) to determine the yield.

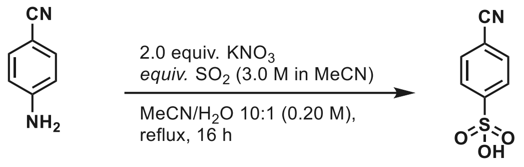

44

Table S17. Reaction outcome with different equivalents of SO_2.

Entry	SO_2 equiv.	Yield **44*** (%)
1	0.5	<5
2	1.0	11
3	2.0	29
4	3.0	30
**5**	**5.0**	**42**

*. The yield was determined by ^{1}H NMR spectroscopy at 500 MHz and 298 K by dissolving the residue of the reaction mixture in 0.6 mL of DMSO-d_6, and using CH_2Br_2 as internal standard (7.0 µL, 17 mg, 0.10 mmol, 1.0 equiv.). The integration of the CH_2Br_2 signal at 4.96 ppm (s, 2H) was compared to the signal of **44** at 7.78 ppm (d, J = 8.3 Hz, 2H).

Reaction with SO_2 in presence of additives

Under an ambient atmosphere, to a 4-mL borosilicate vial equipped with a Teflon-coated magnetic stir bar were added the 4-aminobenzonitrile (11.8 mg, 0.100 mmol, 1.00 equiv.) and KNO_3 (20.0 mg, 0.200 mmol, 2.00 equiv.). Then, acetonitrile (0.5 mL), additive (2.00 equiv., 0.200 mmol) and SO_2 (3 M in MeCN, 83.3 µL, 0.250 mmol, 2.50 equiv.) were added, the vial was quickly sealed with a septum cap and the mixture stirred at 85 °C (oil bath temperature) for 18 h. After cooling to 23 °C, the resulting mixture was concentrated by rotary evaporation under reduced pressure. The residue was dissolved in DMSO d_6 (0.6 mL) and dibromomethane was added as internal standard (7.0 µL, 17 mg,

0.10 mmol, 1.0 equiv.). The mixture was filtered and a ^{1}H-NMR spectrum recorded. The integration of the CH_2Br_2 signal (s, 5.0 ppm) was compared to the integration of a signal corresponding to aromatic protons of sulfonic acid **44** (d, 7.78 ppm) to determine the yield.

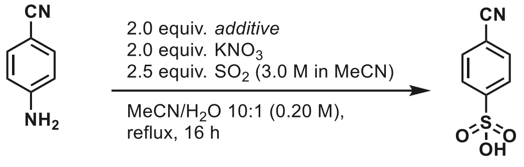

44

Table S18. Reaction outcome with different additives.

Entry	Additive	Yield **44*** (%)
1	-	56
**2**	**HCl (aq. 37%)**	**70**
3	K_2CO_3	62

*. The yield was determined by ^{1}H NMR spectroscopy at 500 MHz and 298 K by dissolving the residue of the reaction mixture in 0.6 mL of DMSO-d_6, and using CH_2Br_2 as internal standard (7.0 µL, 17 mg, 0.10 mmol, 1.0 equiv.). The integration of the CH_2Br_2 signal at 4.96 ppm (s, 2H) was compared to the signal of **44** at 7.78 ppm (d, J = 8.3 Hz, 2H).

Reaction with DABSO

Under an ambient atmosphere, to a 4-mL borosilicate vial equipped with a Teflon-coated magnetic stir bar were added the 4-aminobenzonitrile (11.8 mg, 0.100 mmol, 1.00 equiv.), KNO_3 (20.0 mg, 0.200 mmol, 2.00 equiv.), and DABSO (48.1 mg, 0.200 mmol, 2.00 equiv.). Then, solvent (0.5 mL) was added, the vial was quickly sealed with a septum cap and the mixture stirred at 85 °C (oil bath temperature) for 18 h. After cooling to 23 °C, the resulting mixture was concentrated by rotary evaporation under reduced pressure. The residue was dissolved in DMSO d_6 (0.6 mL) and dibromomethane was added as internal standard (7.0 µL, 17 mg, 0.10 mmol, 1.0 equiv.). The mixture was filtered and a ^{1}H-NMR spectrum recorded. The integration of the CH_2Br_2 signal (s, 5.0 ppm) was compared to the integration of a signal corresponding to aromatic protons of sulfonic acid **44** (d, 7.78 ppm) to determine the yield.

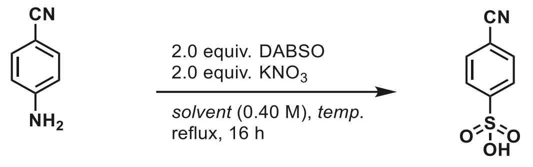

44

Table S19. Reaction outcome with different solvent/temperature combinations.

Entry	Solvent	Temperature	Yield **44*** (%)
1	MeCN + 2.0 equiv H_2O	85 °C	38
2	MeCN + 2.0 equiv H_2O	100 °C	48
**3**	**MeCN/H_2O 10:1**	**85 °C**	**66**
4	MeCN/H_2O 10:1	100 °C	65

*. The yield was determined by ^{1}H NMR spectroscopy at 500 MHz and 298 K by dissolving the residue of the reaction mixture in 0.6 mL of DMSO-d_6, and using CH_2Br_2 as internal standard (7.0 µL, 17 mg, 0.10 mmol, 1.0 equiv.). The integration of the CH_2Br_2 signal at 4.96 ppm (s, 2H) was compared to the signal of **44** at 7.78 ppm (d, J = 8.3 Hz, 2H).

Radical trapping experiments

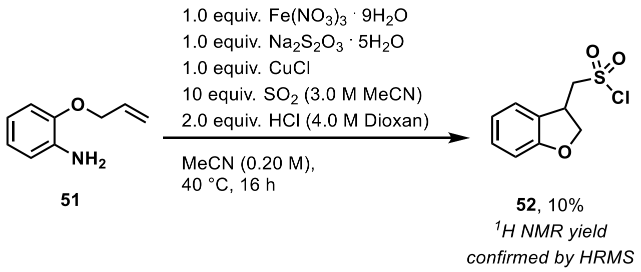

Caution: A blast shield must be used to minimize personal damage in case of an accident. See safety statement on page S8.

Under an ambient atmosphere, to a 20-mL borosilicate vial equipped with a Teflon-coated magnetic stir bar were added 2-(allyloxy)aniline (123 mg, 0.500 mmol, 1.00 equiv.), $Na_2S_2O_3$ $5H_2O$ (124 mg, 0.500 mmol, 1.00 equiv.) and CuCl (49.5 mg, 0.500 mmol, 1.0 equiv.). Then, acetonitrile (2.5 mL), HCl (4.0 M in 1,4-dioxane, 0.25 mL, 1.00 mmol, 2.0 equiv.), and SO_2 (3.0 M in MeCN, 1.7 mL, 5.0 mmol, 10 equiv.) were added, the vial was quickly sealed with a septum cap and the mixture stirred for 5 min at 23 °C. Subsequently, iron(III) nitrate nonahydrate (202 mg, 0.500 mmol, 1.00 equiv.) was added to the suspension and the vial was quickly resealed with a septum cap. The mixture was then stirred at 40 °C for 18 h. After cooling to 23 °C, the resulting mixture was concentrated by rotary evaporation and the residue dissolved in $CDCl_3$ (2.5 mL). Dibromomethane was added as internal standard (35.0 µL, 86.9 mg, 0.50 mmol, 1.0 equiv.). The mixture was filtered and a ^{1}H-NMR spectrum recorded of a 0.5 mL aliquot. The integration of the CH_2Br_2 signal (s, 5.0 ppm) was compared to the integration of a signal corresponding to aliphatic protons of **52** (d, 6.72 ppm) to determine the yield.

HRMS-EI(m/z) of **54** calc'd for $C_9H_9O_3S_1Cl_1$ $[M]^+$, 231.9956; found, 231.9955; deviation: +0.2 ppm.

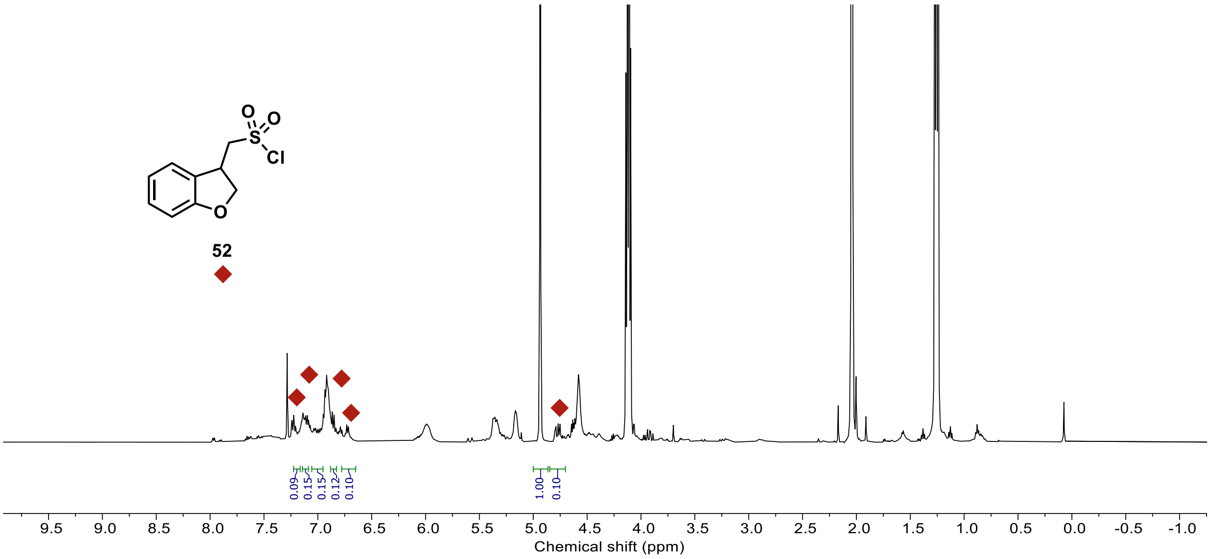

Fig S7. Excerpt of ^{1}H NMR spectrum recorded in $CDCl_3$ solution at 23°C and 500 MHz of the deaminative sulfochlorination to give **52**.

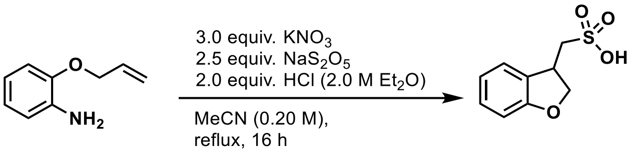

Caution: A blast shield must be used to minimize personal damage in case of an accident. See safety statement on page S8.

Under an ambient atmosphere, to a 4-mL borosilicate vial equipped with a Teflon-coated magnetic stir bar were added the 2-(allyloxy)aniline (14.9 mg, 0.100 mmol, 1.00 equiv.), KNO_3 (30.0 mg, 0.300 mmol, 2.00 equiv.) and $Na_2S_2O_5$ (48.0 mg, 0.250 mmol, 2.50 equiv.). Then, acetonitrile (0.5 mL), and HCl (2.0 M Et_2O, 0.10 mL, 2.0 mmol, 2.0 equiv.) were added, the vial was quickly sealed with a septum cap and the mixture stirred at 85 °C (oil bath temperature) for 18 h. After cooling to 23 °C, the resulting mixture was concentrated by rotary evaporation and the residue dissolved in DMSO-d_6 (0.6 mL). Dibromomethane was added as internal standard (7.0 µL, 17 mg, 0.10 mmol, 1.0 equiv.). The mixture was filtered and a ^{1}H-NMR spectrum recorded. The integration of the CH_2Br_2 signal (s, 5.0 ppm) was compared to the integration of a signal corresponding to aliphatic protons of **53** (d, 3.06 ppm) to determine the yield.

HRMS-ESI(m/z) of **53** calc'd for $C_9H_9O_4S_1$ $[M+H]^-$, 213.0227; found, 213.0230; deviation: −1.2 ppm.

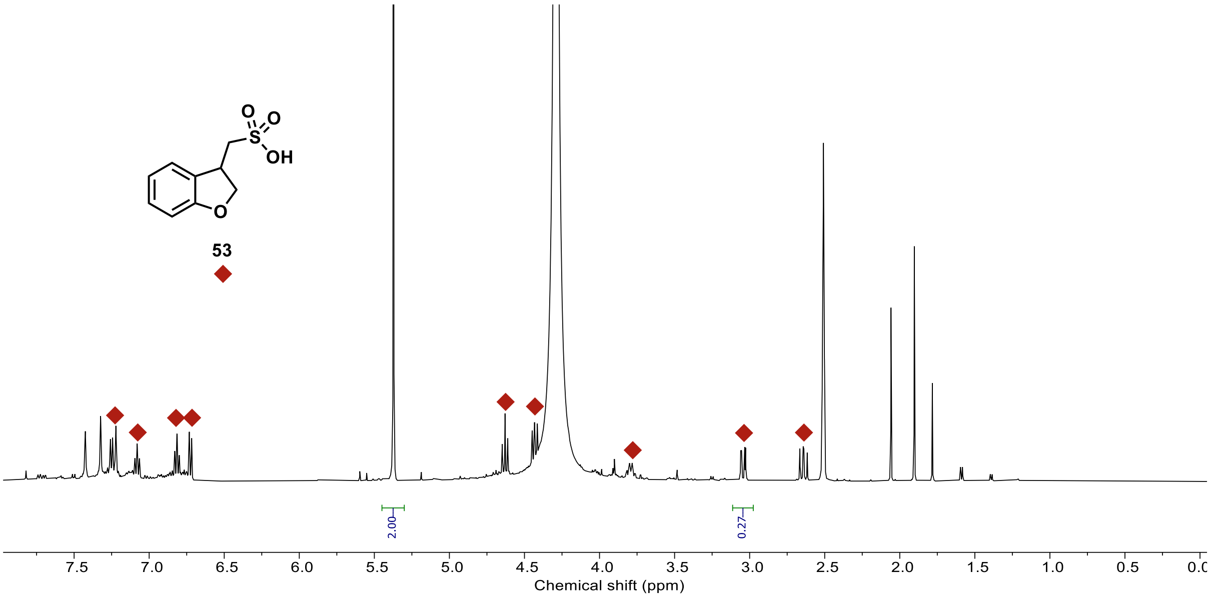

Fig S8. Excerpt of ^{1}H NMR spectrum recorded in DMSO-d_6 solution at 23°C and 500 MHz of the deaminative sulfonylation reaction to give **53**.

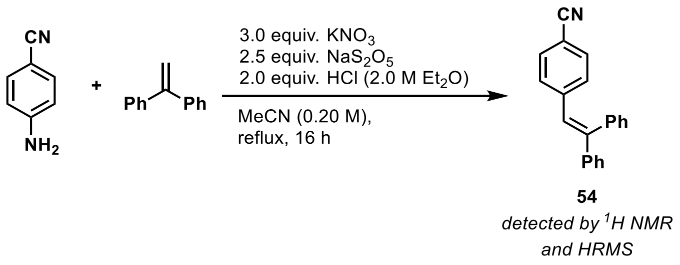

Caution: A blast shield must be used to minimize personal damage in case of an accident. See safety statement on page S8.

Under an ambient atmosphere, to a 4-mL borosilicate vial equipped with a Teflon-coated magnetic stir bar were added the 4-aminobenzonitrile (11.8 mg, 0.100 mmol, 1.00 equiv.), KNO_3 (30.0 mg, 0.300 mmol, 2.00 equiv.) and $Na_2S_2O_5$ (48.0 mg, 0.250 mmol, 2.50 equiv.). Then, acetonitrile (0.5 mL), 1,1-diphenylethylene (53.0 µL, 54.1 mg, 0.300 mmol, 3.00 equiv.) and HCl (2.0 M Et_2O, 0.10 mL, 2.0 mmol, 2.0 equiv.) were added, the vial was quickly sealed with a septum cap and the mixture stirred at 85 °C (oil bath temperature) for 18 h. After cooling to 23 °C, the resulting mixture was concentrated by rotary evaporation under reduced pressure. The residue was dissolved in hexanes/DCM 1:1 (0.5 mL) and filtered over a silica plug using hexanes/DCM 1:1 as eluent (4 mL). The filtrate was concentrated by rotary evaporation under reduced pressure and the resulting residue dissolved in $CDCl_3$ (0.6 mL). The mixture was filtered and a ^1H NMR spectrum recorded. The signals are in accordance to the literature reference.[10]

HRMS-ESI(m/z) of **54** calc'd for $C_{21}H_{15}N_1Na_1$ $[M+Na]^+$, 304.1097; found, 304.1094; deviation: +1.0 ppm.

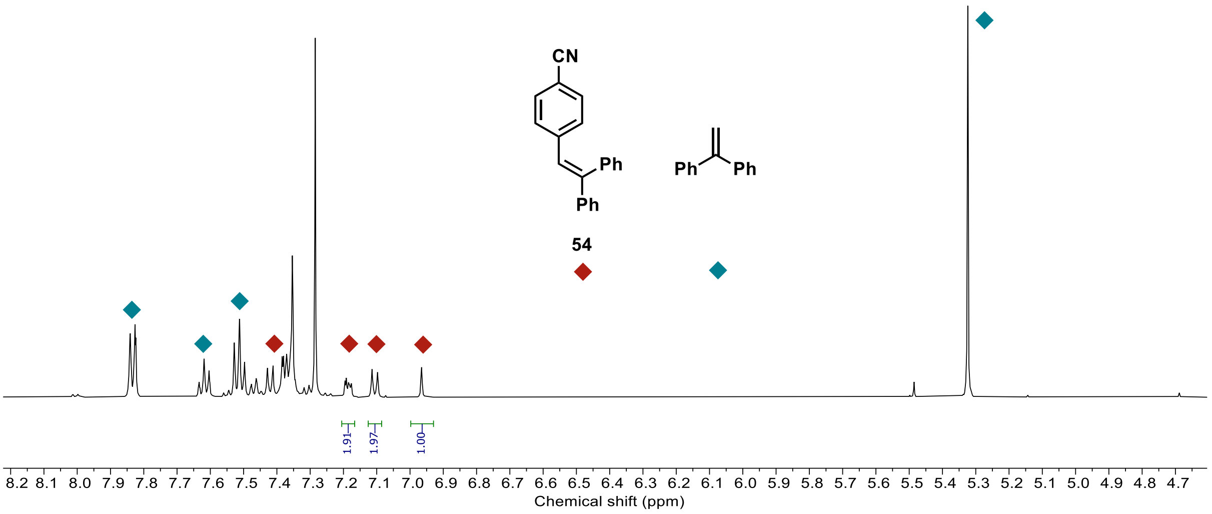

Fig S9. Excerpt of ^1H NMR spectrum recorded in $CDCl_3$ solution at 23°C and 500 MHz of the deaminative sulfonylation reaction in presence of 1,1-diphenylethylene.

Reaction monitoring with NMR spectroscopy

Reaction monitoring by ^{1}H NMR spectroscopy

Under an ambient atmosphere, to a 4-mL borosilicate vial were added the 4-aminobenzonitrile (11.8 mg, 0.100 mmol, 1.00 equiv.) and $TBANO_3$ (91.3 mg, 0.300 mmol, 3.00 equiv.). Then, acetonitrile (0.5 mL) and SO_2 (3.0 M in MeCN, 83 µL, 0.25 mmol, 2.5 equiv.) were added, the vial was quickly sealed and inverted several times until a clear solution is obtained. The mixture was transferred to a J-Young NMR tube and the tube sealed with a Teflon cap. Then, the tube was inserted in the NMR spectrometer in a 80°C preheated probe. The reaction was monitored by recording single scan ^{1}H NMR spectra (every 5 minutes).

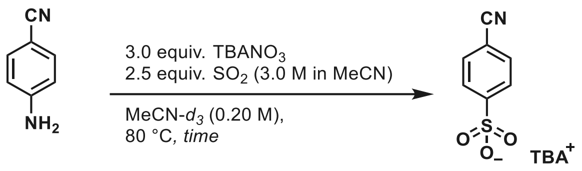

55

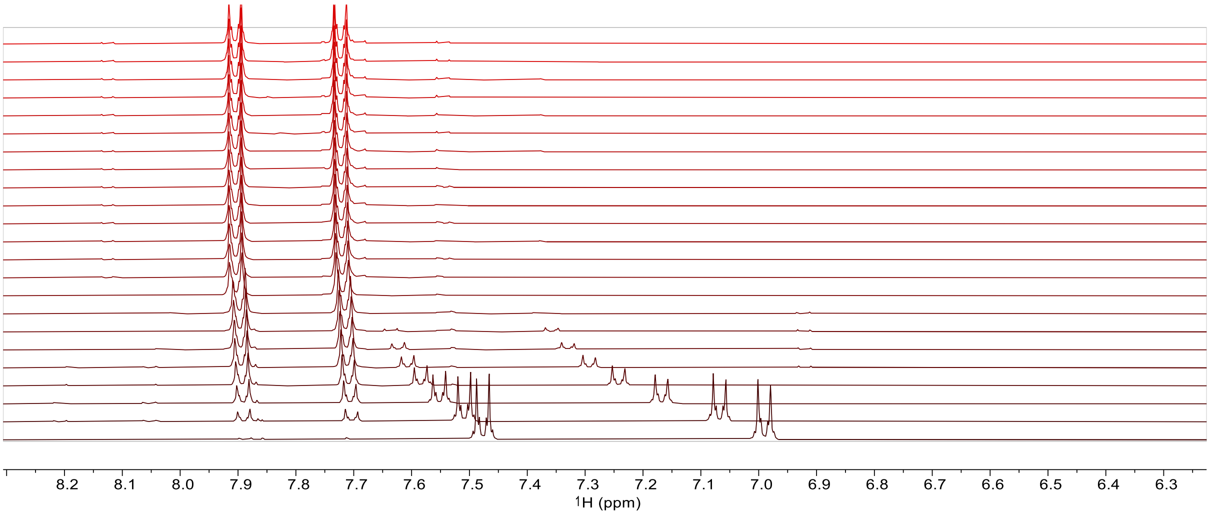

Fig S10. Excerpt of stacked ^{1}H NMR spectra recorded in MeCN-d_3 solution at 80°C and 400 MHz of the deaminative sulfonylation reaction to give **55**. Bottom, t = 0: Top, t = 110 min.

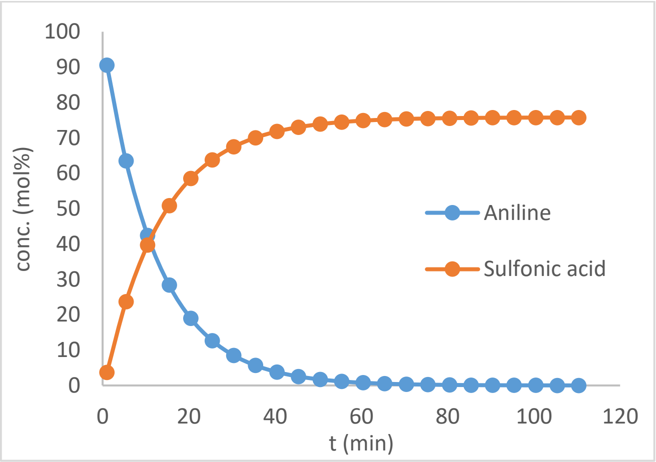

Fig S11. Concentration changes of the deaminative sulfonic acid synthesis depicted in Fig. S8.

Reaction monitoring by ^{17}O and ^{14}N NMR spectroscopy

Under an ambient atmosphere, to a 4-mL borosilicate vial were added the 4-aminobenzonitrile (11.8 mg, 0.100 mmol, 1.00 equiv.) and $TBANO_3$ (91.3 mg, 0.300 mmol, 3.00 equiv.). Then, acetonitrile (0.5 mL) and SO_2 (3 M in MeCN, 83.3 µL, 0.250 mmol, 2.50 equiv.) were added, the vial was quickly sealed and inverted several times until a clear solution is obtained. The mixture was transferred to a J-Young NMR tube and the tube sealed with a Teflon cap. Then, a ^{17}O and ^{14}N NMR spectrum was recorded. Subsequently, the J-Young tube was heated at 80 °C for 2 h. After cooling to 23 °C, another ^{17}O and ^{14}N NMR spectrum was measured.

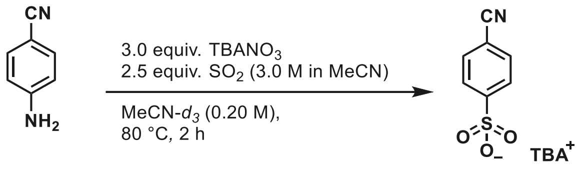

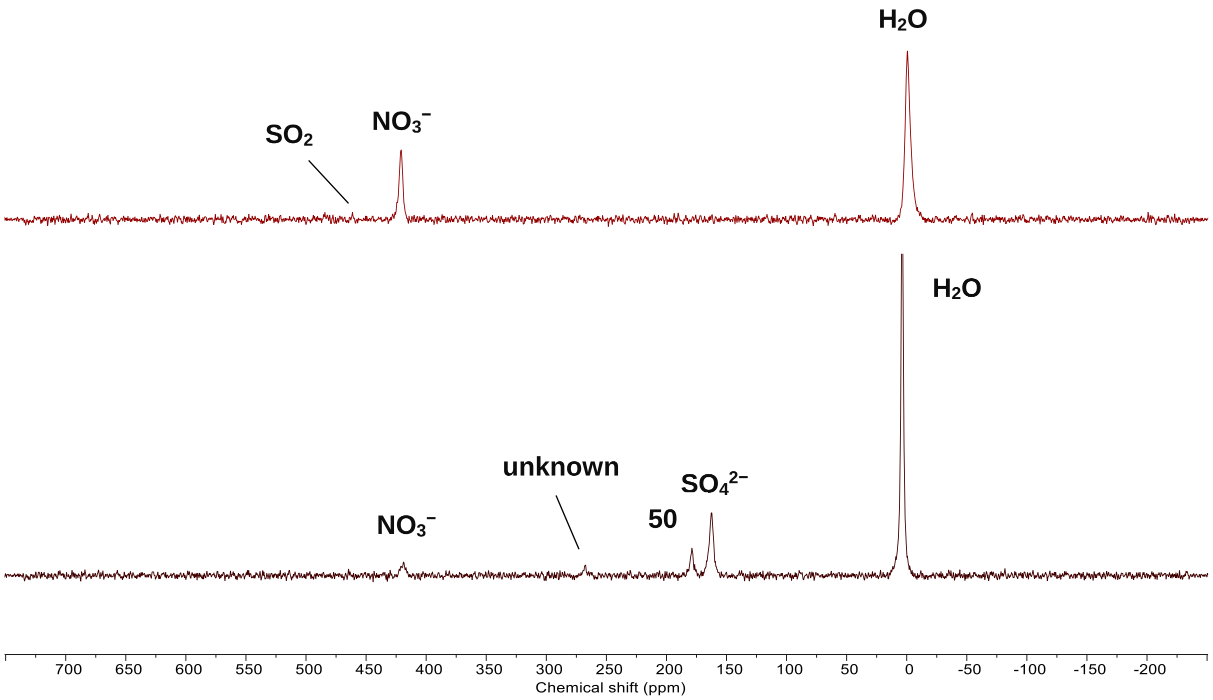

Fig S12. Stacked ^{17}O NMR spectra recorded in MeCN-d_3 solution at 23°C and 54 MHz of the deaminative sulfonylation reaction to give **55**. Top, t = 0: Bottom, t = 120 min. Whittaker smoother correction applied during processing of the spectrum, which might suppress broad signals.

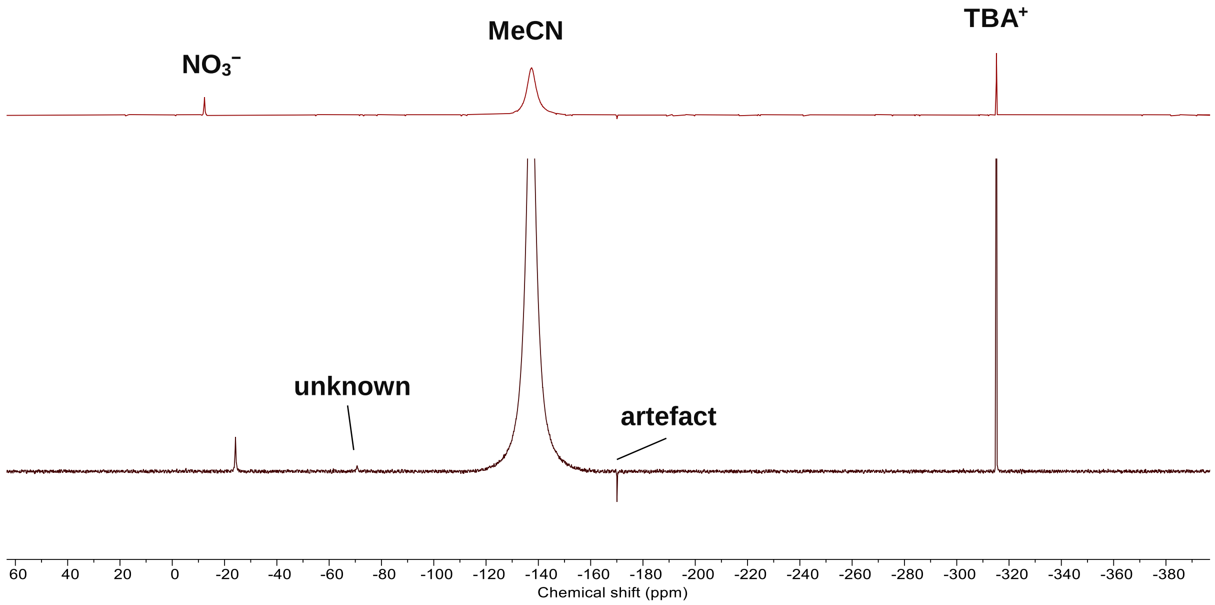

Fig S13. Stacked ^{14}N NMR spectra recorded in MeCN-d_3 solution at 23°C and 29 MHz of the deaminative sulfonylation reaction to give **55**. Top, t = 0: Bottom, t = 120 min. Nitrate signal shifts due to change of pH during the deaminative sulfonylation reaction. NO_2 not visible in ^{14}N NMR because NO_2 is paramagnetic. Both spectra scaled differently.

COMPUTATIONAL DATA

Nitrate reduction with thiosulfate

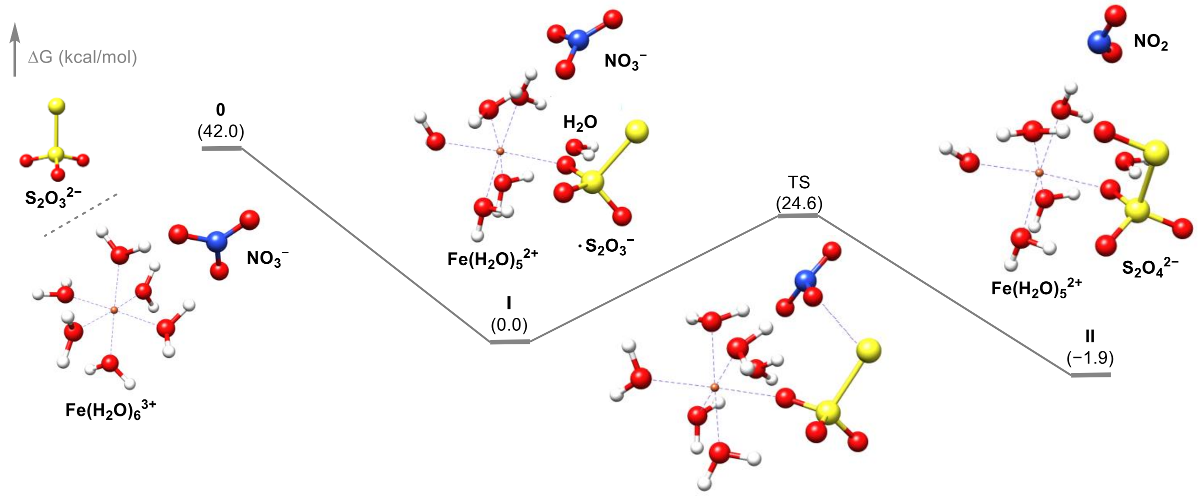

Fig S14. Nitrate reduction path of iron nitrate mixed with thiosulfate obtained by computations at the M06-2X-D3(0)/ma-def2-TZVP and M06-2X-D3(0)/ma-def2-QZVP//M06-2X-D3(0)/ma-def2-TZVP levels using the ORCA program package.

Table S20. The electronic energy Δ*E*, enthalpy Δ*H*, and free energy ΔG_{298} of the stationary points (in kcal/mol) on the lowest sextet energy surface for NO_2 release in the reaction of $Fe(H_2O)_6^{3+}$, NO_3^-, and $S_2O_3^{2-}$ computed at the M06-2X-D3(0)/ma-def2-TZVP and M06-2X-D3(0)/ma-def2-QZVP//M06-2X-D3(0)/ma-def2-TZVP levels, treating acetonitrile solvent molecules implicitly with the SMD model. All the energies are given with respect to those of **I**.

	M06-2X-D3(0)/ma-def2-TZVP			M06-2X-D3(0)/ma-def2-QZVP		
	ΔE	ΔH	ΔG_{298}	ΔE	ΔH	ΔG_{298}
**0**[a]	59.9	55.7	44.2	57.7	53.5	42.0
**I**	0.0	0.0	0.0	0.0	0.0	0.0
**TS**	26.0	23.8	24.8	25.8	23.6	24.6
**II**	0.8	−1.1	-2.0	0.9	−1.0	−1.9

[a] **0** corresponds to the isolated $S_2O_3^{2-}$ and $^6Fe(H_2O)_6^{3+}$•••NO_3^-. Upon interaction between these two reactants, an electron is spontaneously transferred from the sulfur center to the iron center, resulting in the iron center adopting a quintet spin state.

Cartesian coordinates (Å) of all optimized structures given on Fig S12.

$S_2O_3^{2-}$

−2 1

S	5.59980476312688	5.07721689144936	3.13840459297513
O	4.95198434279361	6.10472852626970	3.97304786997611
O	4.60889841949760	4.38189282887533	2.29755819117306
O	6.34785484097304	4.11823975794843	3.97117876242132
S	6.93604149462400	6.02007329901855	1.89691249231059

$Fe(H_2O)_6^{3+}\cdots NO_3^-$

2 6

Fe	7.11878421343954	7.06244030040815	6.61884806060835
O	8.75955698771920	8.10799954354331	6.57789717988639
H	8.89994436174132	8.95099657297225	7.03936489549749
H	9.62804664685927	7.81031783534027	6.02199669591563
O	6.20727590698356	8.30882628764609	7.94609815486203
H	6.34811443952278	8.29127440997160	8.90942981634011
H	5.69757219817164	9.10523874510423	7.71374651392354
O	7.74181603095680	6.02284432846447	8.24822980384103
H	8.66204992946417	6.03575170518340	8.56794910991110
H	7.28949159417407	5.24638514850289	8.62422240074690
O	5.41588908006755	5.96243773930338	6.61929587719366
H	5.22113516260299	5.22402916678908	6.01388928795824
H	4.61819179946612	6.15987489700150	7.14259730216147
O	7.91689370856593	5.83016074229654	5.30965099632010
H	8.15119993919778	4.90446098812671	5.49107491884794
H	8.57918433262807	6.24821014148097	4.61317772235512
O	6.31515394832945	8.21982818685817	5.15654724573723
H	5.53629115585452	7.98477389333195	4.62101671055853
H	6.80647184842198	8.92676565220480	4.70089477685715
N	10.58478815369924	7.22761573906756	4.09348676044635
O	9.42837307634433	6.93363466807096	3.68148501741910
O	10.75131242566449	7.41927350396993	5.33519422522408
O	11.50642719910823	7.32414350079876	3.33354361853092

I

0 6

Fe	7.58629685584309	7.89622035114666	6.06757952219953
O	9.75626800693373	7.93049082586199	6.04592170749906
H	10.29545915300971	7.44861460042823	6.68797814828024
H	10.03946383337687	7.65093711616576	5.15401321338447
O	7.49891391484648	9.30901621219558	7.66646902770411
H	7.00287608159069	9.04040256903847	8.45421566938773
H	7.23145691911560	10.21668120727233	7.46001779911904
O	7.64579680712506	9.54460704737571	4.73258359171044
H	7.22429298789245	9.34300918331908	3.85320423324736
H	8.49247244285760	9.97798500018042	4.56118241346701
O	5.56965357582298	5.11729694461345	6.05441426855993
H	6.15390509121555	5.03786767500173	5.28336290812025
H	5.00808993107296	4.33109502084873	6.07734640868902
O	7.31287687083576	6.45010010743601	7.65081281506064
H	6.71131655060755	5.74510423115289	7.34128510601539
H	8.05125663150494	6.03131642259326	8.11372637091932
O	5.45146653374125	7.90854557653605	5.84310564325155
H	5.17418155440735	8.27507699746690	4.97463938341630
H	5.10923297306111	7.00014365303069	5.88907933135448
N	5.35833206486100	8.66802483694016	2.36758198896783
O	6.60978913608146	8.81112013269676	2.44455518124999
O	4.66943064548108	8.80872233366073	3.39723050789210

O	4.84568458451804	8.39647116224507	1.29657394116465
S	8.55779966018481	6.06705438092263	3.48725854358477
O	7.65413565659780	6.32730401206655	4.63750401046631
O	9.60497144978157	7.07166841237893	3.43370668426186
O	8.99615080821788	4.69454536184439	3.44069156932670
S	7.40969827940756	6.19462762557245	1.78213001169427

TS

0 6

Fe	7.62860608859008	7.97486248807171	6.11764508388080
O	9.76541343738051	7.85541698167977	6.24661070186950
H	10.27290763552113	7.45776096541978	6.96652468790961
H	10.11700114799742	7.53410460997832	5.39362123776336
O	7.61886474862046	9.45705853609256	7.67318467746164
H	7.60986665310573	9.18534692361802	8.60255551161045
H	7.01668757298434	10.21110142246528	7.59438925824792
O	7.95980578287398	9.53894202686177	4.67674275944261
H	7.47089463139738	9.34336614998525	3.85948616660784
H	8.87613189905834	9.71546013250048	4.41757663729687
O	5.28584039887828	5.29410554312150	5.78052340516995
H	5.57092997012786	5.64787932566324	4.92140422962389
H	5.12522716005117	4.35135523144387	5.64433585118442
O	7.08564725327780	6.38514411348923	7.43406011490921
H	6.44744762867735	5.82148780292813	6.93029772242840
H	7.76240737010985	5.80448071639168	7.80777582775509
O	5.56251390362317	8.31888844046304	5.83416143983666
H	5.23498617617532	7.92045949452985	4.97075287465785
H	4.92998836733244	8.08425692295815	6.52610295515432
N	5.20301859009264	7.96097198880683	2.60393426852962
O	6.51400981523877	8.29553070771691	2.52629037851398
O	4.87085579585477	7.23621318987065	3.59862046444555
O	4.57598442339781	7.97131511188752	1.54261897521606
S	8.60425968081228	6.32756664882400	3.44610624852153
O	7.67634902545073	6.45496485860468	4.58613420027487
O	9.75097149034030	7.21182462744342	3.61719469244971
O	8.93514351719655	4.96492898180185	3.11845801575629
S	7.54950883582543	6.96925505737394	1.76106161347627

II

0 6

Fe	7.52698560234611	7.87711432109408	6.05414564932478
O	9.67628587240846	7.88993722389587	6.02547148262988
H	10.20397833238204	7.56585273066767	6.76769469052974
H	9.93290636989167	7.38002812192499	5.22071271214080
O	7.52211175361991	9.36318930526451	7.61307740890567
H	6.91140642860854	9.24875563303280	8.35532497563433
H	7.57138651578452	10.31226767665187	7.43011969334615
O	7.35013036913433	9.29002337104899	4.51097356869753
H	7.45365874073192	8.92181521904155	3.52904080412949
H	7.87260246128320	10.09891722111847	4.57473093710871
O	5.66534502246068	5.03730142693507	6.14077477005009
H	6.28705619805683	5.06620402356865	5.39104258722123
H	5.18141058563141	4.20274371026711	6.09638100612745
O	7.30517139039211	6.51874585818830	7.72444412712873
H	6.81268483205829	5.72164379548411	7.45351889633207
H	8.00497841480563	6.25369199319314	8.33623922816850
O	5.36639465894827	7.78664926722989	5.96161754741114
H	5.08223518822183	8.14237795524209	5.10632925570553
H	5.09162130035006	6.85151648517377	5.98656906380817
N	5.09886280458979	9.19690652695191	2.41725611134617

O	7.62529483409955	8.44946197361386	2.20071914995260
O	4.67823111156788	8.31582344674402	3.09021876303602
O	4.87773509222332	9.56283008829218	1.32288714469987
S	8.47212424160659	5.97117859425864	3.48496603462265
O	7.62351853632655	6.23282008030858	4.69271617595606
O	9.79532182216557	6.55682757300307	3.71378810545036
O	8.46345540976953	4.57043875647416	3.13020965241373
S	7.56837511053177	6.90898662132703	1.86720045811994

Sulfonic acid formation from aryldiazonium salt

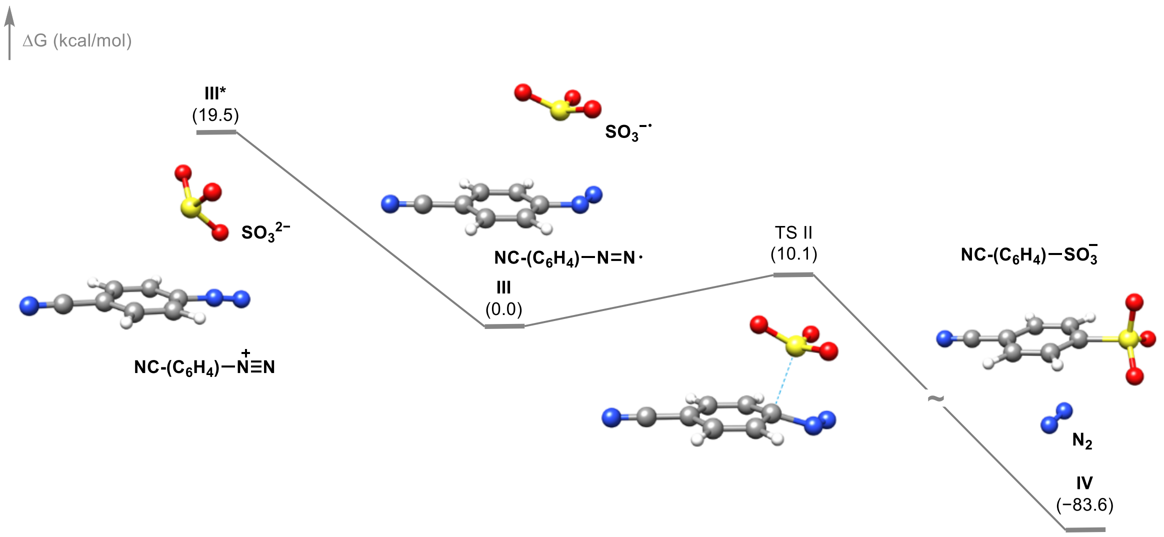

Fig S15. Sulfonylation path of the diazonium salt through SO_3^{2-} obtained by computations at the M06-2X-D3(0)/ma-def2-TZVP and M06-2X-D3(0)/ma-def2-QZVP//M06-2X-D3(0)/ma-def2-TZVP levels using the ORCA program package.

Table S21. The electronic energy ΔE, enthalpy ΔH, and free energy ΔG_{298} of the stationary points (in kcal/mol) on the sulfonylation path of the diazonium salt through SO_3^{2-} computed at the M06-2X-D3(0)/ma-def2-TZVP and M06-2X-D3(0)/ma-def2-QZVP//M06-2X-D3(0)/ma-def2-TZVP levels, treating acetonitrile solvent molecules implicitly with the SMD model. All the energies are given with respect to the lowest energy reactant intermediate, i.e., **III**.

	M06-2X-D3(0)/ma-def2-TZVP			M06-2X-D3(0)/ma-def2-QZVP		
	ΔE	ΔH	ΔG_{298}	ΔE	ΔH	ΔG_{298}
**III***[a]	16.2	16.6	18.3	17.5	17.8	19.5
**III**	0.0	0.0	0.0	0.0	0.0	0.0
**TS II**	8.5	7.2	10.1	8.4	7.2	10.1
**IV**	−85.5	−84.6	−83.6	−85.5	−84.6	−83.6

[a] **III*** corresponds to the excited state closed-shell intermediate of the initially mixed diazonium salt and SO_3^{2-}. The corresponding broken-symmetry solution labeled as intermediate **III** is significantly lower in energy than **III***. Hence, upon mixing diazonium salt and SO_3^{2-}, one electron is spontaneously transferred from SO_3^{2-} to the diazonium salt. When SO_4^{2-} and $S_2O_5^{2-}$ are used instead of SO_3^{2-}, there is no such a broken-symmetry solution and thus a plausible sulfonylation path.

Cartesian coordinates (Å) of all optimized structures given on Fig S13.

III*

-1 1			
C	-1.18804721392200	-0.25518770112592	-0.12927068914757
C	-1.13035762715846	1.13309905494305	-0.24992868810831
C	0.06775975562913	1.78640351562369	-0.03483027591551
C	1.15859904929990	1.00504265499755	0.31738974351584
C	1.14699828501098	-0.38006764822979	0.40761246693885
C	-0.05820959606446	-1.01402051896269	0.18160949826233
C	-2.43899823843242	-0.92383833156332	-0.34870560596716
N	2.36306424954097	1.65692641622165	0.56682361832908
N	3.28581637219991	2.16856879347572	0.83749080023482
H	-2.01542012631482	1.69616149721570	-0.51124664616398
H	0.15889204552123	2.85999078098696	-0.12018802920797
H	2.04510103597348	-0.92827389377266	0.65551449976500
H	-0.12622778006228	-2.09072558662940	0.24933853006380
N	-3.43939525485720	-1.45725807103330	-0.52277908914018
S	1.92434500137164	0.97041200405083	-2.89275135691410
O	2.71425661757127	-0.07748259791098	-3.64705742094000
O	2.84124726051286	1.50738323831897	-1.79191785112993
O	1.65888716418023	2.12128739339390	-3.83997650447498

III

-1 1			
C	-1.18704813240220	-0.36545284029024	-0.15883622761731
C	-1.29461593279179	1.02167456910797	-0.23738372242235
C	-0.18059456390660	1.80033641070140	0.02737161313771
C	1.00697206234306	1.17572028303943	0.36472792900397
C	1.12852653796410	-0.20481397632354	0.44850044198046
C	0.01841085298736	-0.98322617846971	0.18449518579466
C	-2.33705716981559	-1.17642788152348	-0.44238138259688
N	2.15367237894130	2.04781506326347	0.59972627382366
N	3.22823189148386	1.68970699937981	0.86685170221303
H	-2.23659871222385	1.47944966041069	-0.50697136046748
H	-0.22267212539085	2.88009887768793	-0.03203824254262
H	2.07806843689099	-0.65538130528629	0.70681764279071
H	0.07768803097720	-2.06196497275265	0.23708047824173
N	-3.25695486042214	-1.82291733028453	-0.67256834860420
S	2.37683798616472	1.24381803222457	-2.81160953721342
O	1.43955733339960	0.49329621738674	-3.65987148373963
O	3.65972859787129	0.55893967784640	-2.59688178588049
O	2.51615738792954	2.65774569388204	-3.18990117590154

TS II

-1 1			
C	-1.14926738074788	-0.37294962852621	-0.25888251571159
C	-1.21679842292430	1.01793597606932	-0.43022434268973
C	-0.07701327756744	1.77575851164428	-0.31048684523584
C	1.15725772667567	1.13753836592091	-0.09337915190966
C	1.21116042087717	-0.24245418935746	0.17890727451032
C	0.06622254556747	-0.99432623845342	0.06332051956325
C	-2.33836970971756	-1.15639252506015	-0.36354139752290
N	2.25893061990762	2.03300552716464	0.44235325861700
N	3.29445264181645	1.65467691108521	0.78700569733590
H	-2.16965325173152	1.48912877624528	-0.63231014413962
H	-0.10543435573025	2.85324206658310	-0.40998463635656
H	2.15401637022132	-0.69732853639296	0.45433450837634
H	0.09381501790812	-2.06196941642024	0.23833070150493
N	-3.29726252161838	-1.78560828171220	-0.44603898934126

S	2.16965053468396	1.27261840888440	-2.29175905132078
O	1.22498267269931	0.51828115974914	-3.10712984811802
O	3.46985377022135	0.63407720736351	-2.11373317075778
O	2.22176659945888	2.70318290521284	-2.57965386680398

IV

-1 1

C	-0.99562697559449	-0.25499323687541	0.07226865752573
C	-1.10244911278893	1.01739881621863	-0.49185658060041
C	-0.05095161608173	1.51644641128956	-1.24083149992746
C	1.09607442156217	0.75177809601812	-1.42021810703796
C	1.19962665219204	-0.51358279910095	-0.85793338123996
C	0.15210852079928	-1.02468746078354	-0.10796491046011
C	-2.07788104738604	-0.77121934282345	0.85941273118449
N	1.06599782027093	1.98867404430920	1.86983084149262
N	1.80499837593017	1.19309956853247	1.86513009479197
H	-1.99793006417561	1.60437413159116	-0.33643670663392
H	-0.11447019276524	2.50646394126509	-1.67374892955764
H	2.10426438389089	-1.09070465990795	-0.99456683143219
H	0.22230366830303	-2.00644268326199	0.34141791523601
N	-2.94323946251985	-1.18269373123131	1.49170056322499
S	2.44275200495376	1.40812130679773	-2.40475175451021
O	2.05698544991415	1.13268790520468	-3.78266196894905
O	3.62390607169516	0.67567647886520	-1.97433163802308
O	2.48184110180026	2.82802021389271	-2.08733049508369

SPECTROSCOPIC DATA

^{1}H NMR spectrum of Flutamide-derived sulfonyl chloride 4

CDCl$_3$, 23°C

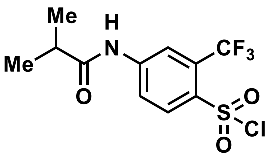

4

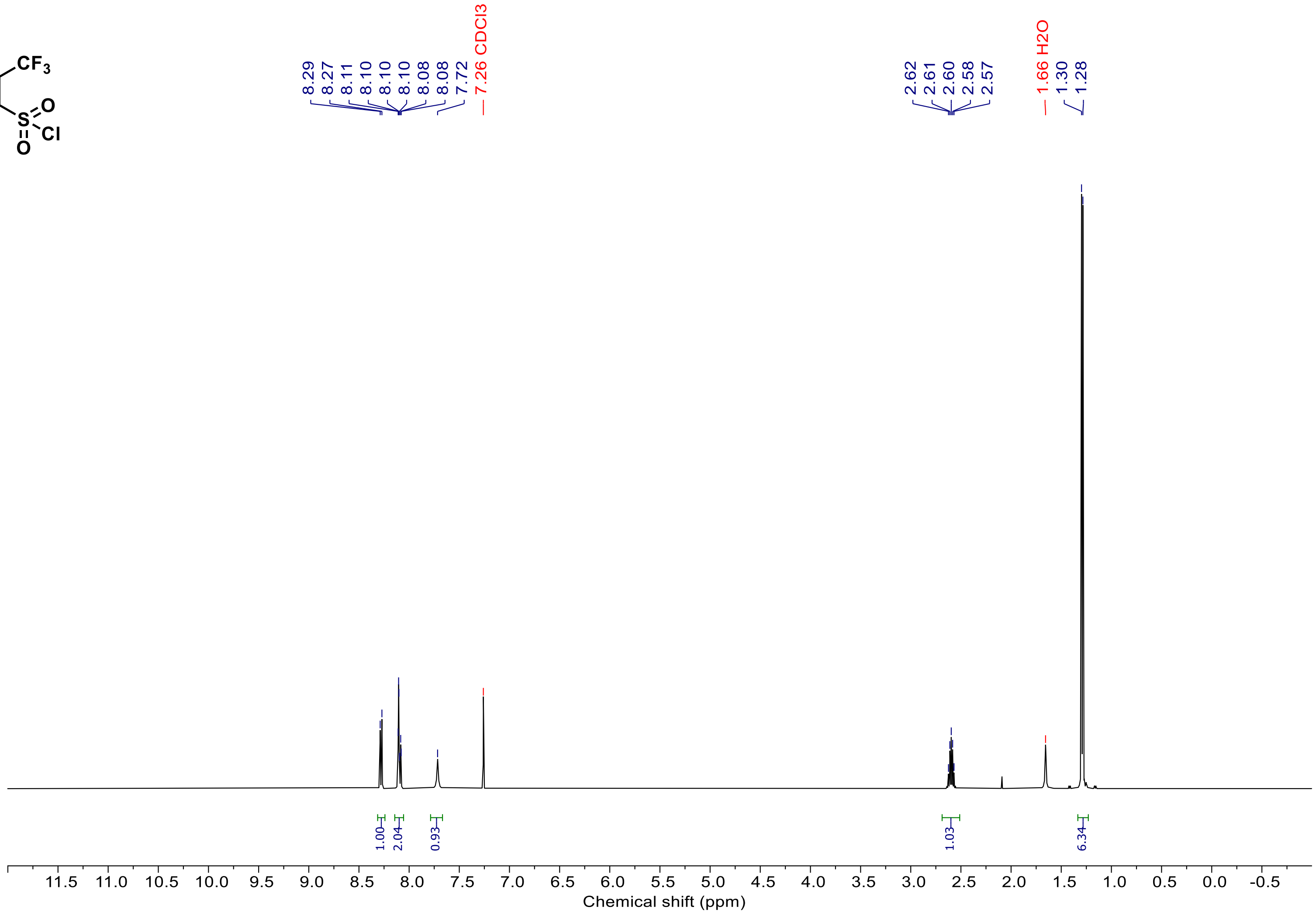

^{13}C NMR spectrum of Flutamide-derived sulfonyl chloride 4

CDCl$_3$, 23°C

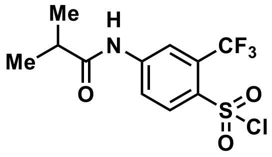

4

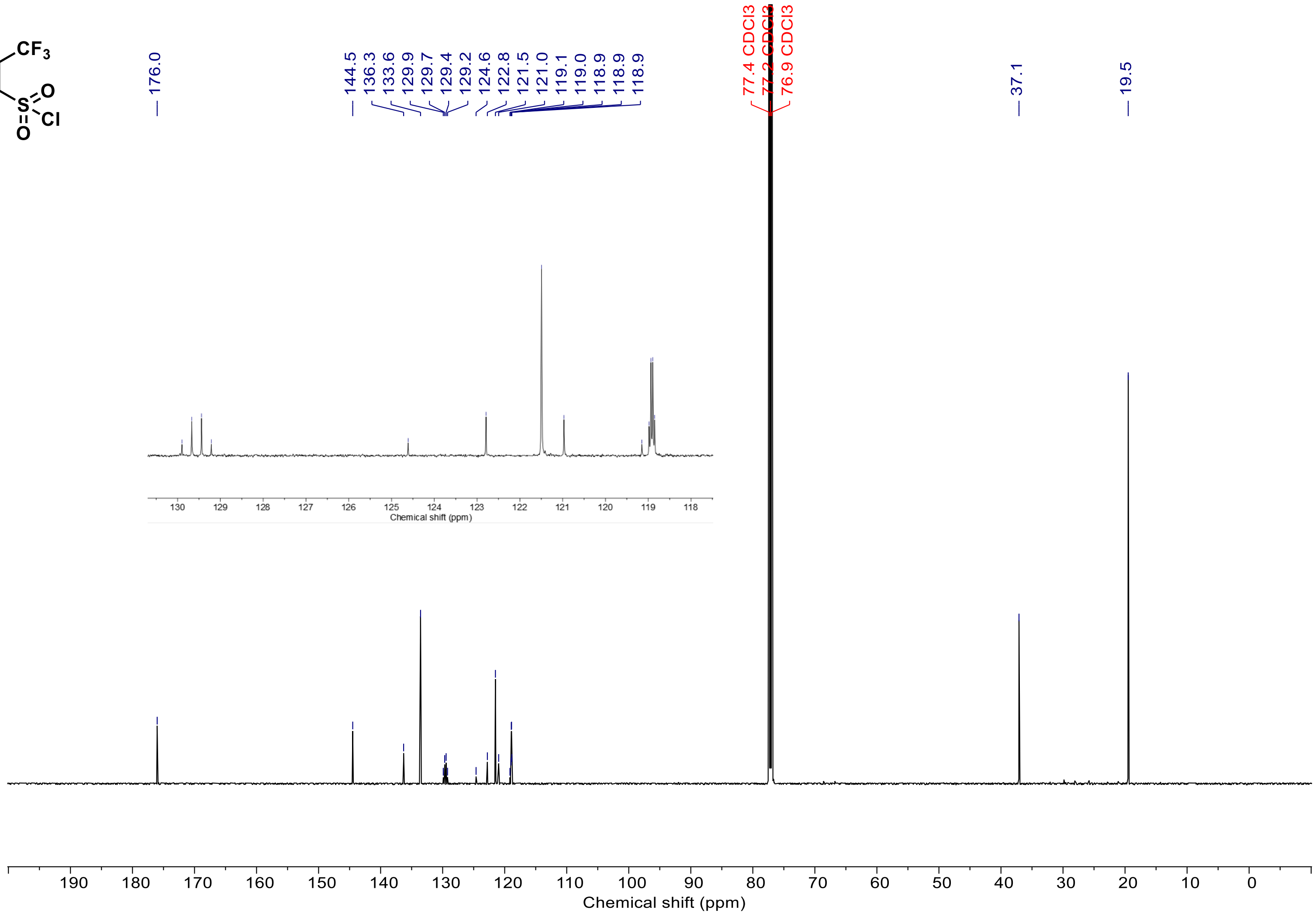

^{19}F NMR spectrum of Flutamide-derived sulfonyl chloride 4

$CDCl_3$, 23°C

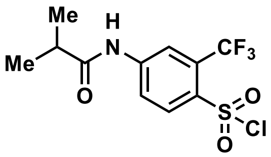

4

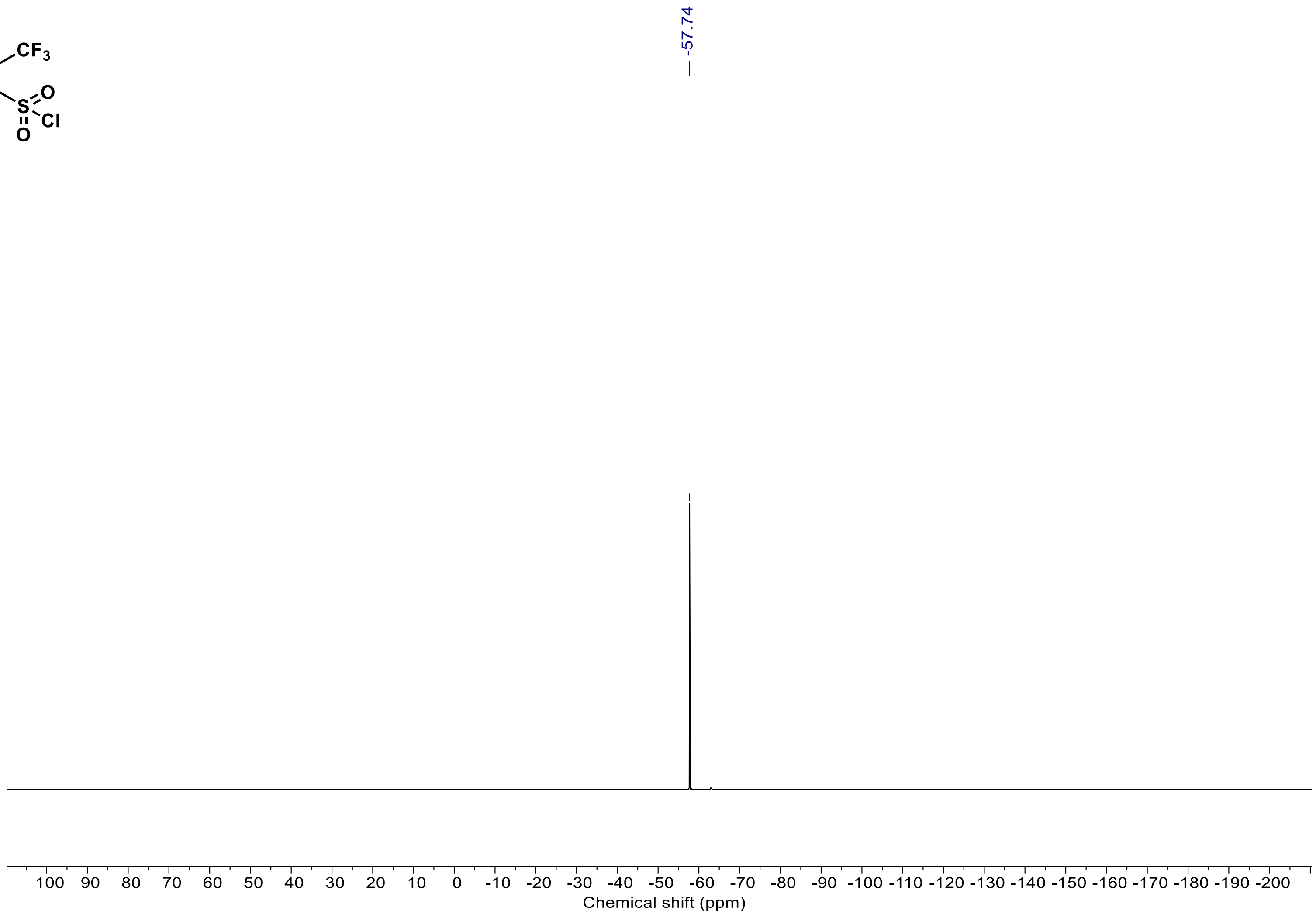

^{1}H NMR spectrum of Flutamide-derived primary sulfonamide 5

MeCN-d_3, 23°C

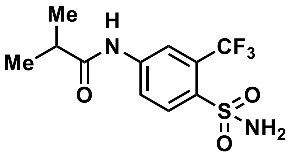

5

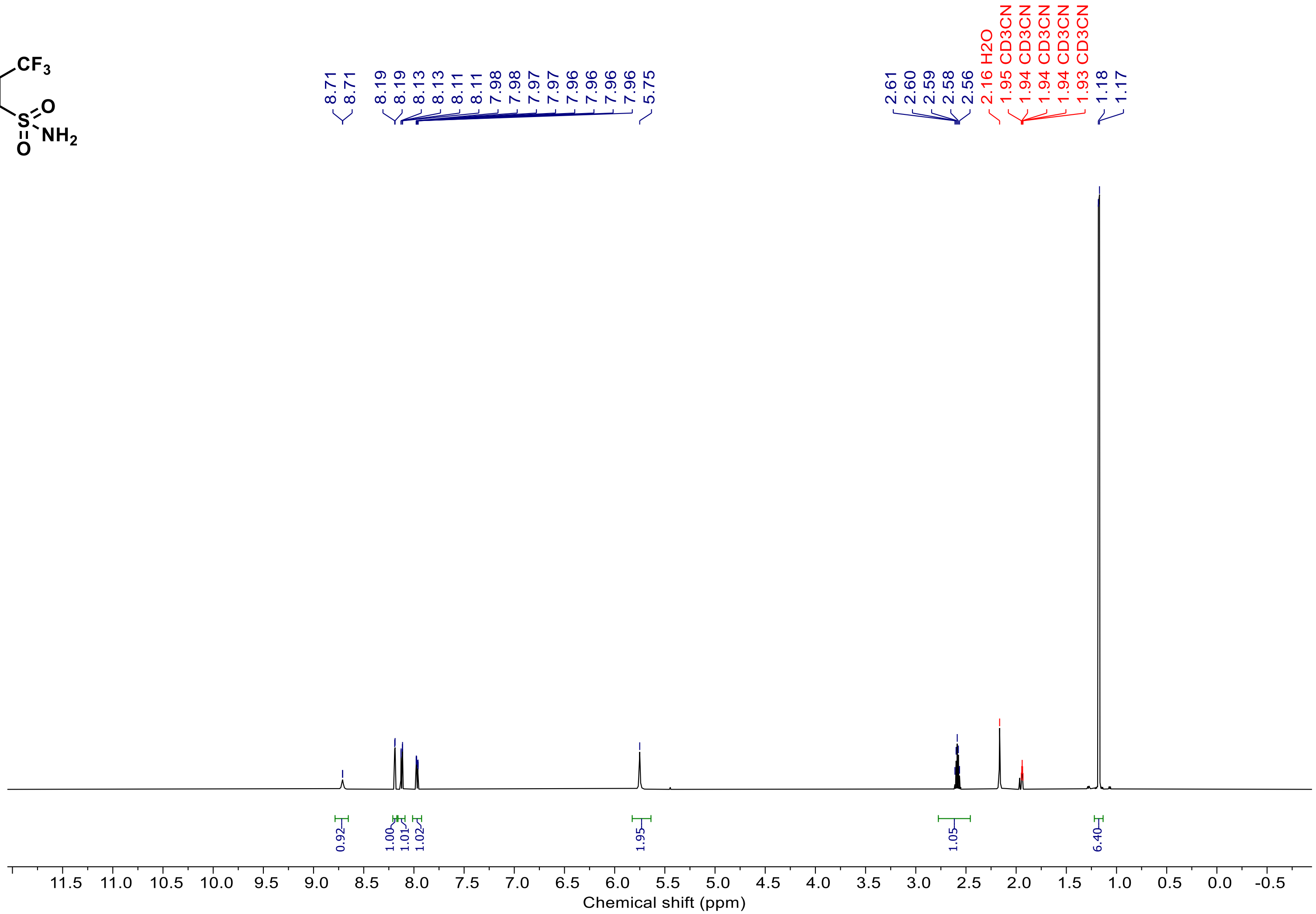

^{13}C NMR spectrum of Flutamide-derived primary sulfonamide 5

MeCN-d_3, 23°C

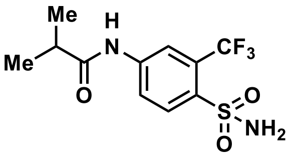

5

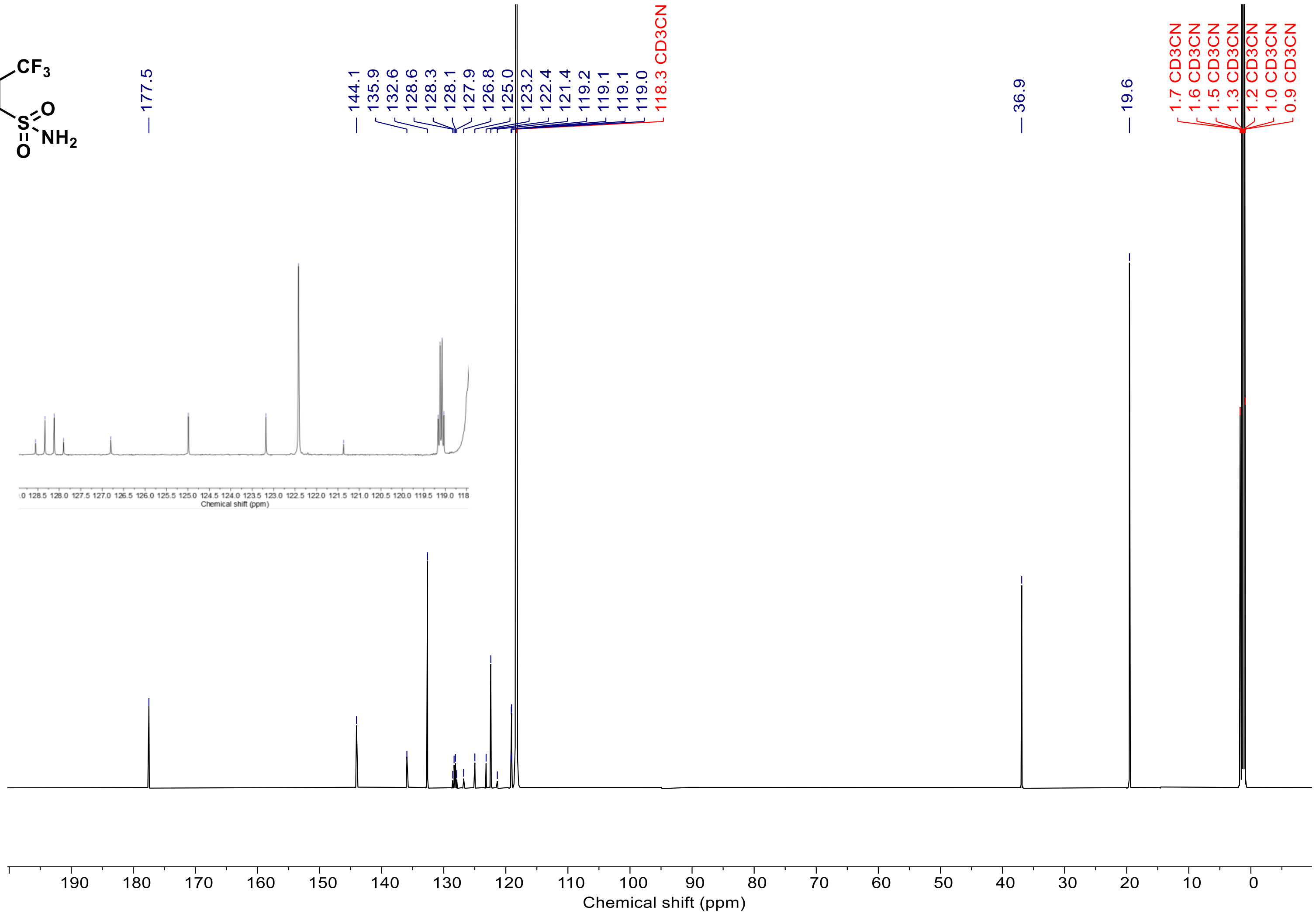

^{19}F NMR spectrum of Flutamide-derived primary sulfonamide 5

MeCN-*d*$_3$, 23°C

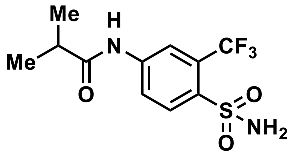

5

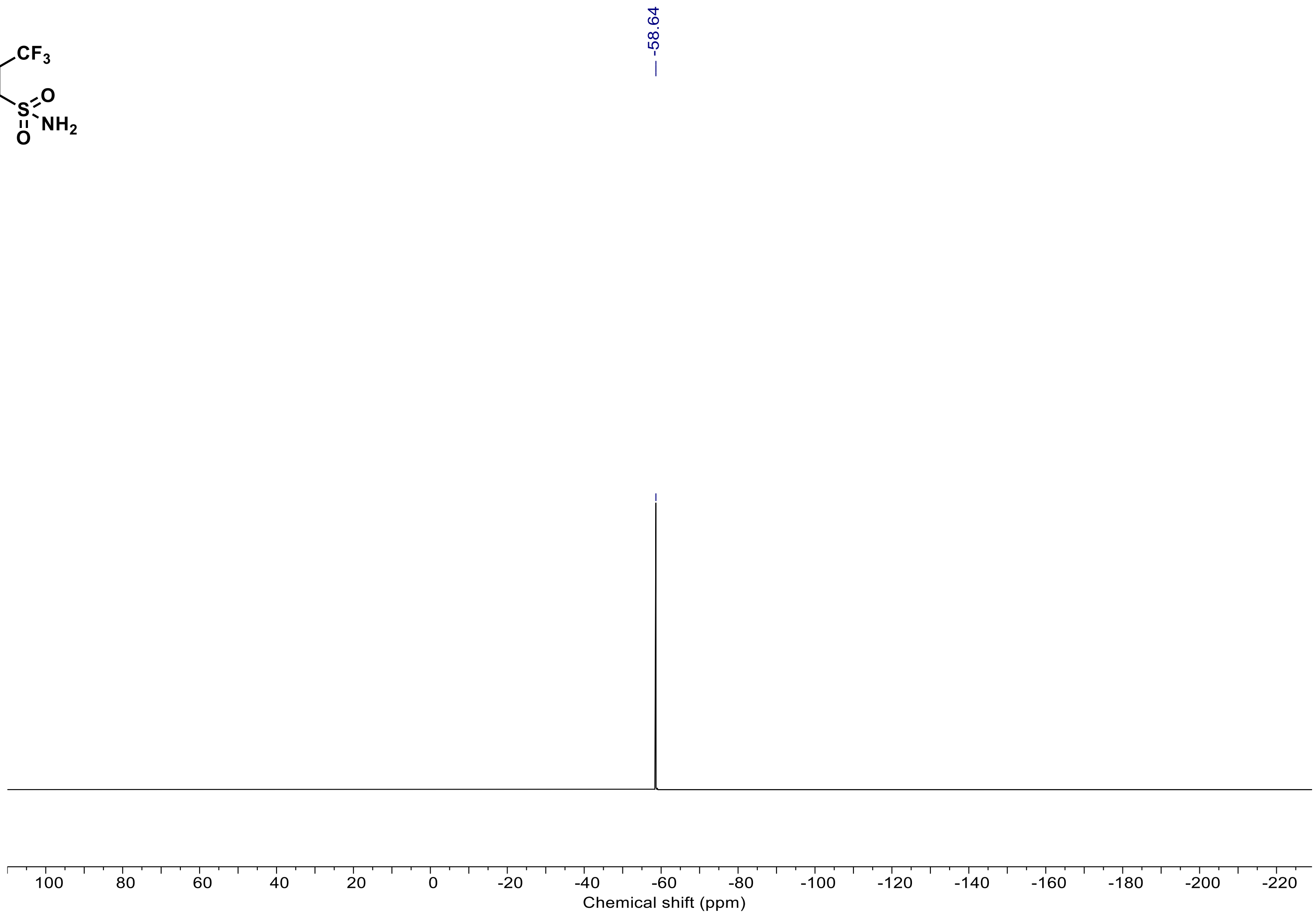

^{1}H NMR spectrum of Flutamide-derived secondary sulfonamide 6

MeCN-d_3, 23°C

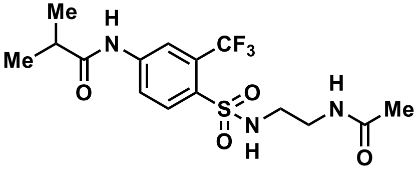

6

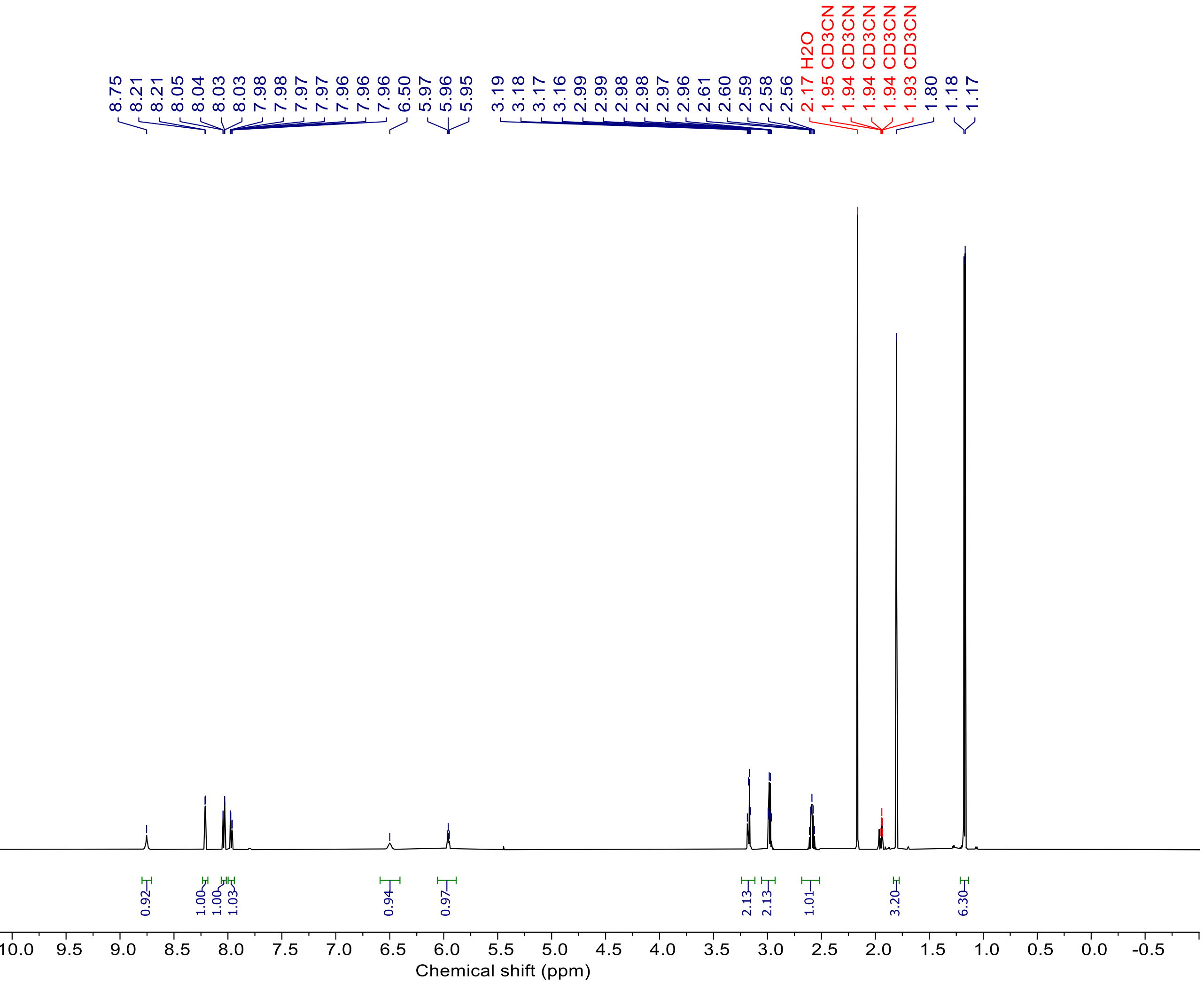

^{13}C NMR spectrum of Flutamide-derived secondary sulfonamide 6

MeCN-d_3, 23°C

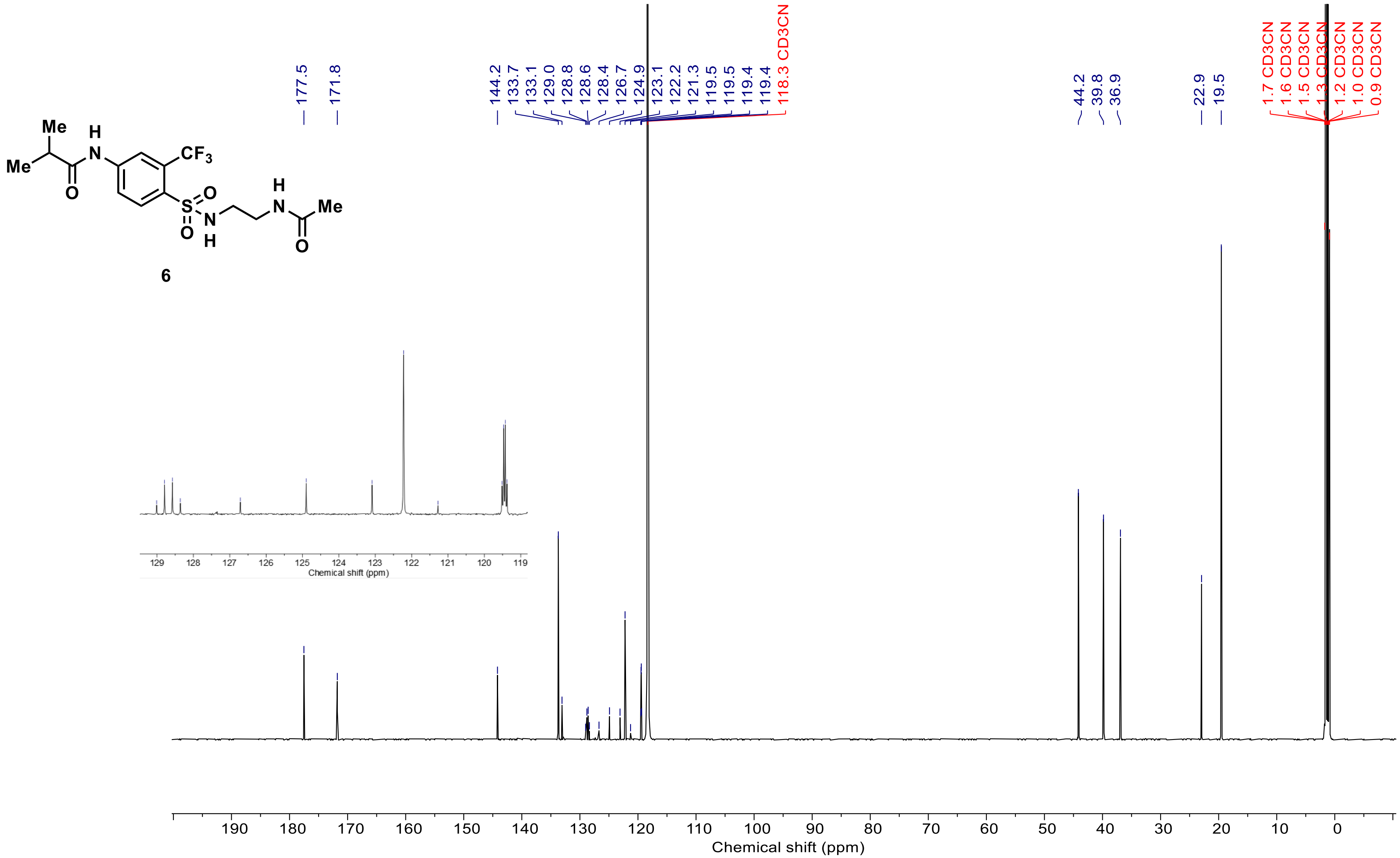

^{19}F NMR spectrum of Flutamide-derived secondary sulfonamide 6

MeCN-d_3, 23°C

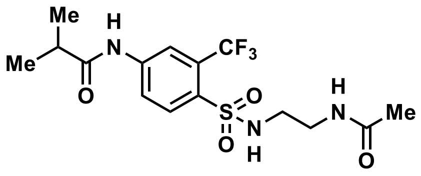

6

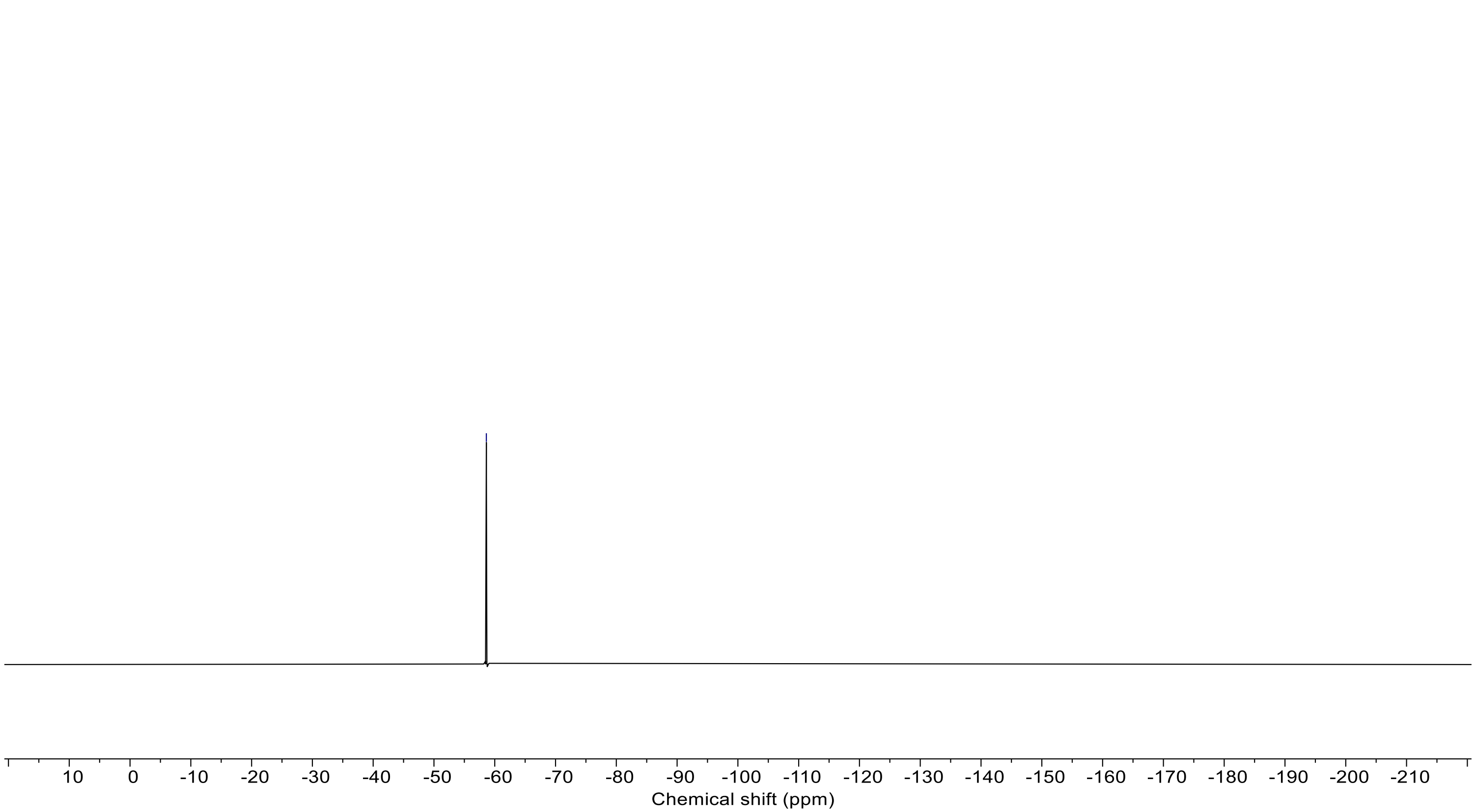

^{1}H NMR specrum of Flutamide-derived tertiary sulfonamide 7

CDCl$_3$, 23°C

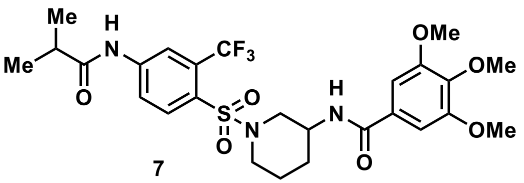

7

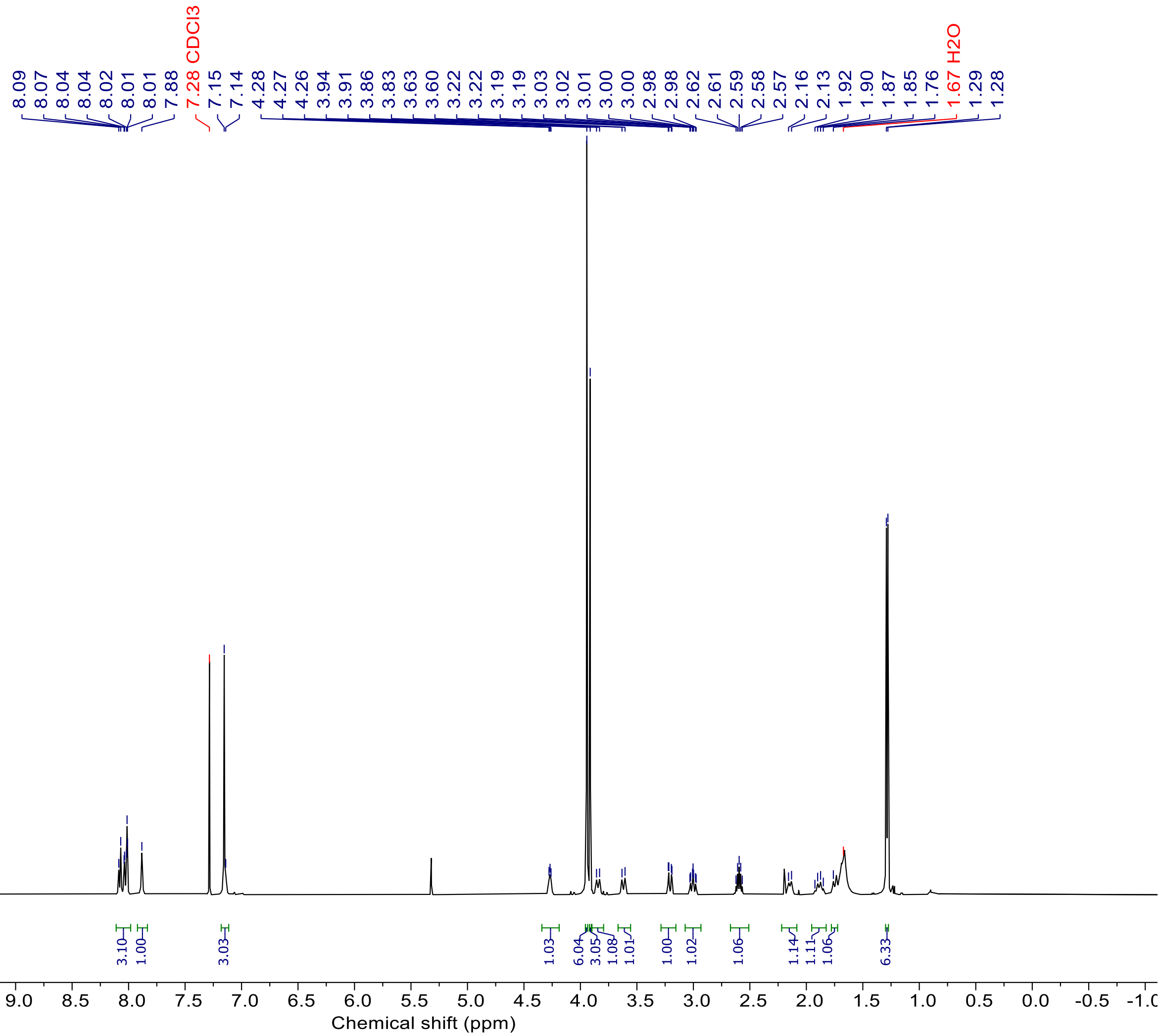

^{13}C NMR spectrum of Flutamide-derived tertiary sulfonamide 7

$CDCl_3$, 23°C

7

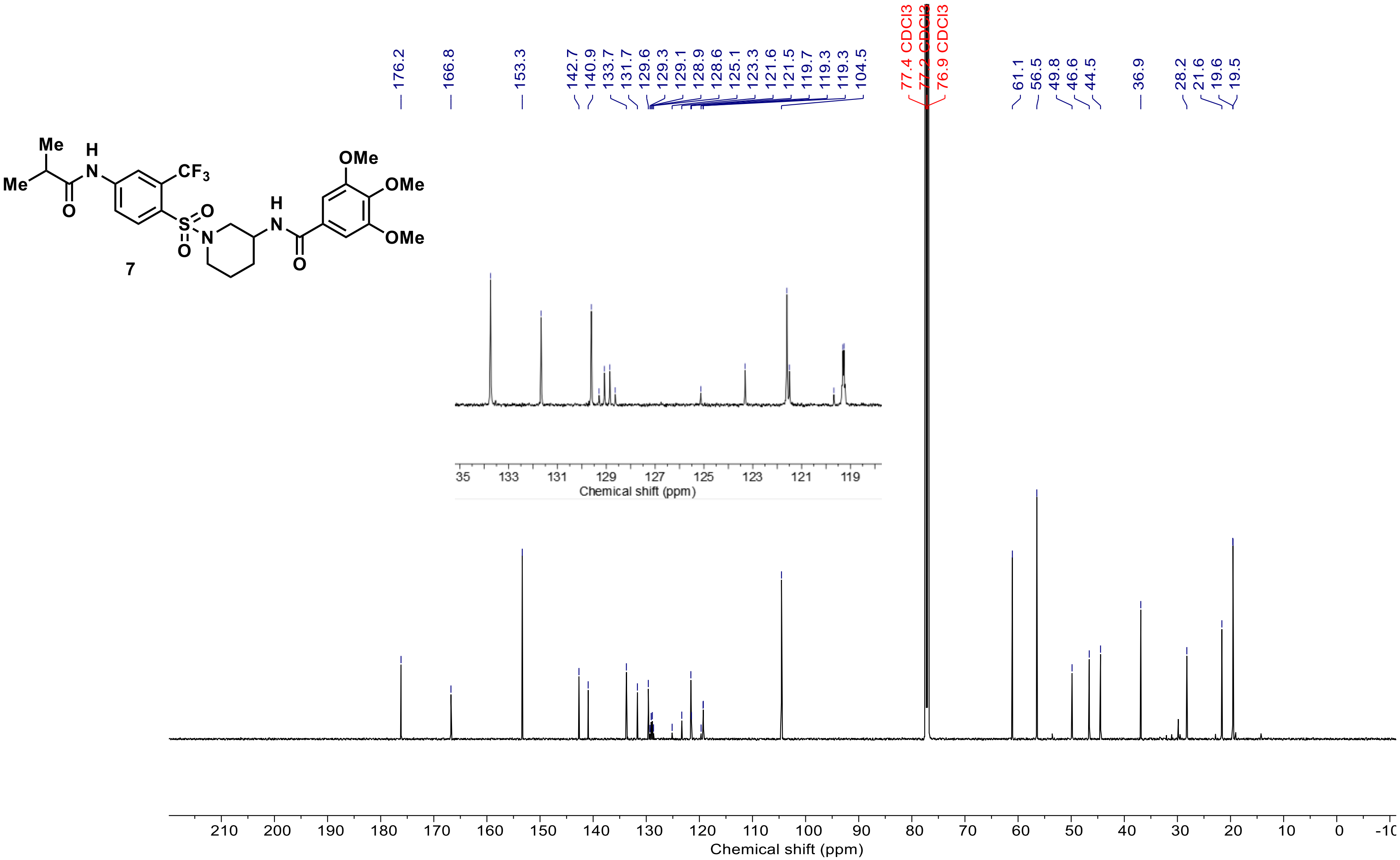

^{19}F NMR spectrum of Flutamide-derived tertiary sulfonamide 7

$CDCl_3$, 23°C

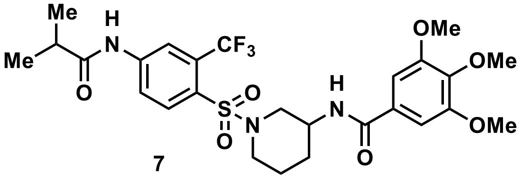

7

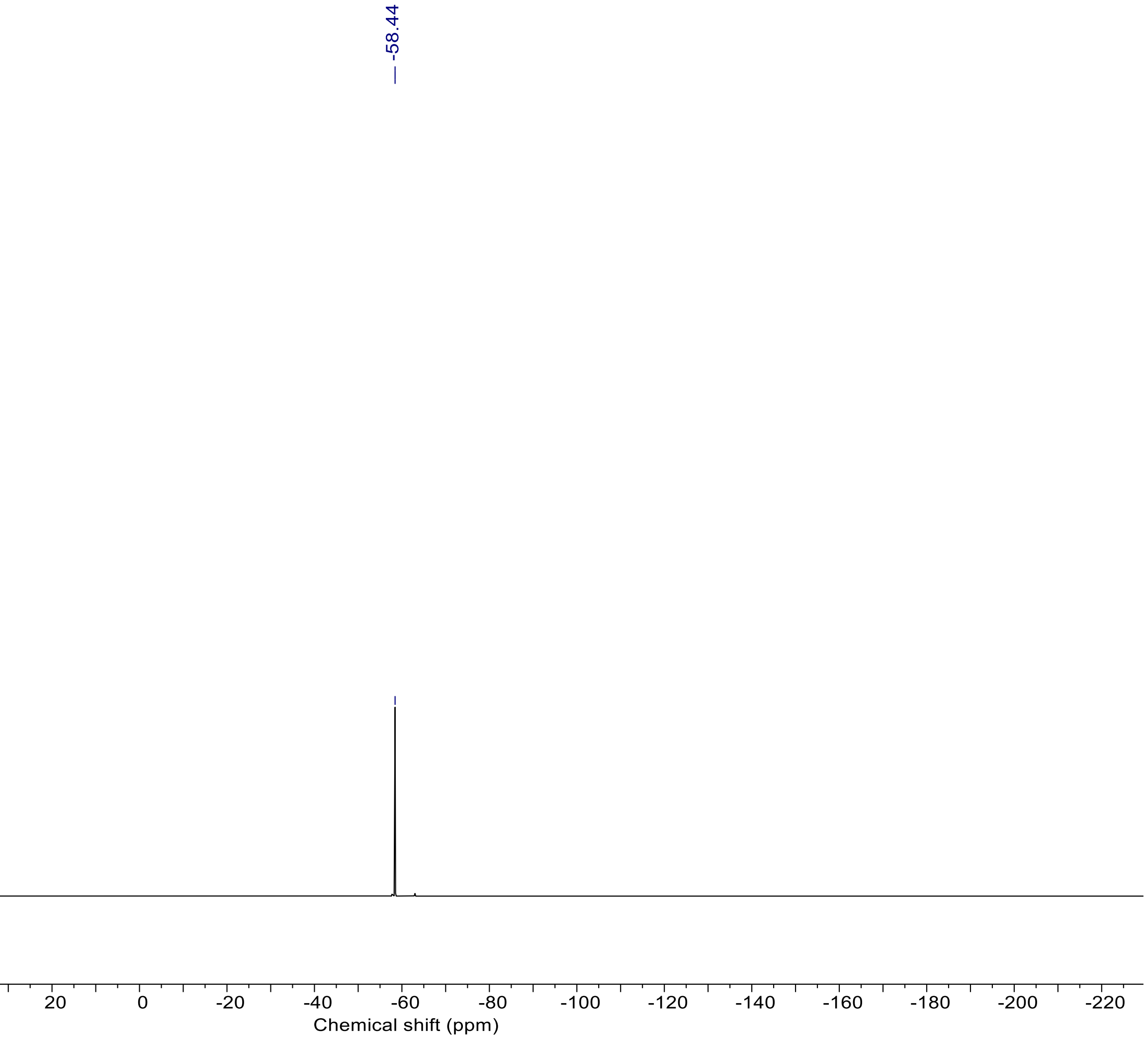

^{1}H NMR spectrum of Flutamide-derived sulfonyl fluoride 8

$CDCl_3$, 23°C

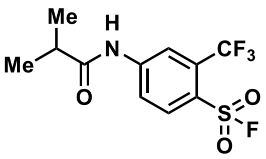

8

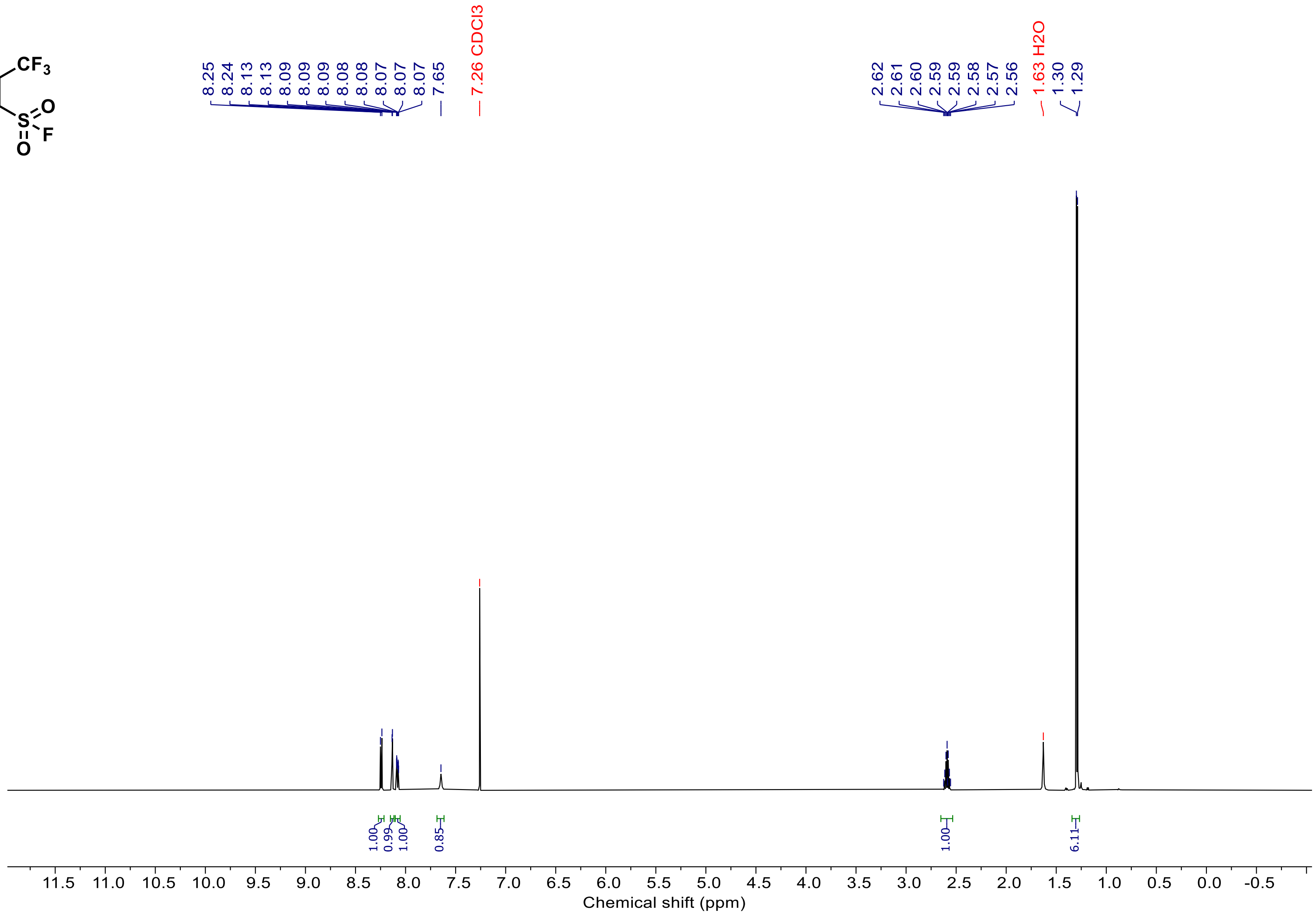

^{13}C NMR spectrum of Flutamide-derived sulfonyl fluoride 8

CDCl$_3$, 23°C

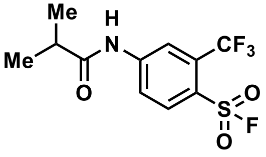

8

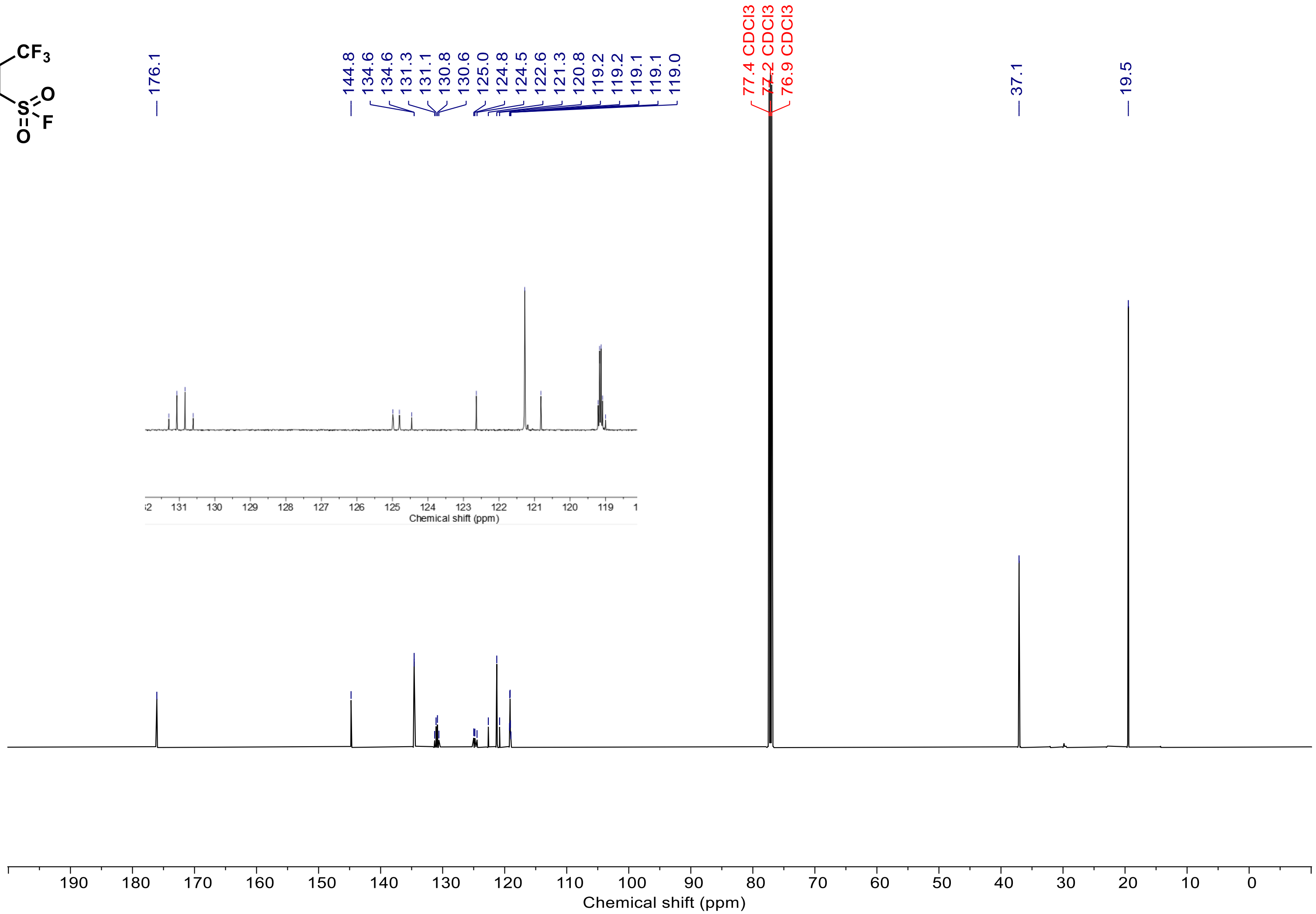

^{19}F NMR spectrum of Flutamide-derived sulfonyl fluoride 8

$CDCl_3$, 23°C

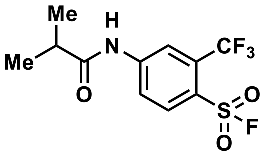

8

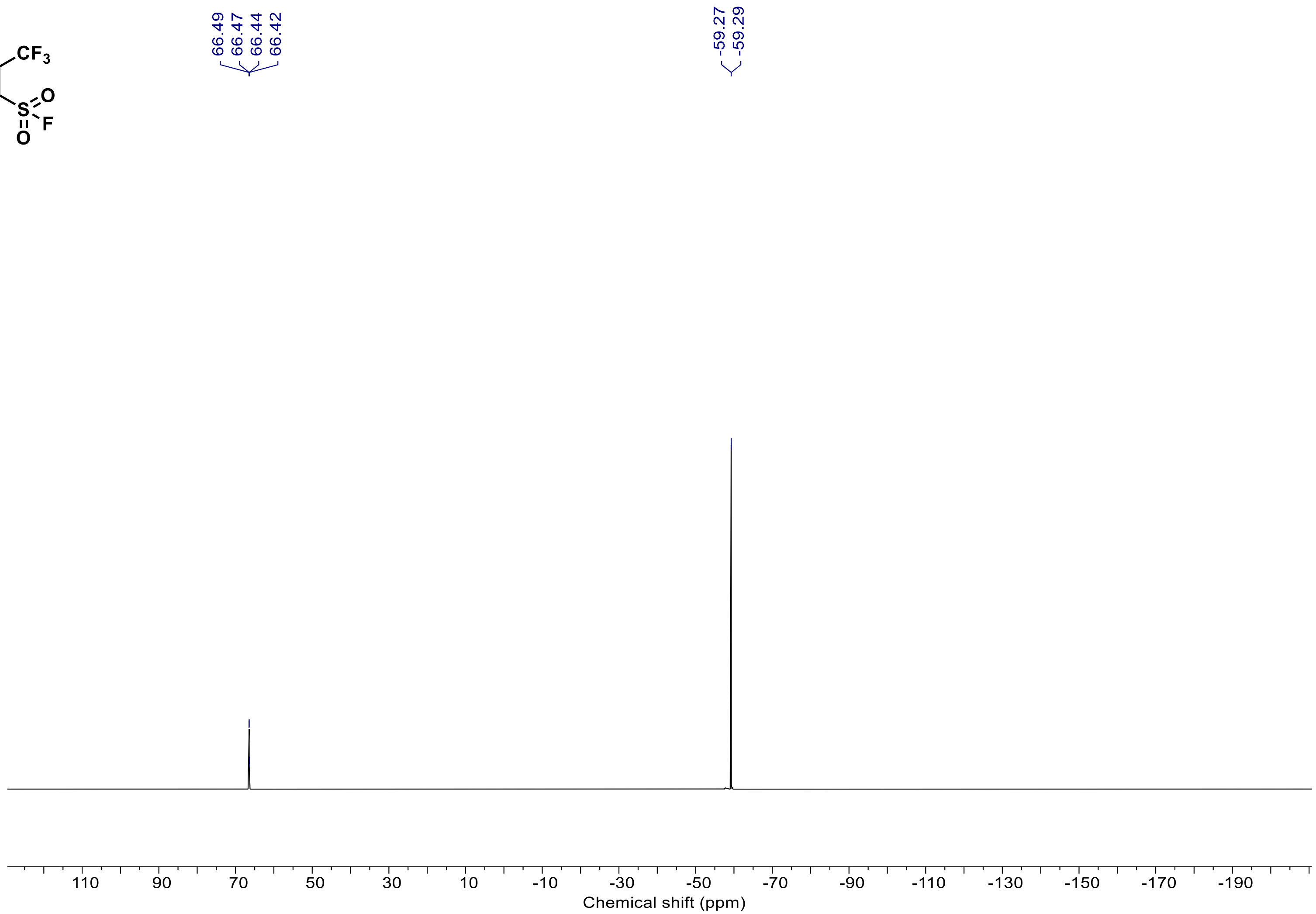

^{1}H NMR spectrum of Flutamide-derived sulfonic acid 9

DMSO-d_6, 23°C

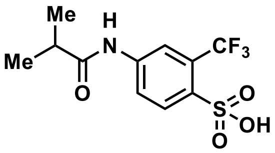

9

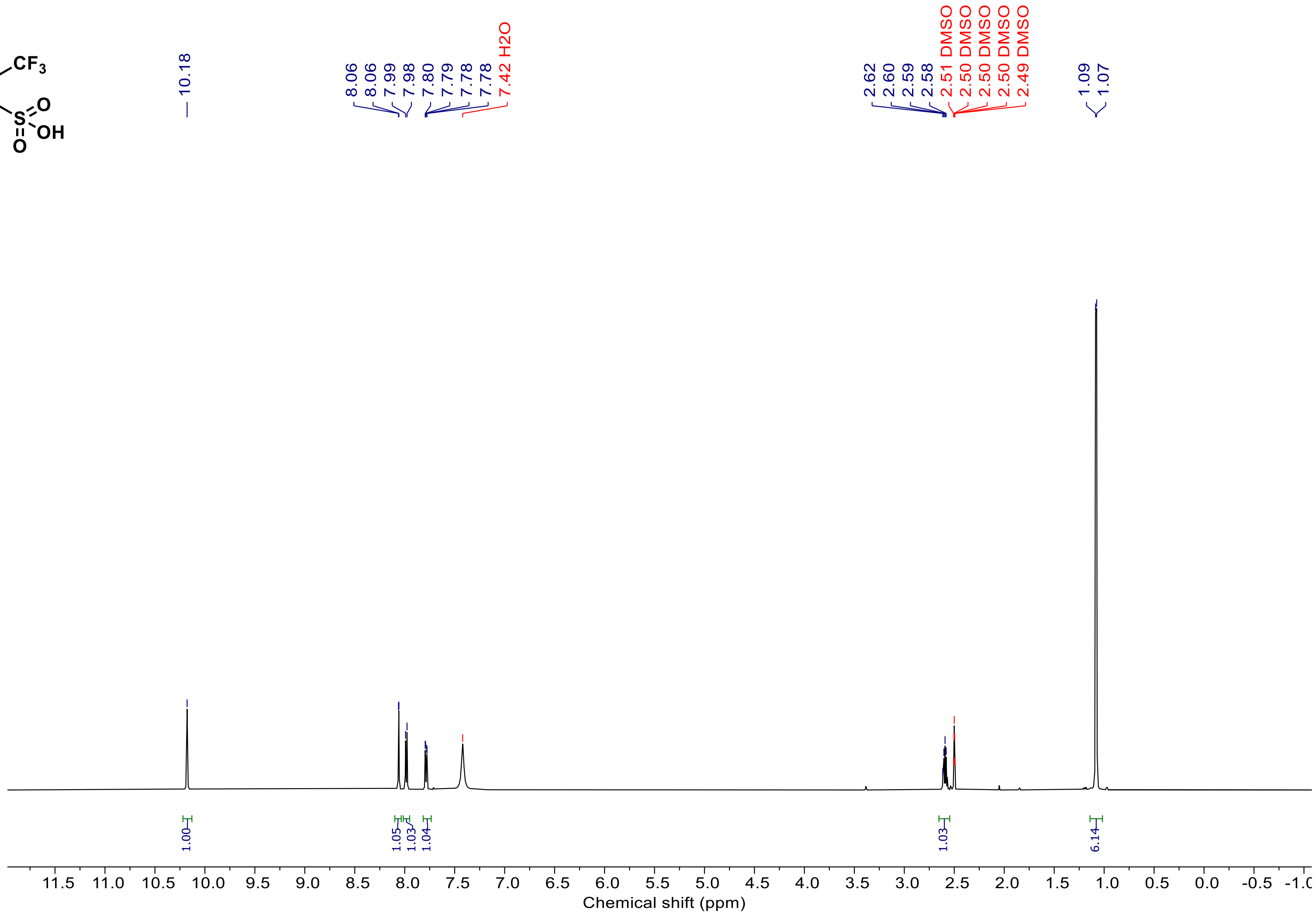

^{13}C NMR spectrum of Flutamide-derived sulfonic acid 9

DMSO-d_6, 23°C

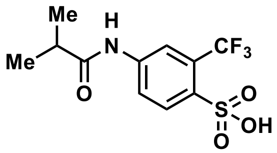

9

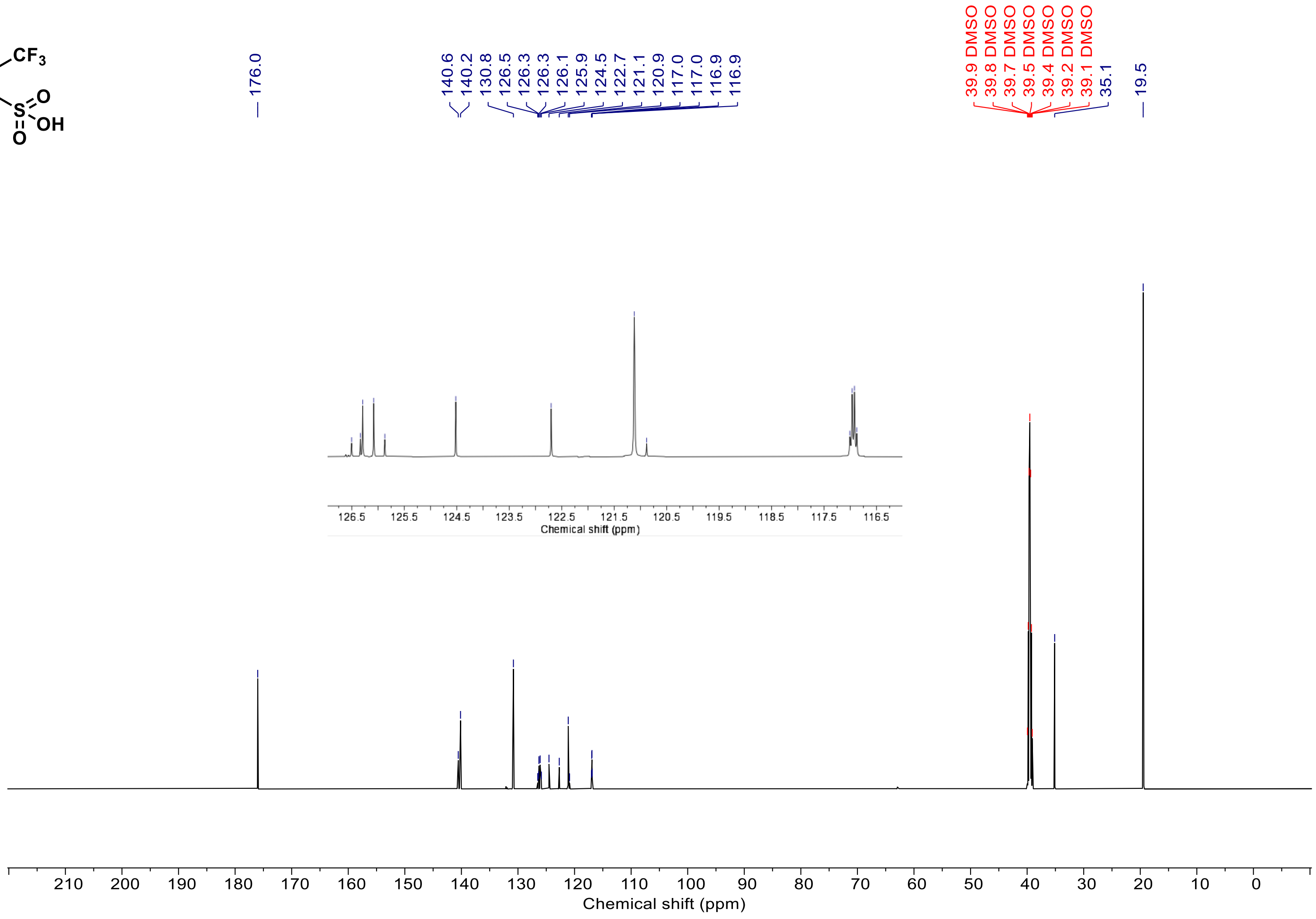

^{19}F NMR spectrum of Flutamide-derived sulfonic acid 9

DMSO-d_6, 23°C

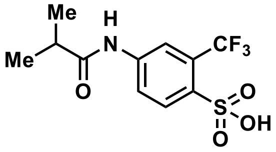

9

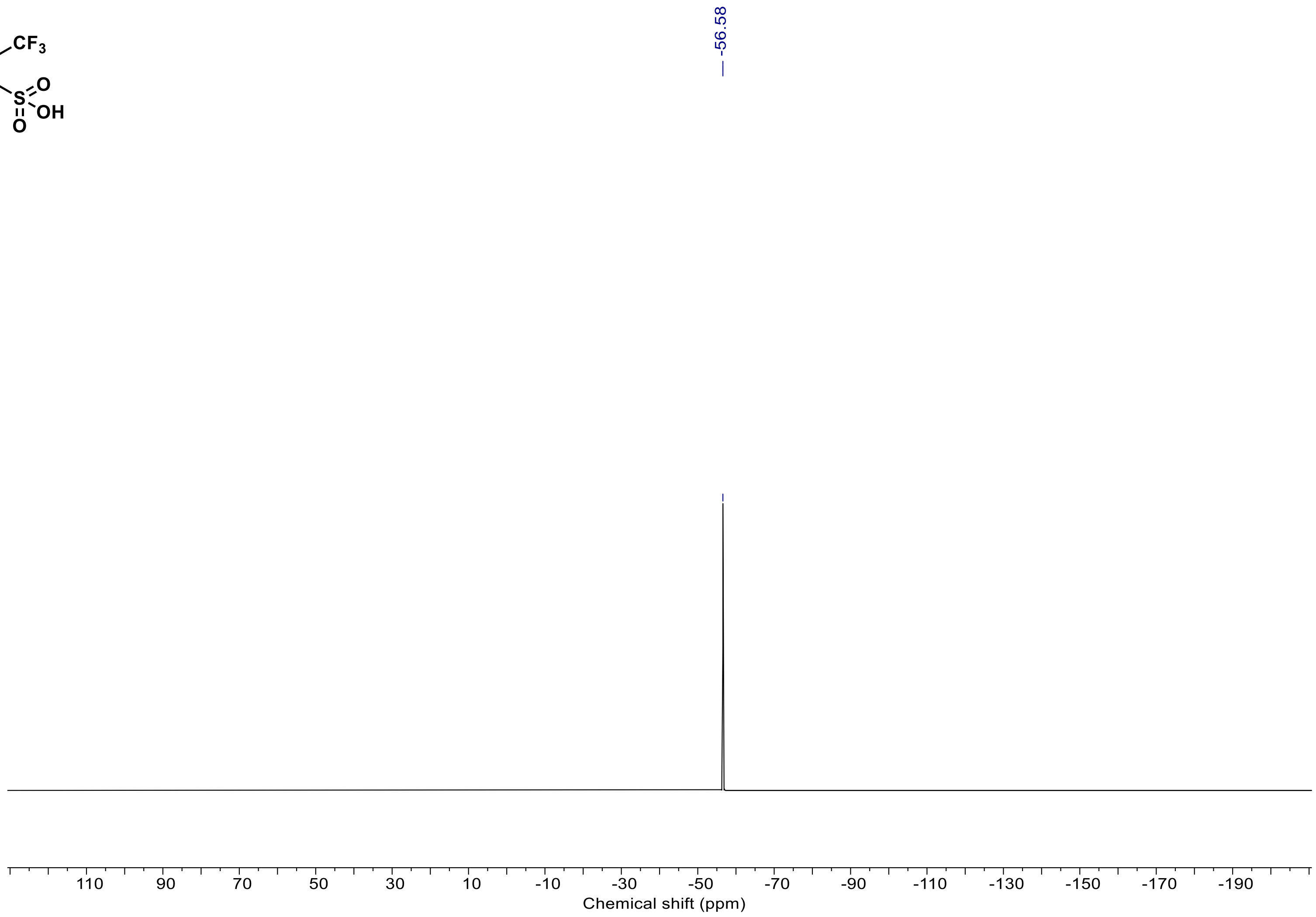

^{1}H NMR spectrum of 4-(pyrrolidin-1-ylsulfonyl)benzonitrile (10)

$CDCl_3$, 23°C

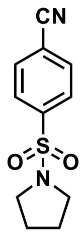

10

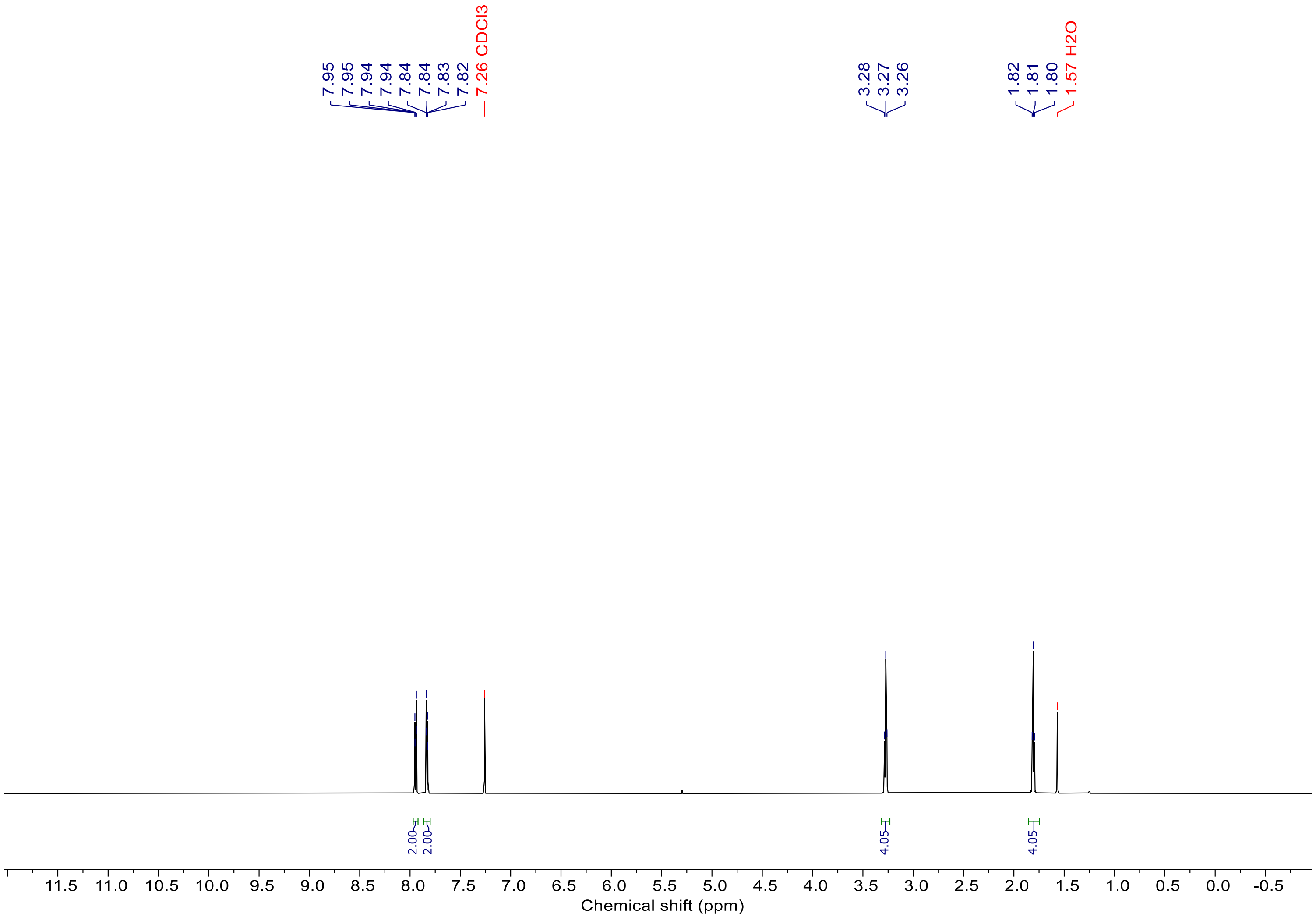

^{13}C NMR spectrum of 4-(pyrrolidin-1-ylsulfonyl)benzonitrile (10)

$CDCl_3$, 23°C

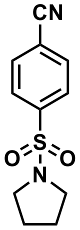

10

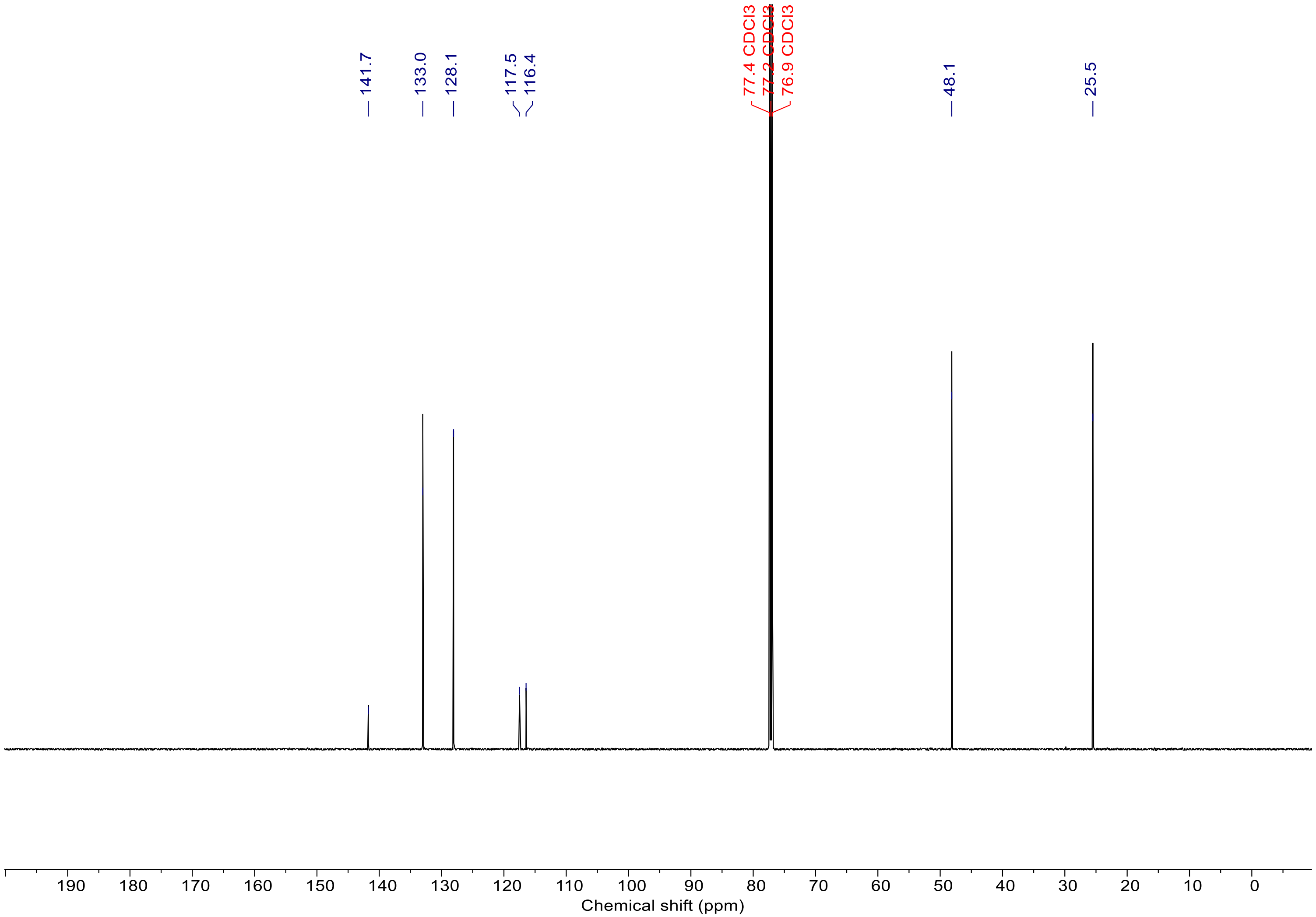

^{1}H NMR spectrum of 1-((4-phenoxyphenyl)sulfonyl)pyrrolidine (11)

CDCl$_3$, 23°C

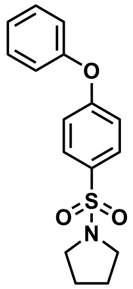

11

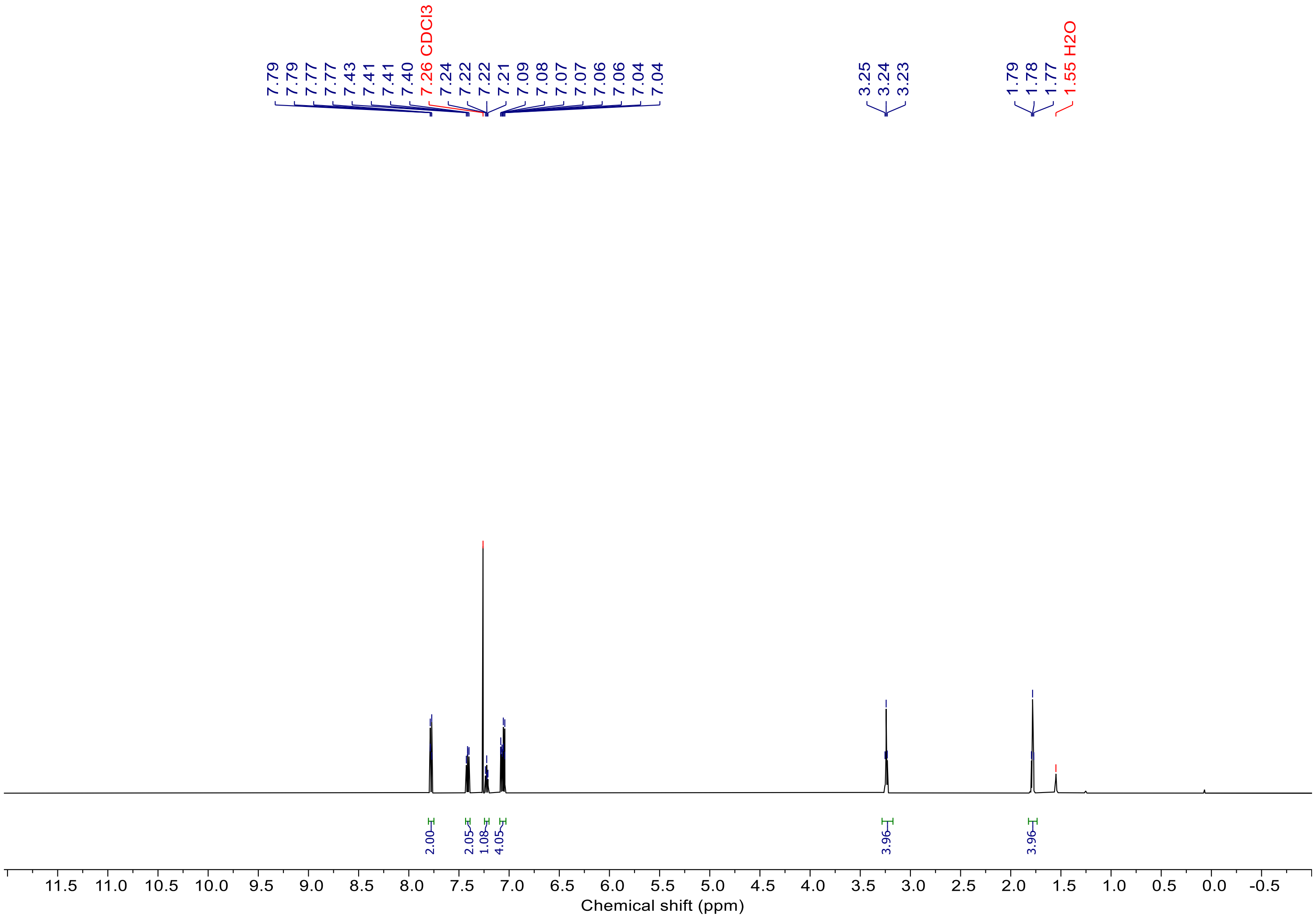

^{13}C NMR spectrum of 1-((4-phenoxyphenyl)sulfonyl)pyrrolidine (11)

$CDCl_3$, 23°C

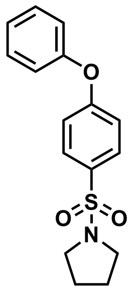

11

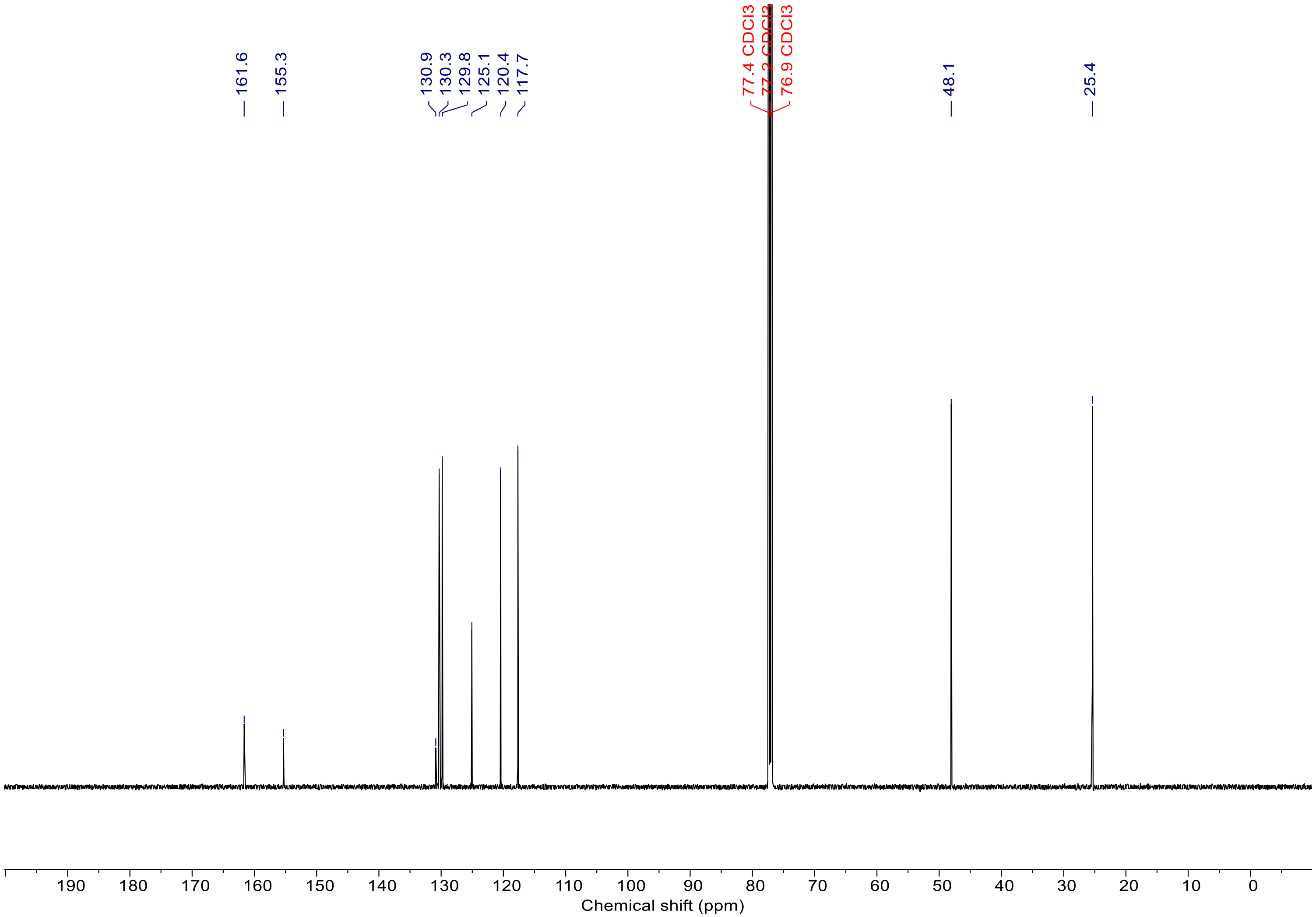

^{1}H NMR spectrum of 1-((4-methoxyphenyl)sulfonyl)pyrrolidine (12)

$CDCl_3$, 23°C

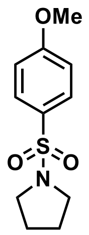

12

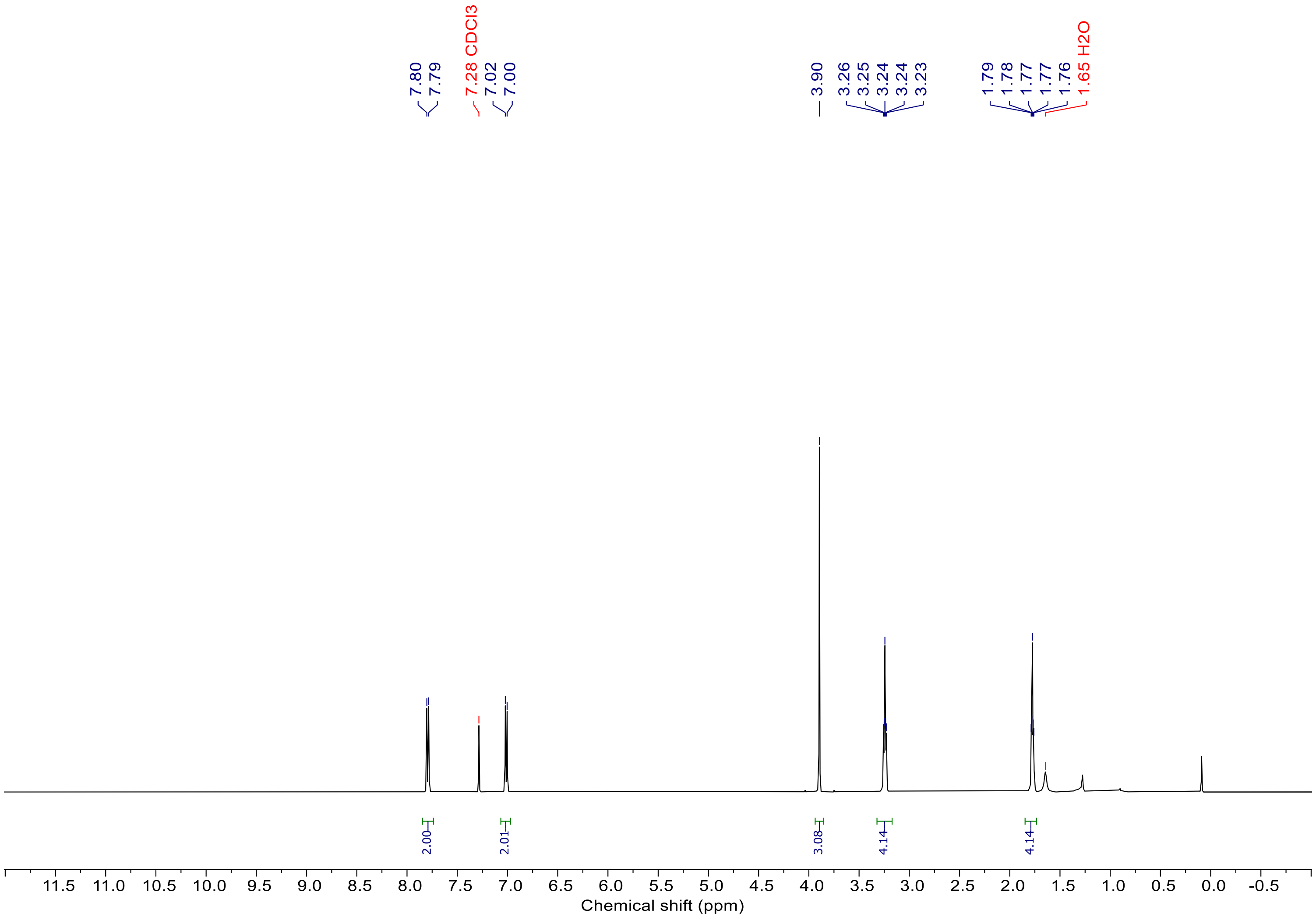

^{13}C NMR spectrum of 1-((4-methoxyphenyl)sulfonyl)pyrrolidine (12)

$CDCl_3$, 23°C

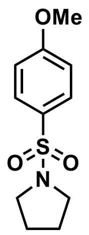

12

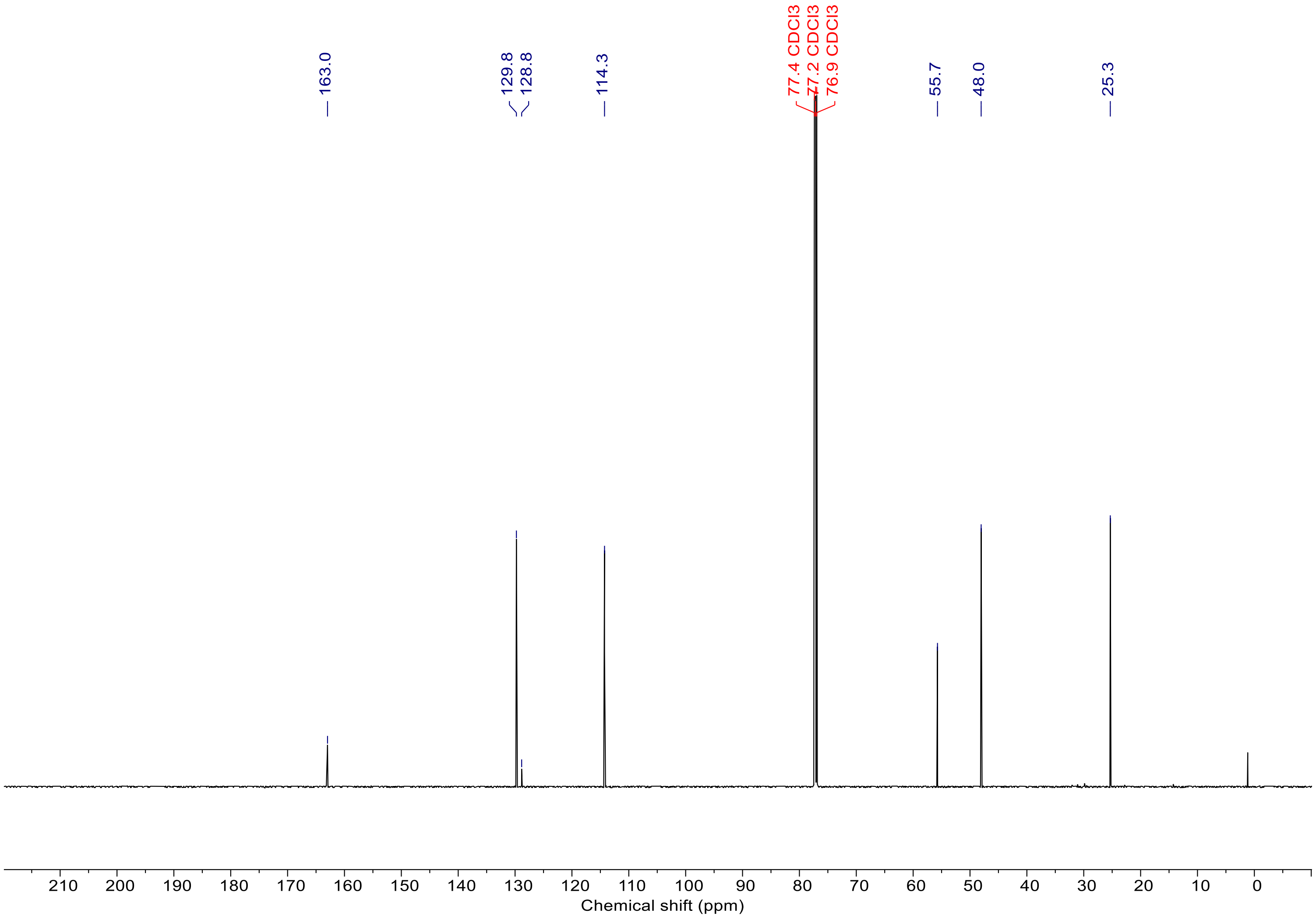

^{1}H NMR spectrum of 2-chloro-3-(pyrrolidin-1-ylsulfonyl)pyridine (13)

CDCl3, 23°C

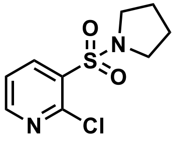

13

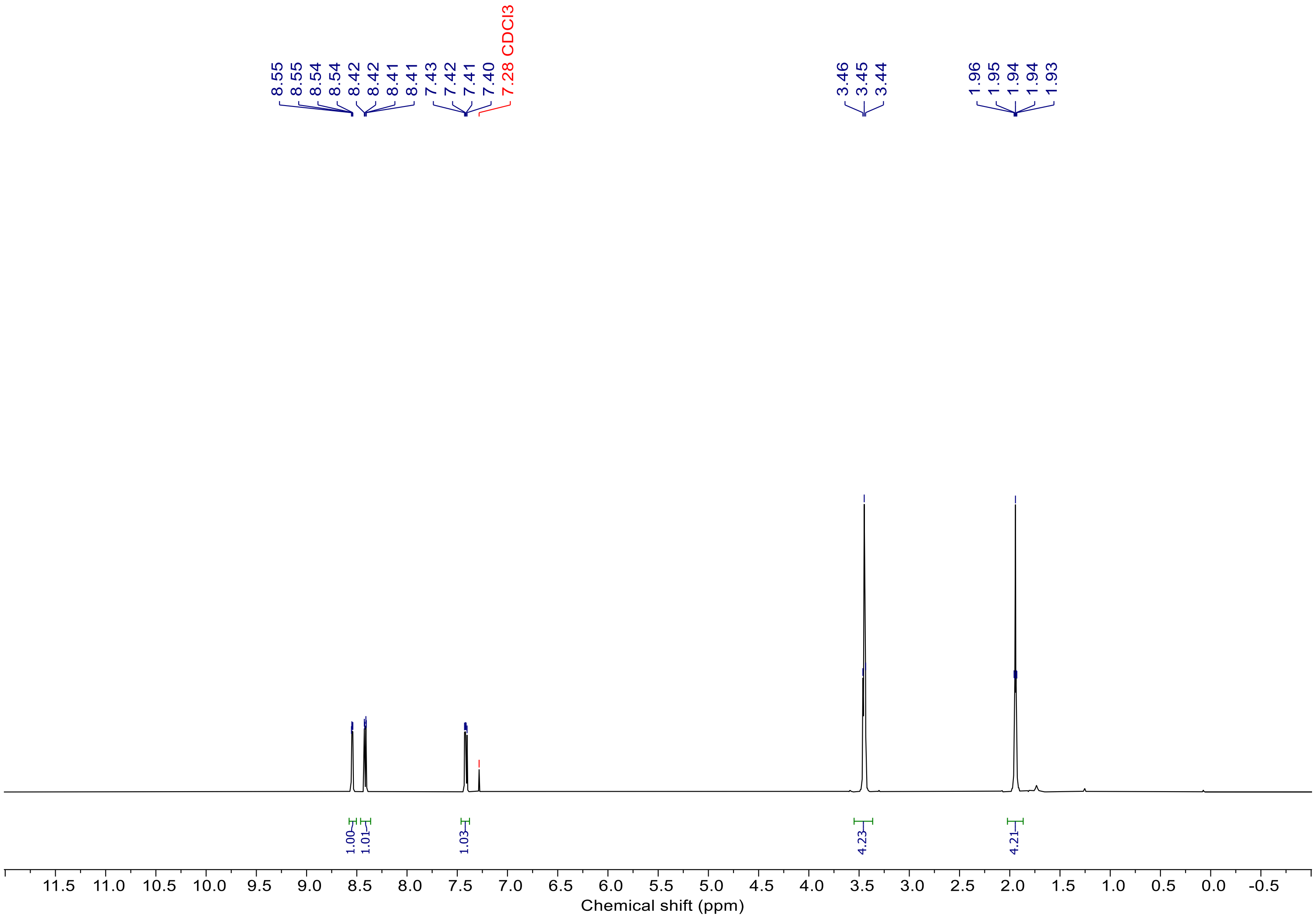

^{13}C NMR spectrum of 2-chloro-3-(pyrrolidin-1-ylsulfonyl)pyridine (13)

$CDCl_3$, 23°C

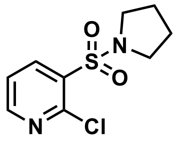

13

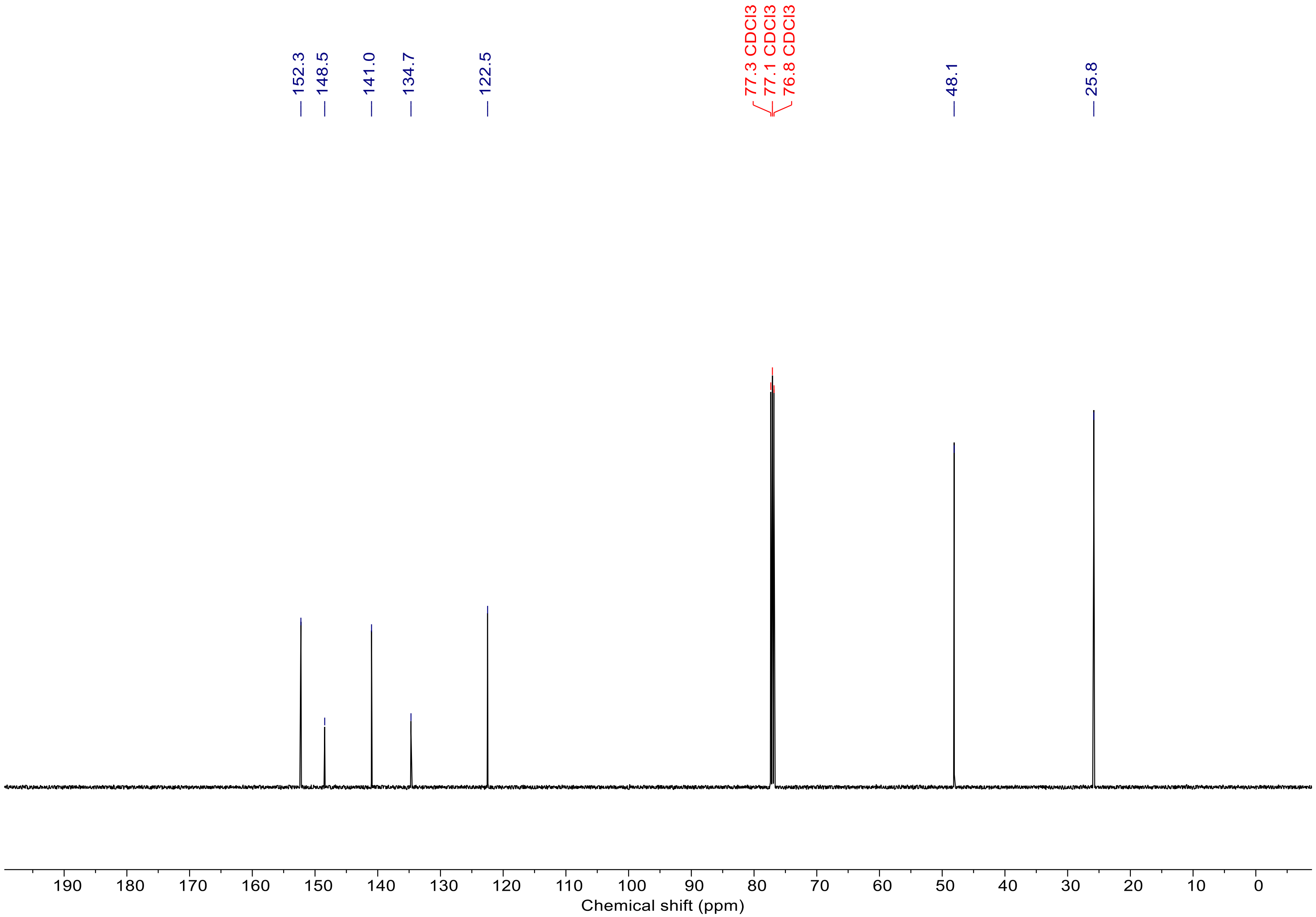

^{1}H NMR spectrum of 1-((4'-chloro-[1,1'-biphenyl]-2-yl)sulfonyl)pyrrolidine (14)

$CDCl_3$, 23°C

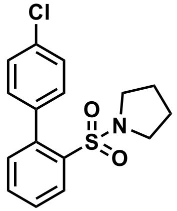

14

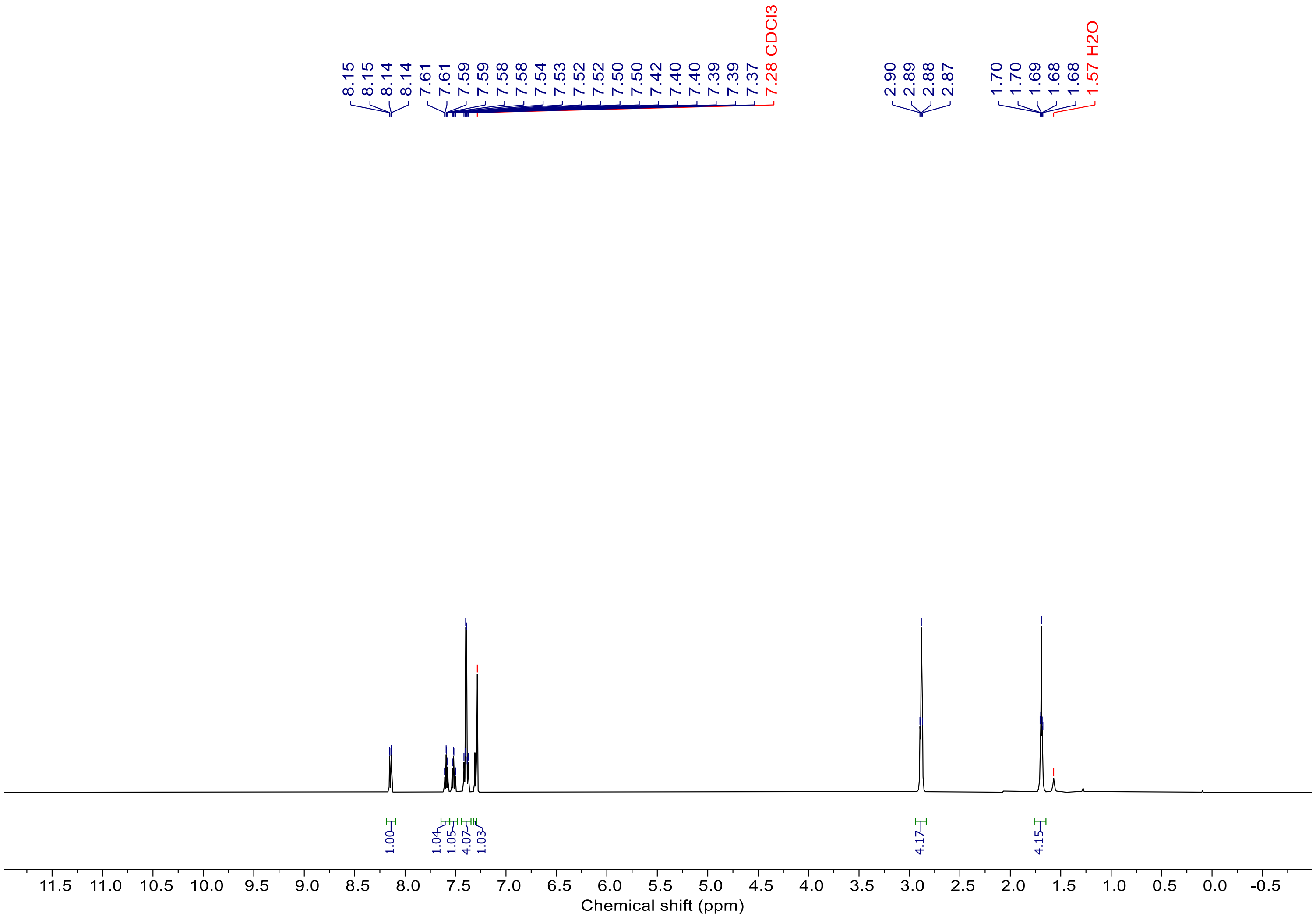

^{13}C NMR spectrum of 1-((4'-chloro-[1,1'-biphenyl]-2-yl)sulfonyl)pyrrolidine (14)

$CDCl_3$, 23°C

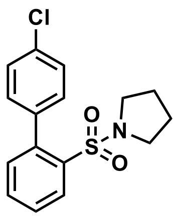

14

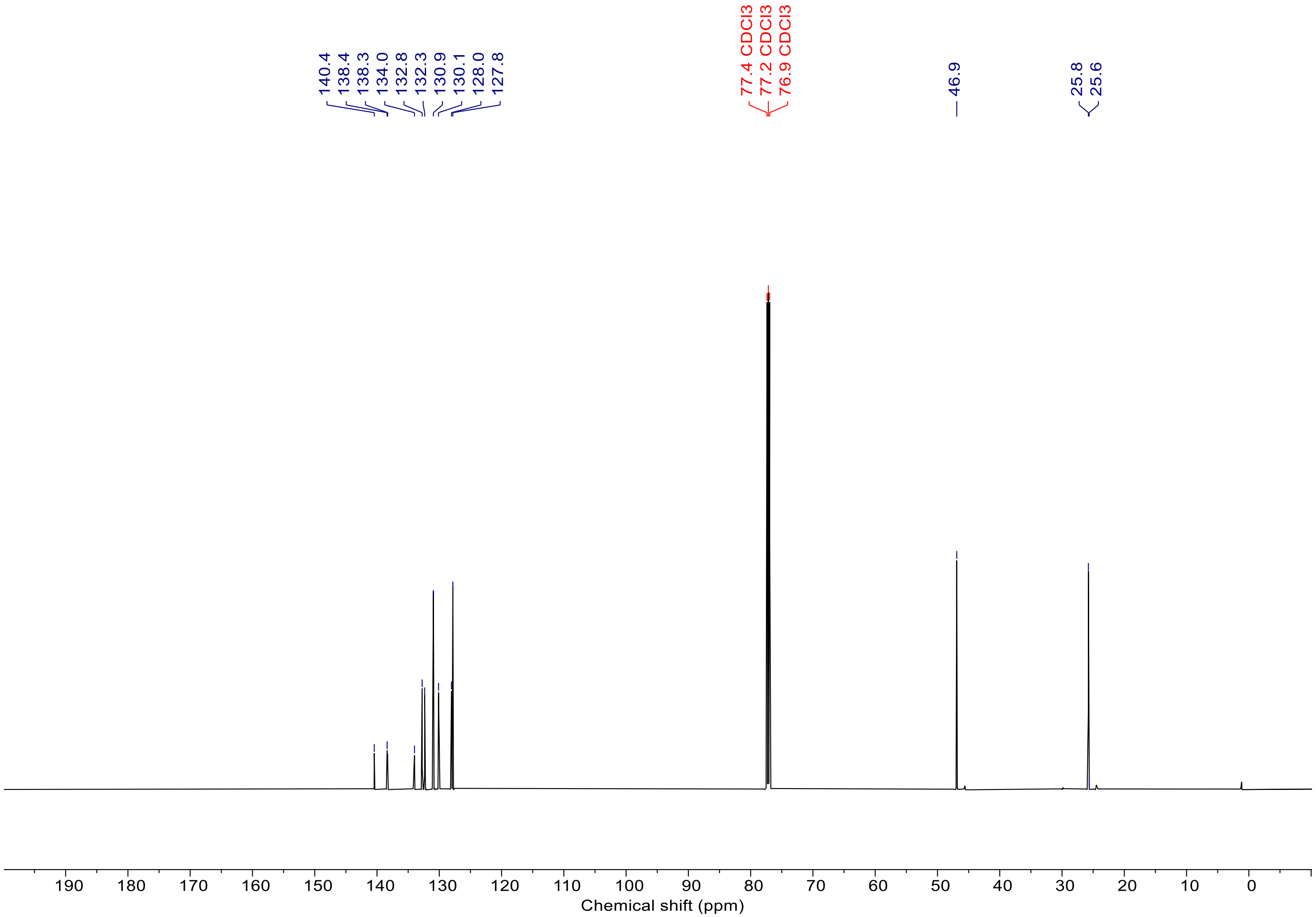

^{1}H NMR spectrum of 3-(pyrrolidin-1-ylsulfonyl)-5-(trifluoromethyl)benzoic acid (15)

DMSO-d_6, 23°C

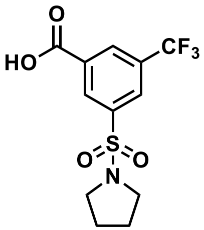

15

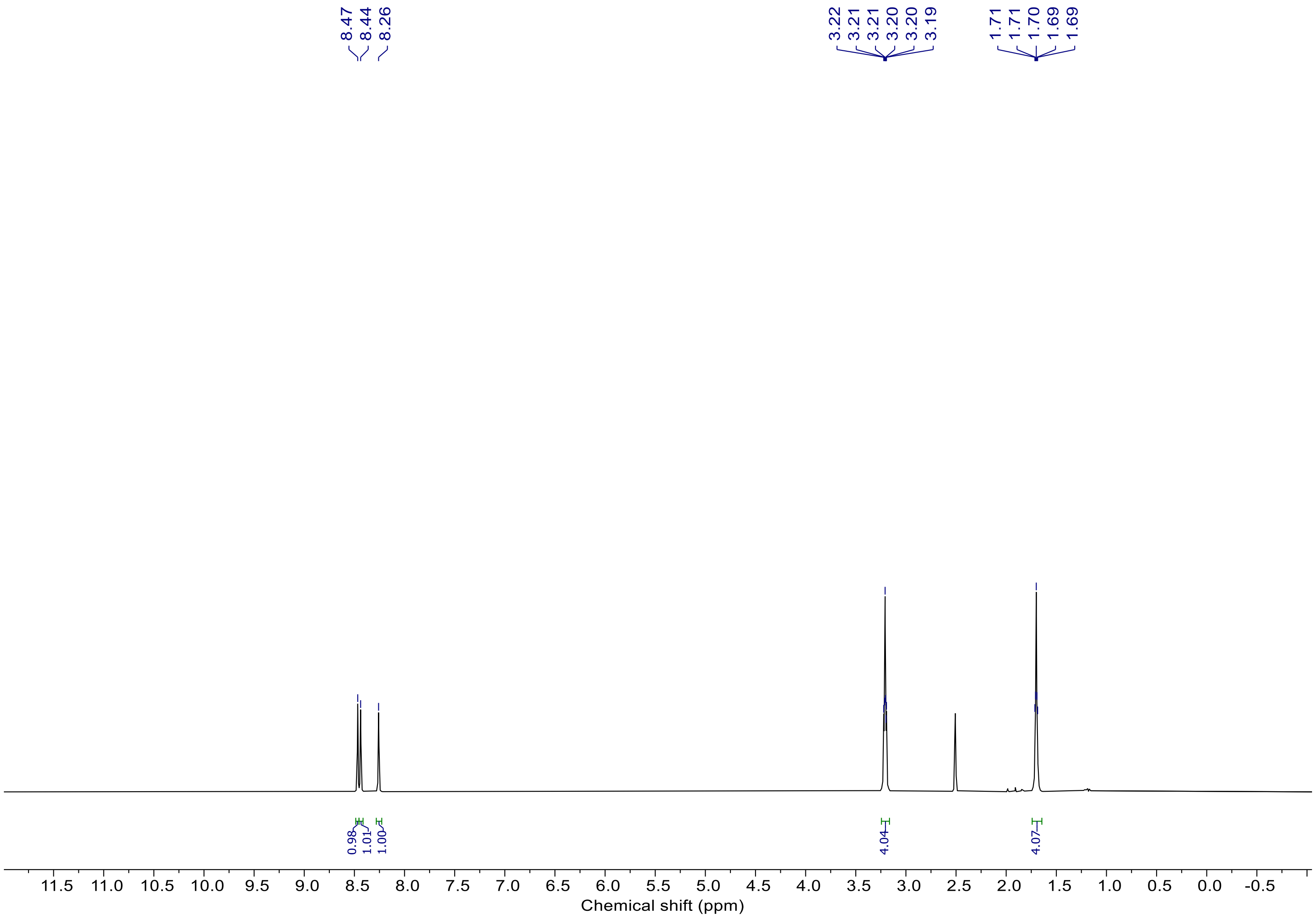

^{13}C NMR spectrum of 3-(pyrrolidin-1-ylsulfonyl)-5-(trifluoromethyl)benzoic acid (15)

DMSO-d_6, 23°C

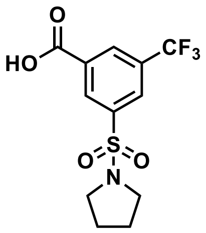

15

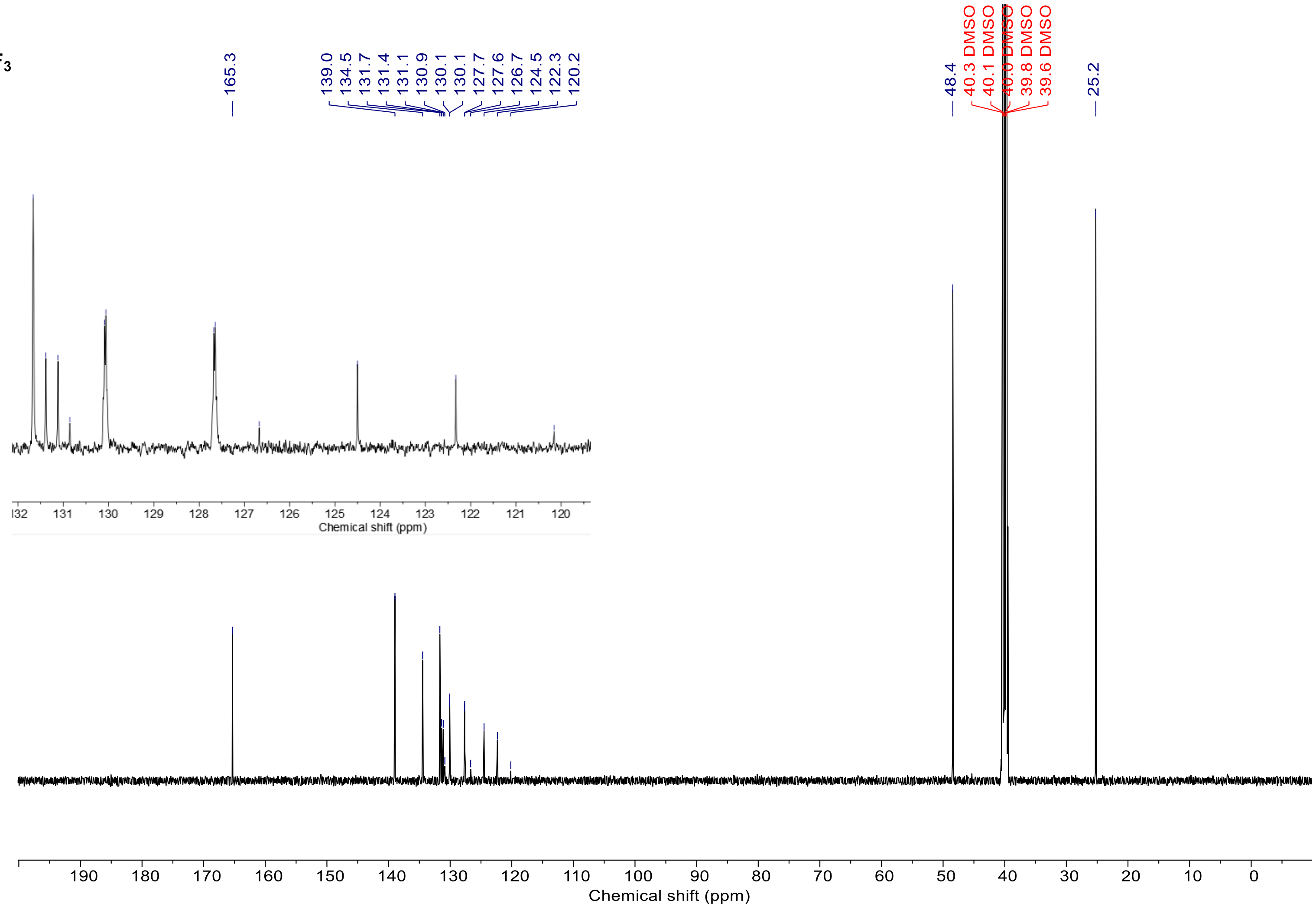

^{19}F NMR spectrum of 3-(pyrrolidin-1-ylsulfonyl)-5-(trifluoromethyl)benzoic acid (15)

DMSO-d_6, 23°C

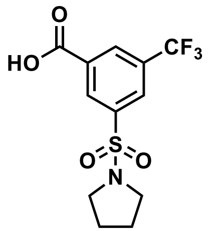

15

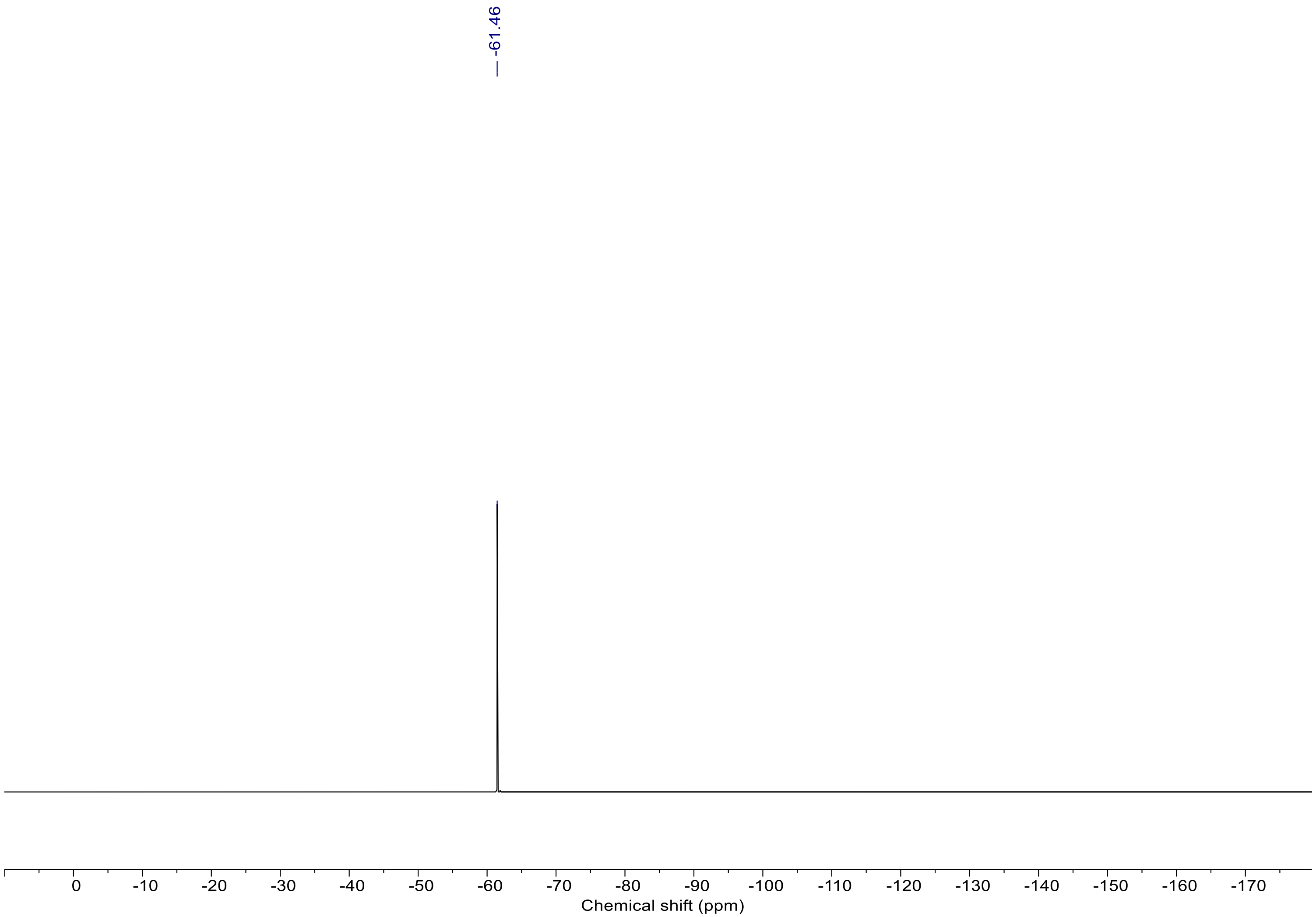

^{1}H NMR spectrum of 4-(pyrrolidin-1-ylsulfonyl)nitrobenzene (16)

$CDCl_3$, 23°C

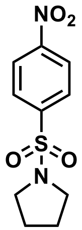

16

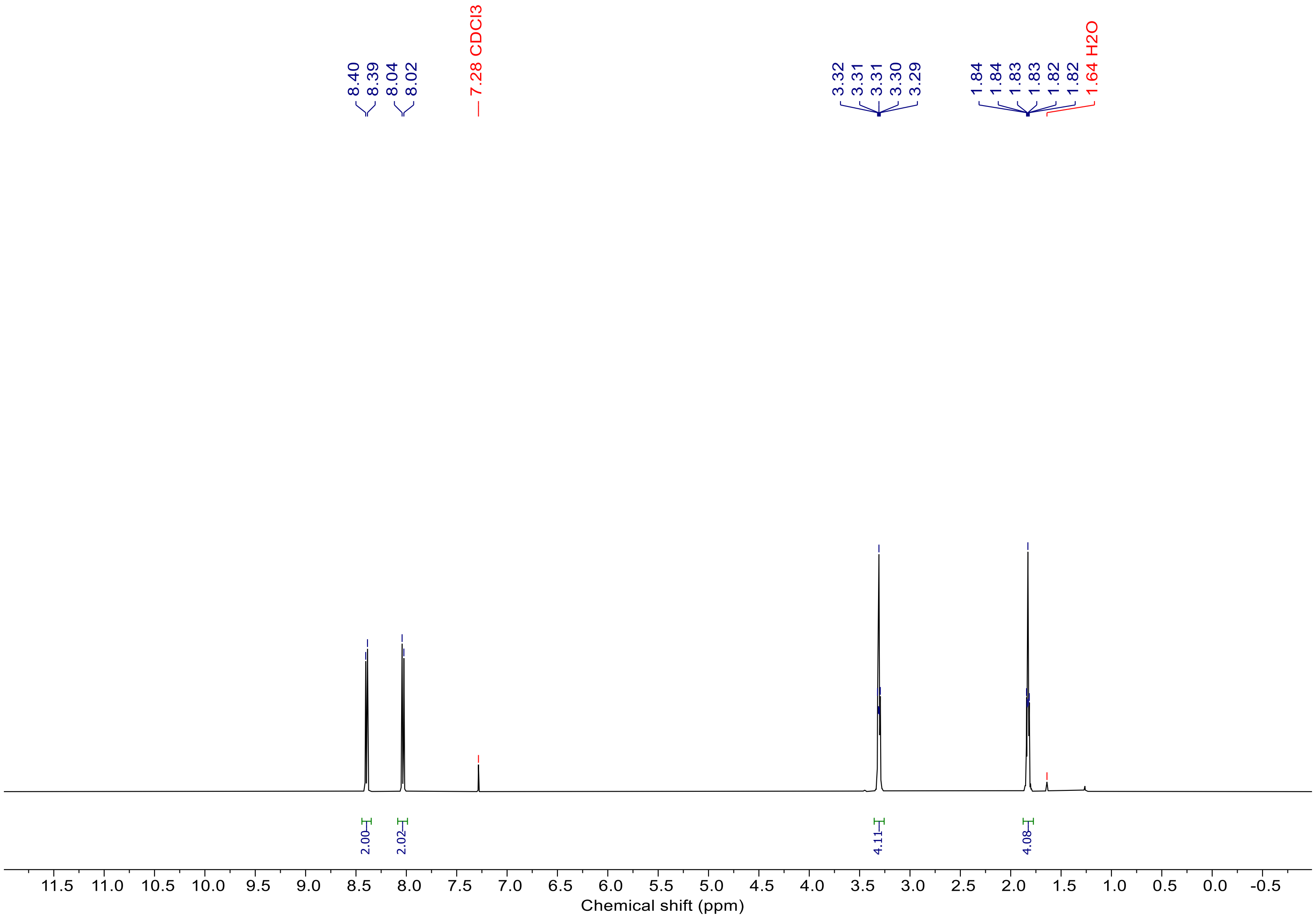

^{13}C NMR spectrum of 4-(pyrrolidin-1-ylsulfonyl)nitrobenzene (16)

$CDCl_3$, 23°C

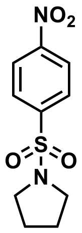

16

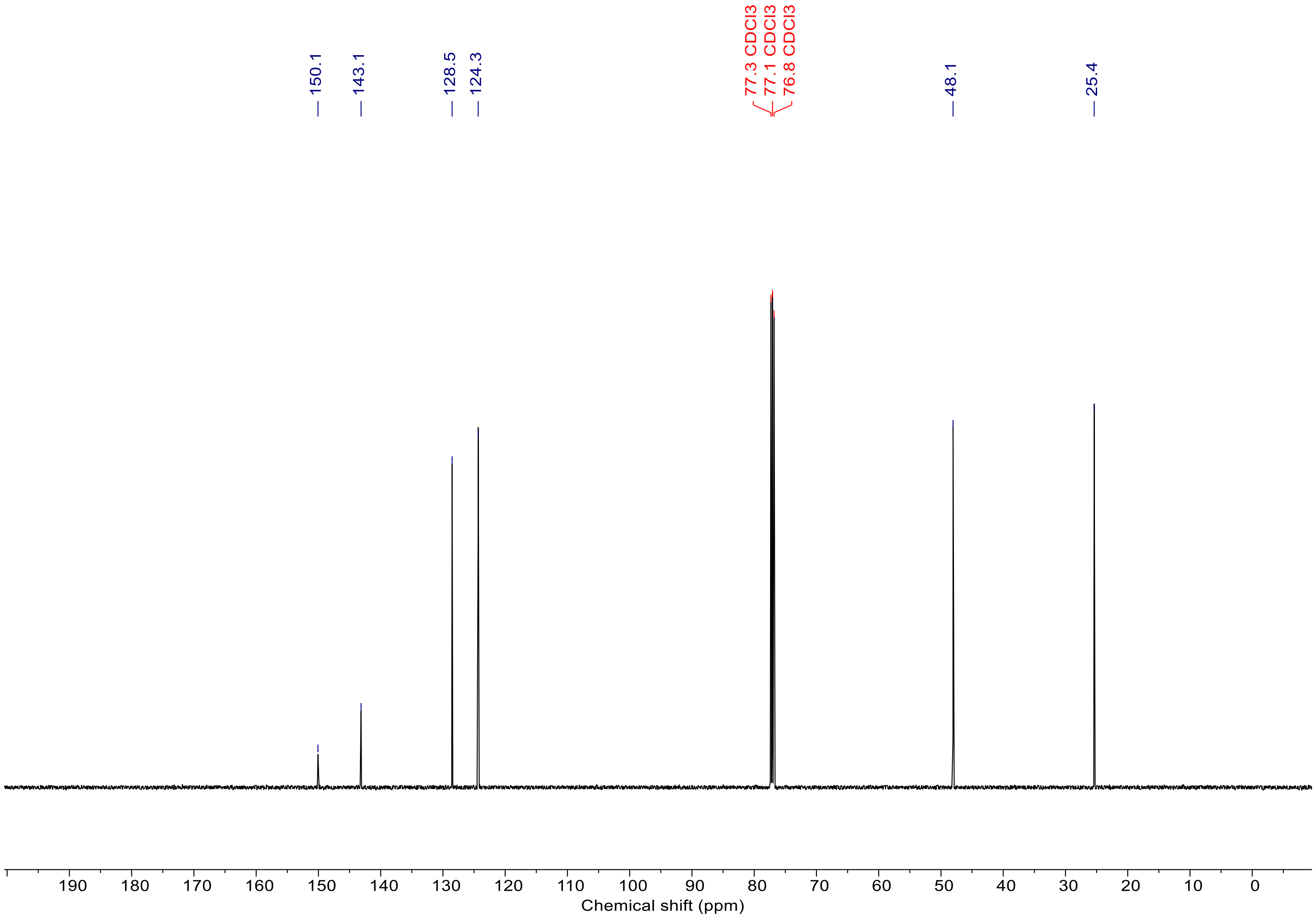

^{1}H NMR spectrum of 1-((2-chloro-4-iodophenyl)sulfonyl)pyrrolidine (17)

$CDCl_3$, 23°C

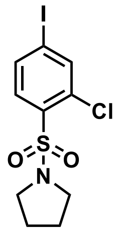

17

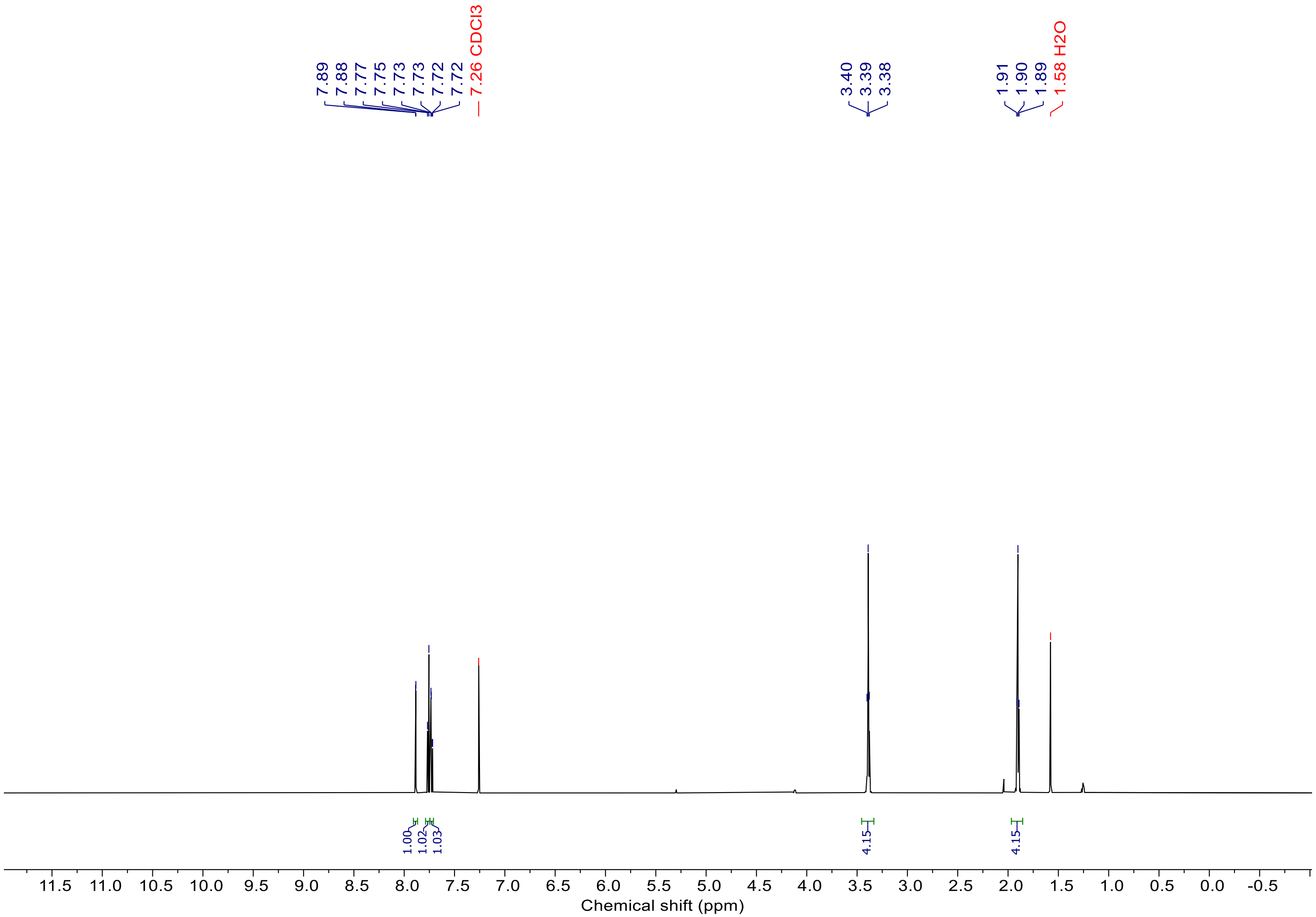

^{13}C NMR spectrum of 1-((2-chloro-4-iodophenyl)sulfonyl)pyrrolidine (17)

$CDCl_3$, 23°C

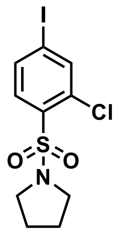

17

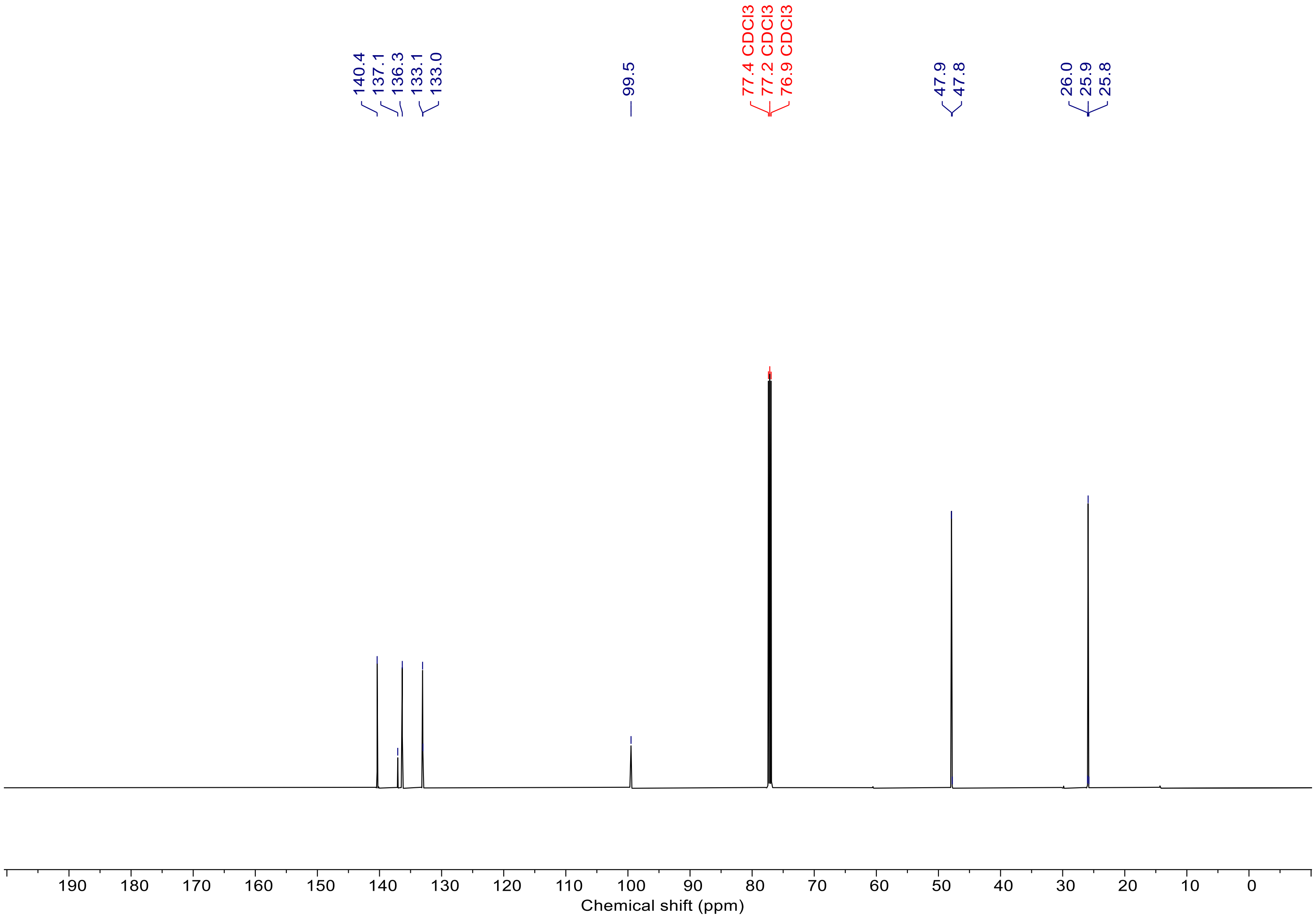

^{1}H NMR spectrum of phenyl(4-(pyrrolidin-1-ylsulfonyl)phenyl)methanone (18)

$CDCl_3$, 23°C

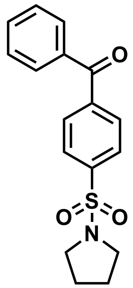

18

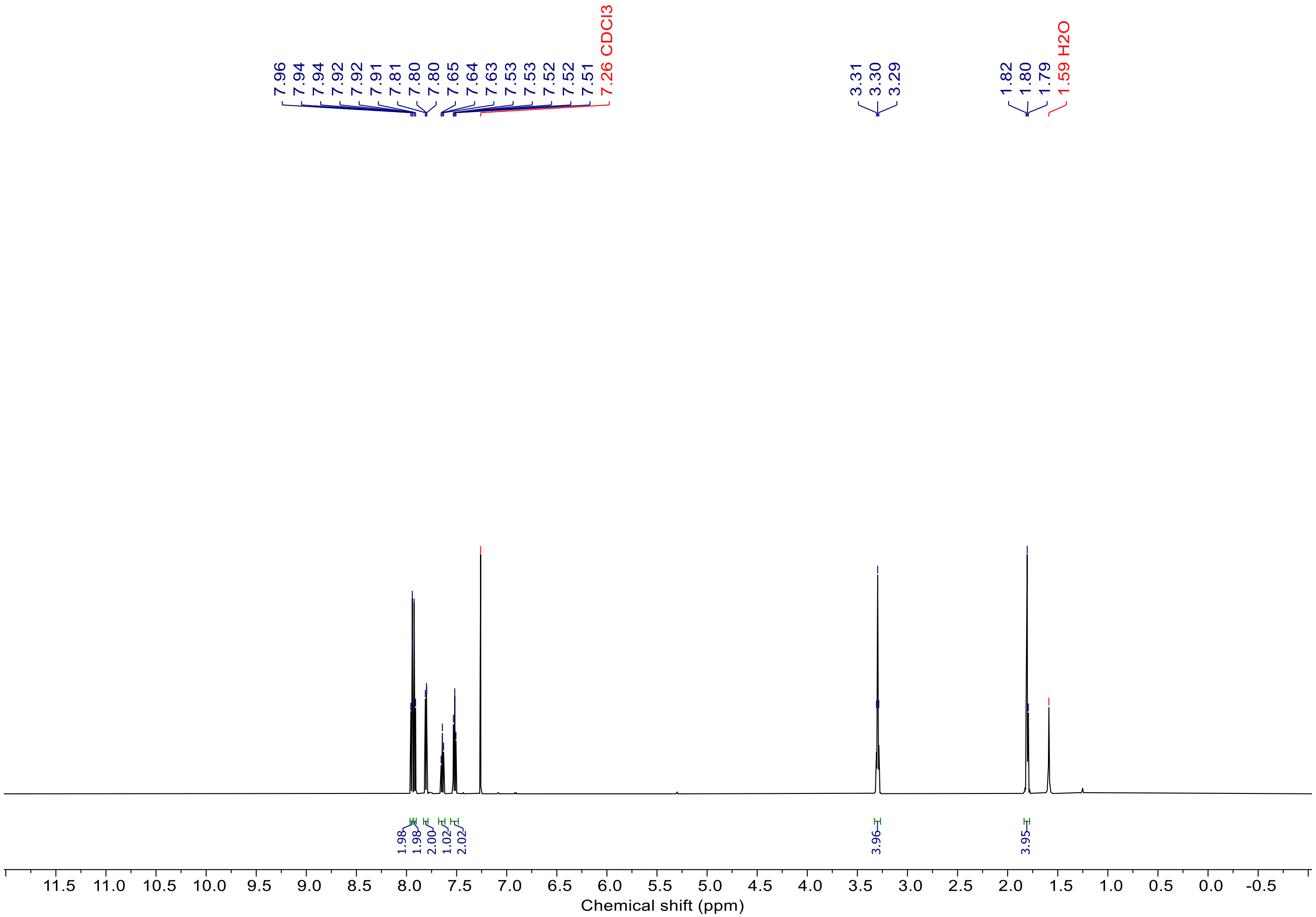

^{13}C NMR spectrum of phenyl(4-(pyrrolidin-1-ylsulfonyl)phenyl)methanone (18)

$CDCl_3$, 23°C

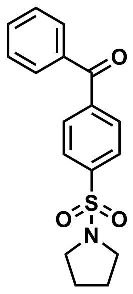

18

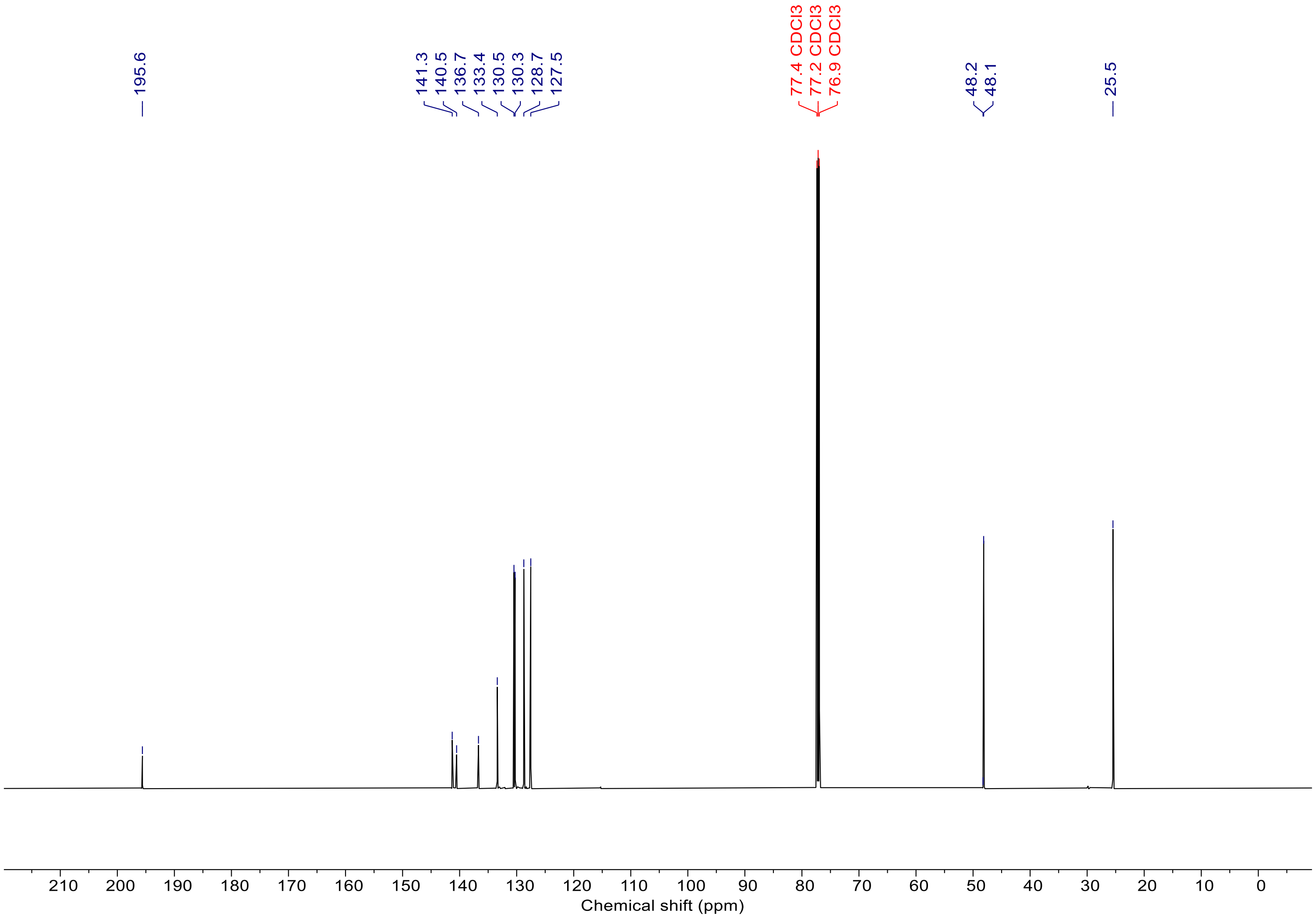

^{1}H NMR spectrum of 1-((6-bromonaphthalen-2-yl)sulfonyl)pyrrolidine (19)

$CDCl_3$, 23°C

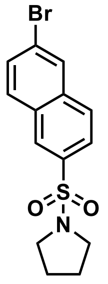

19

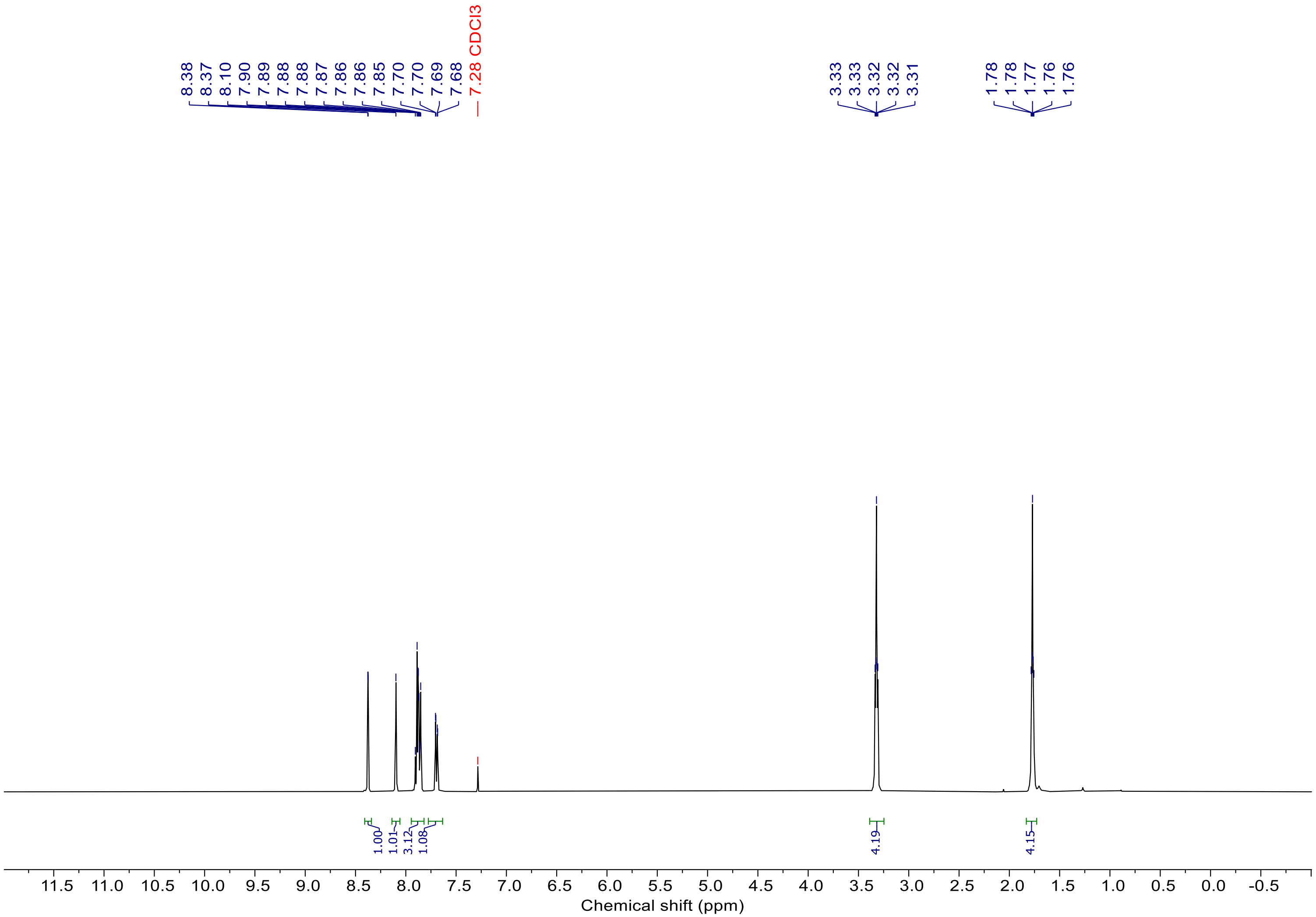

^{13}C NMR spectrum of 1-((6-bromonaphthalen-2-yl)sulfonyl)pyrrolidine (19)

$CDCl_3$, 23°C

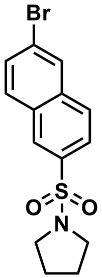

19

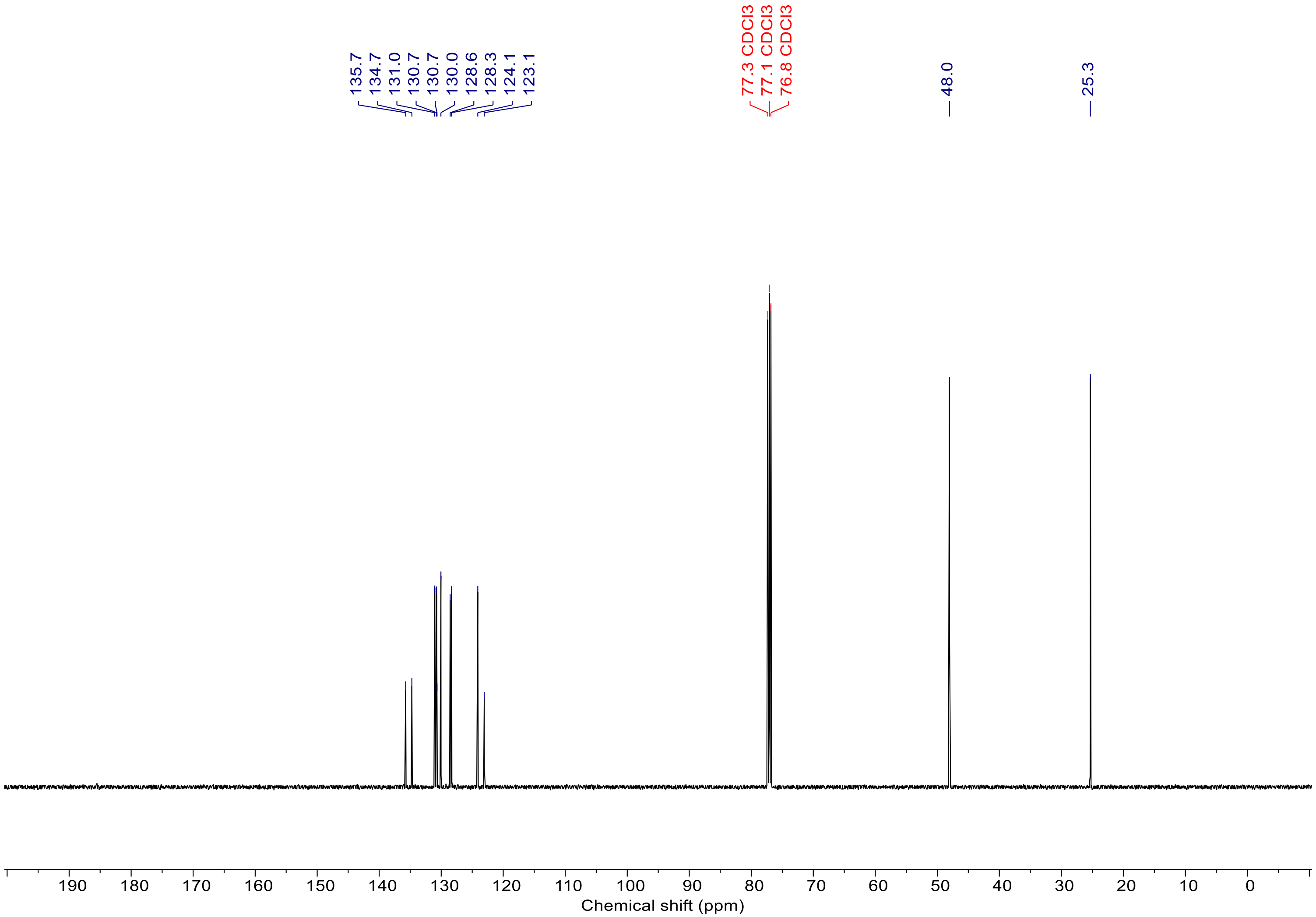

^{1}H NMR spectrum of 2-chloro-4-(pyrrolidin-1-ylsulfonyl)benzonitrile (20)

$CDCl_3$, 23°C

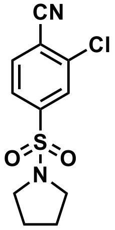

20

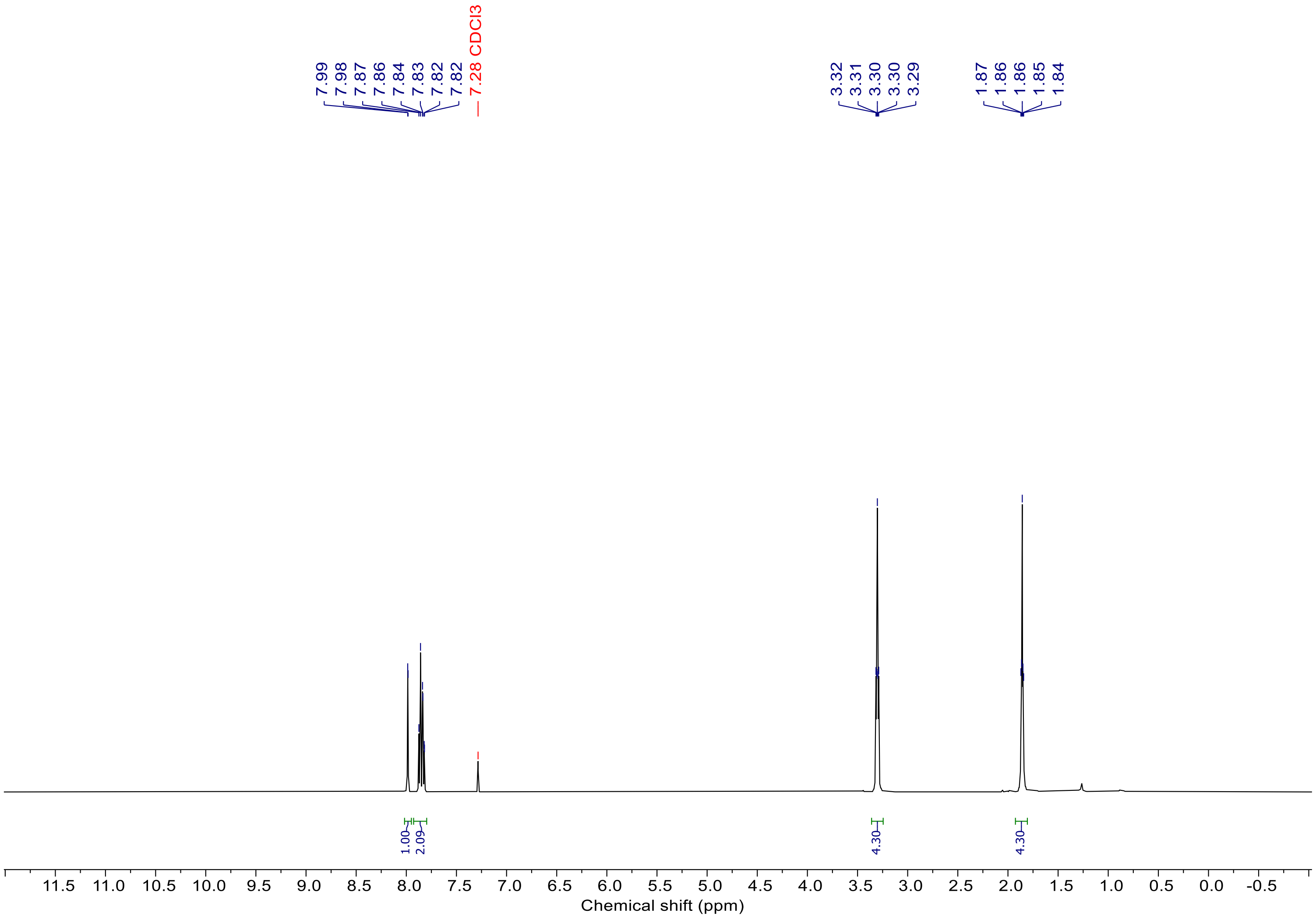

^{13}C NMR spectrum of 2-chloro-4-(pyrrolidin-1-ylsulfonyl)benzonitrile (20)

$CDCl_3$, 23°C

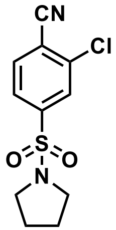

20

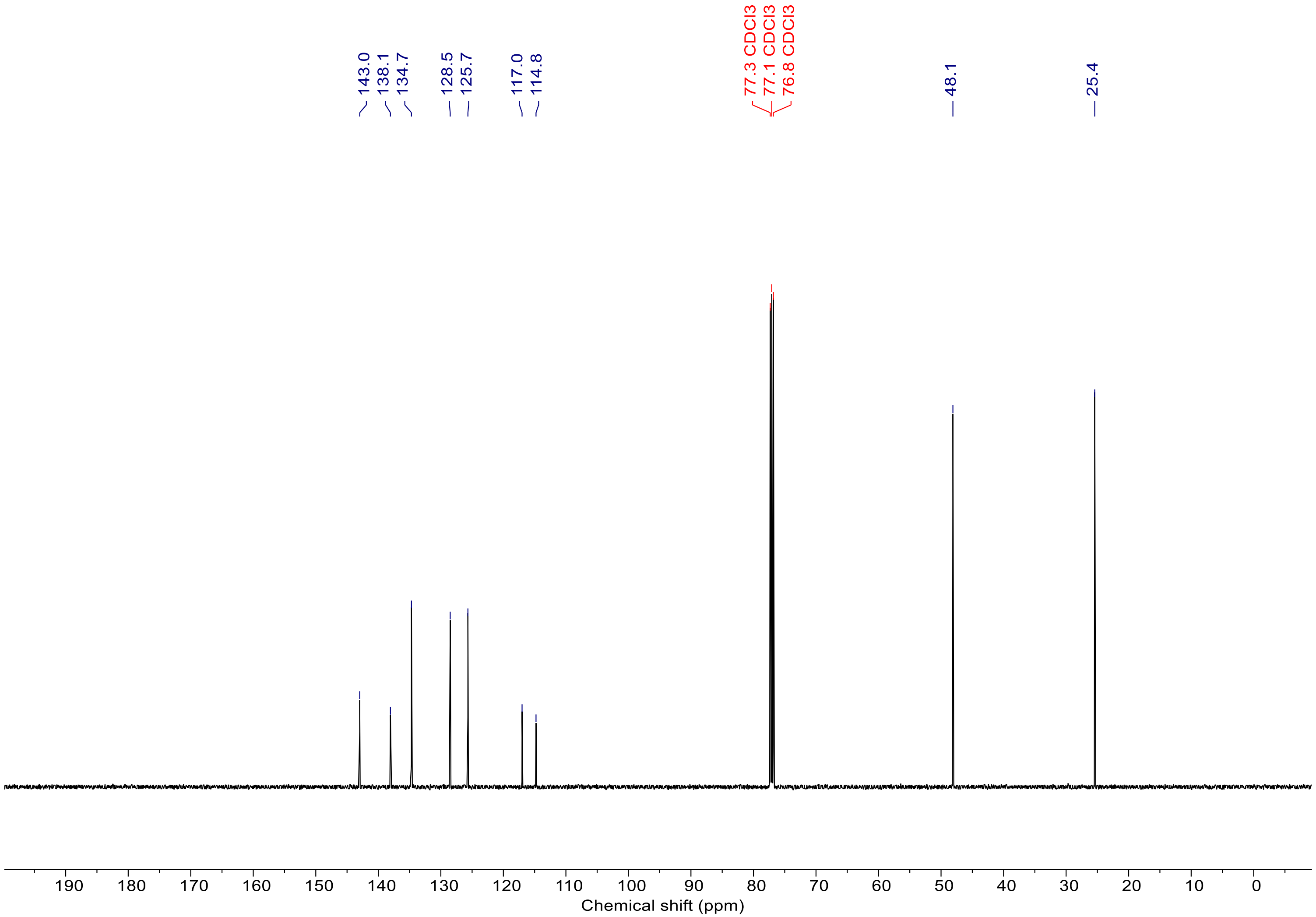

^{1}H NMR spectrum of 4-(pyrrolidin-1-ylsulfonyl)trifluoromethoxybenzene (21)

$CDCl_3$, 23°C

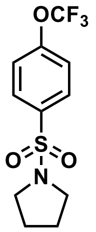

21

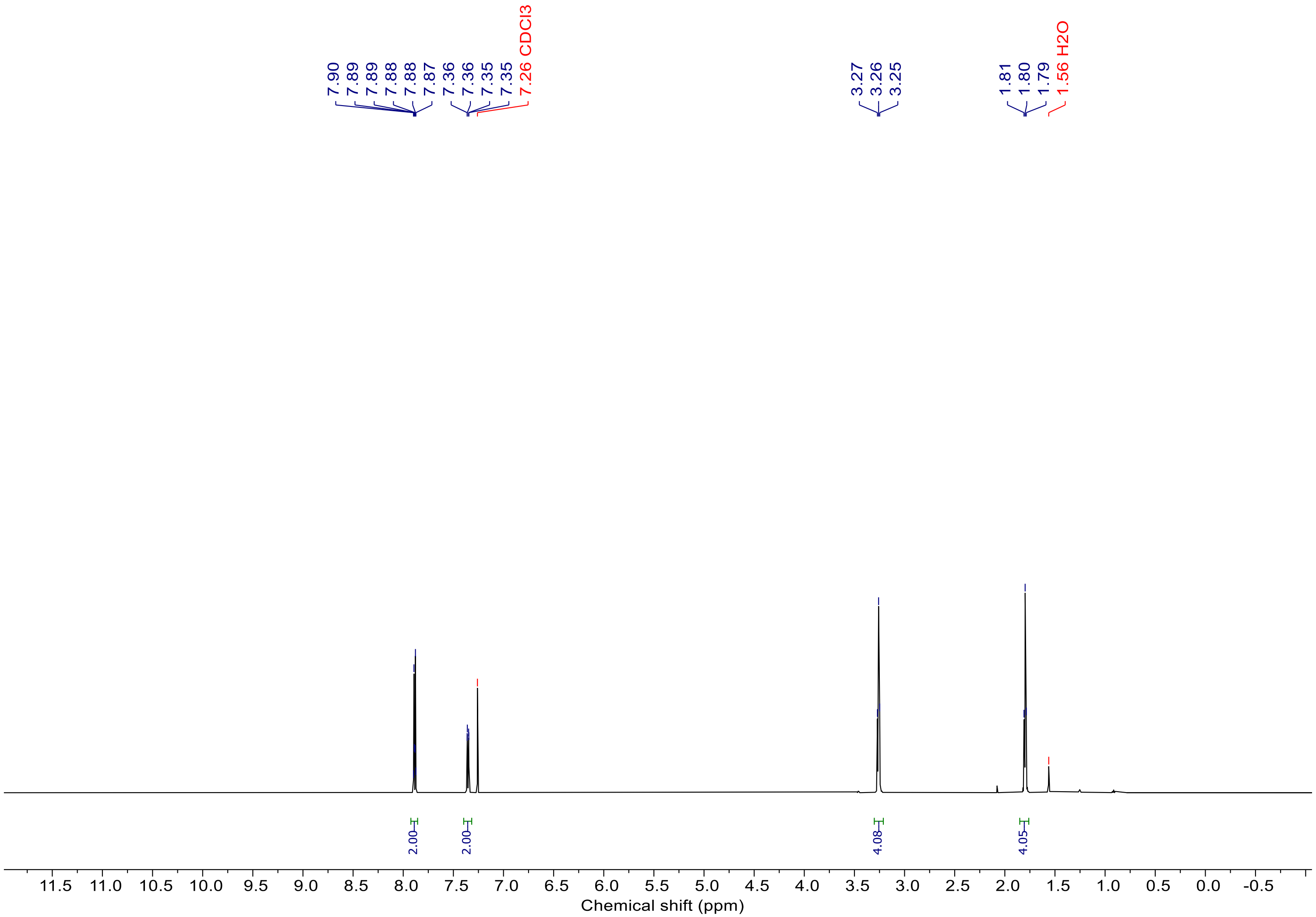

^{13}C NMR spectrum of 4-(pyrrolidin-1-ylsulfonyl)trifluoromethoxybenzene (21)

$CDCl_3$, 23°C

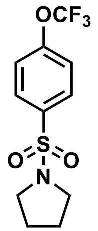

21

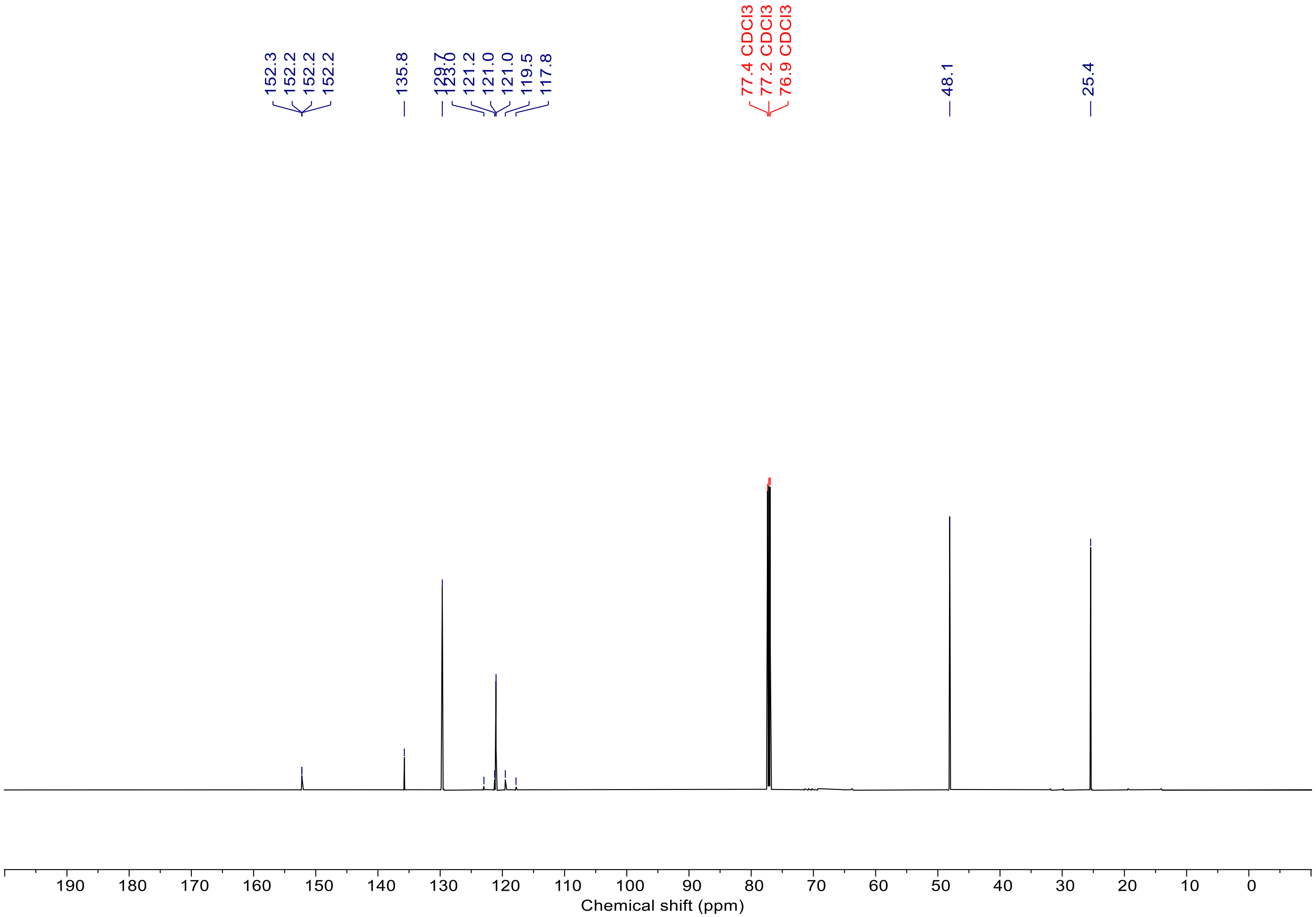

^{19}F NMR spectrum of 4-(pyrrolidin-1-ylsulfonyl)trifluoromethoxybenzene (21)

CDCl$_3$, 23°C

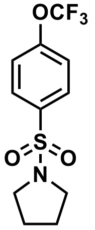

21

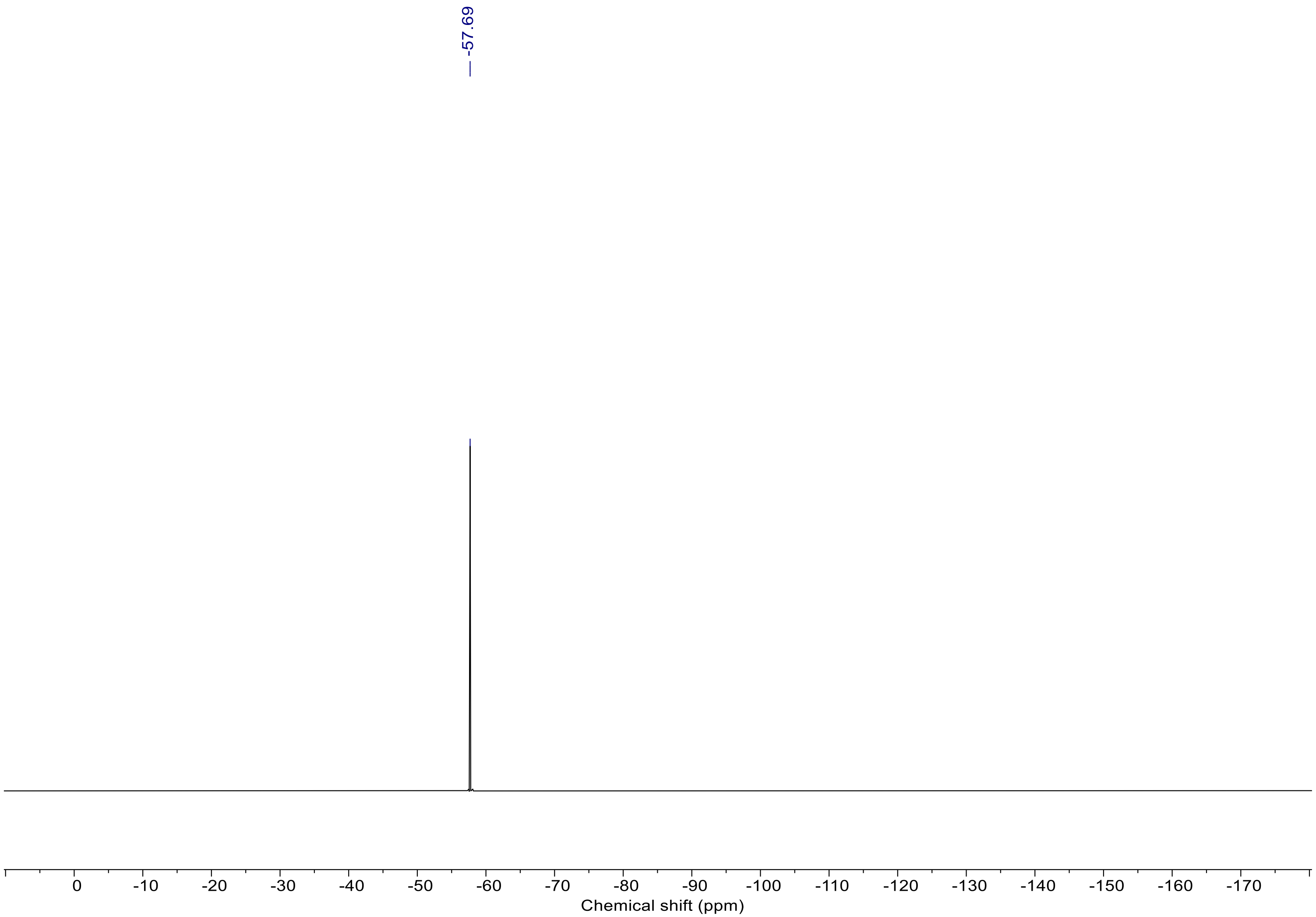

^{1}H NMR spectrum of 1-((2-((trifluoromethyl)thio)phenyl)sulfonyl)pyrrolidine (22)

$CDCl_3$, 23°C

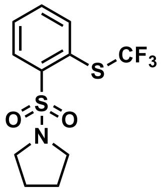

22

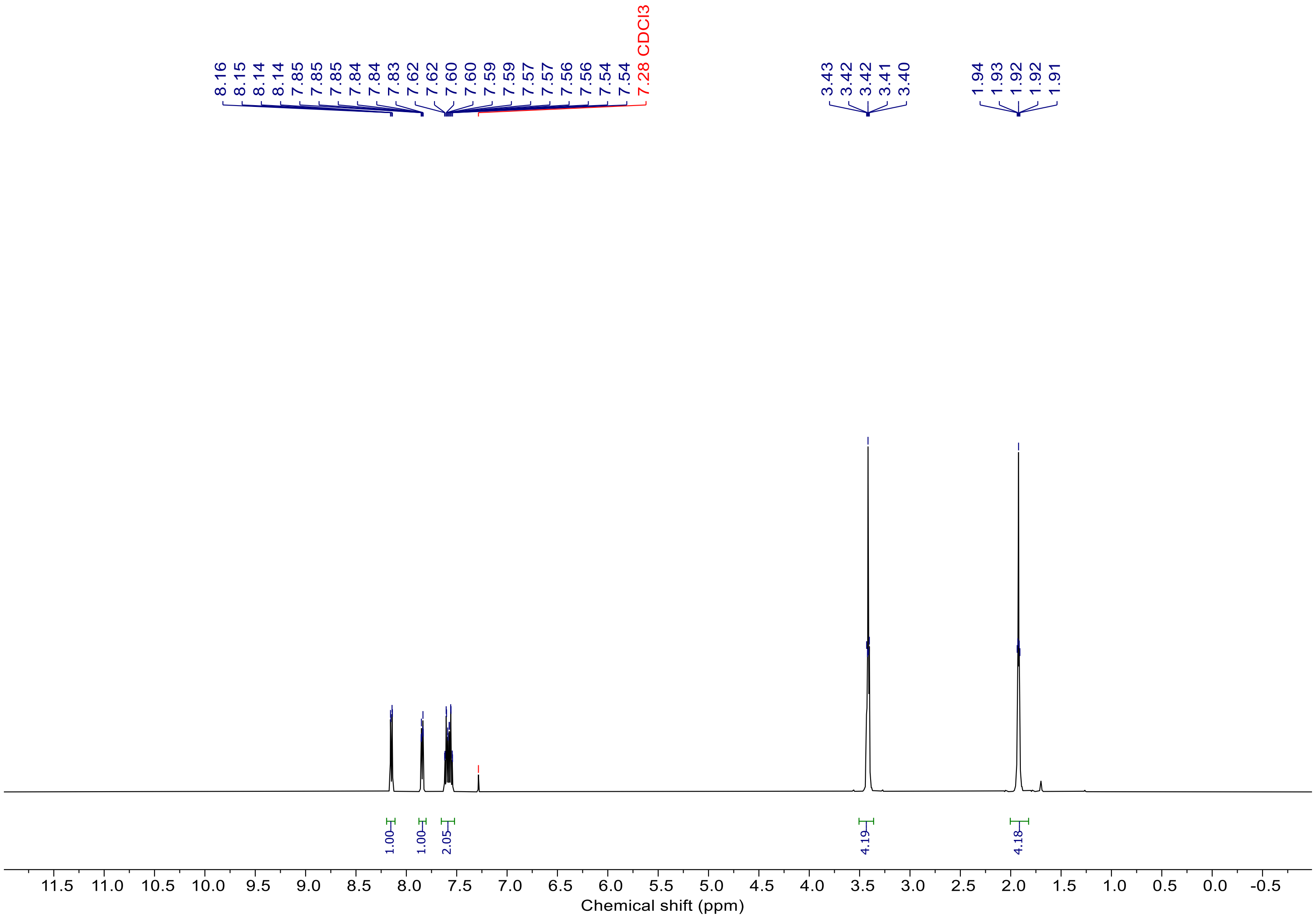

^{13}C NMR spectrum of 1-((2-((trifluoromethyl)thio)phenyl)sulfonyl)pyrrolidine (22)

$CDCl_3$, 23°C

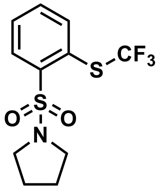

22

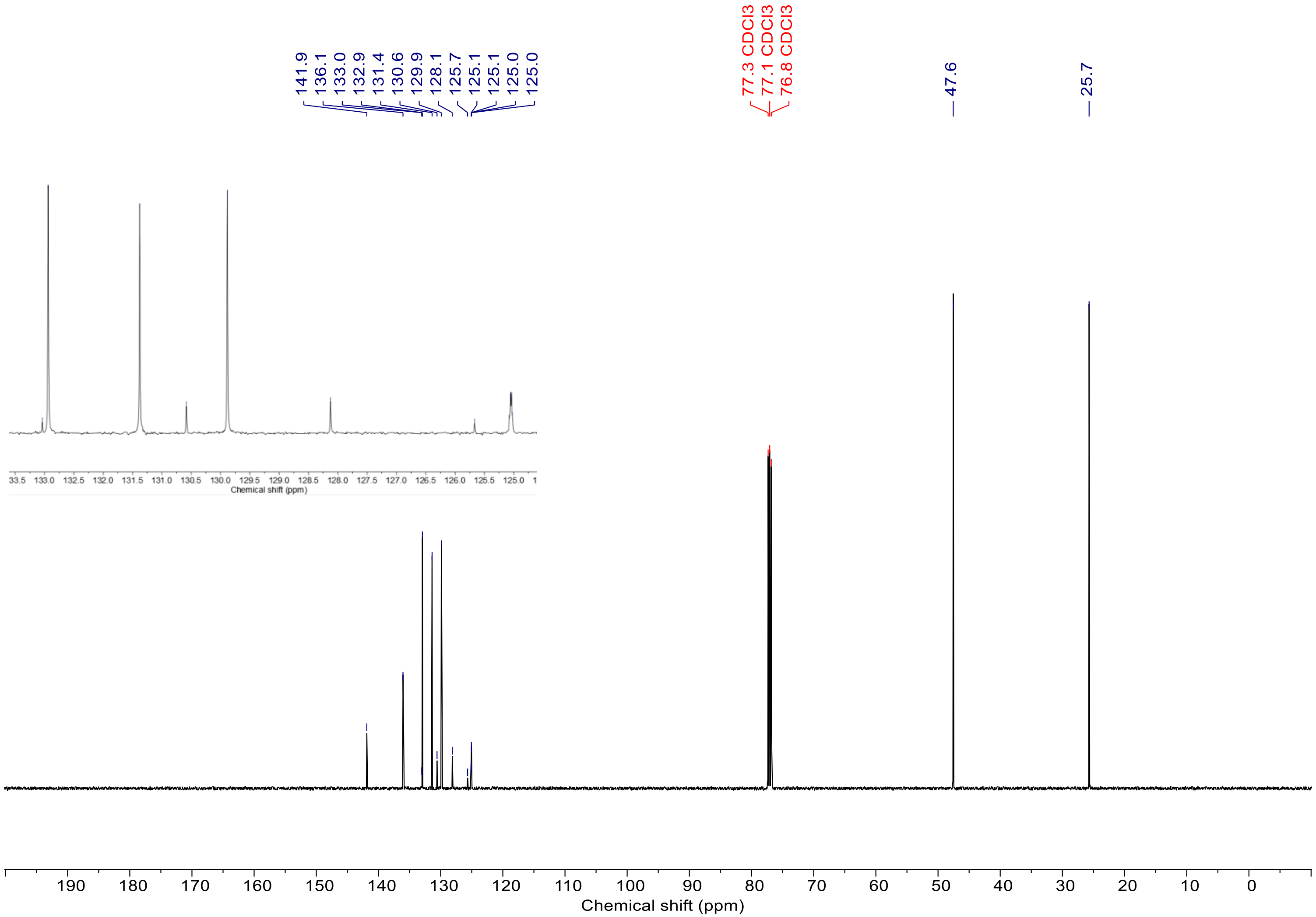

^{19}F NMR spectrum of 1-((2-((trifluoromethyl)thio)phenyl)sulfonyl)pyrrolidine (22)

$CDCl_3$, 23°C

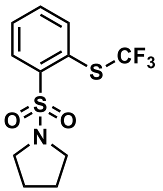

22

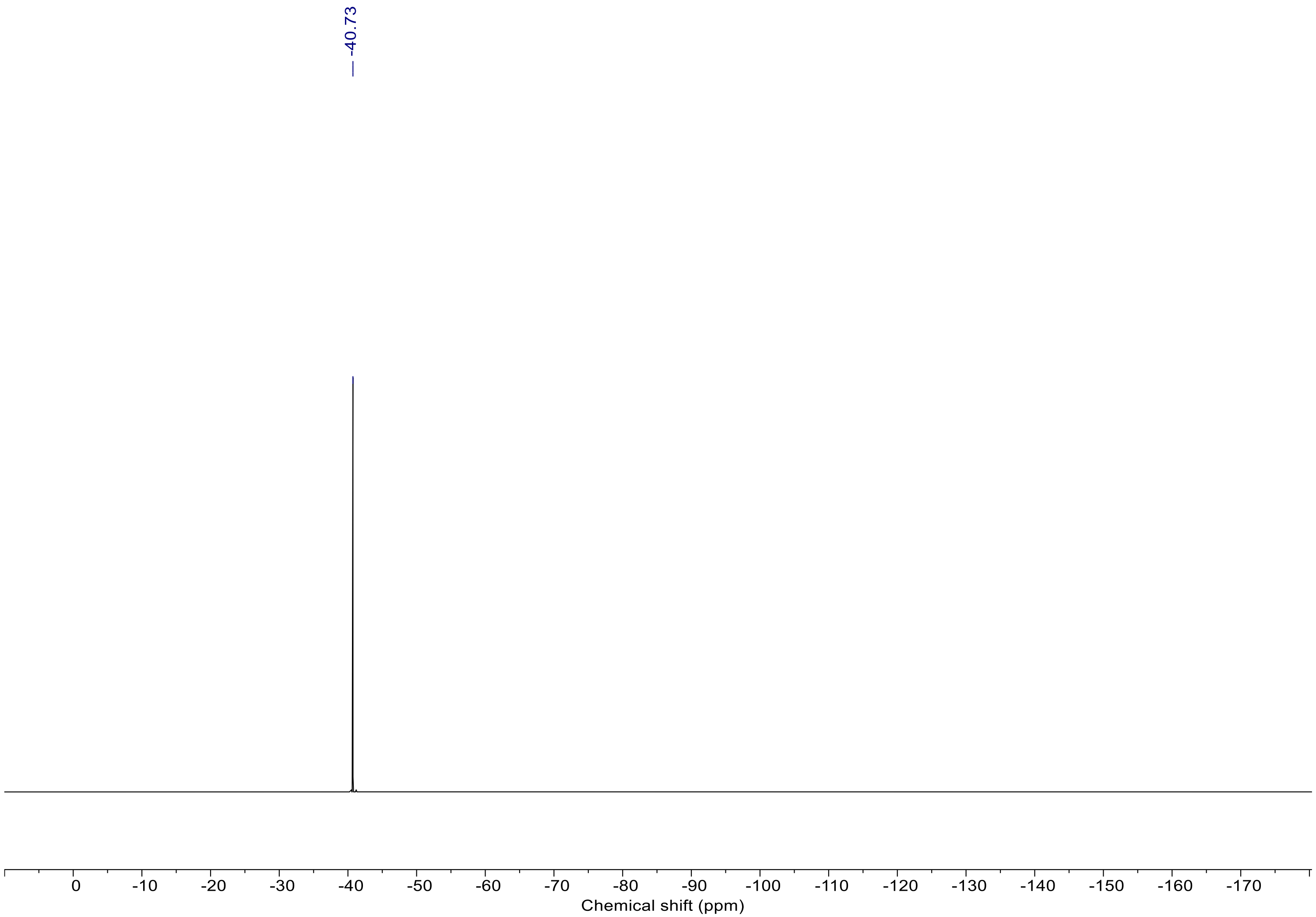

^{1}H NMR spectrum of 3-bromo-4-(pyrrolidin-1-ylsulfonyl)benzonitrile (23)

$CDCl_3$, 23°C

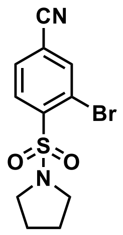

23

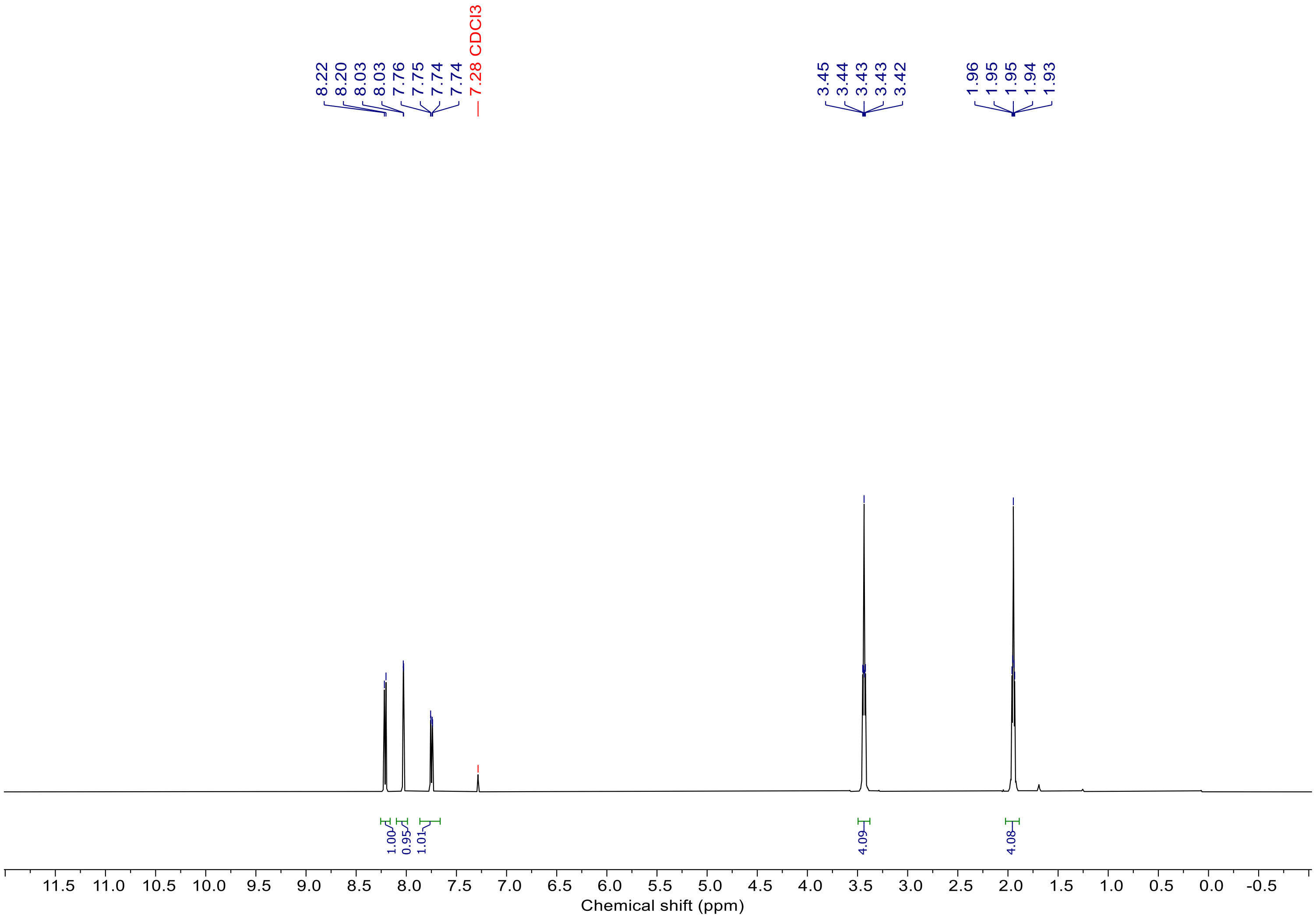

^{13}C NMR spectrum of 3-bromo-4-(pyrrolidin-1-ylsulfonyl)benzonitrile (23)

$CDCl_3$, 23°C

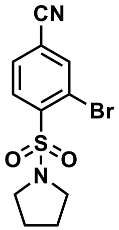

23

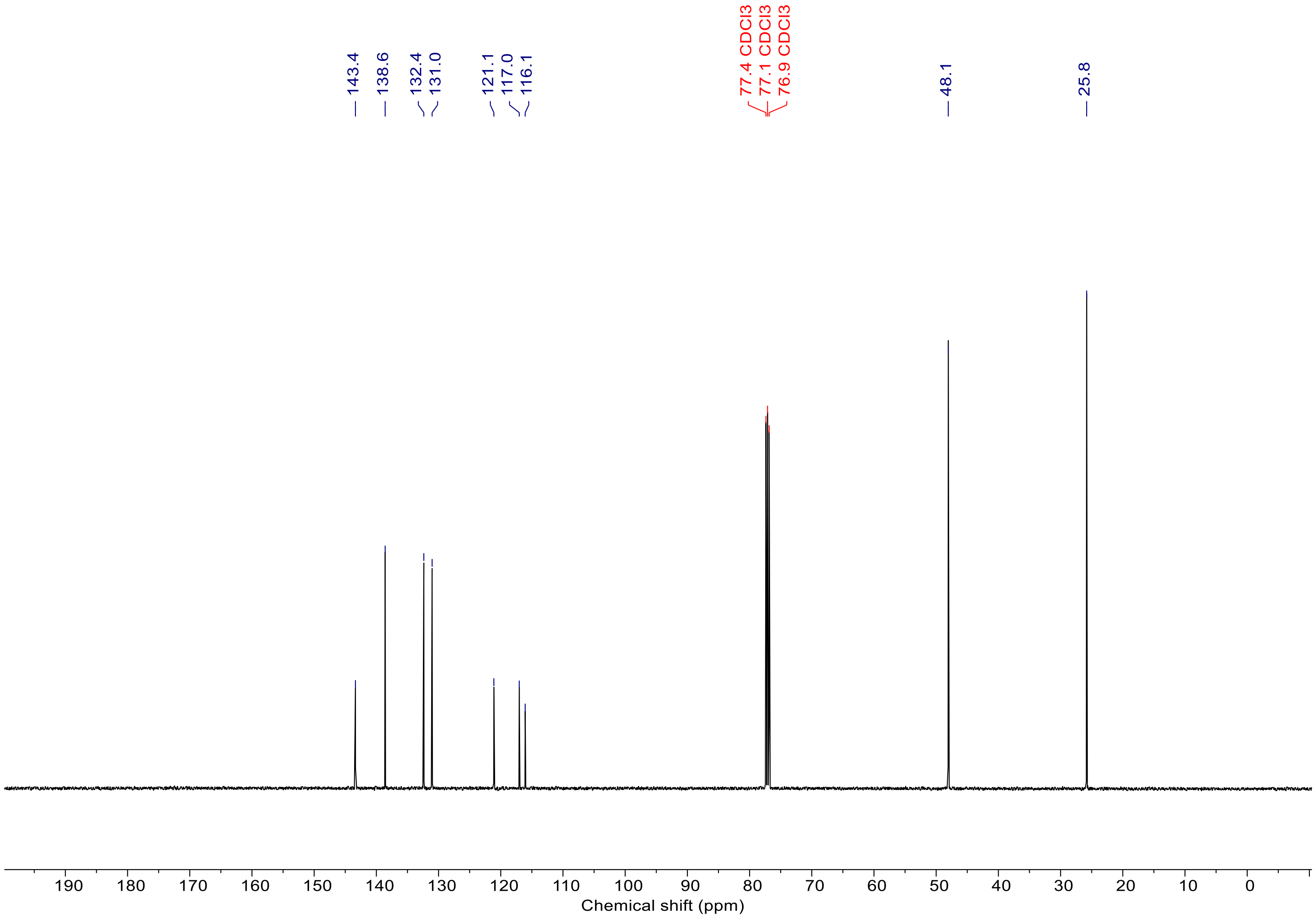

^{1}H NMR spectrum of 1-((2-methyl-3-nitrophenyl)sulfonyl)pyrrolidine (24)

$CDCl_3$, 23°C

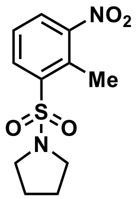

24

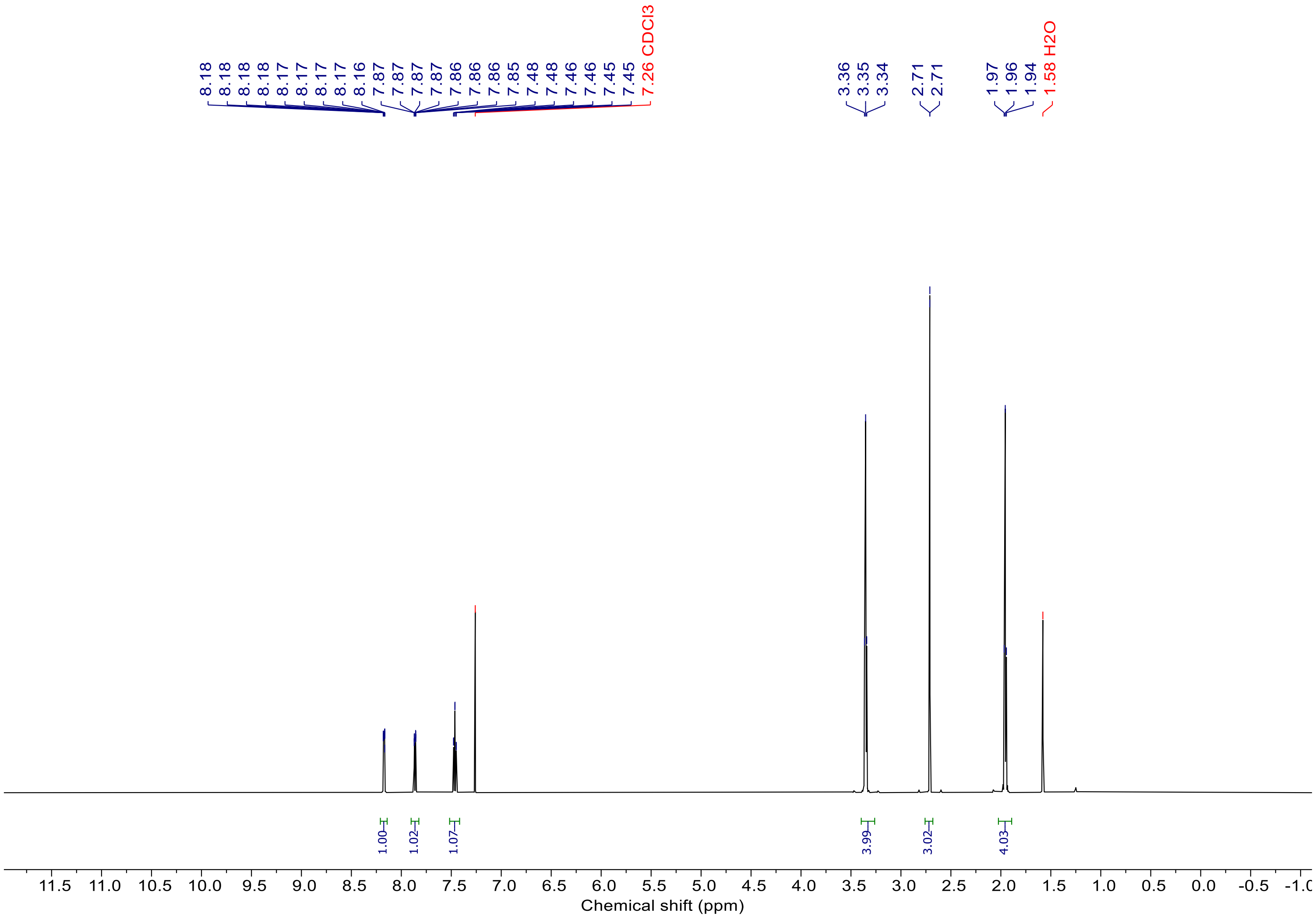

^{13}C NMR spectrum of 1-((2-methyl-3-nitrophenyl)sulfonyl)pyrrolidine (24)

$CDCl_3$, 23°C

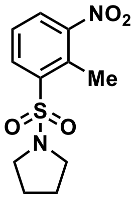

24

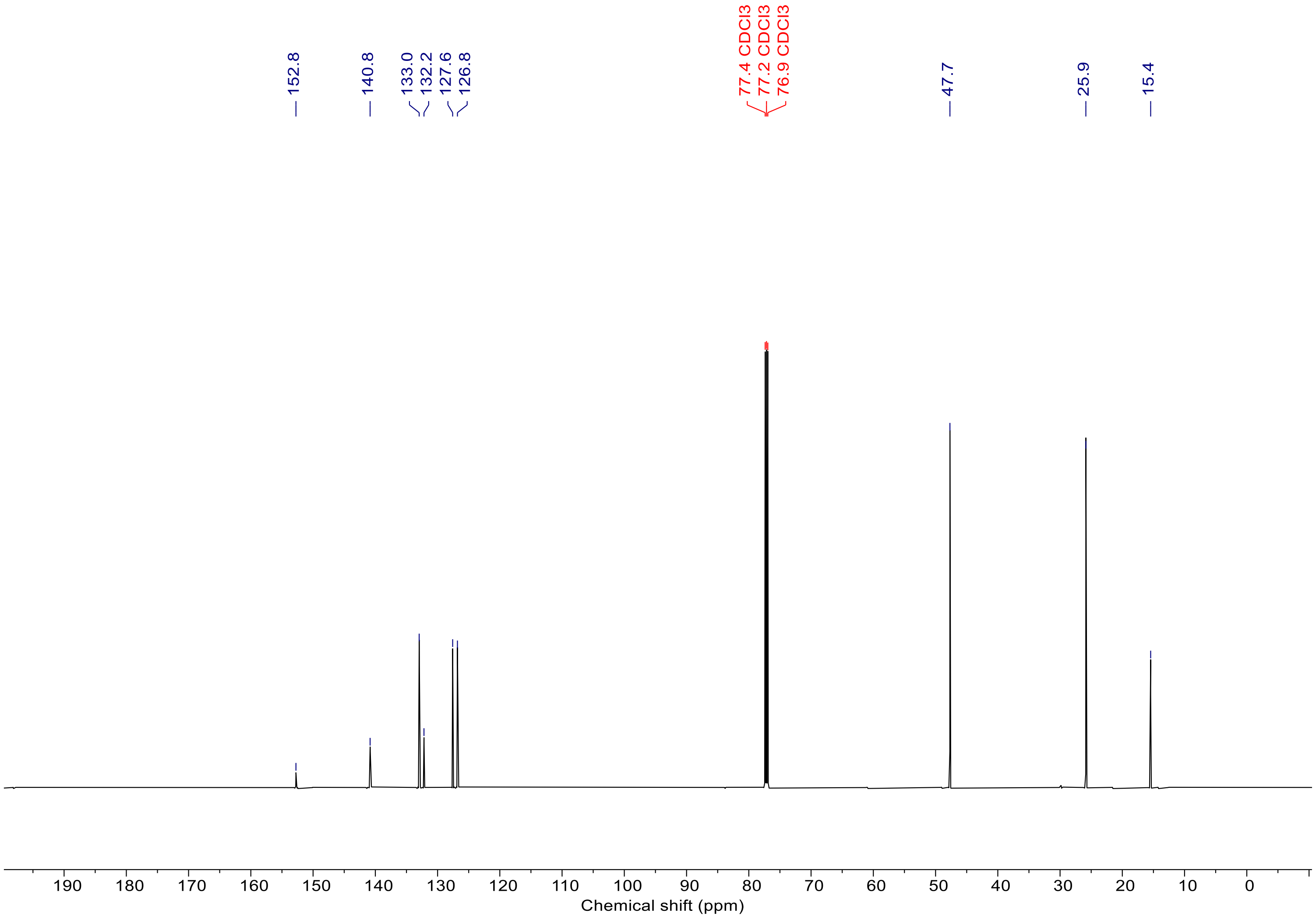

^{1}H NMR spectrum of 4-(pyrrolidin-1-ylsulfonyl)benzenesulfonamide (25)

DMSO-d_6, 23°C

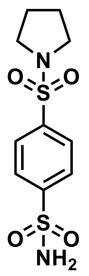

25

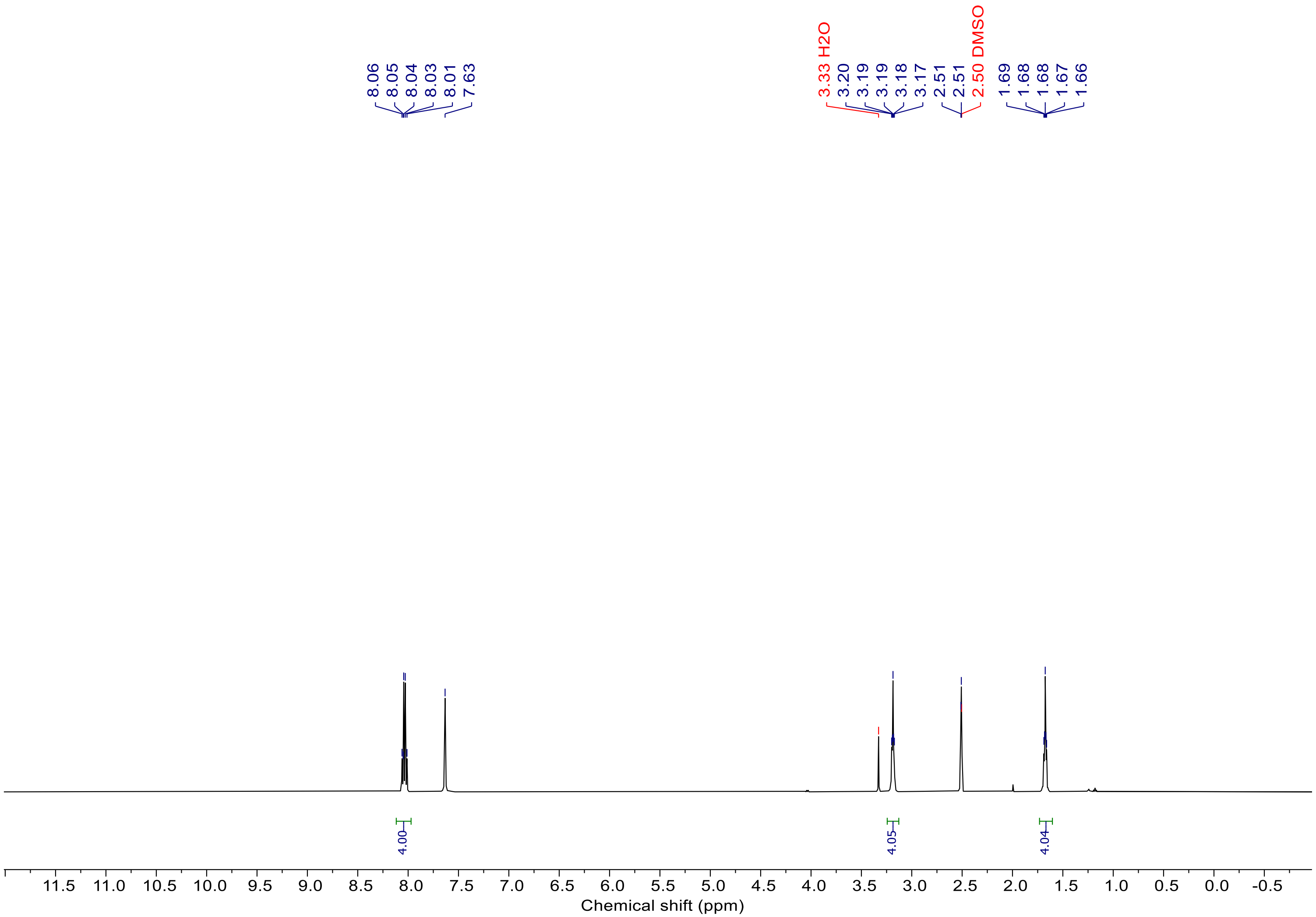

^{13}C NMR spectrum of 4-(pyrrolidin-1-ylsulfonyl)benzenesulfonamide (25)

DMSO-d_6, 23°C

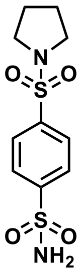

25

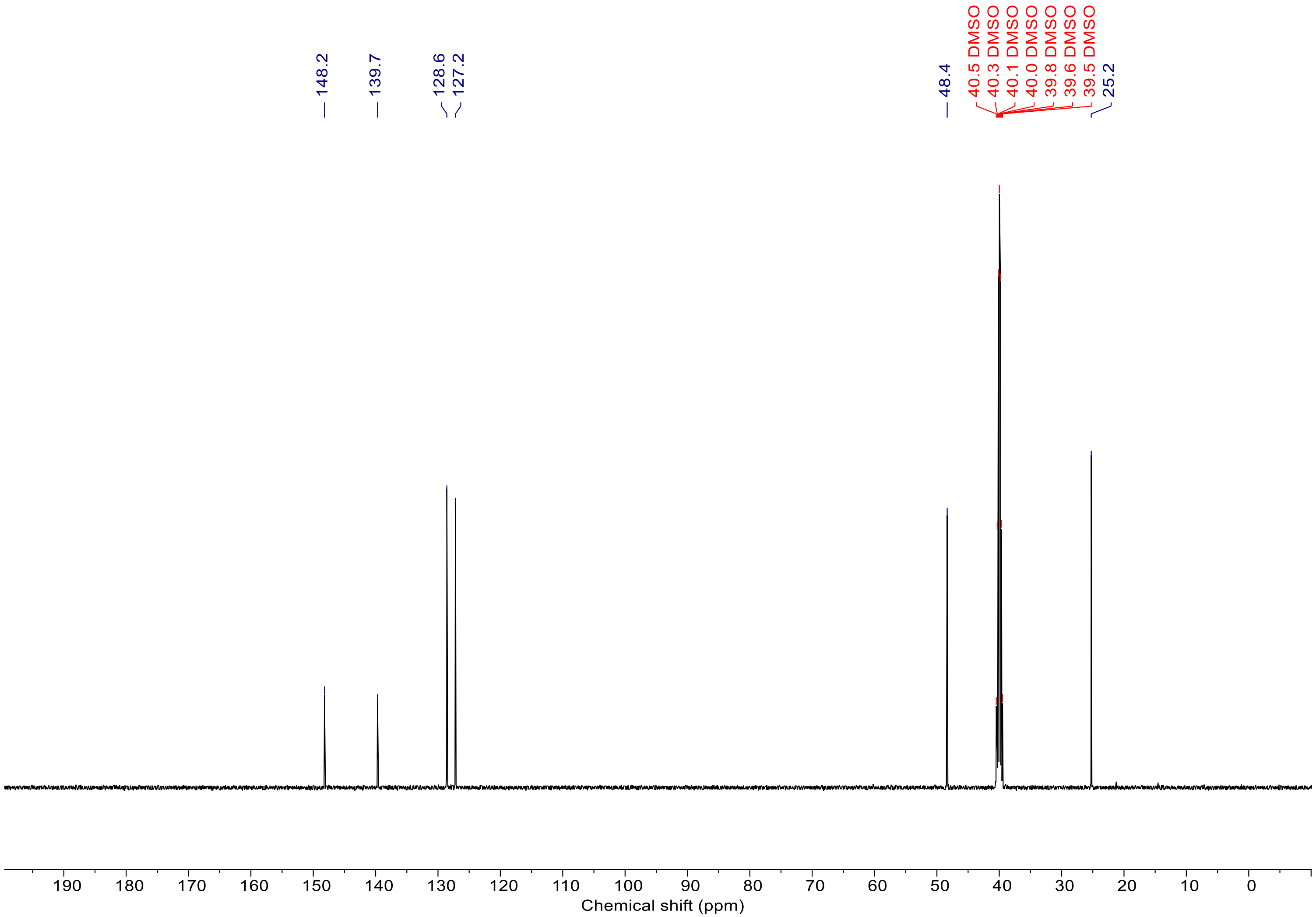

^{1}H NMR spectrum of 1-((3,5-dibromo-4-methylphenyl)sulfonyl)pyrrolidine (26)

$CDCl_3$, 23°C

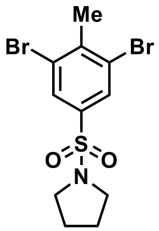

26

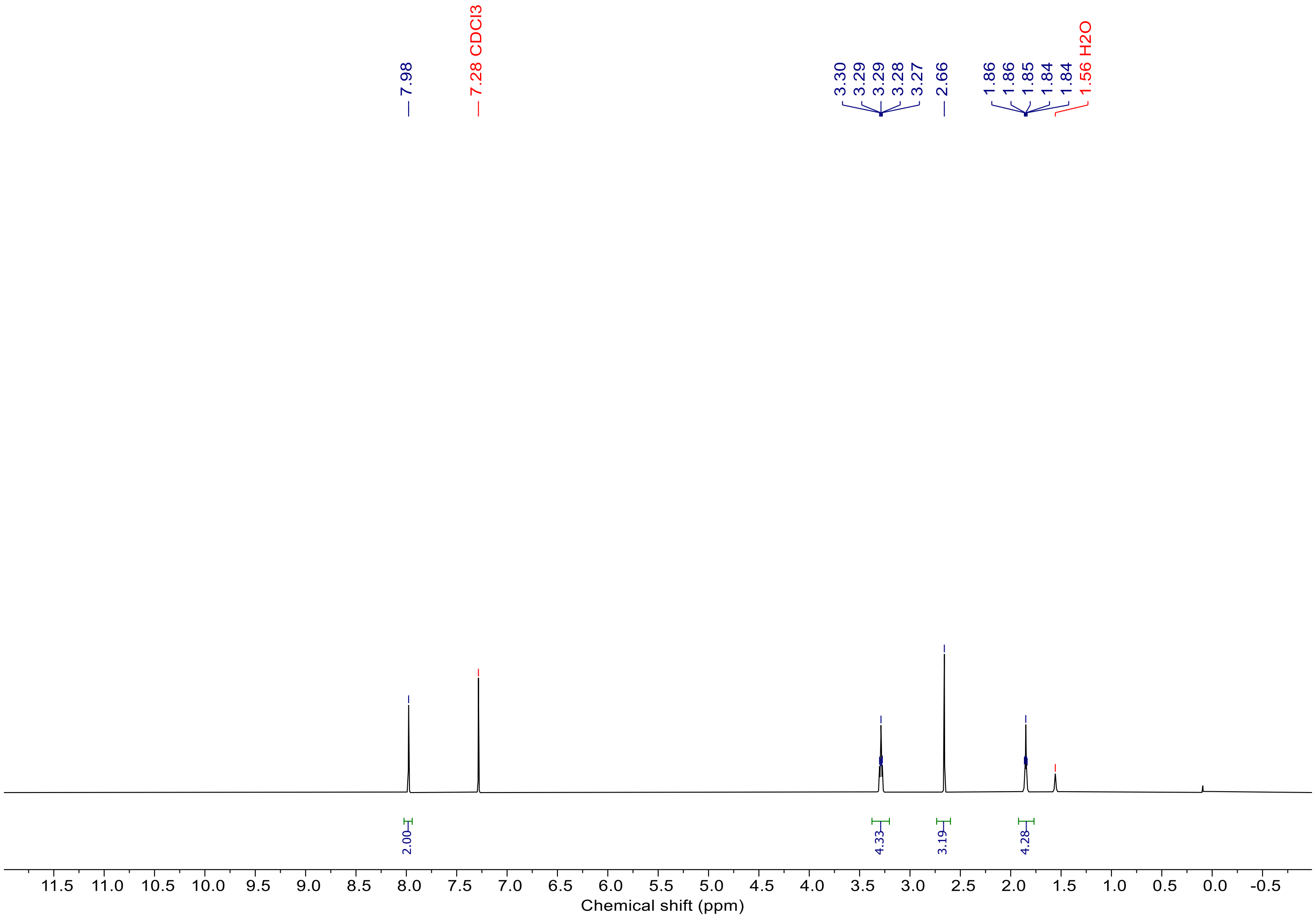

^{13}C NMR spectrum of 1-((3,5-dibromo-4-methylphenyl)sulfonyl)pyrrolidine (26)

$CDCl_3$, 23°C

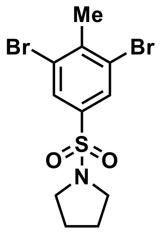

26

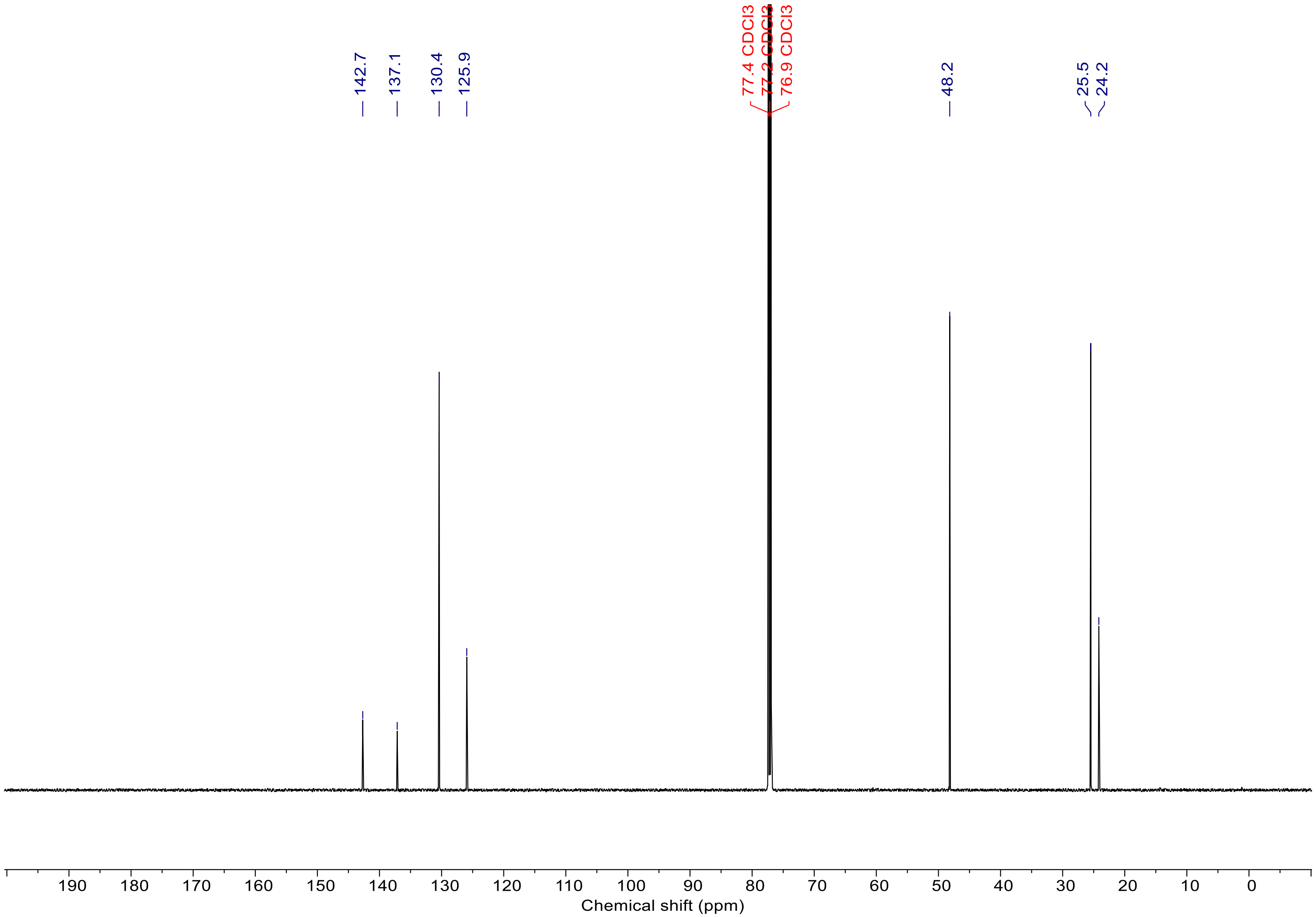

^{1}H NMR spectrum of 4-(pyrrolidin-1-ylsulfonyl)difluoromethoxybenzene (27)

$CDCl_3$, 23°C

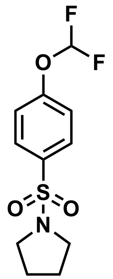

27

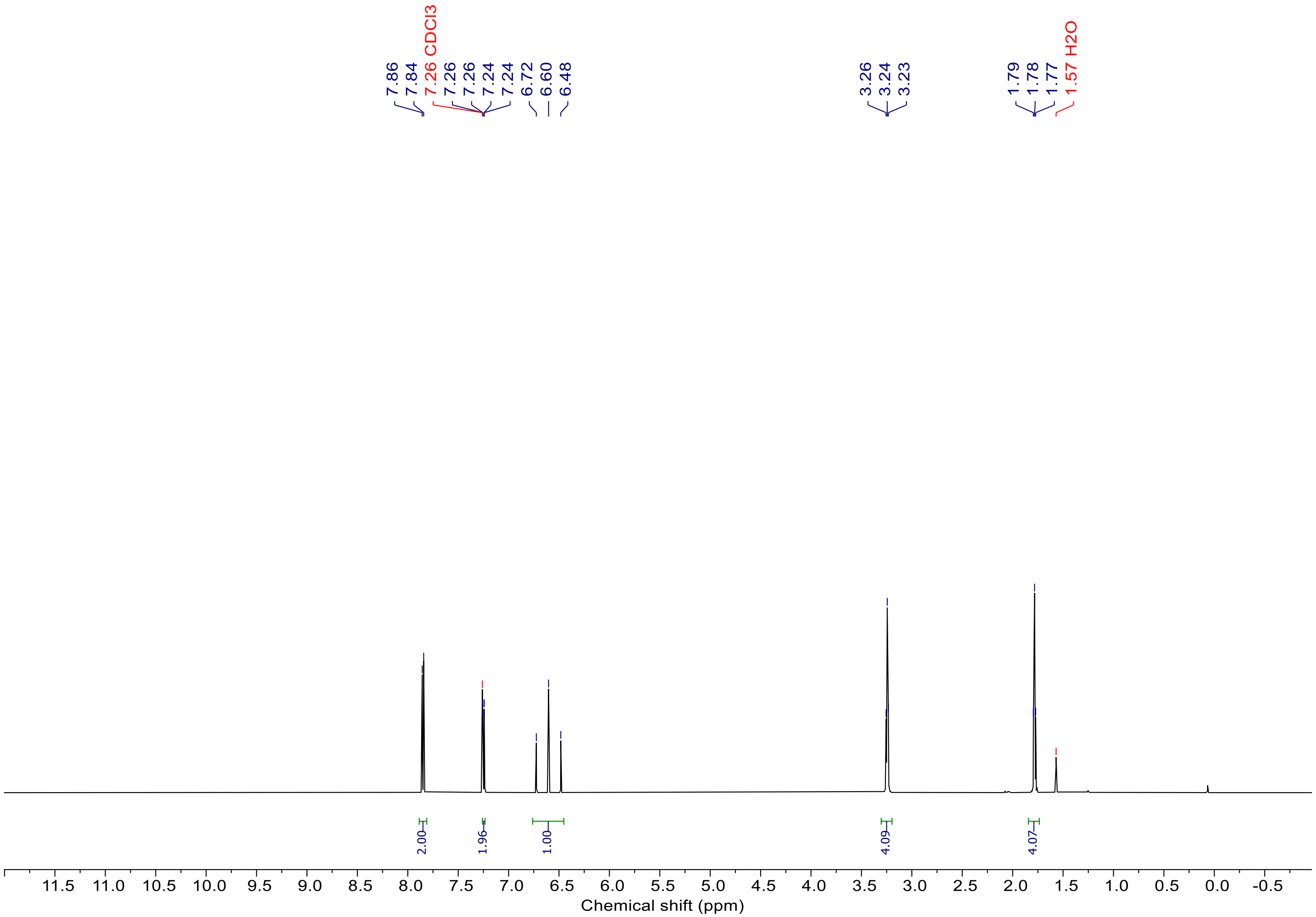

^{13}C NMR spectrum of 4-(pyrrolidin-1-ylsulfonyl)difluoromethoxybenzene (27)

CDCl$_3$, 23°C

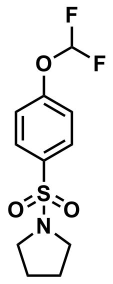

27

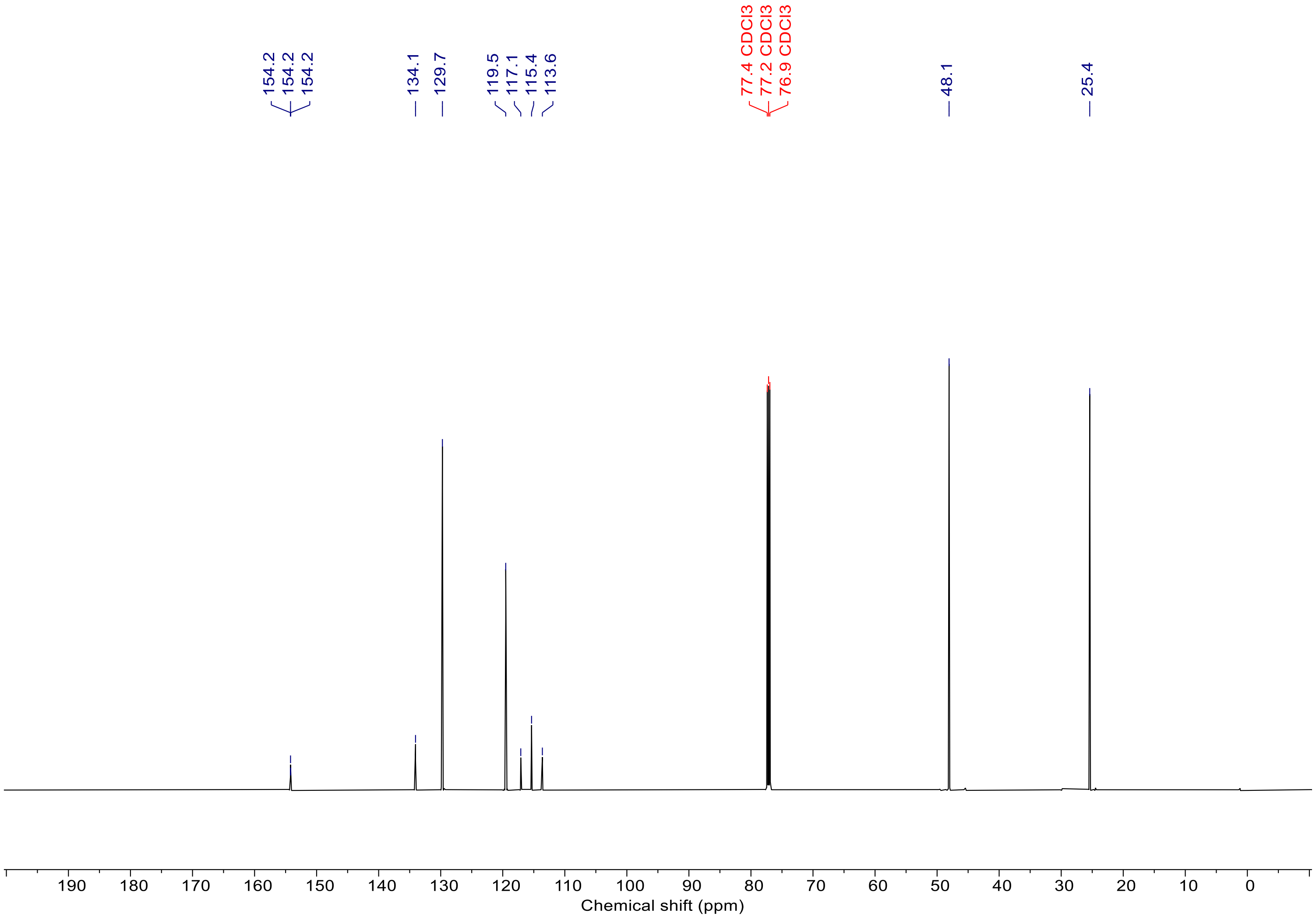

^{19}F NMR spectrum of 4-(pyrrolidin-1-ylsulfonyl)difluoromethoxybenzene (27)

$CDCl_3$, 23°C

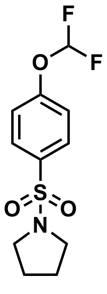

27

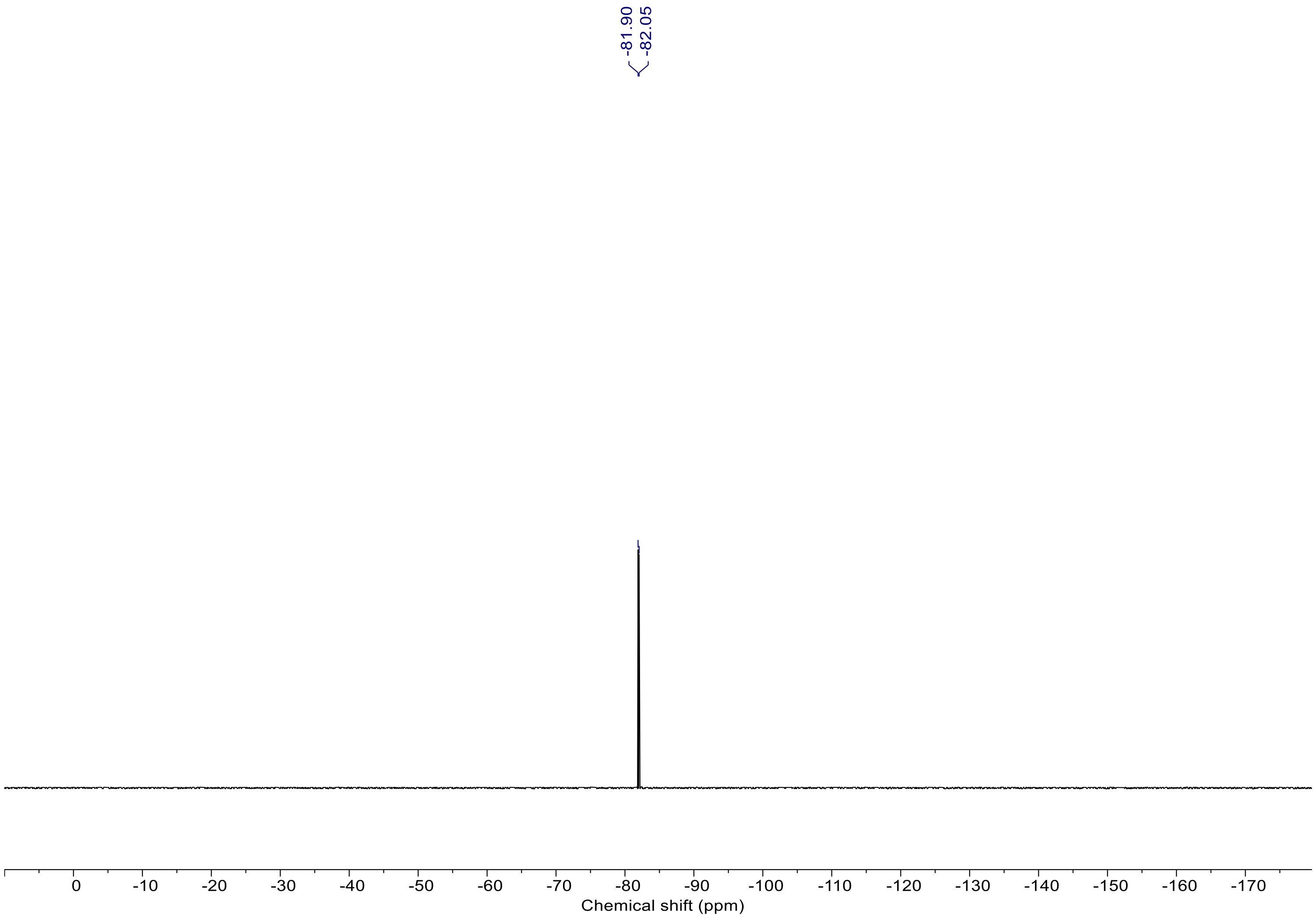

^{1}H NMR spectrum of 4-methyl-7-(pyrrolidin-1-ylsulfonyl)-2*H*-chromen-2-one (28)

$CDCl_3$, 23°C

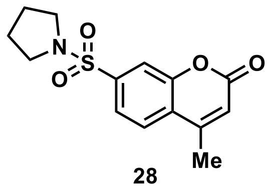

28

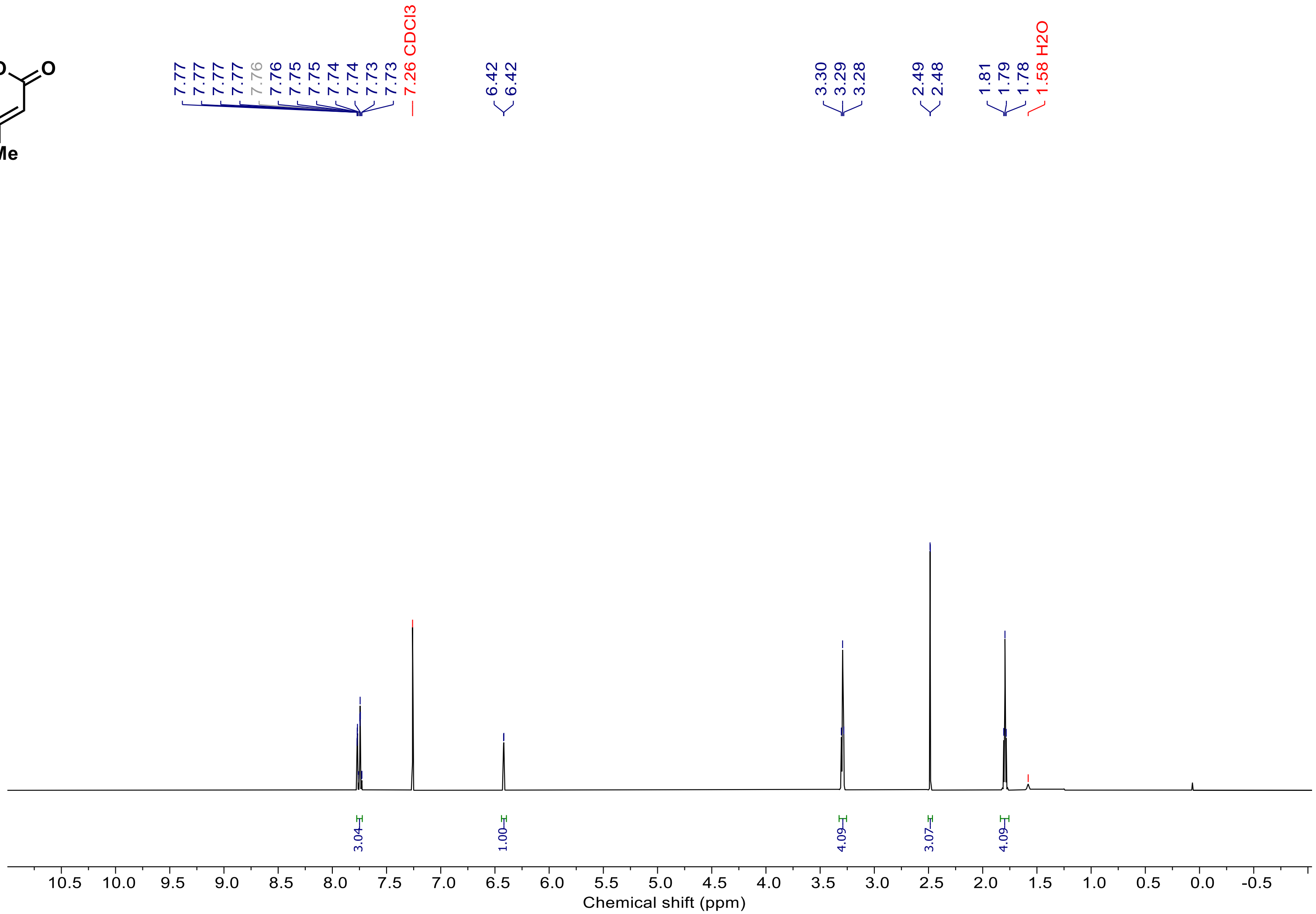

^{13}C NMR spectrum of 4-methyl-7-(pyrrolidin-1-ylsulfonyl)-2*H*-chromen-2-one (28)

$CDCl_3$, 23°C

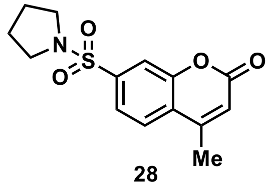

28

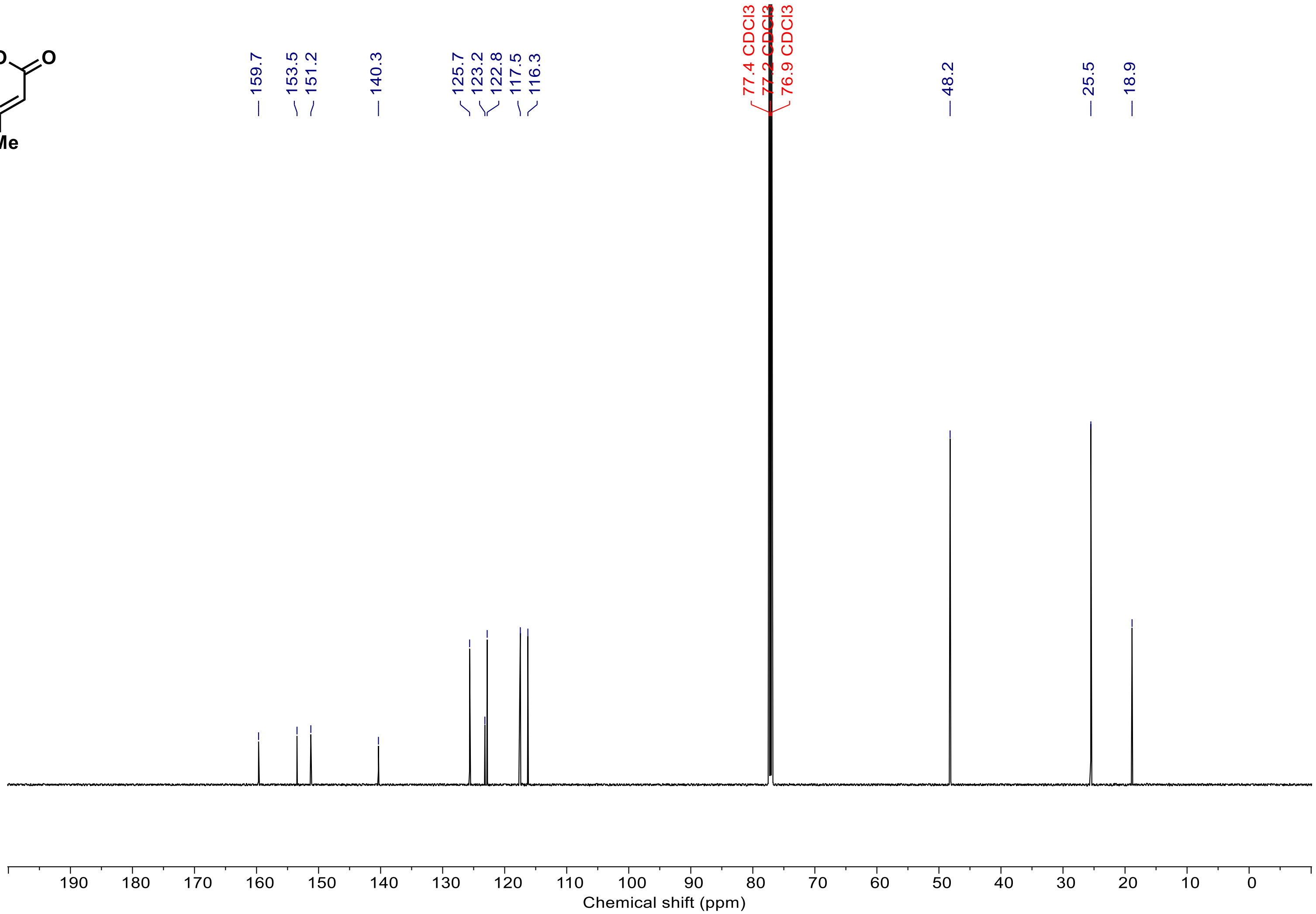

^{1}H NMR spectrum of methyl 3-(pyrrolidin-1-ylsulfonyl)thiophene-2-carboxylate (29)

$CDCl_3$, 23°C

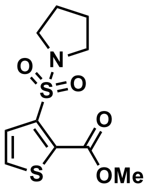

29

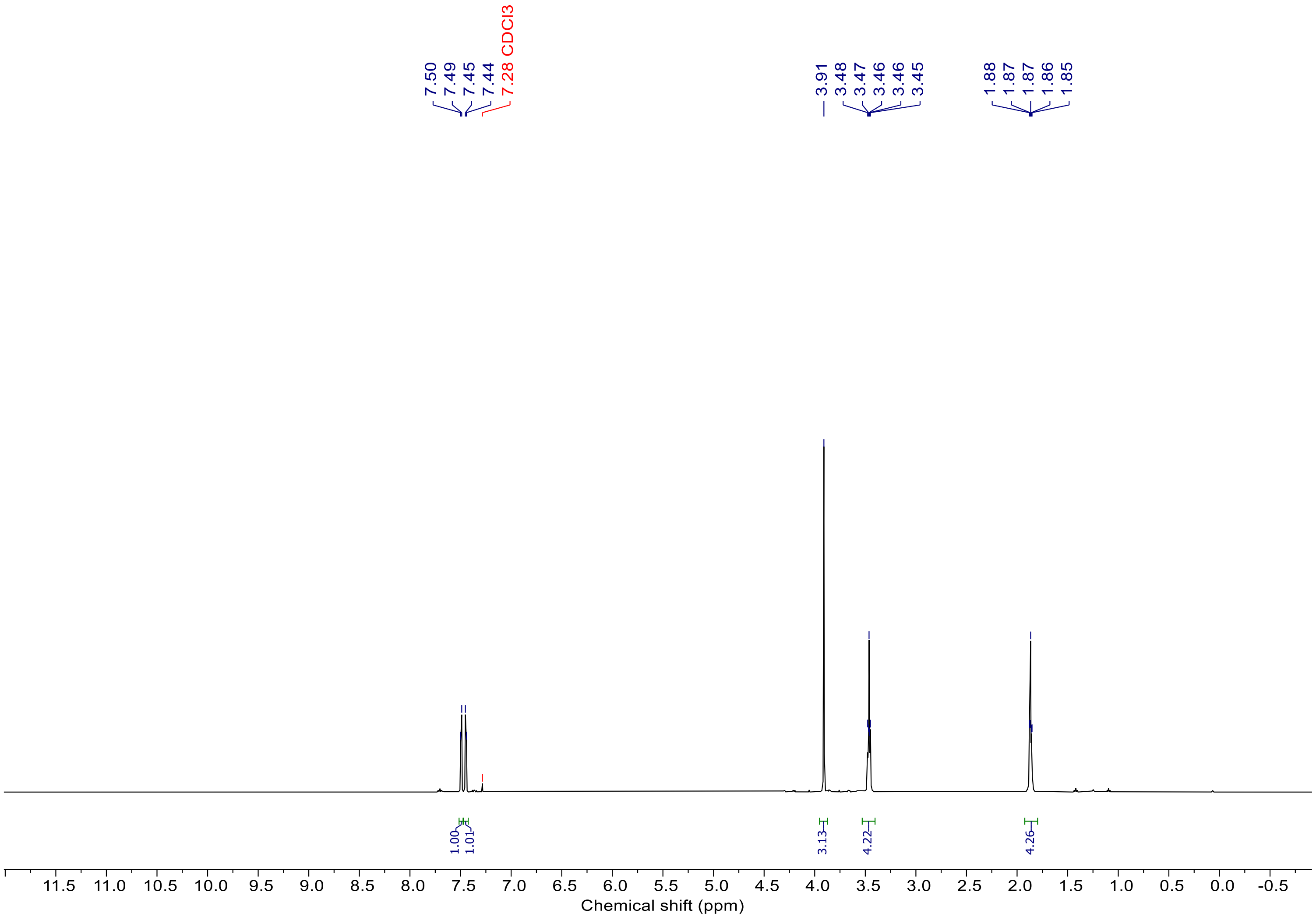

^{13}C NMR spectrum of methyl 3-(pyrrolidin-1-ylsulfonyl)thiophene-2-carboxylate (29)

$CDCl_3$, 23°C

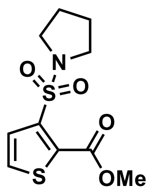

29

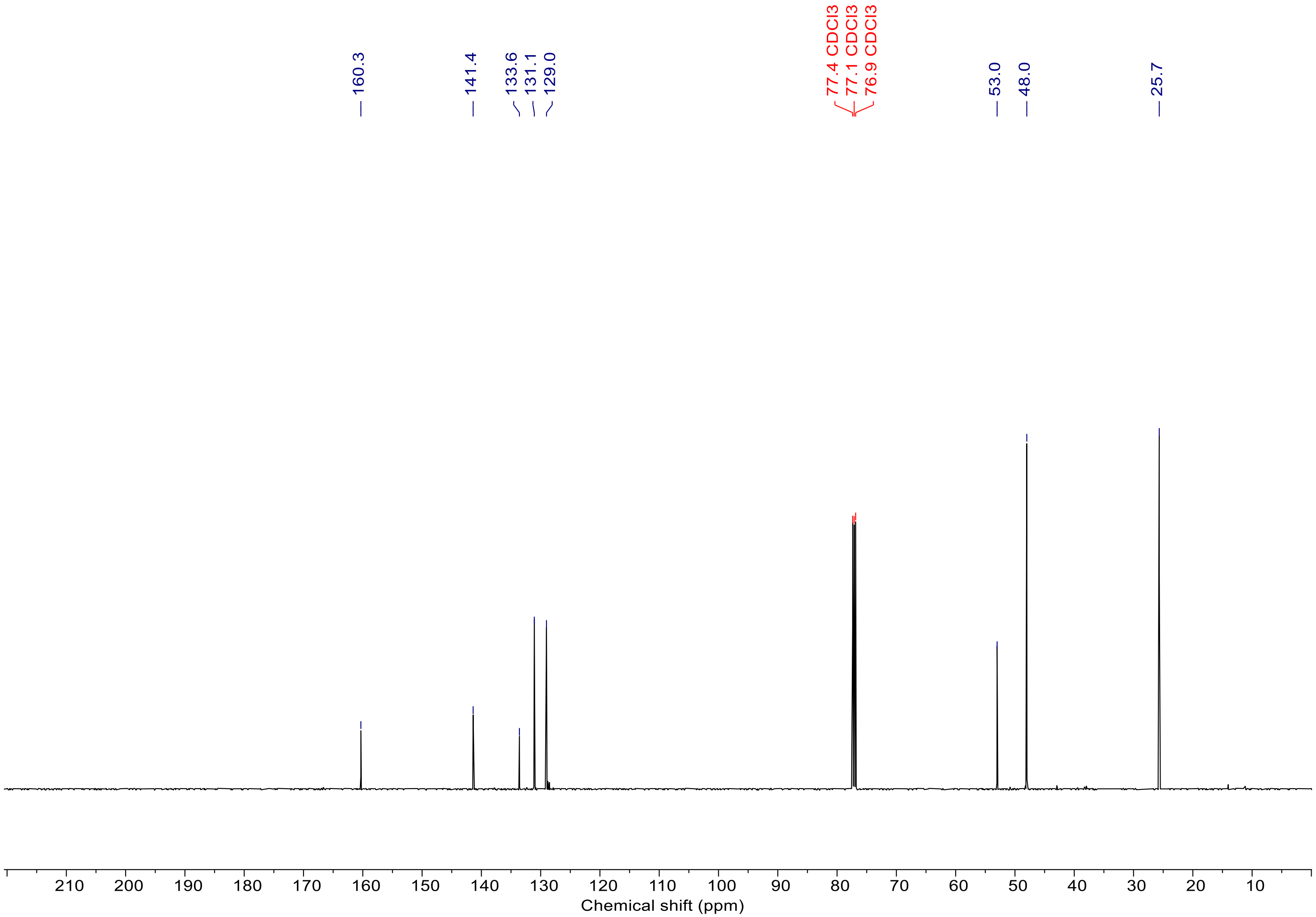

^{1}H NMR spectrum of 1-((2,2-difluorobenzo[d][1,3]dioxol-4-yl)sulfonyl)pyrrolidine (30)

$CDCl_3$, 23°C

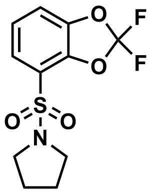

30

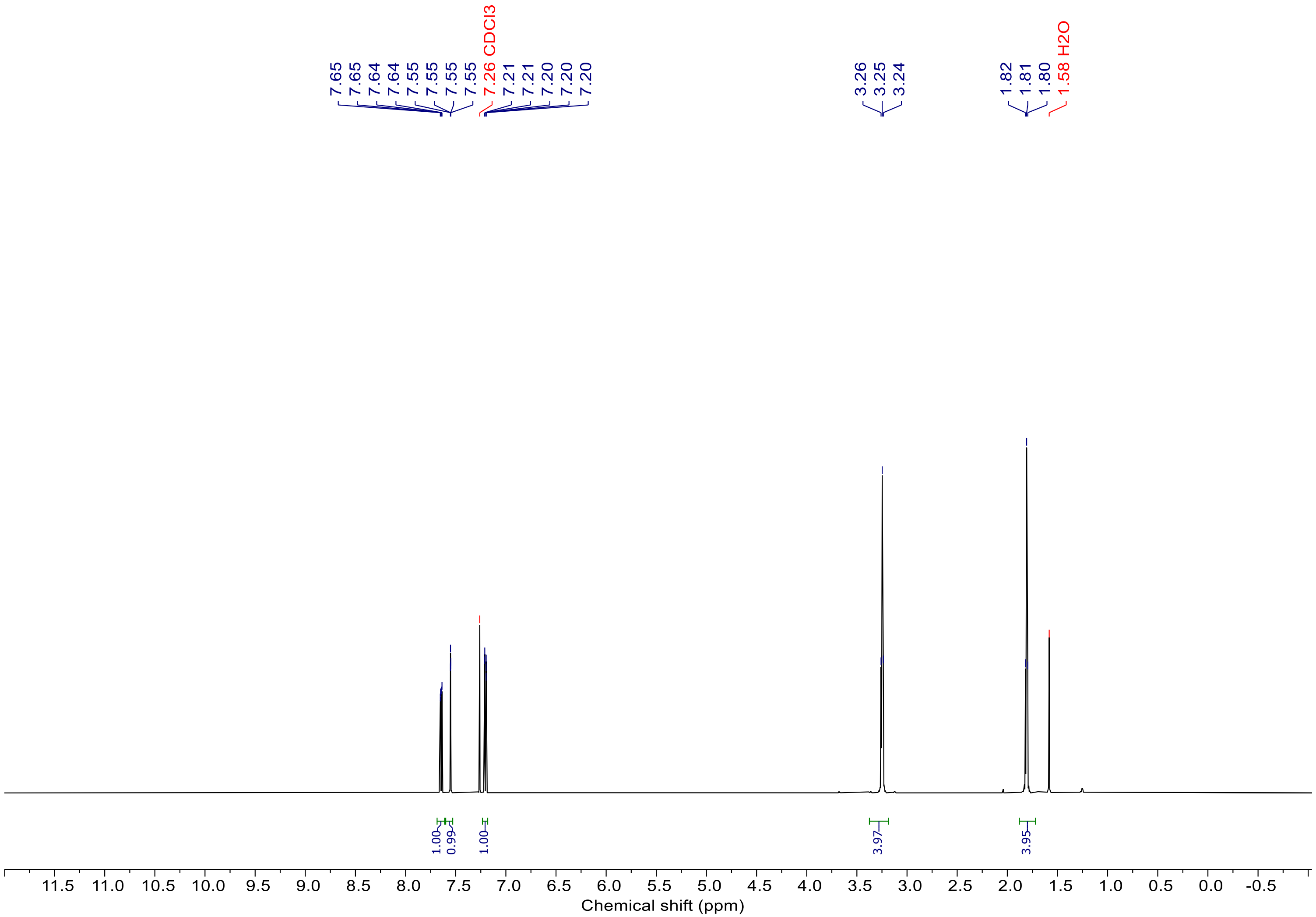

^{13}C NMR spectrum of 1-((2,2-difluorobenzo[d][1,3]dioxol-4-yl)sulfonyl)pyrrolidine (30)

$CDCl_3$, 23°C

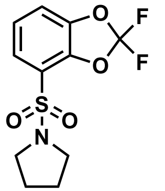

30

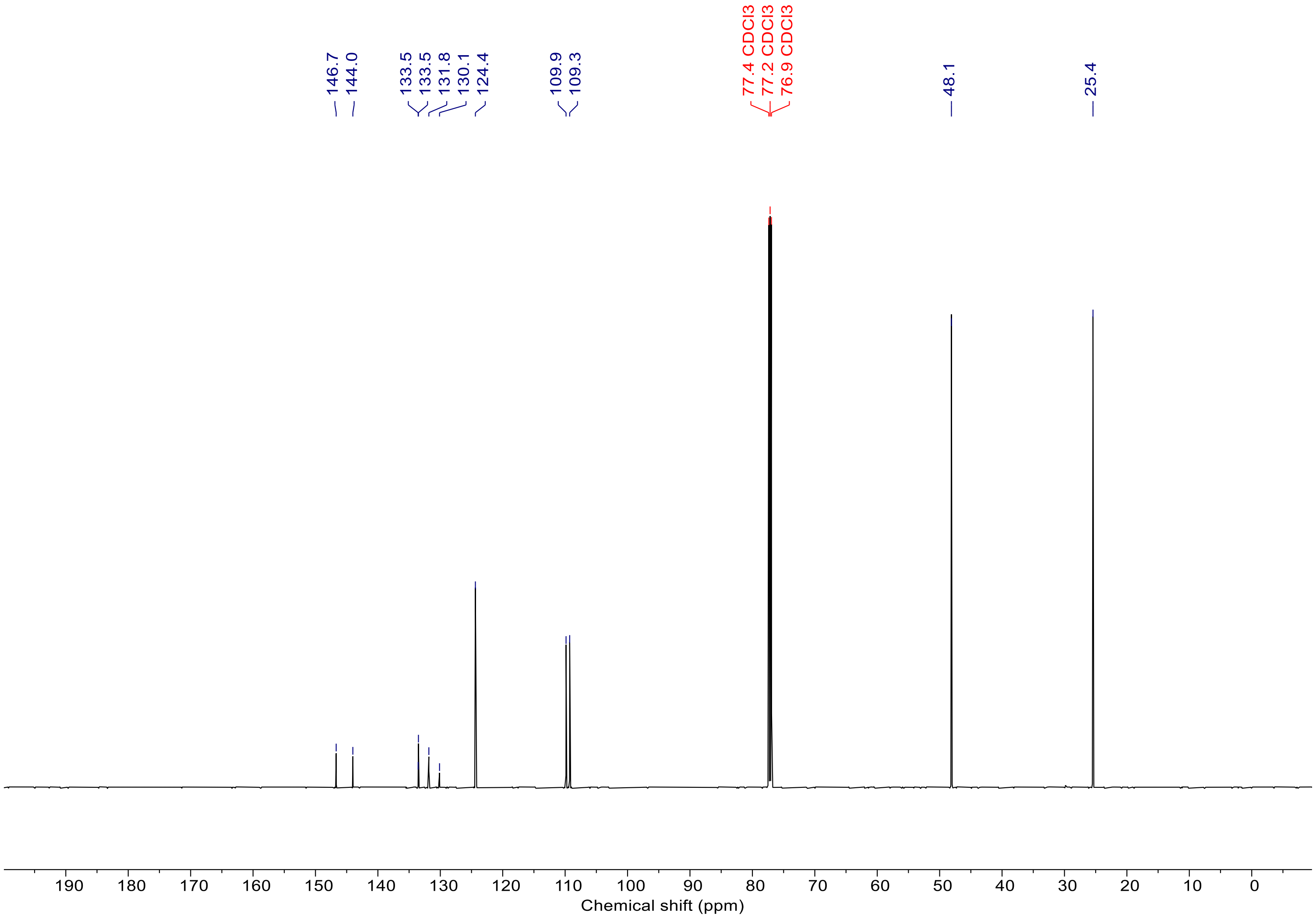

^{19}F NMR spectrum of 1-((2,2-difluorobenzo[d][1,3]dioxol-4-yl)sulfonyl)pyrrolidine (30)

CDCl$_3$, 23°C

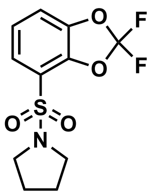

30

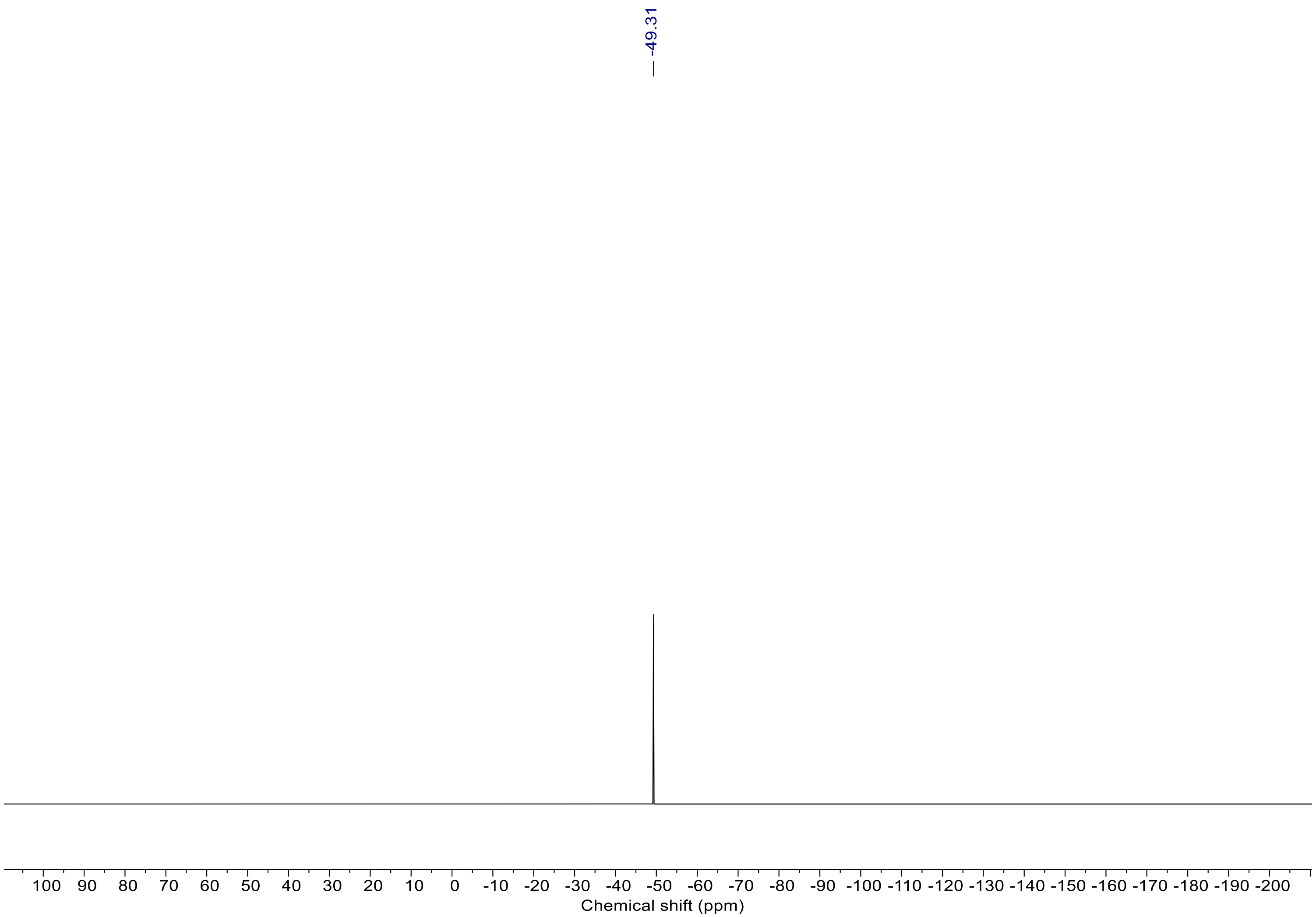

^{1}H NMR spectrum of hippuric acid-derived sulfonamide 31

DMSO-d_6, 23°C

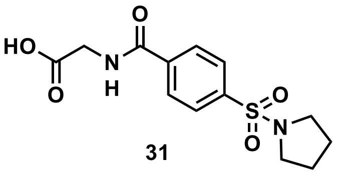

31

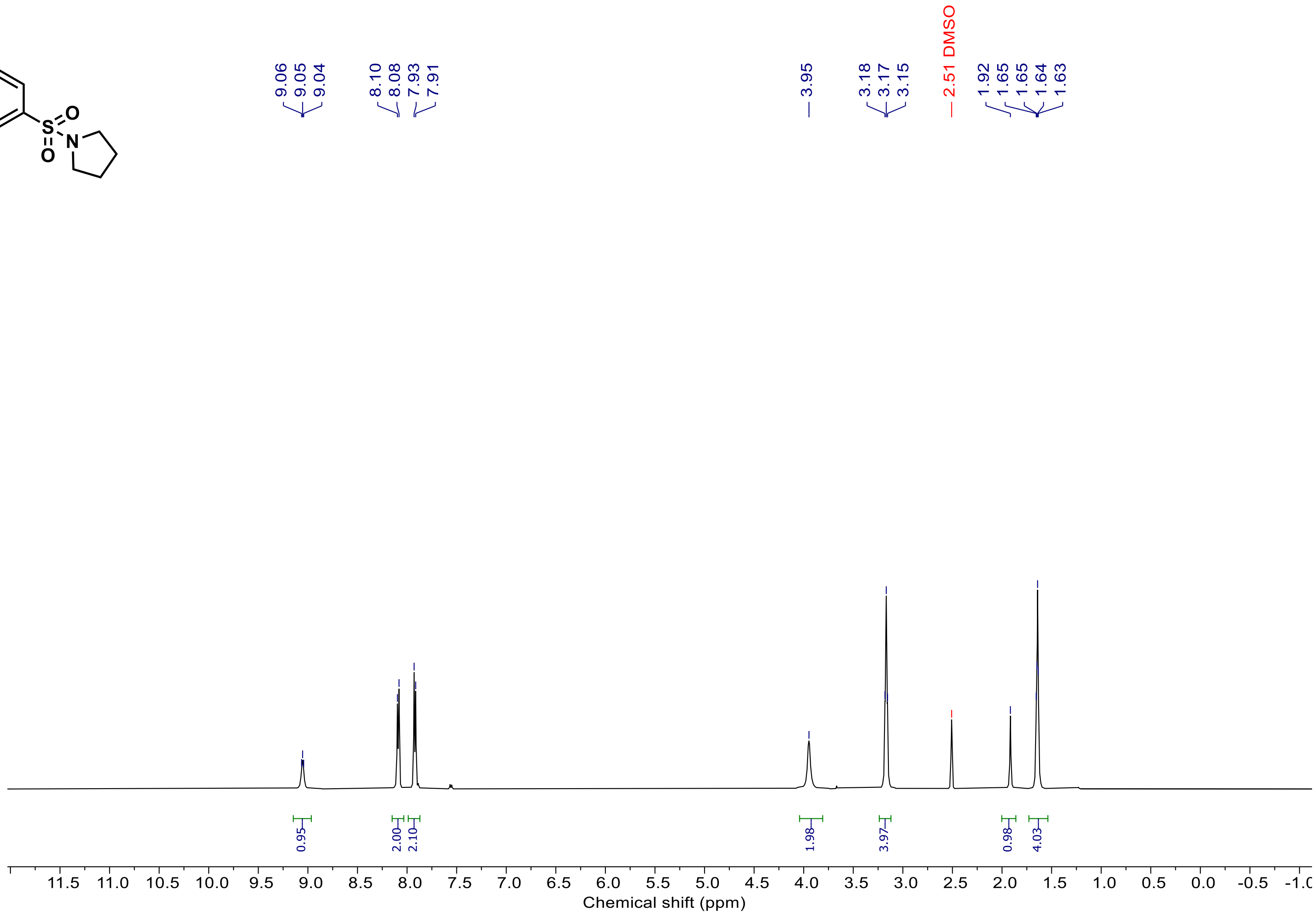

^{13}C NMR spectrum of hippuric acid-derived sulfonamide 31

DMSO-d_6, 23°C

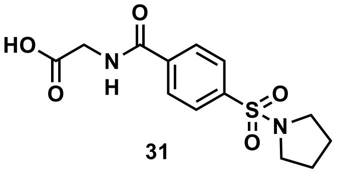

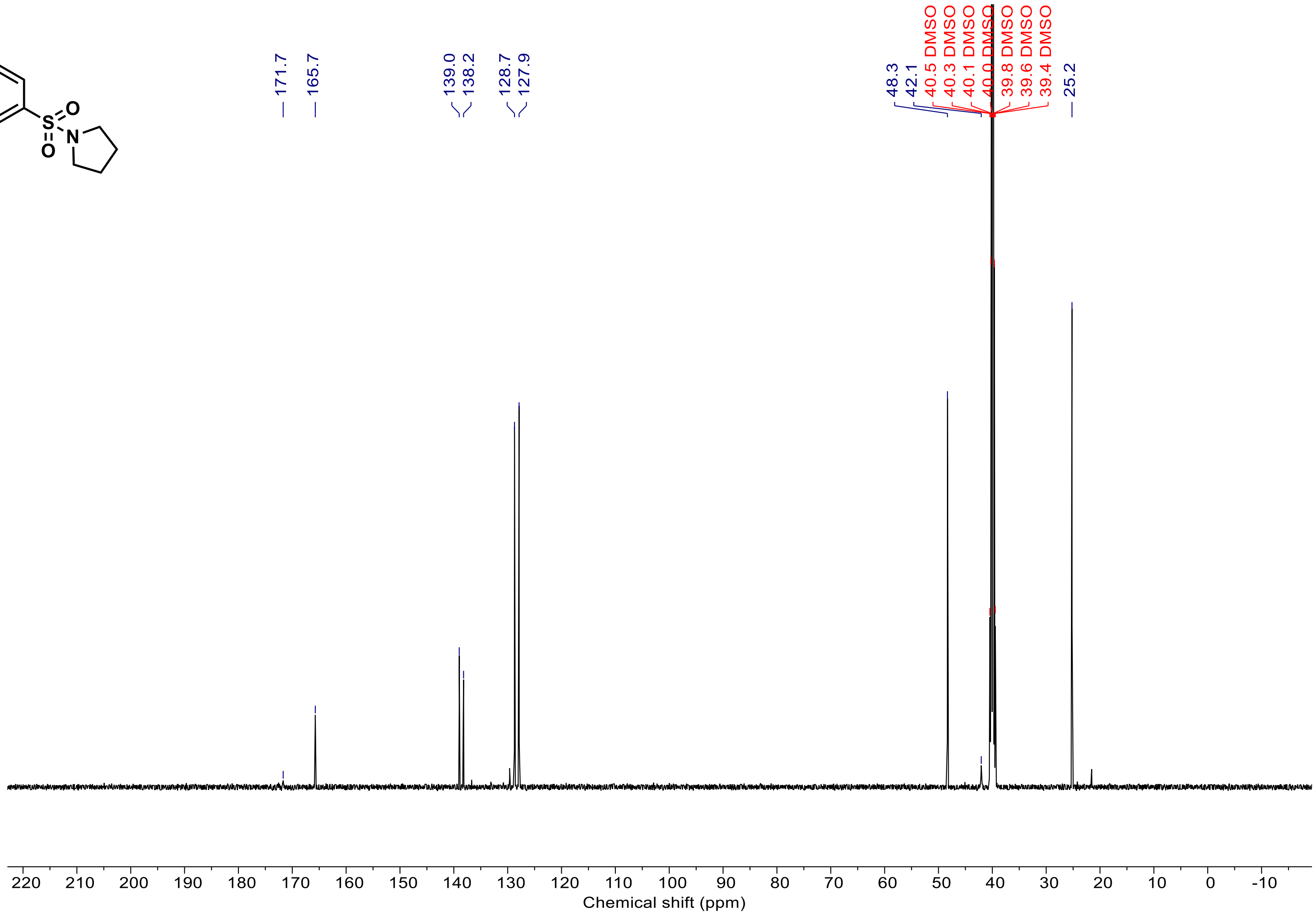

^{1}H NMR spectrum of Flutamide-derived sulfonamide 32

CDCl$_3$, 23°C

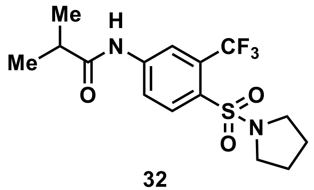

32

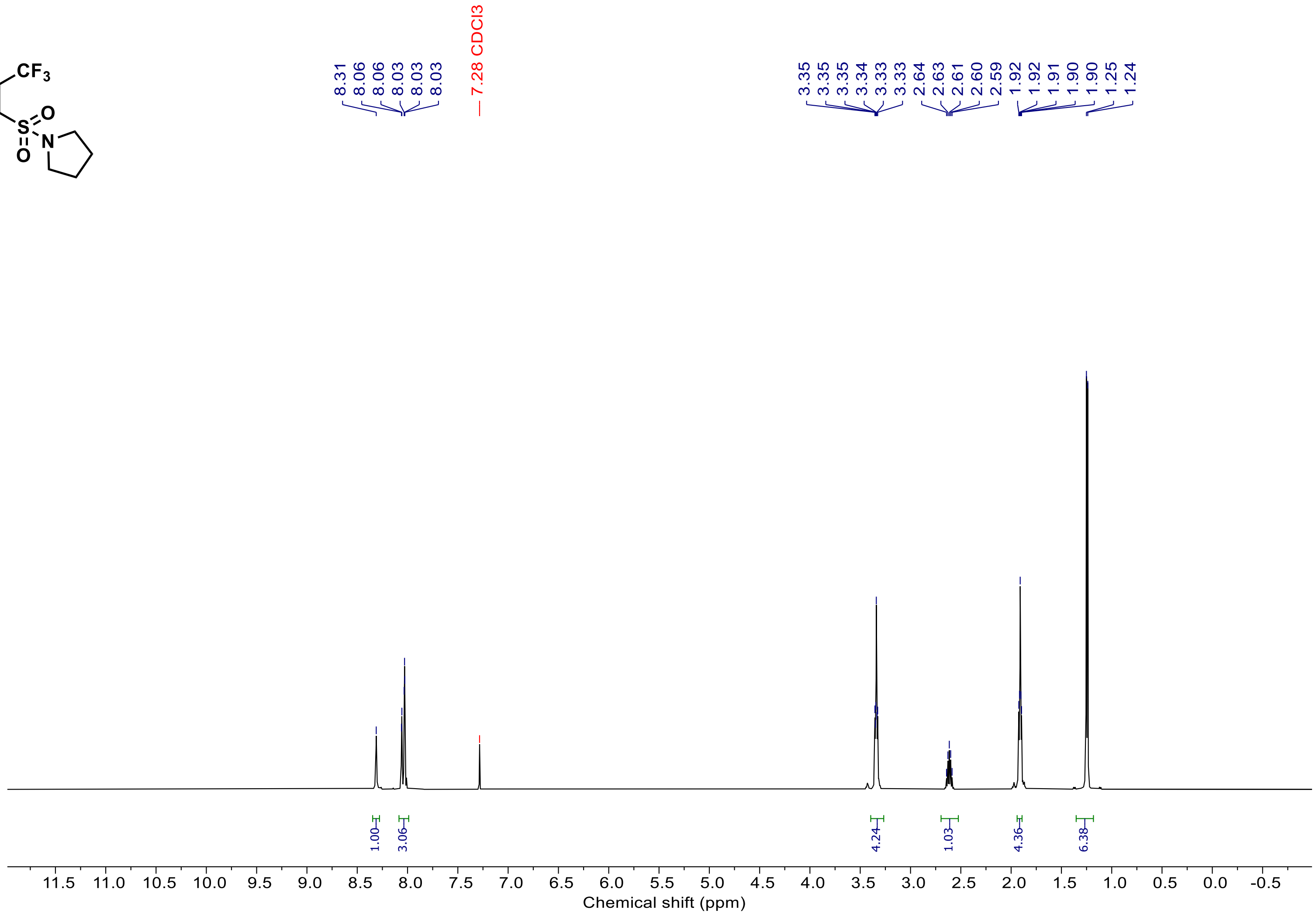

^{13}C NMR spectrum of Flutamide-derived sulfonamide 32

CDCl$_3$, 23°C

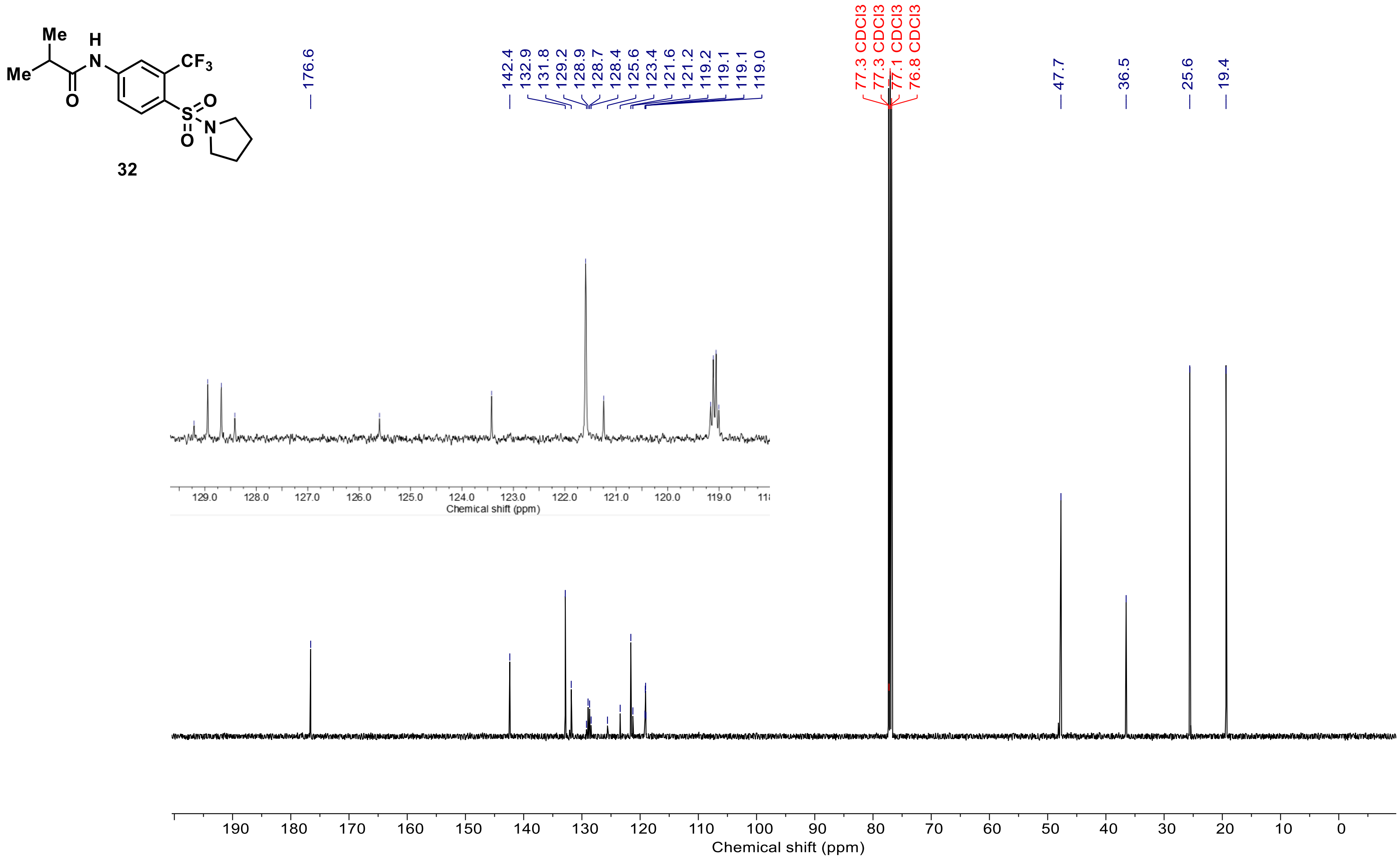

^{19}F NMR spectrum of Flutamide-derived sulfonamide 32

CDCl$_3$, 23°C

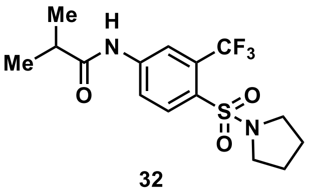

32

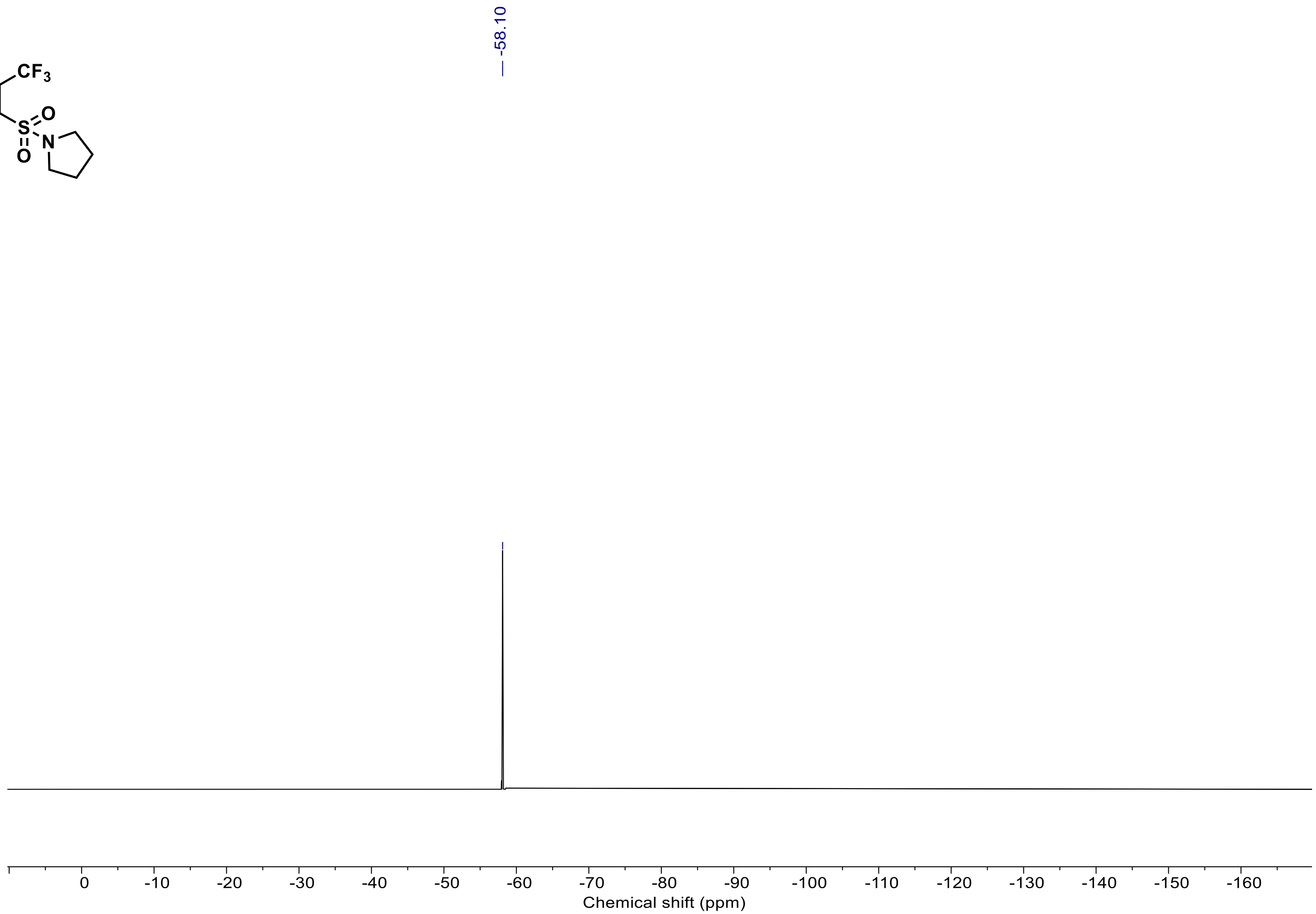

^{1}H NMR spectrum of 5-(pyrrolidin-1-ylsulfonyl)picolinonitrile (33)

DMSO-d_6, 23°C

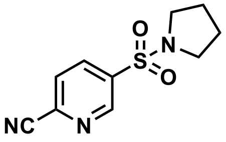

33

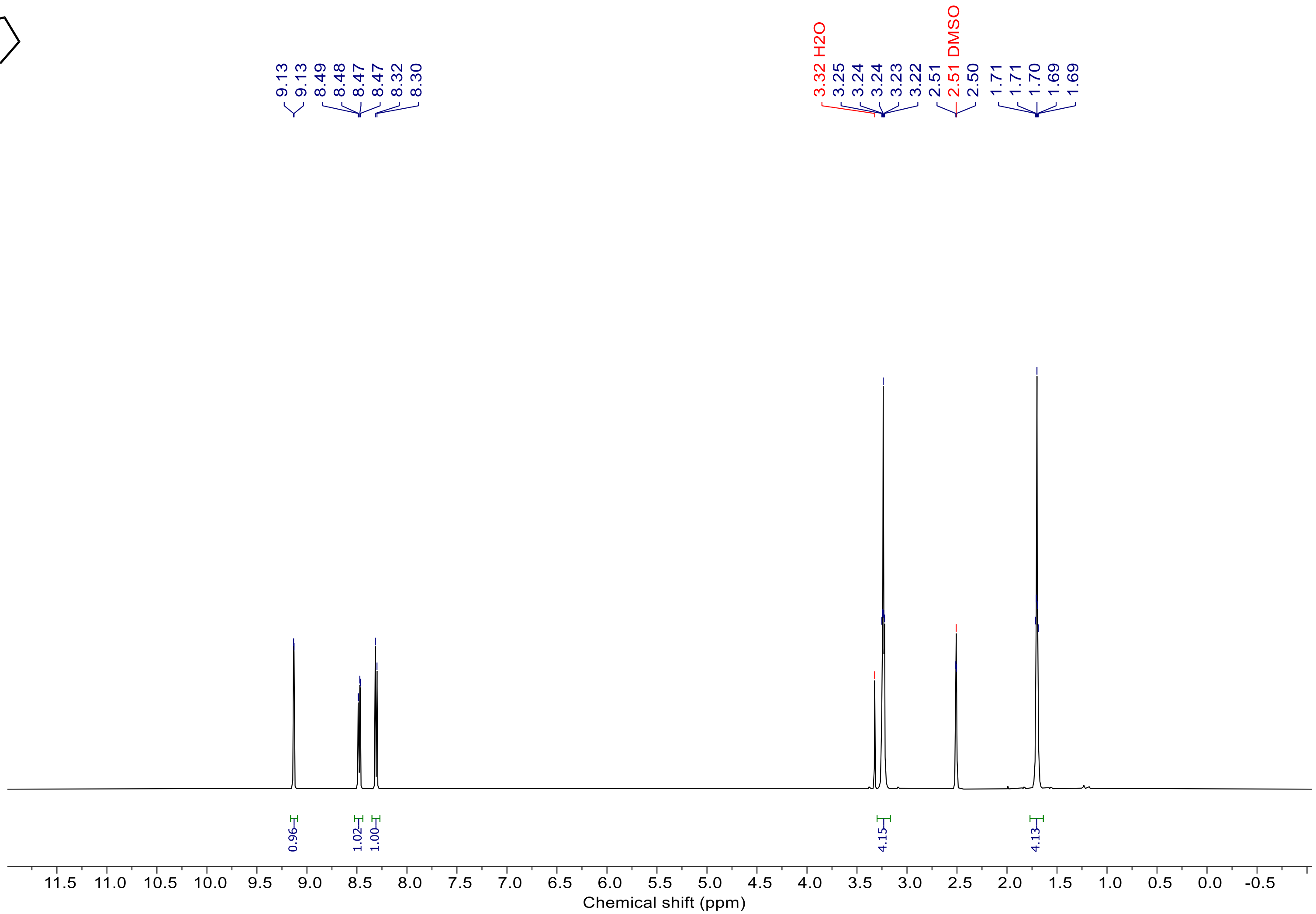

^{13}C NMR spectrum of 5-(pyrrolidin-1-ylsulfonyl)picolinonitrile (33)

DMSO-d_6, 23°C

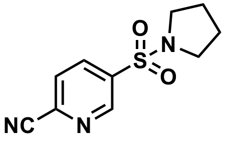

33

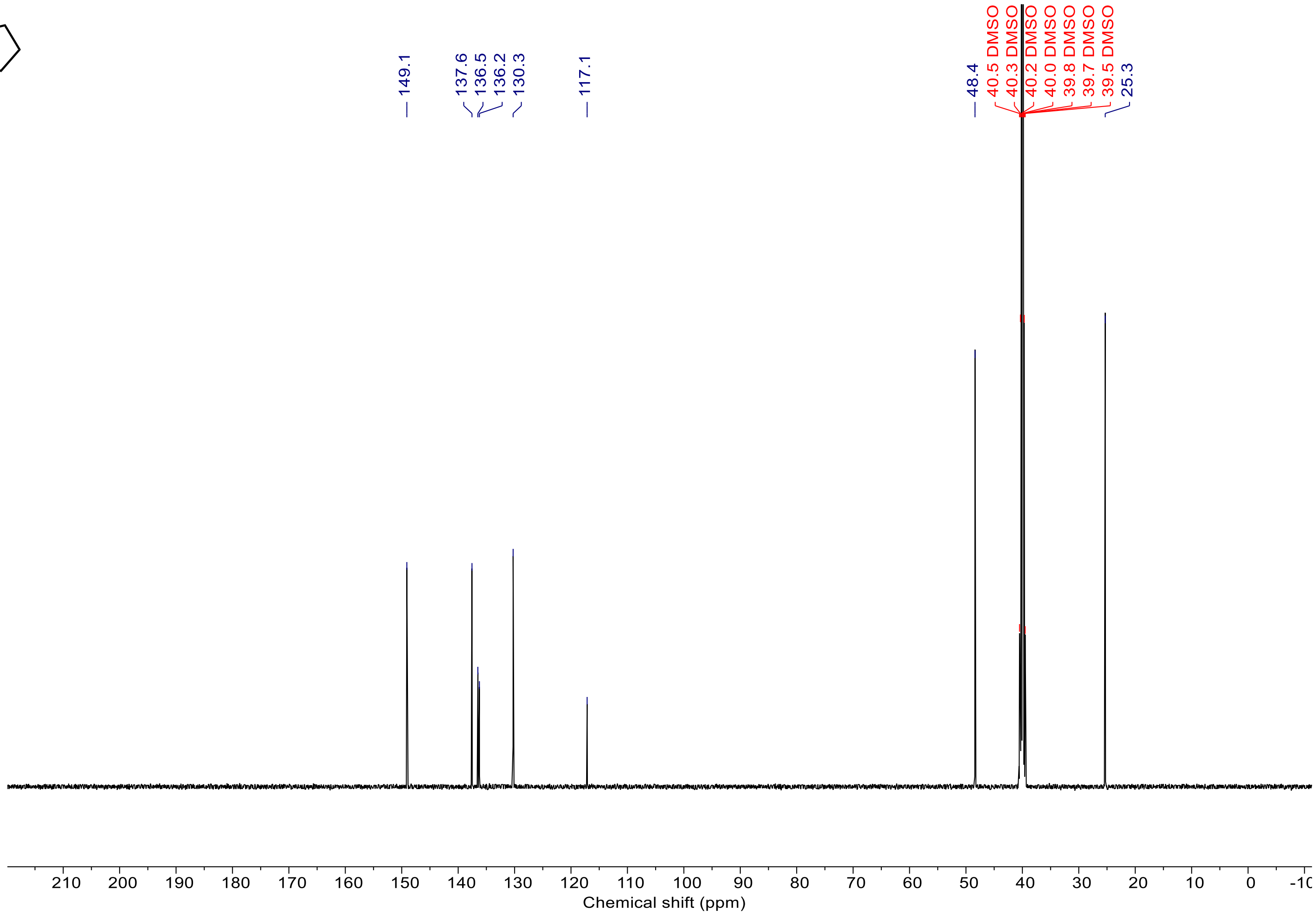

^{1}H NMR spectrum of Sulfadoxin-derived sulfonyl chloride (34a)

DMSO-d_6, 23°C

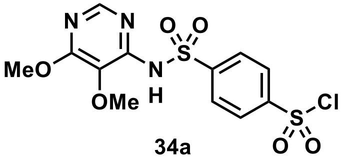

34a

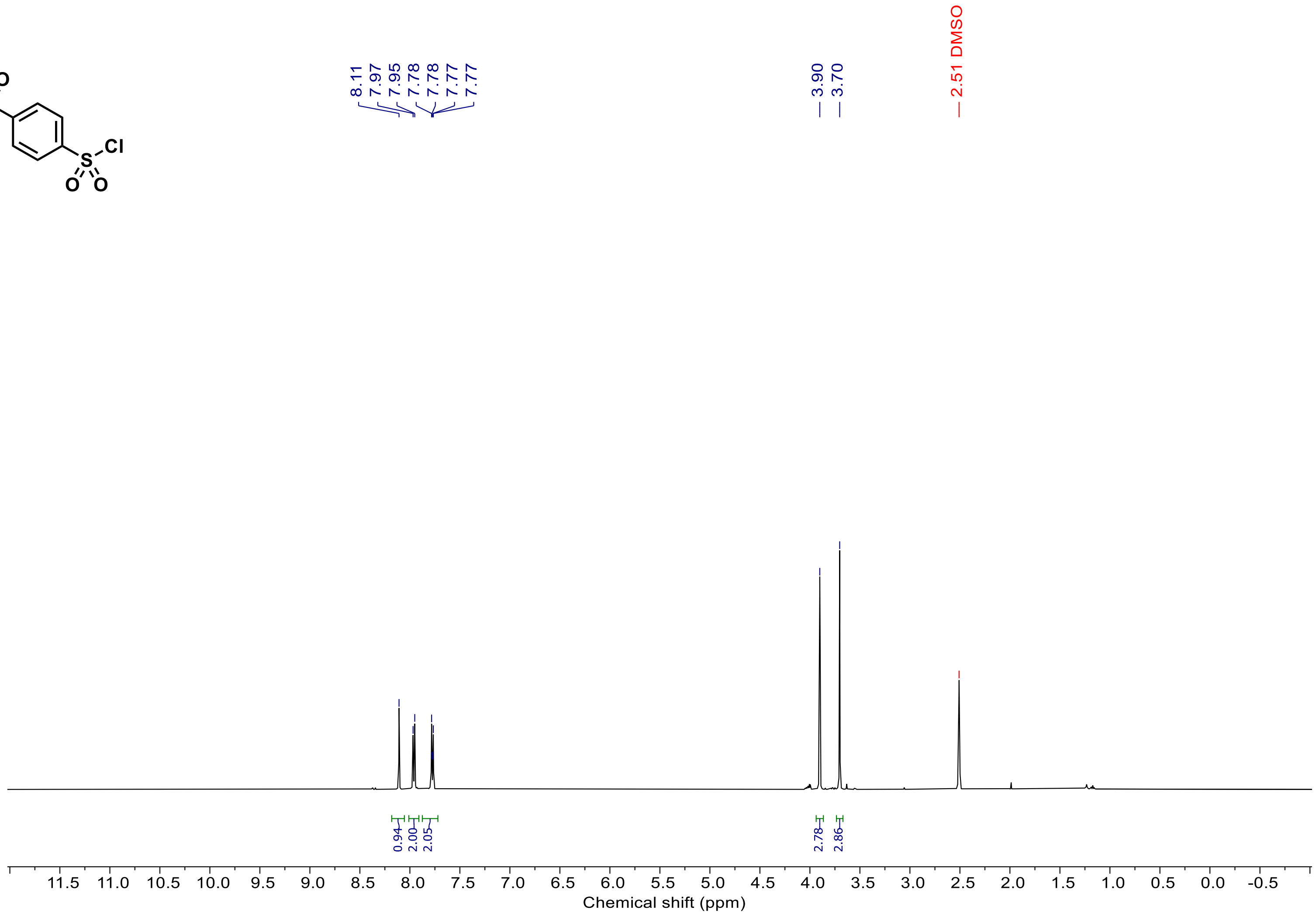

^{13}C NMR spectrum of Sulfadoxin-derived sulfonyl chloride (34a)

DMSO-d_6, 23°C

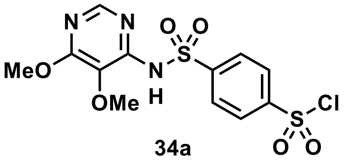

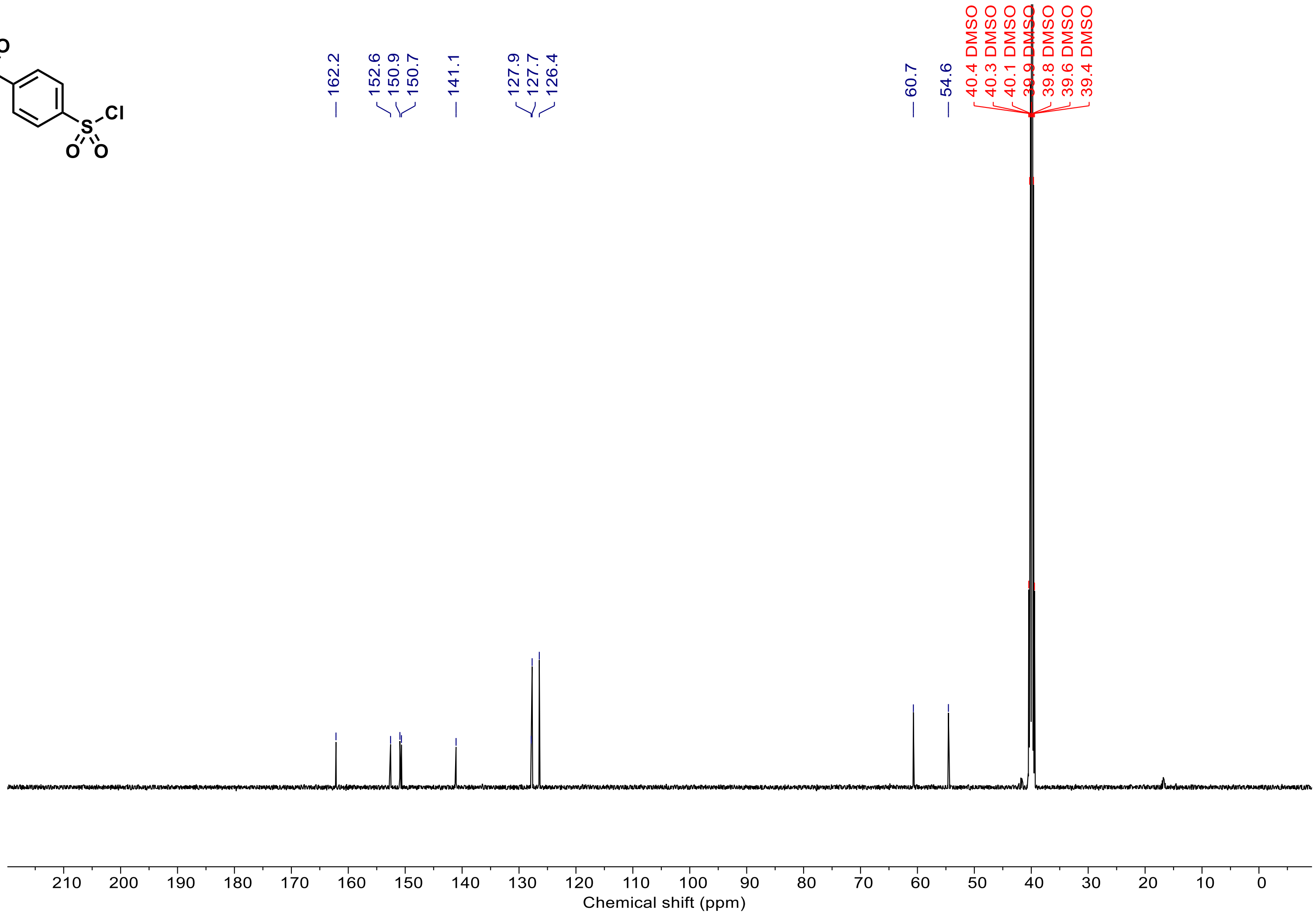

^{1}H NMR spectrum of ethyl-4-fluorobenzoate (35)

$CDCl_3$, 23°C

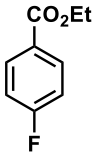

35

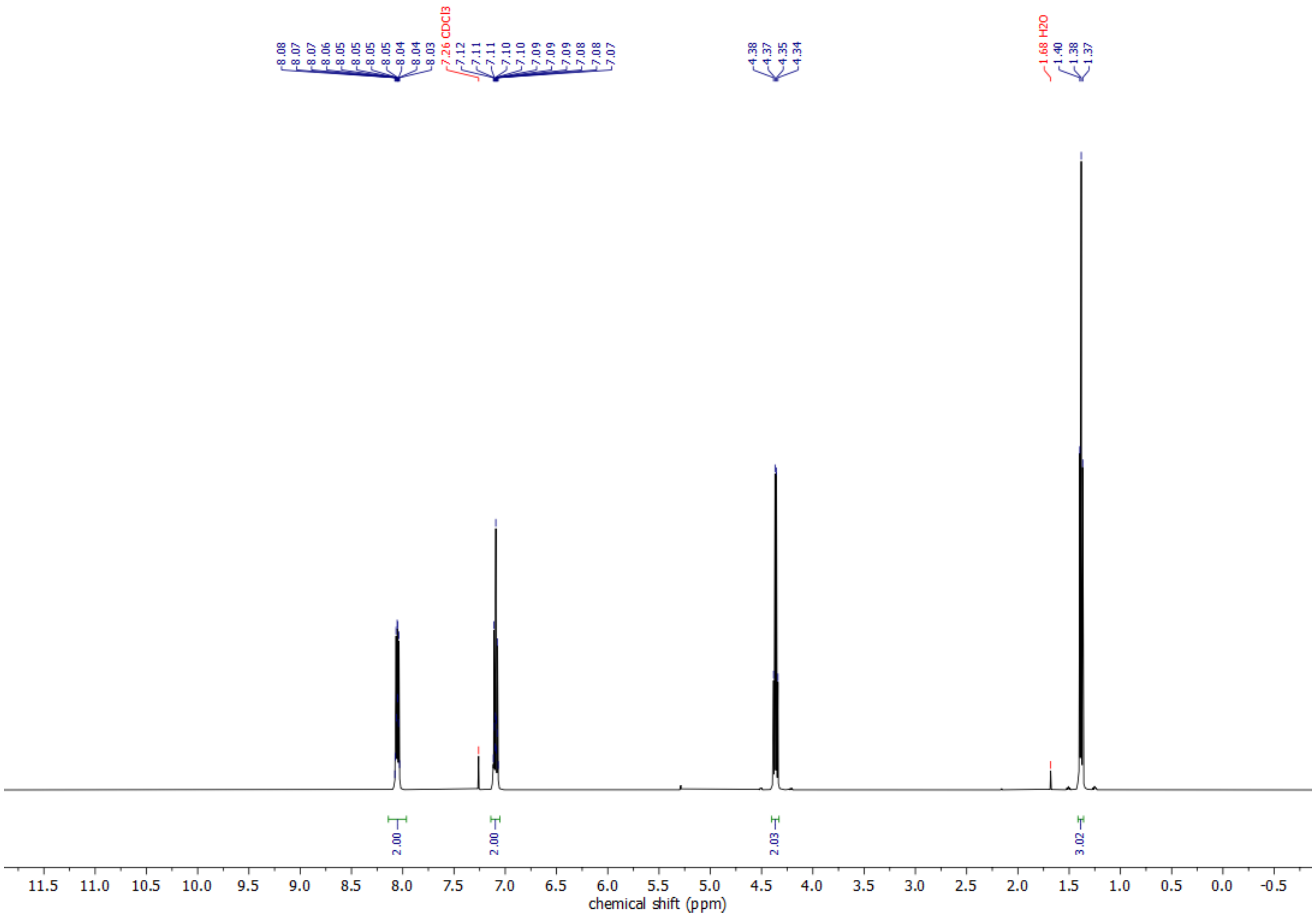

^{13}C NMR spectrum of ethyl-4-fluorobenzoate (35)

$CDCl_3$, 23°C

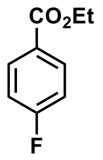

35

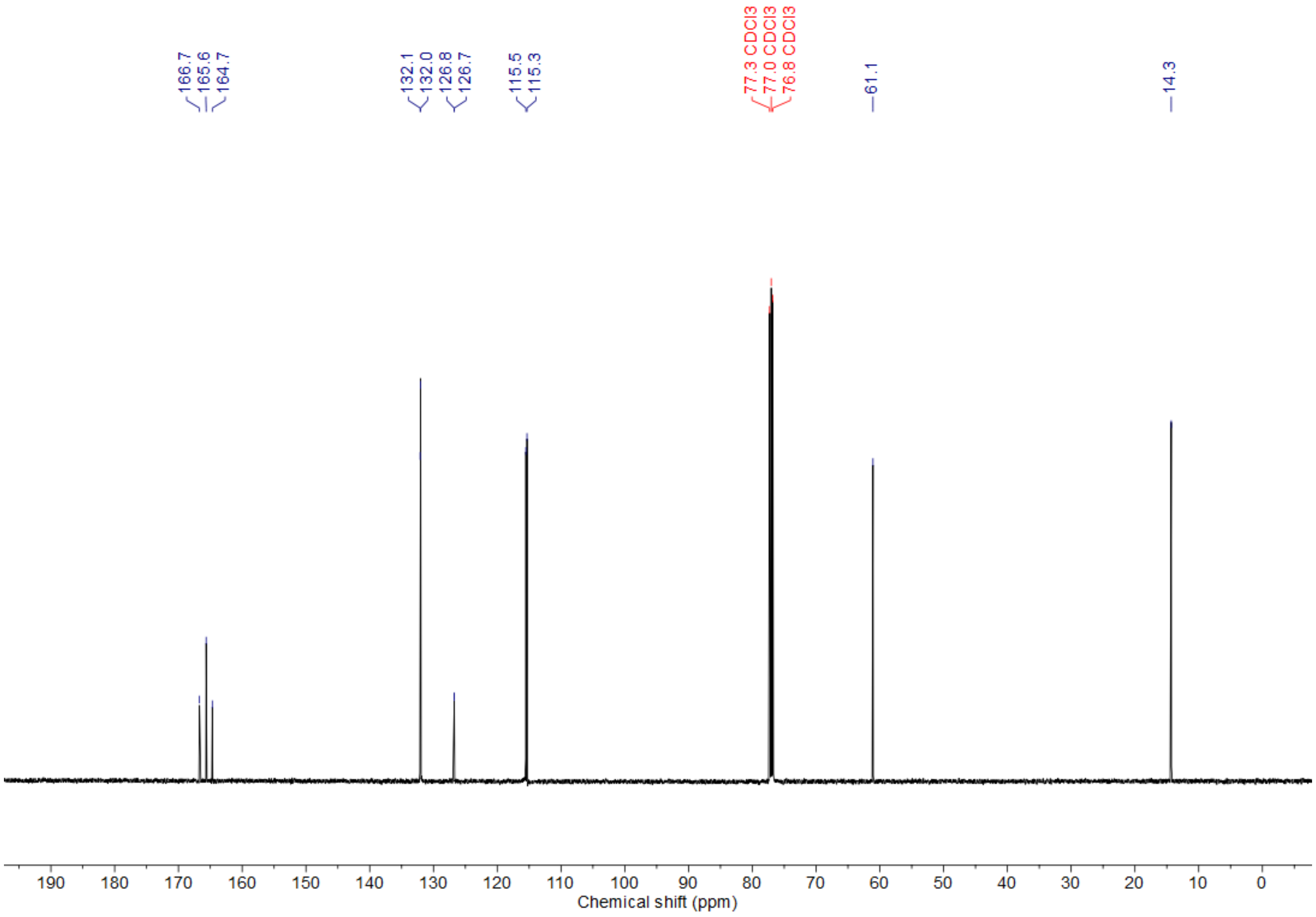

^{19}F NMR spectrum of ethyl-4-fluorobenzoate (35)

$CDCl_3$, 23°C

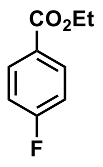

35

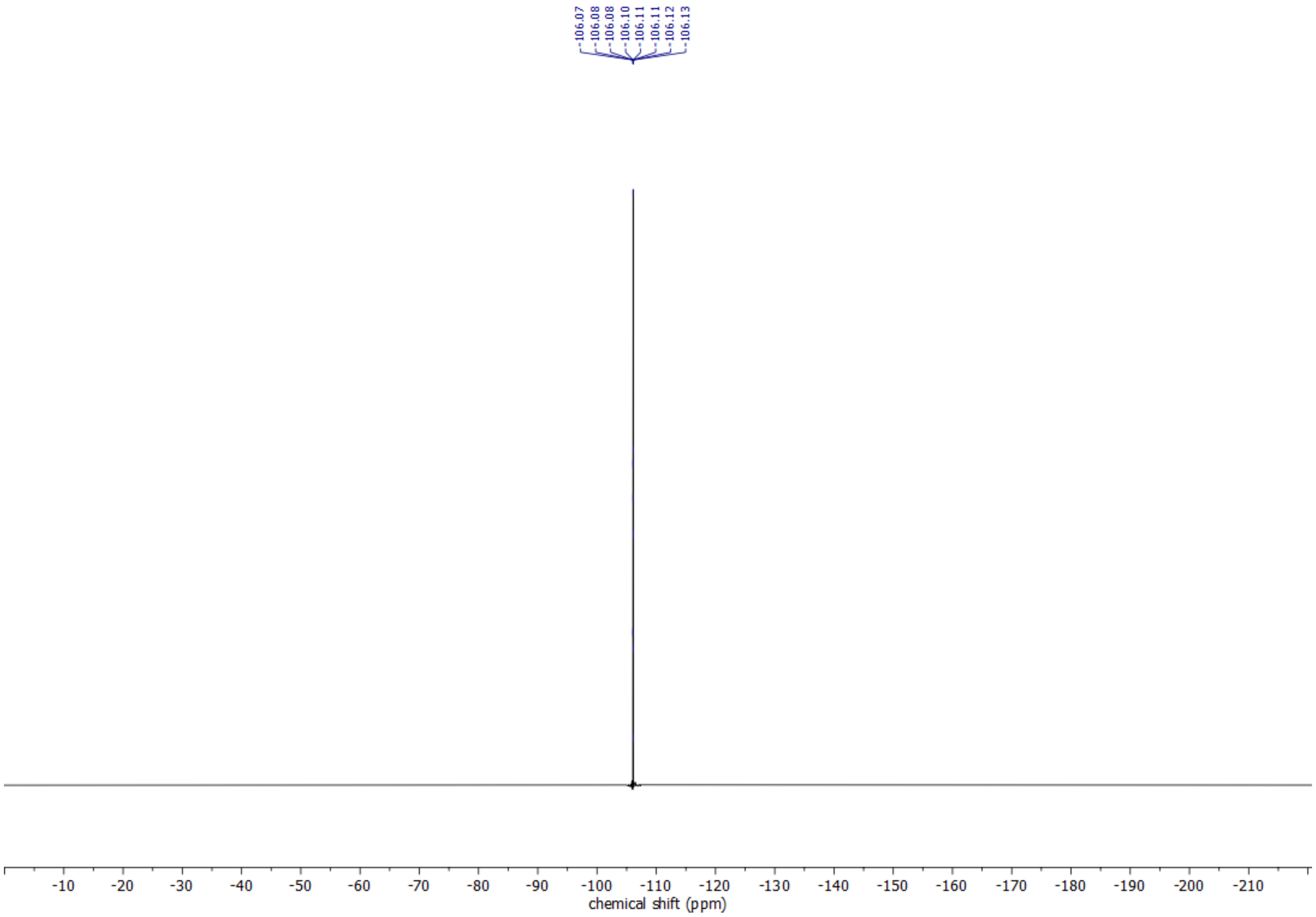

^{1}H NMR spectrum of 4-fluoro-3,5-dimethylbenzonitrile (36)

$CDCl_3$, 23°C

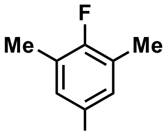

36

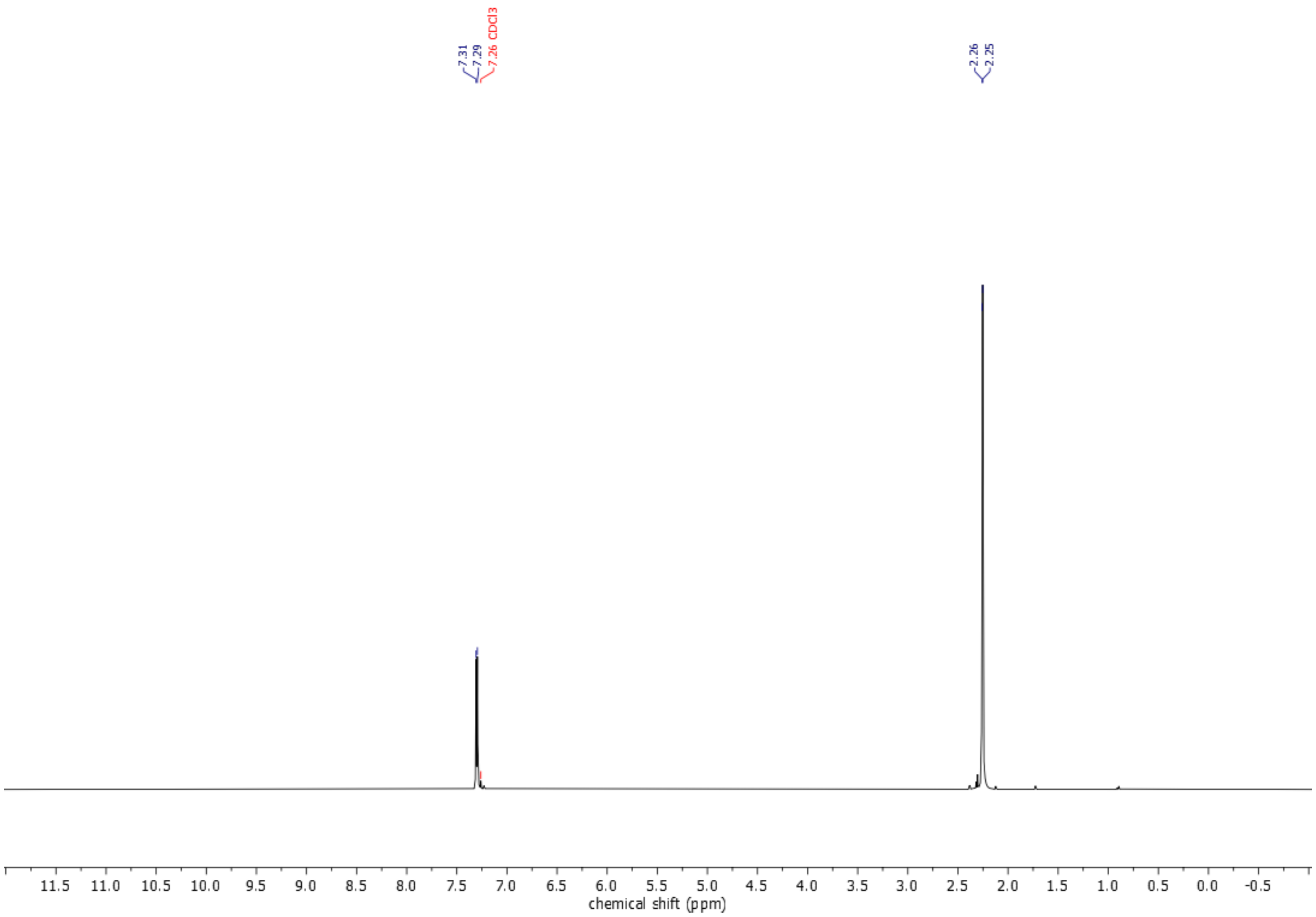

^{13}C NMR spectrum of 4-fluoro-3,5-dimethylbenzonitrile (36)

$CDCl_3$, 23°C

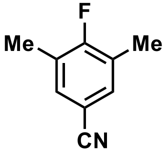

36

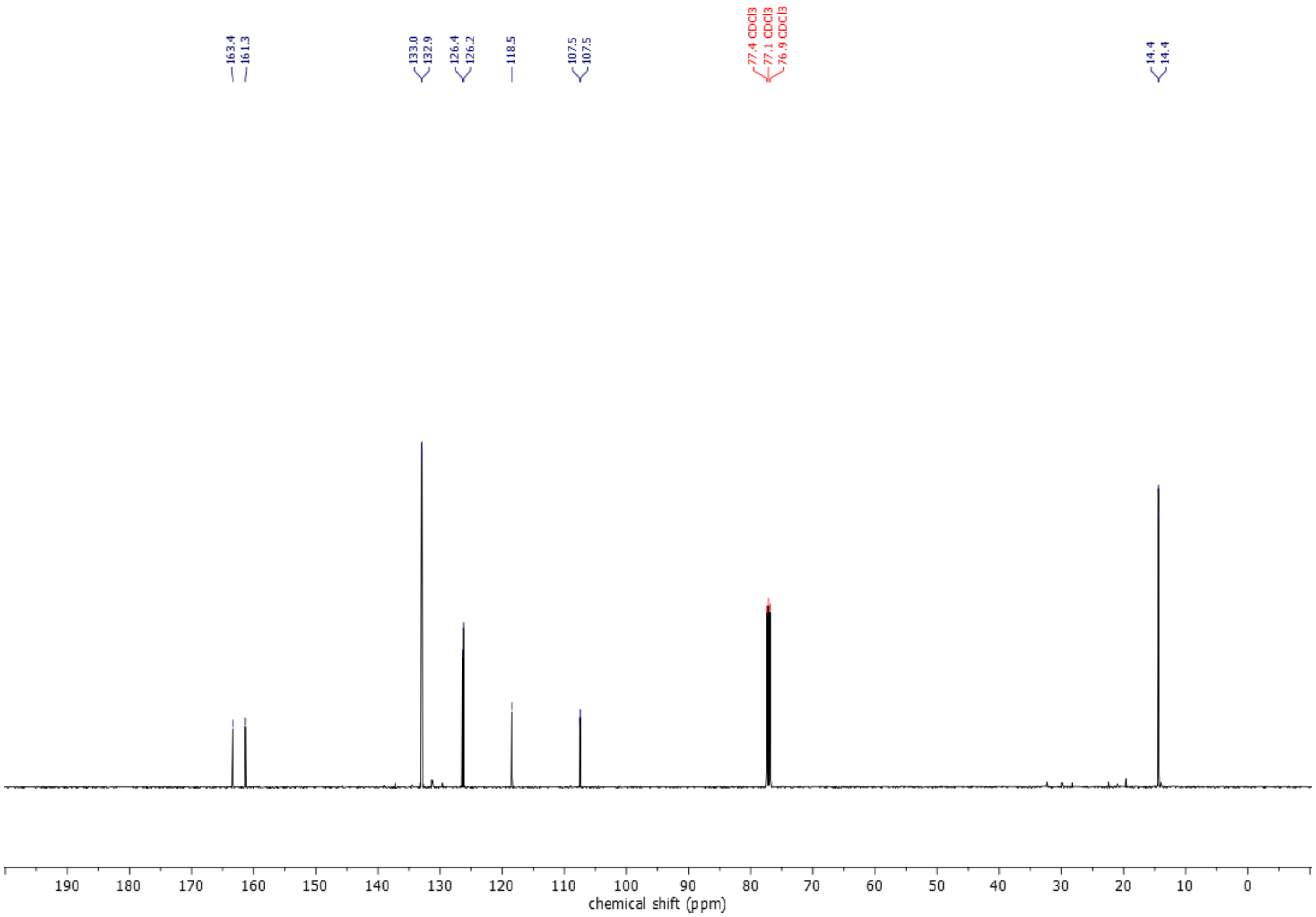

^{19}F NMR spectrum of 4-fluoro-3,5-dimethylbenzonitrile (36)

$CDCl_3$, 23°C

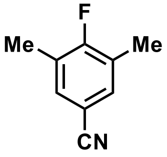

36

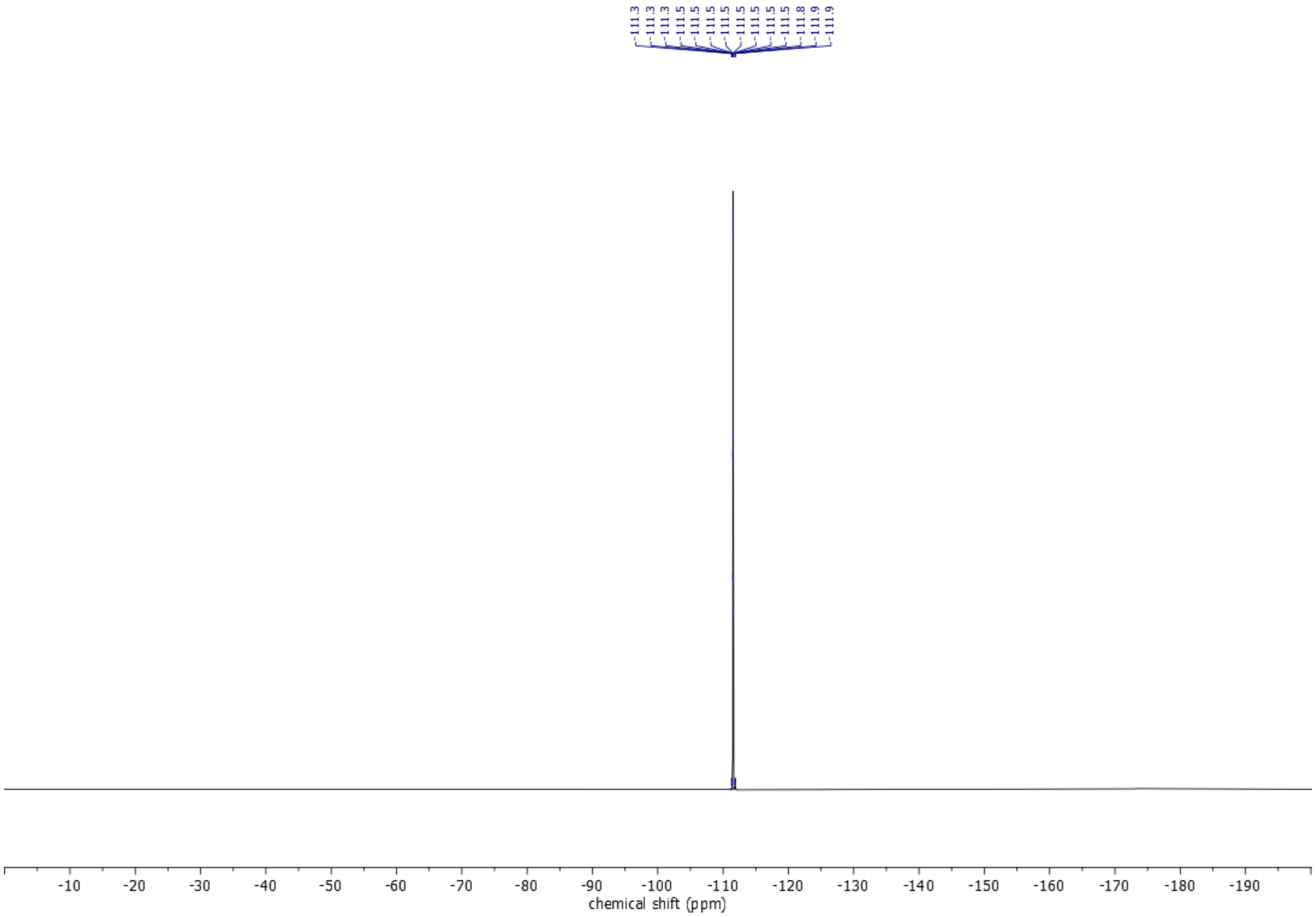

^{1}H NMR spectrum of (4-fluorophenyl)(phenyl)methanone (37)

$CDCl_3$, 23°C

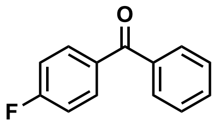

37

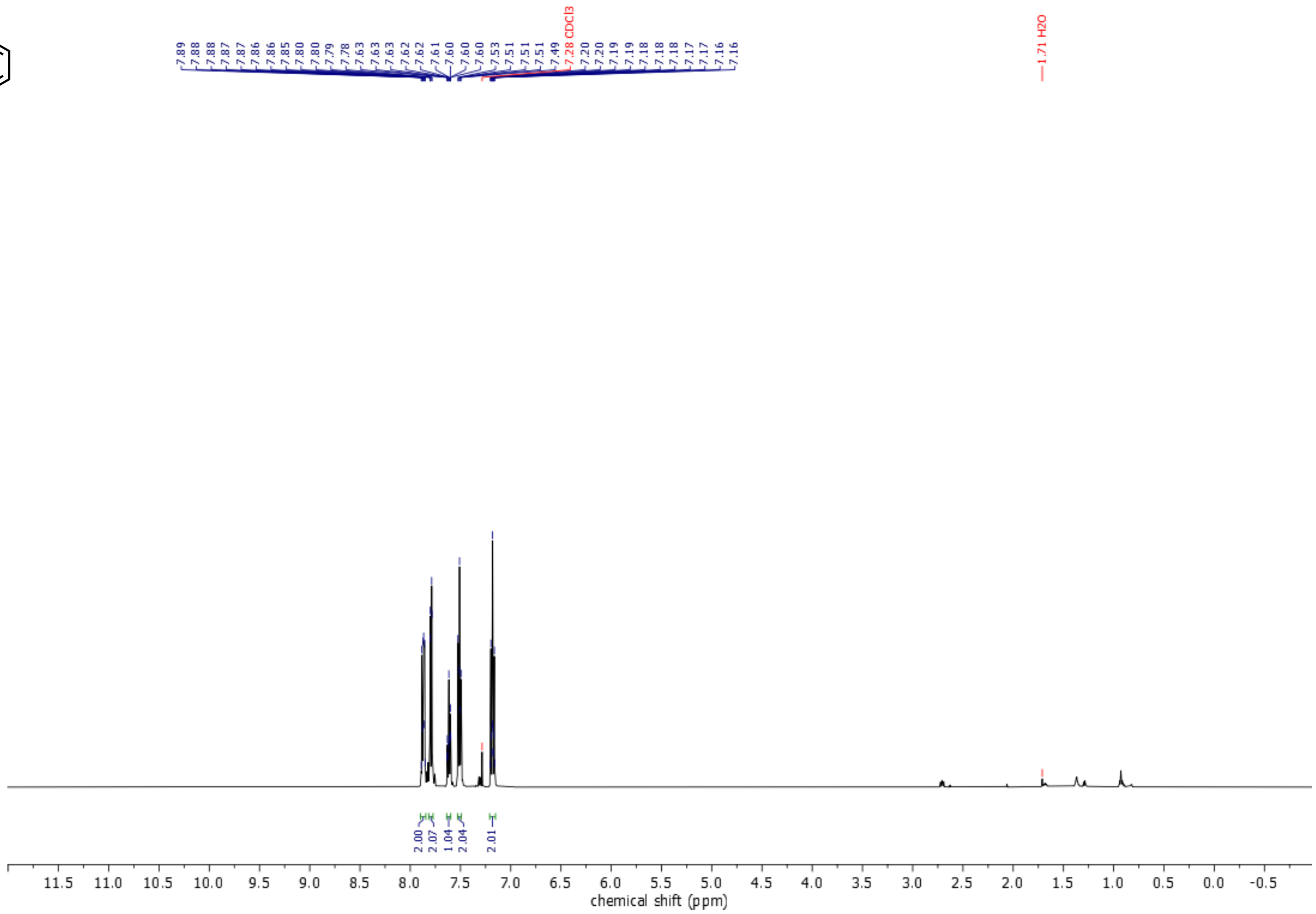

^{13}C NMR spectrum of (4-fluorophenyl)(phenyl)methanone (37)

$CDCl_3$, 23°C

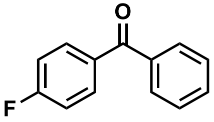

37

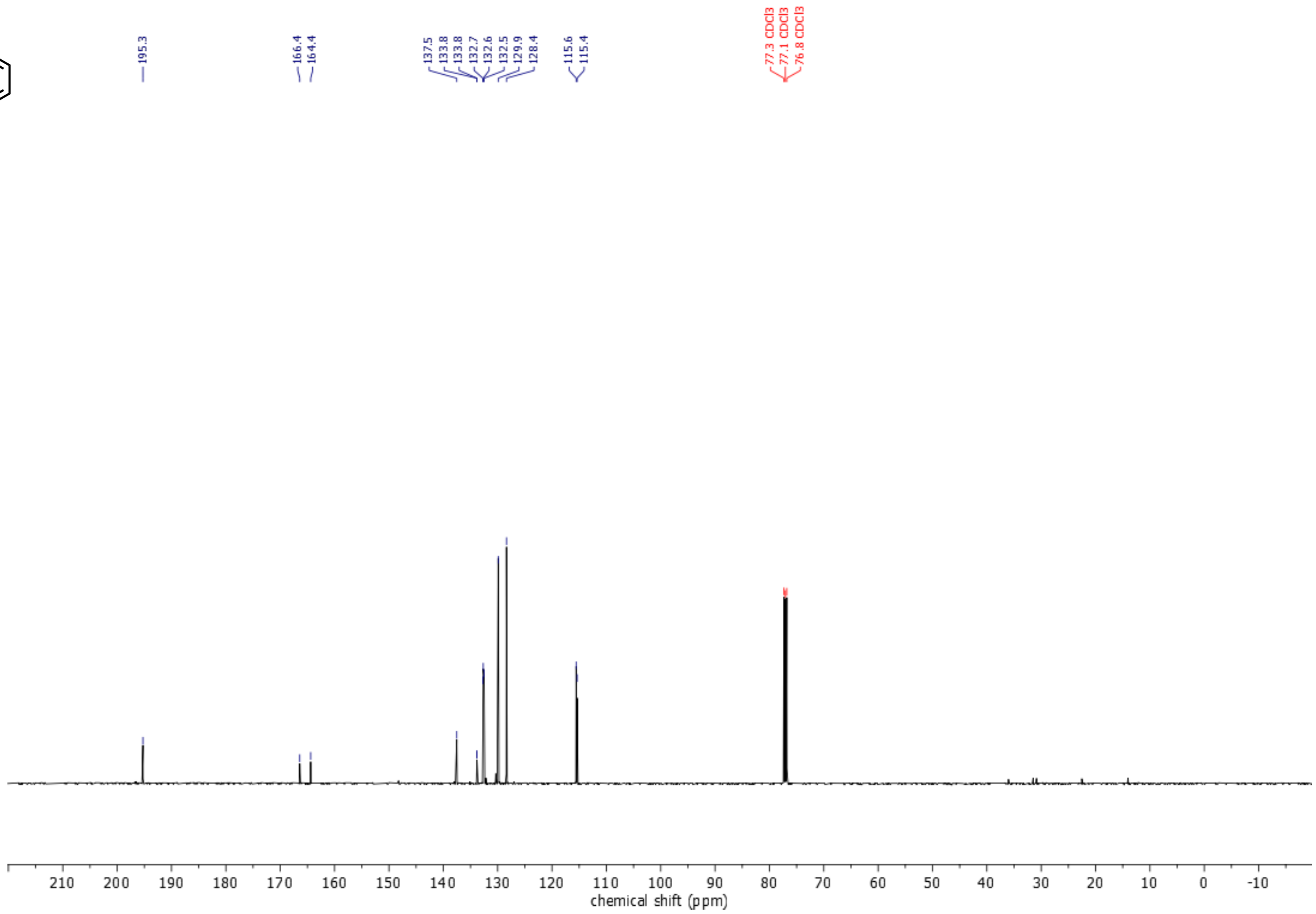

^{19}F NMR spectrum of (4-fluorophenyl)(phenyl)methanone (37)

$CDCl_3$, 23°C

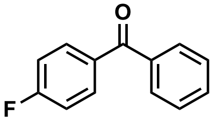

37

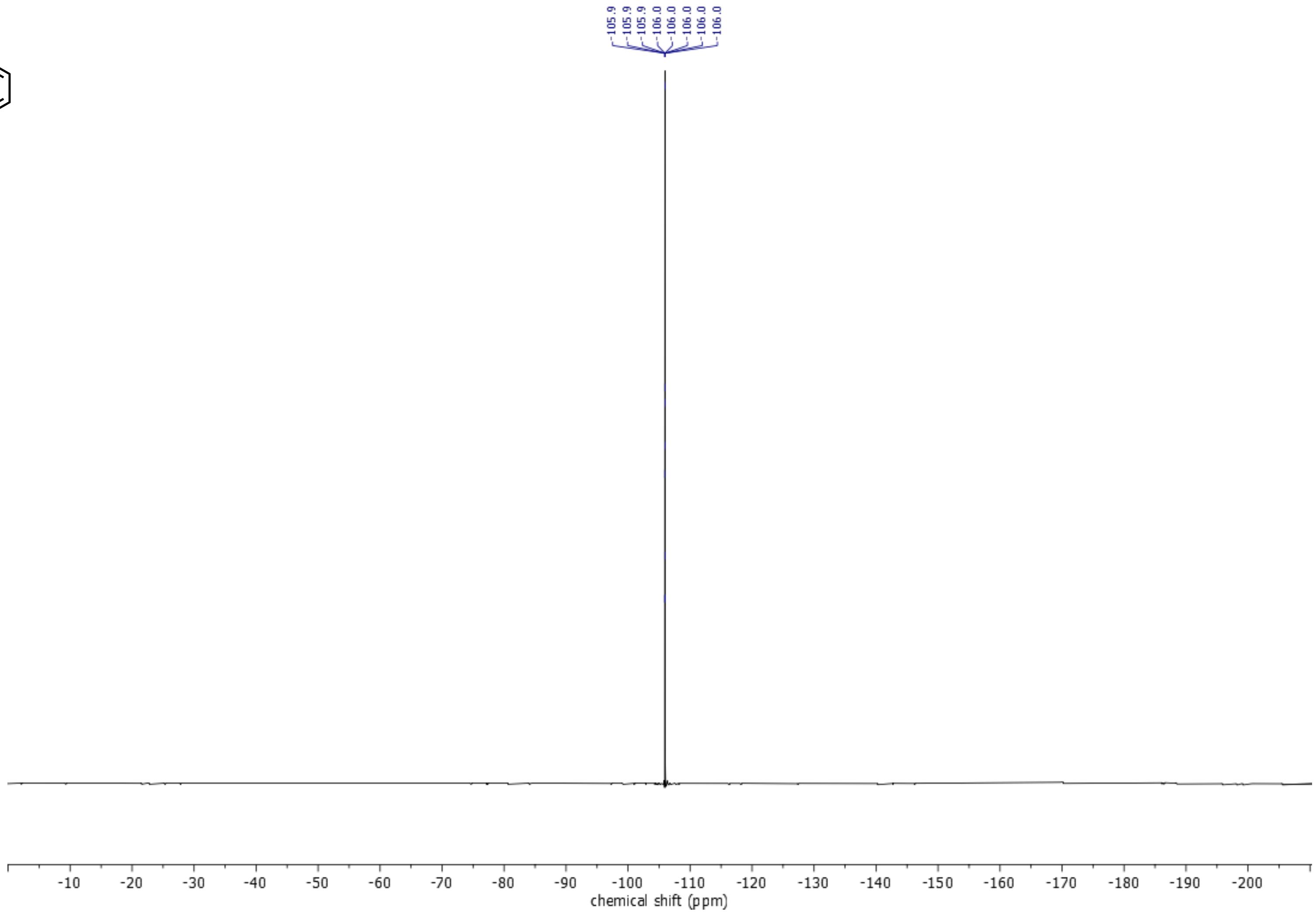

^{1}H NMR spectrum of 4'-chloro-2-fluoro-1,1'-biphenyl (40)

$CDCl_3$, 23°C

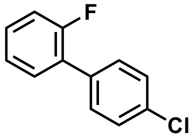

40

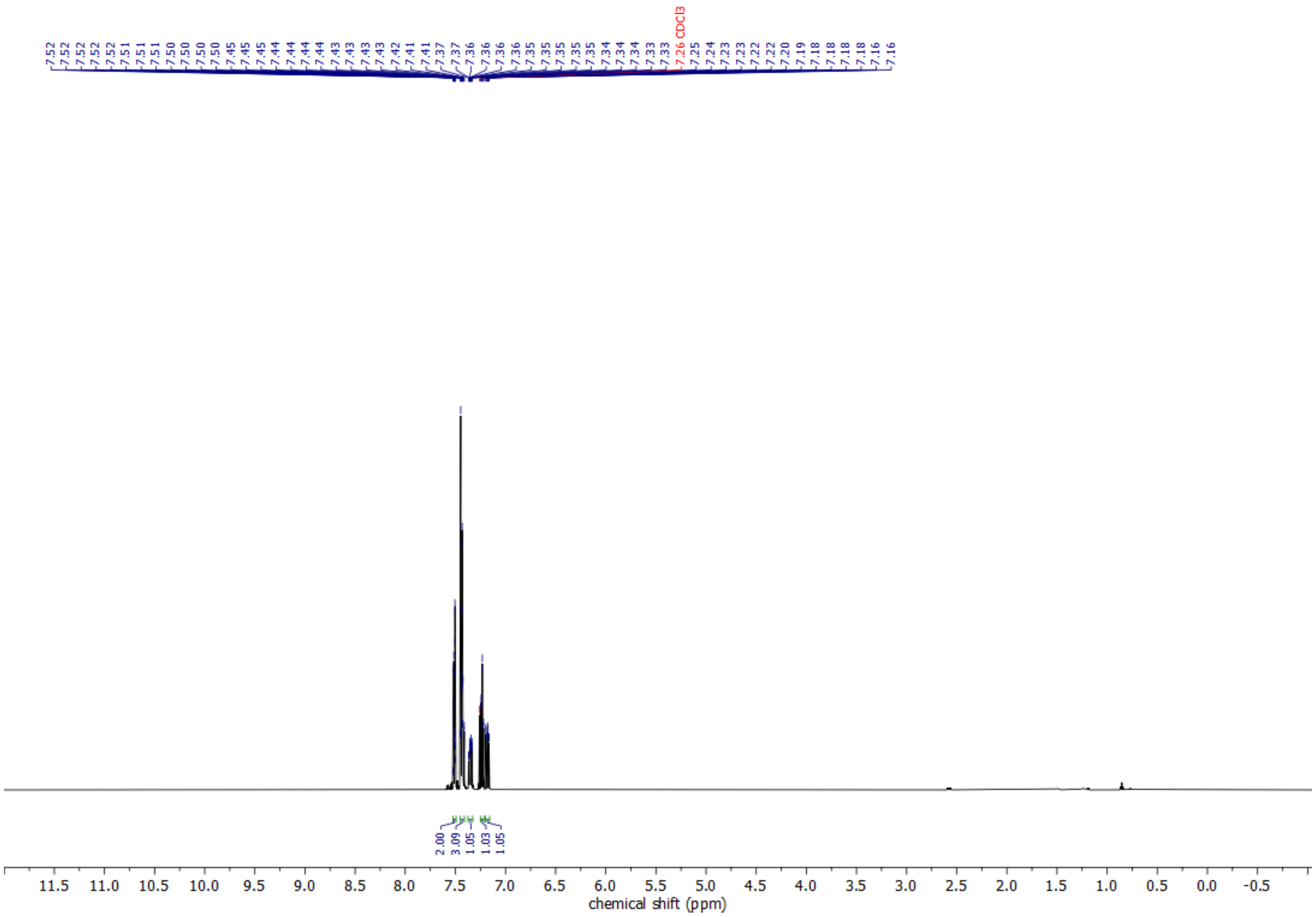

^{13}C NMR spectrum of 4'-chloro-2-fluoro-1,1'-biphenyl (40)

$CDCl_3$, 23°C

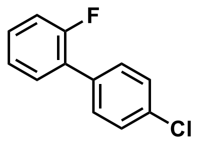

40

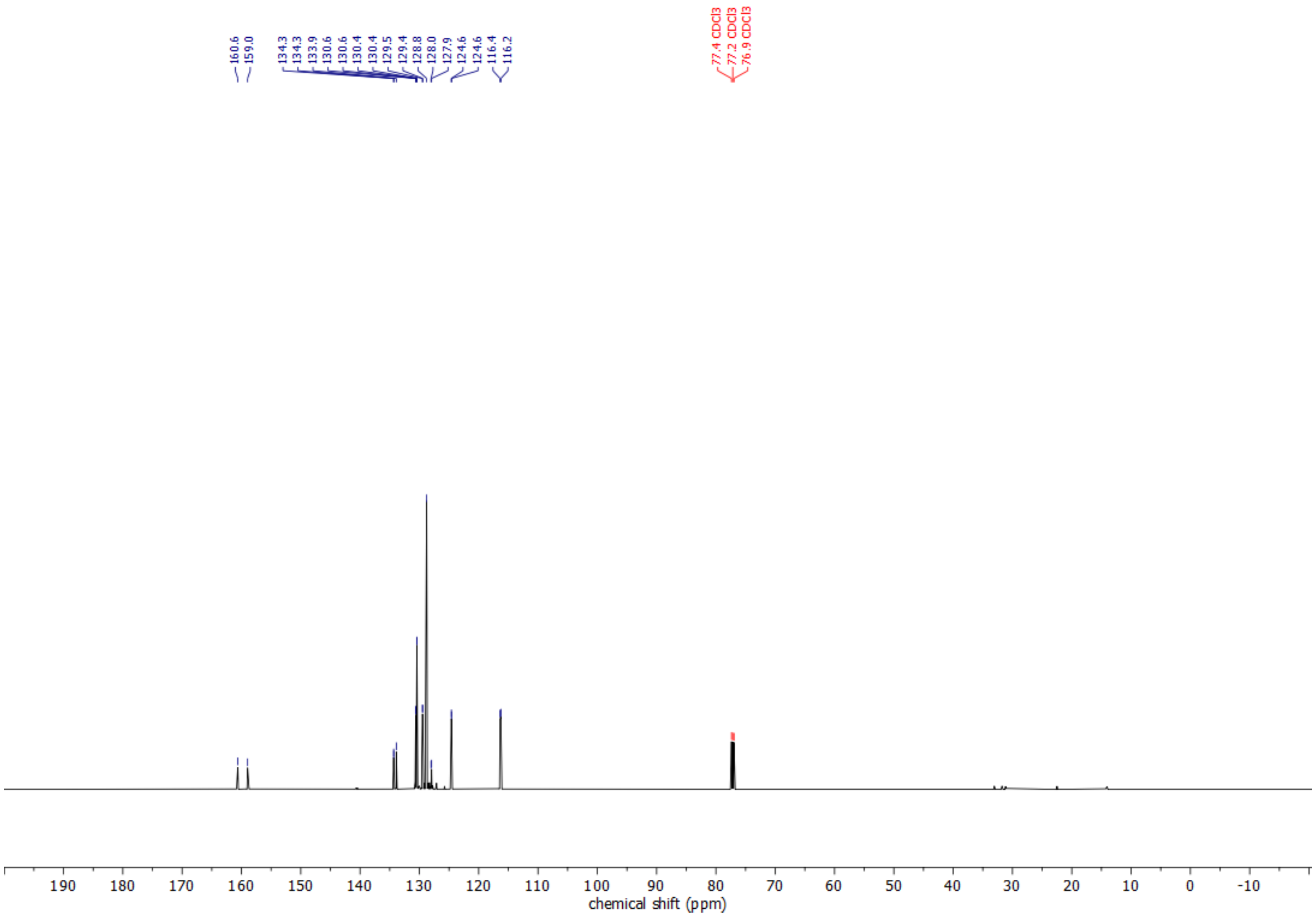

^{19}F NMR spectrum of 4'-chloro-2-fluoro-1,1'-biphenyl (40)

$CDCl_3$, 23°C

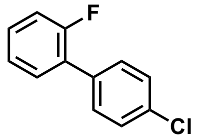

40

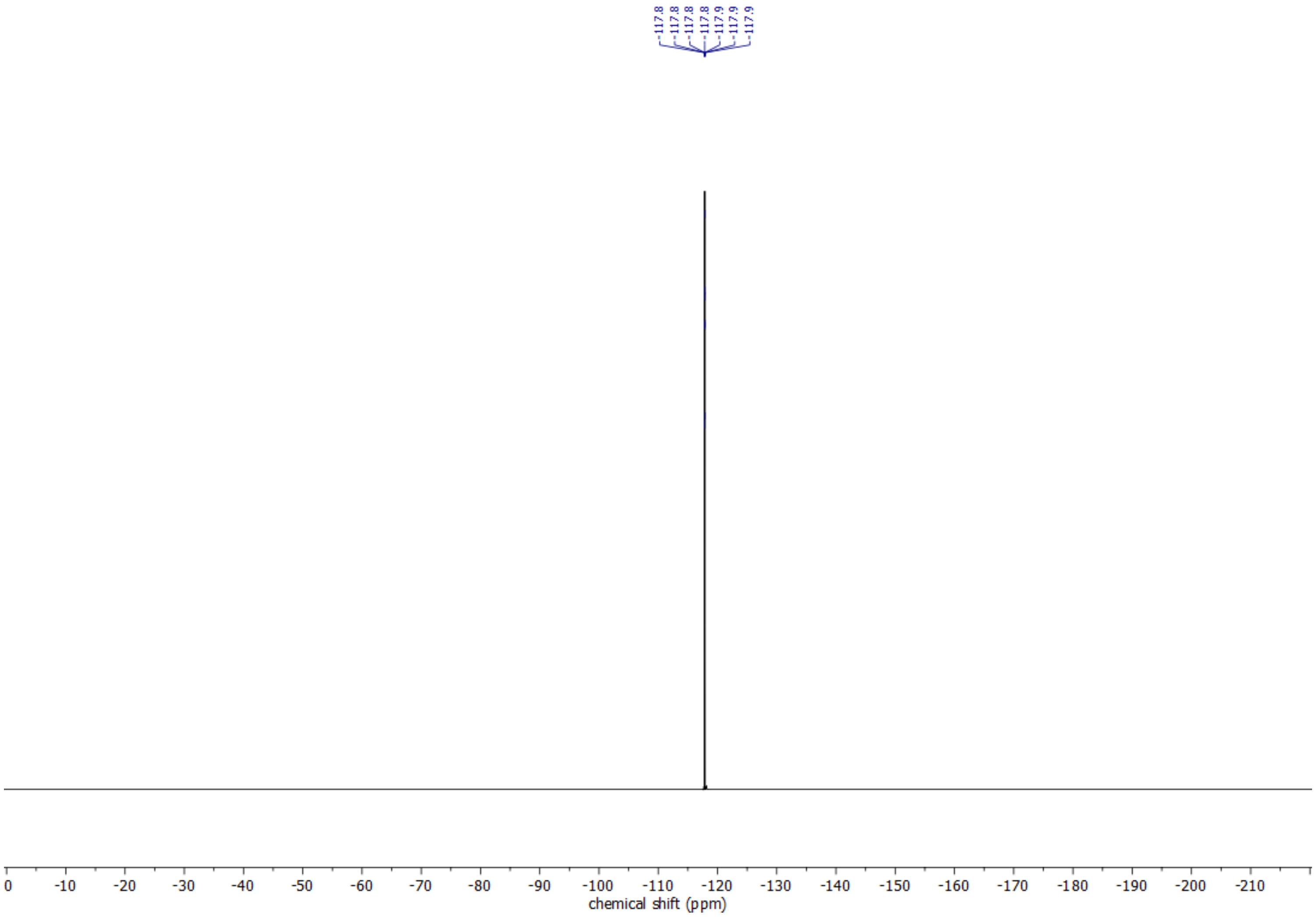

^{1}H NMR spectrum of Lenalidomide-derived fluoroarene 41

DMSO-d_6, 23°C

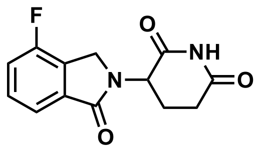

41

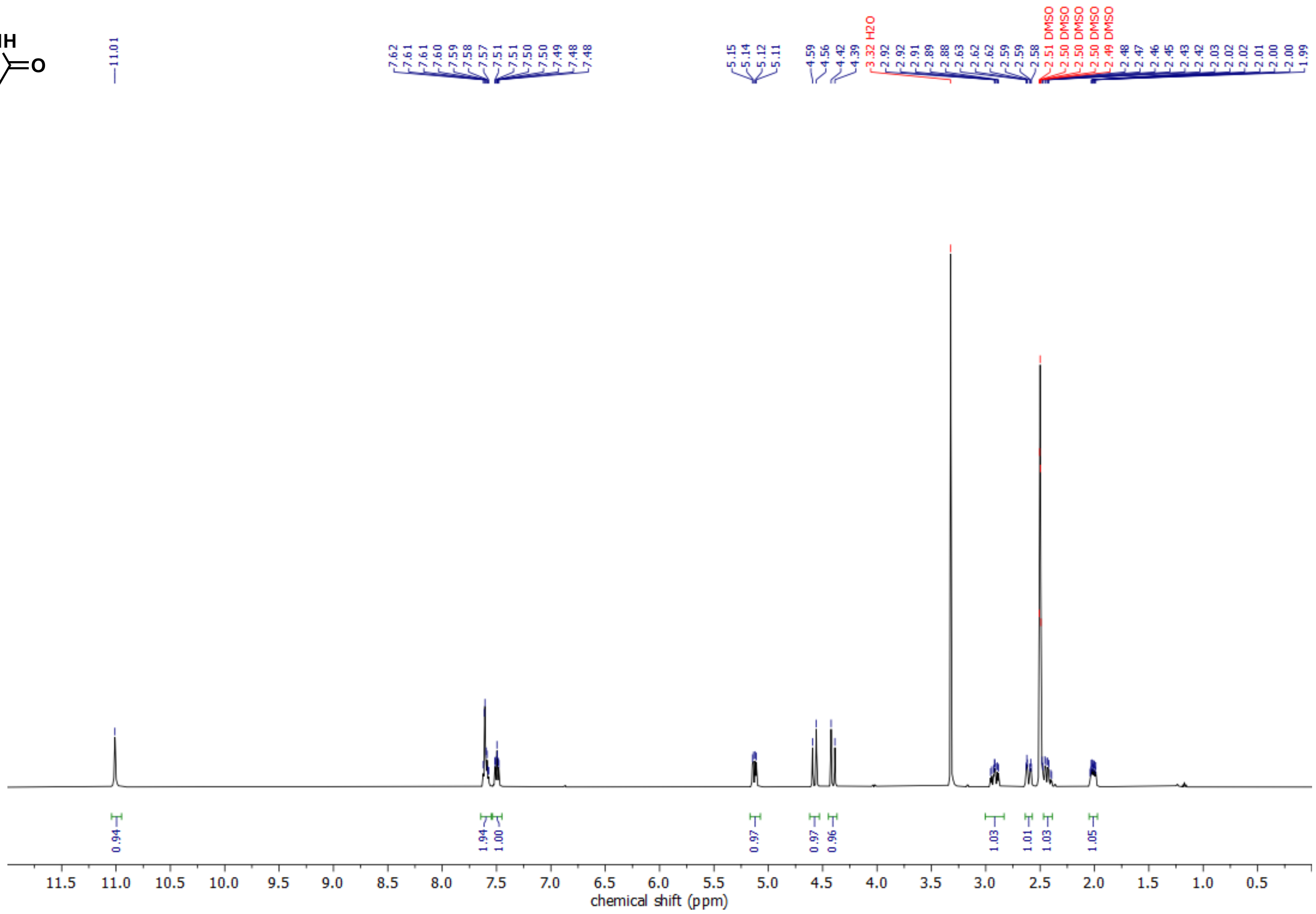

^{13}C NMR spectrum of Lenalidomide-derived fluoroarene 41

DMSO-d_6, 23°C

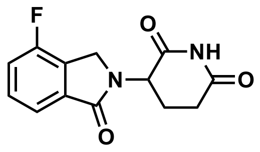

41

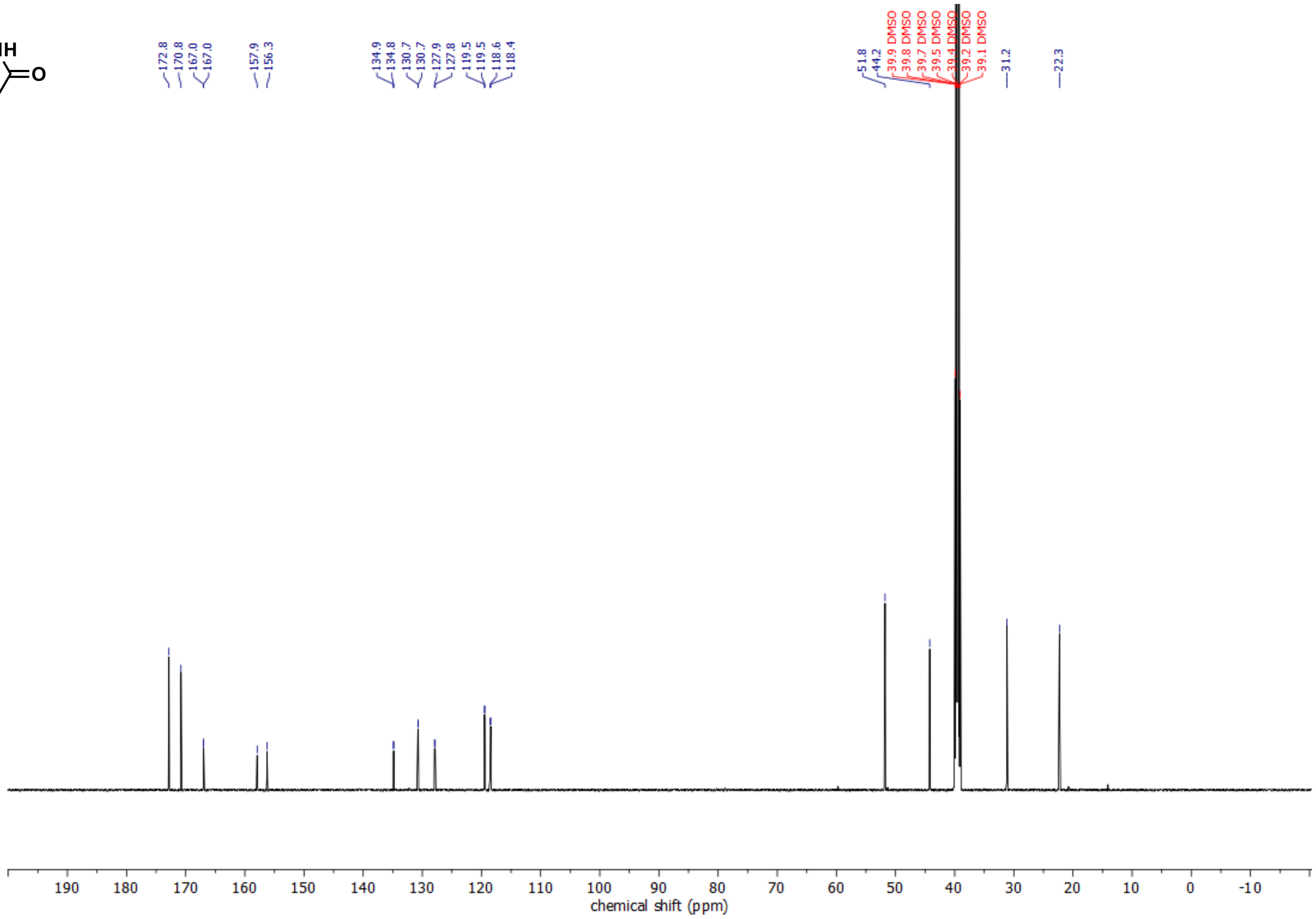

^{19}F NMR spectrum of Lenalidomide-derived fluoroarene 41

DMSO-d_6, 23°C

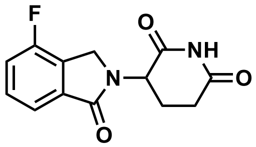

41

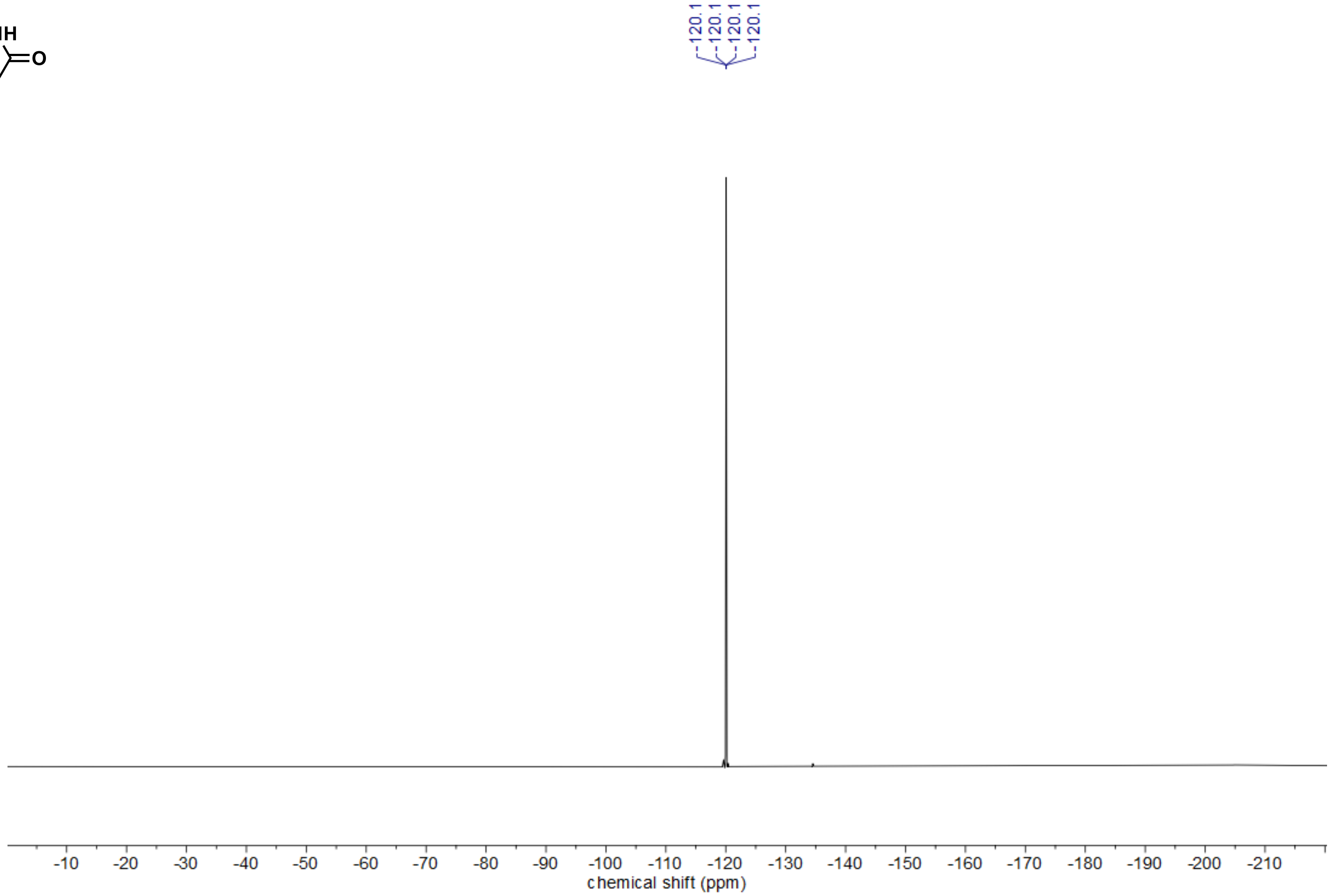

^{1}H NMR spectrum of 4-benzoylbenzenesulfonic acid (42)

DMSO-d_6, 23°C

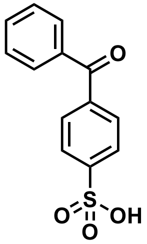

42

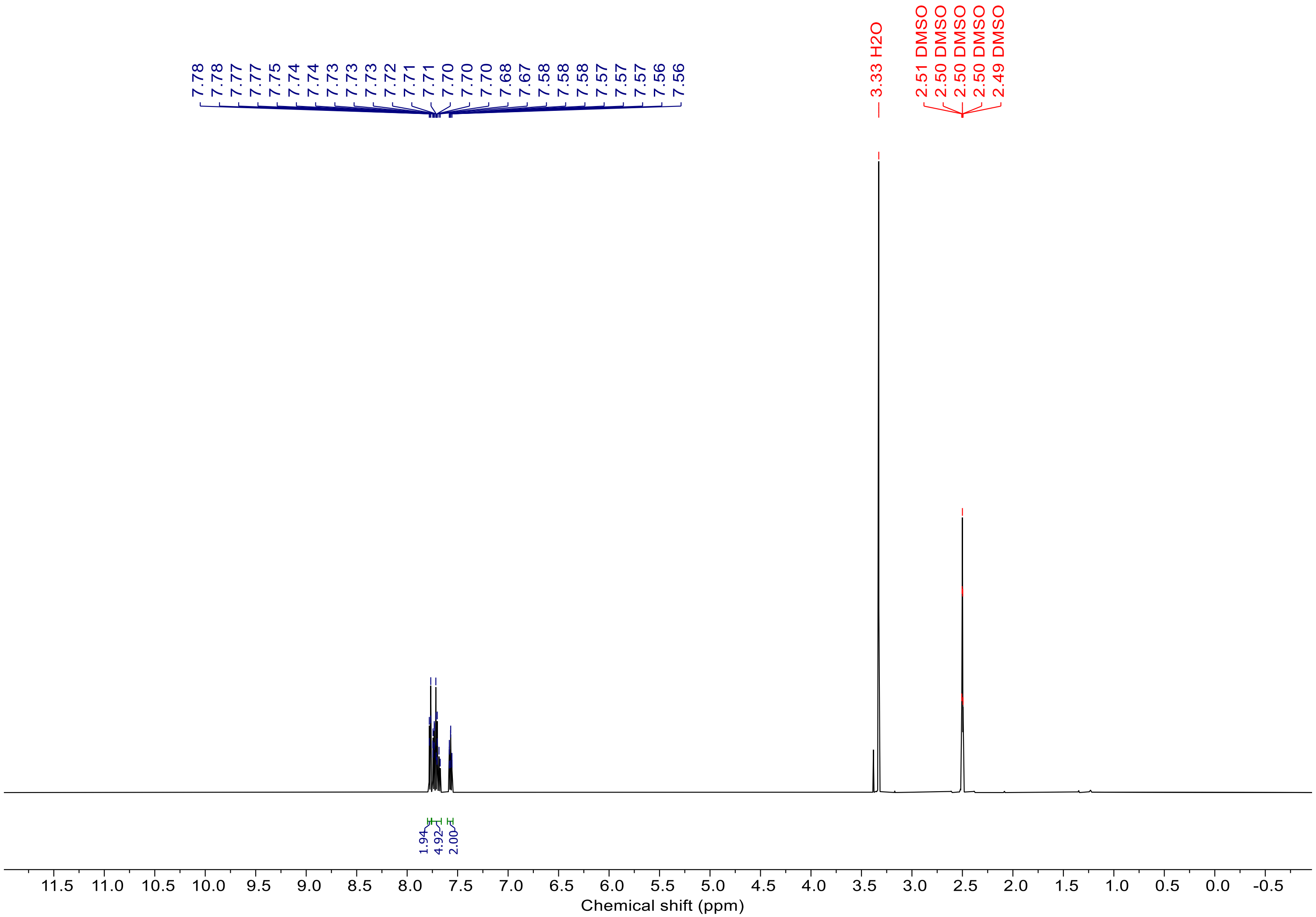

^{13}C NMR spectrum of 4-benzoylbenzenesulfonic acid (42)

DMSO-d_6, 23°C

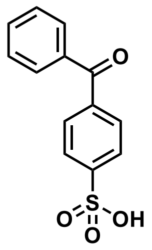

42

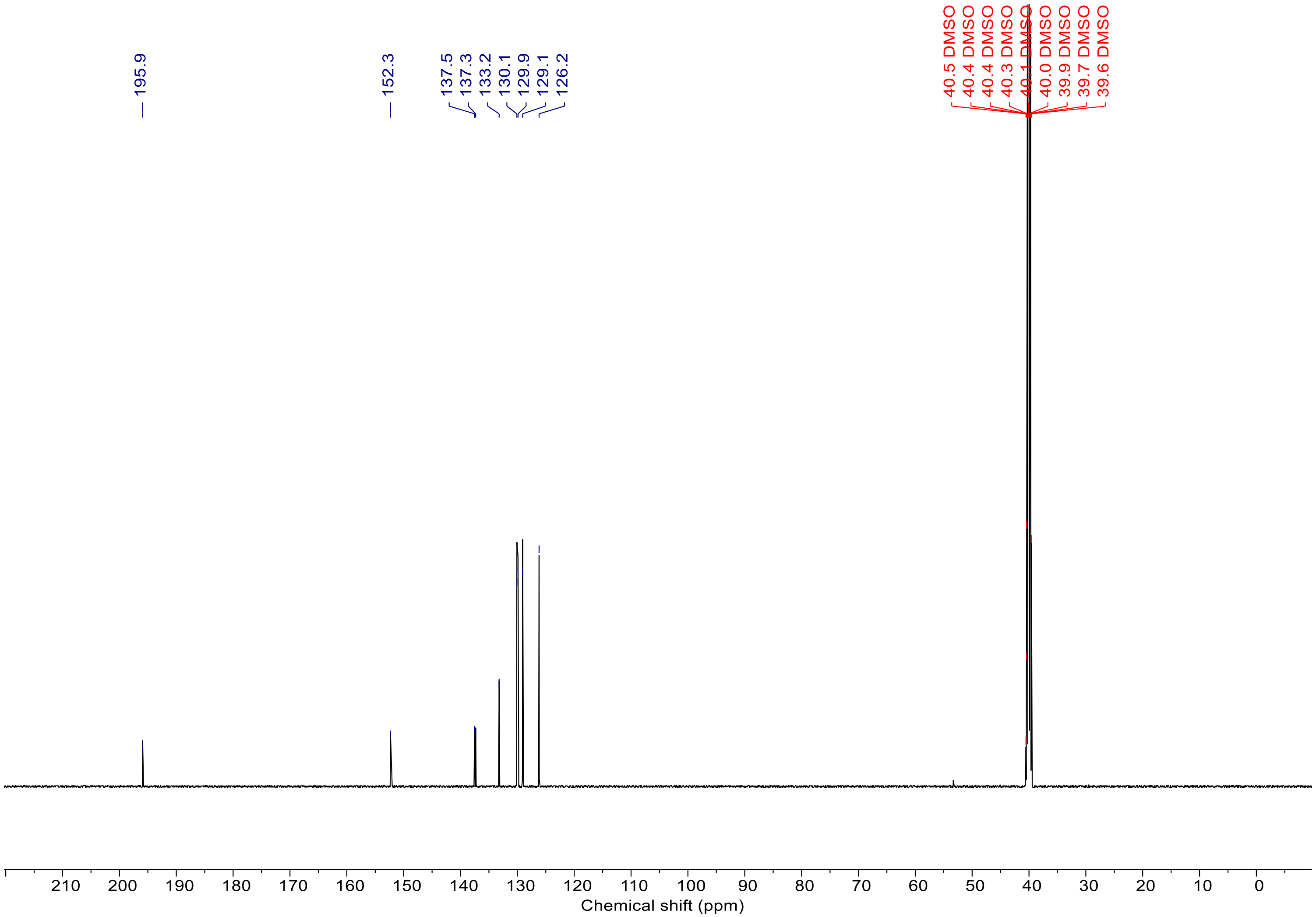

^{1}H NMR spectrum of 4-phenoxybenzenesulfonic acid (43)

DMSO-d_6, 23°C

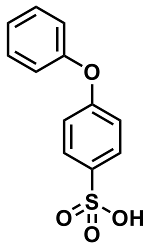

43

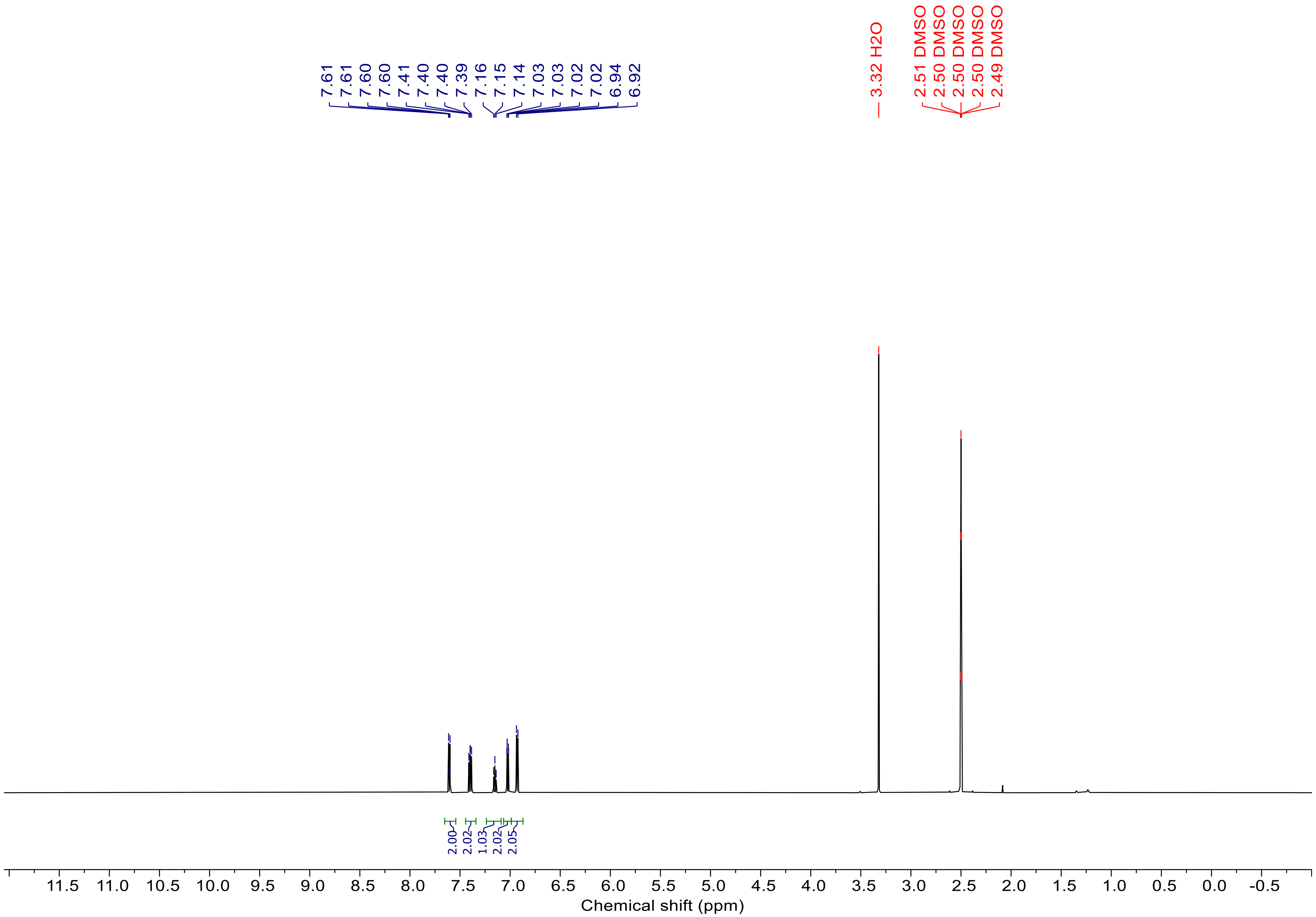

^{13}C NMR spectrum of 4-phenoxybenzenesulfonic acid (43)

DMSO-d_6, 23°C

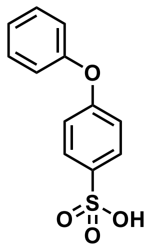

43

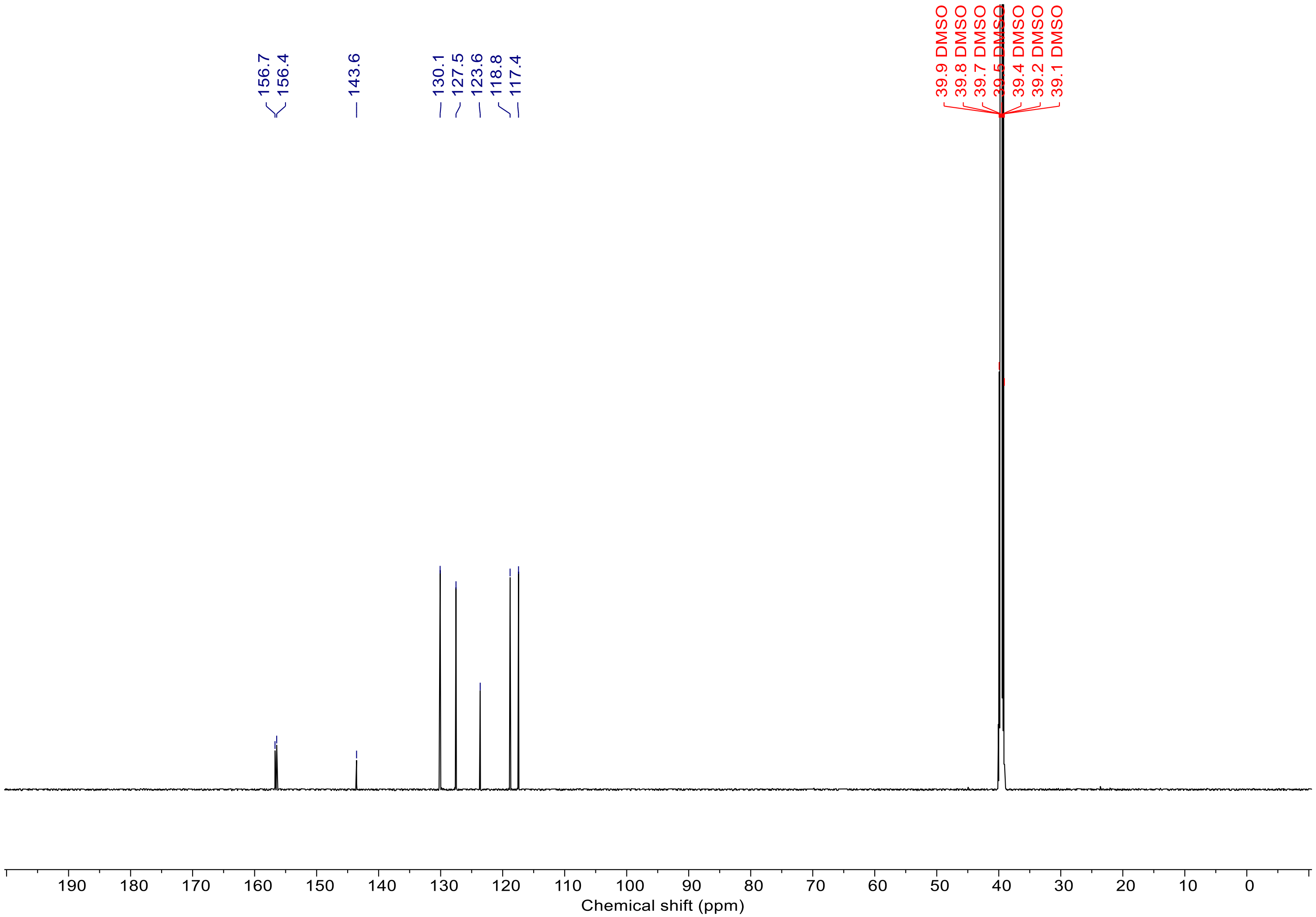

^{1}H NMR spectrum of 4-cyanobenzenesulfonic acid (44)

DMSO-d_6, 23°C

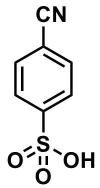

44

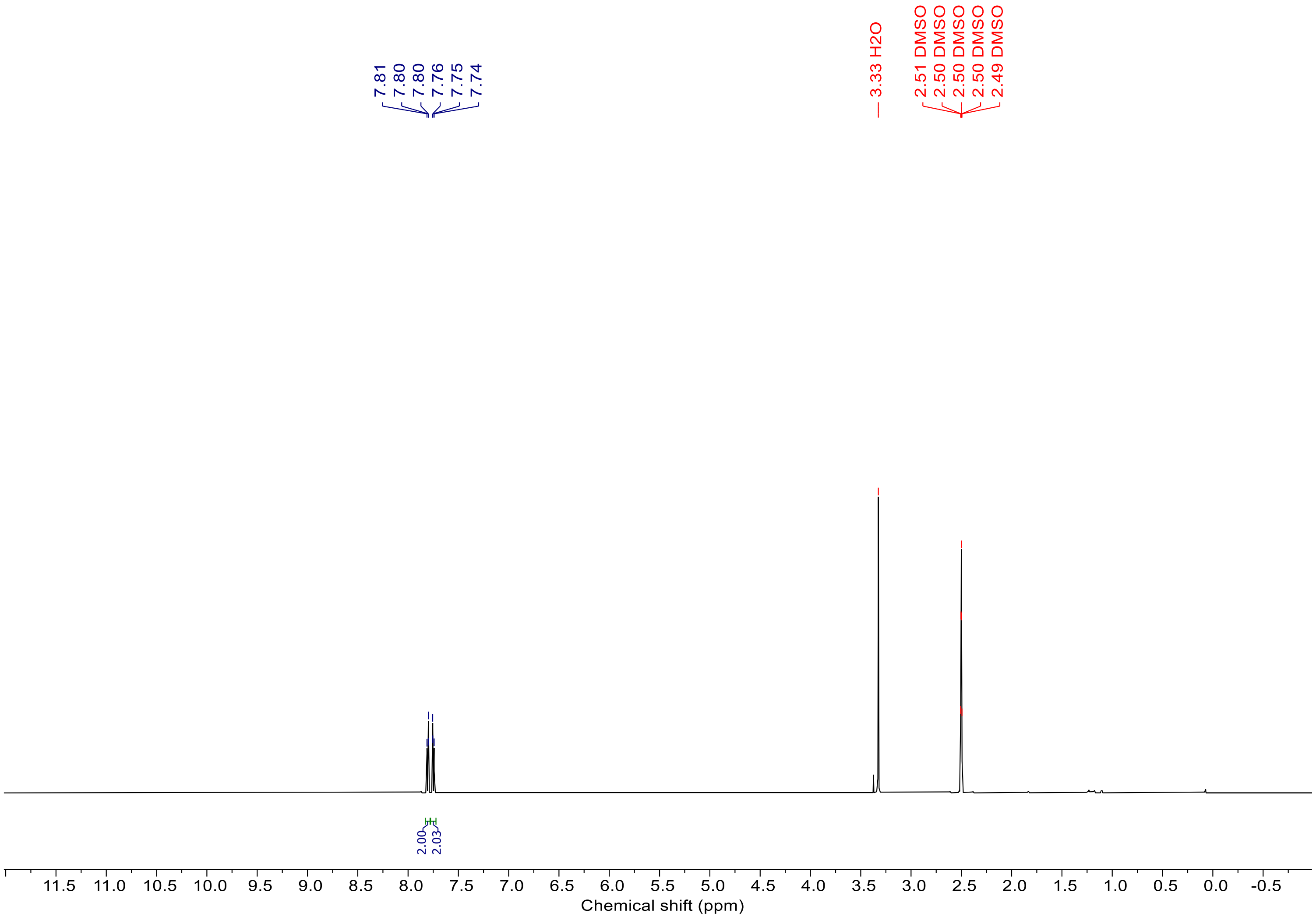

^{13}C NMR spectrum of 4-cyanobenzenesulfonic acid (44)

DMSO-d_6, 23°C

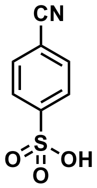

44

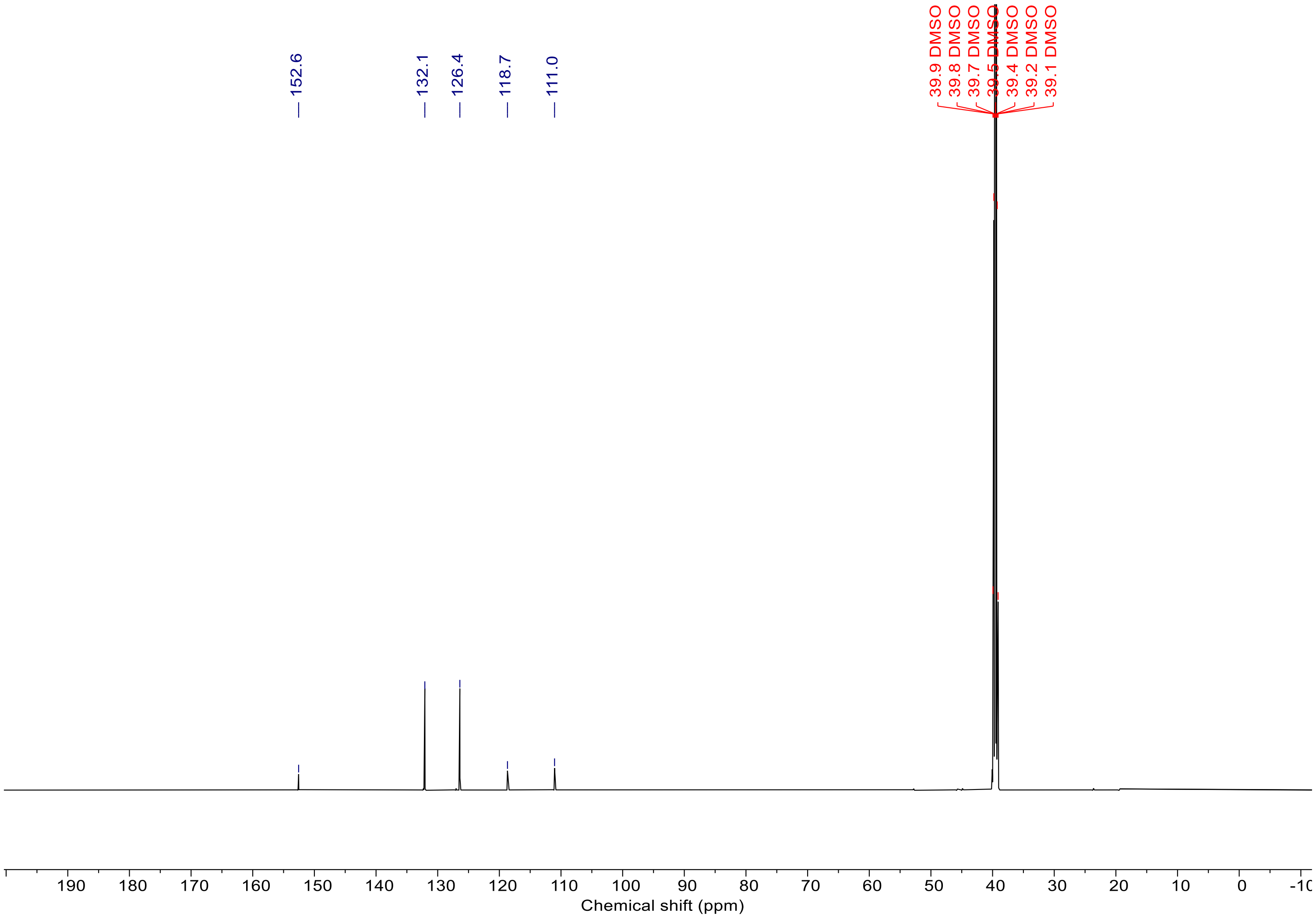

^{1}H NMR spectrum of 3-chloro-4-methylbenzenesulfonic acid (45)

CD_3OD, 23°C

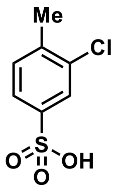

45

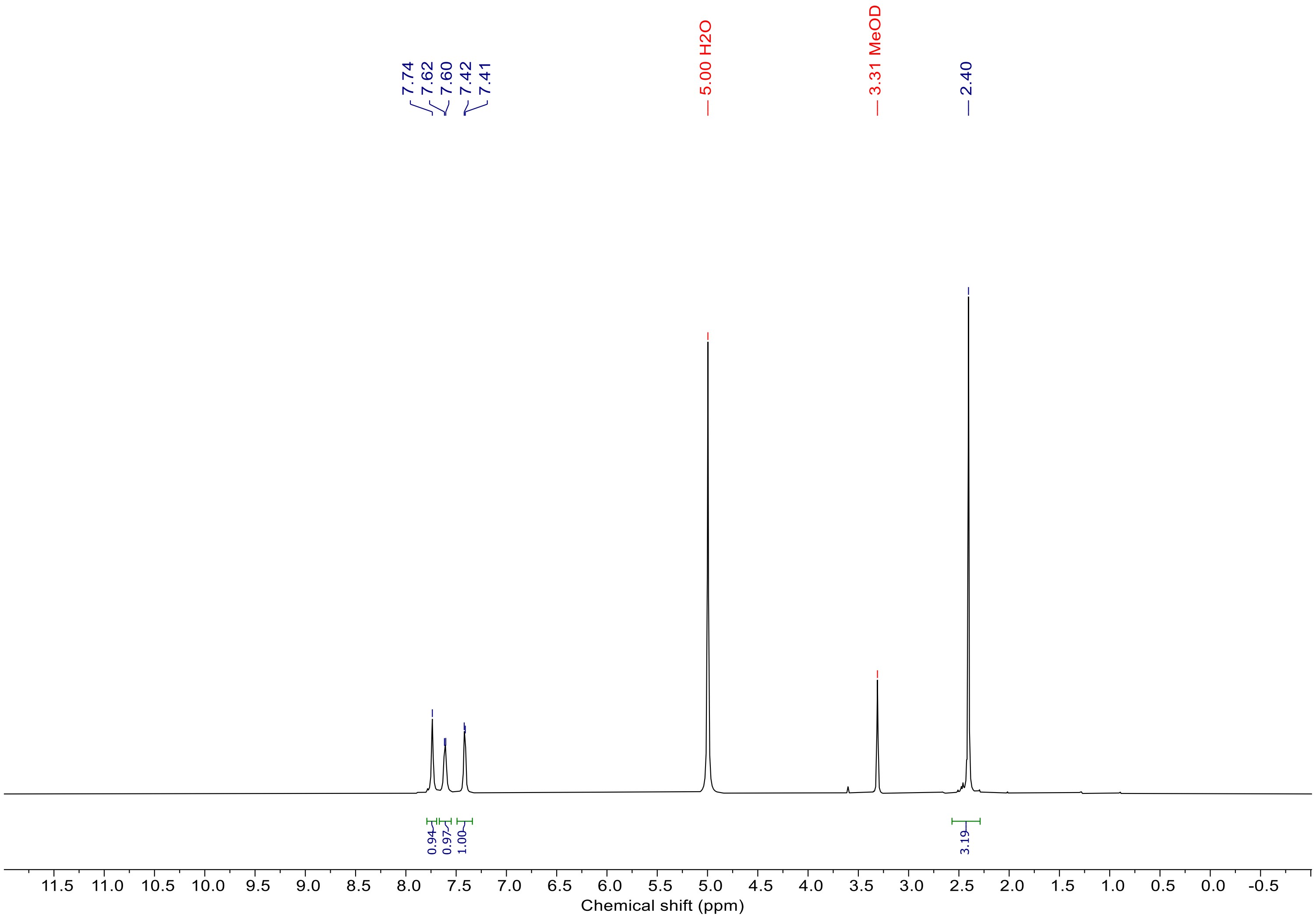

^{13}C NMR spectrum of 3-chloro-4-methylbenzenesulfonic acid (45)

CD_3OD, 23°C

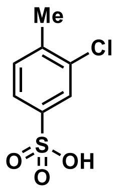

45

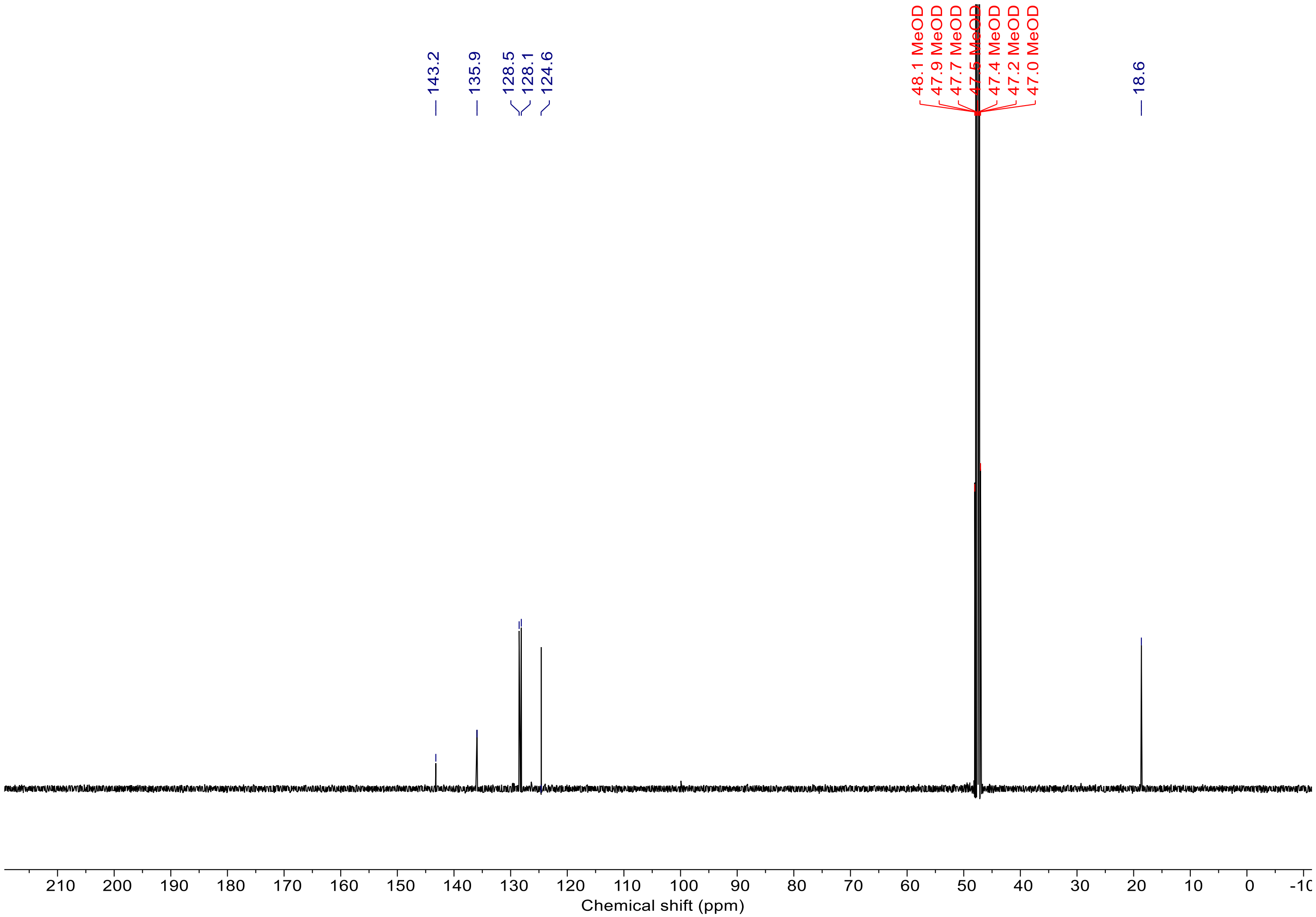

^{1}H NMR spectrum of 4-fluorobenzenesulfonic acid (46)

CD_3OD, 23°C

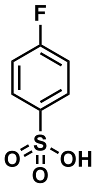

46

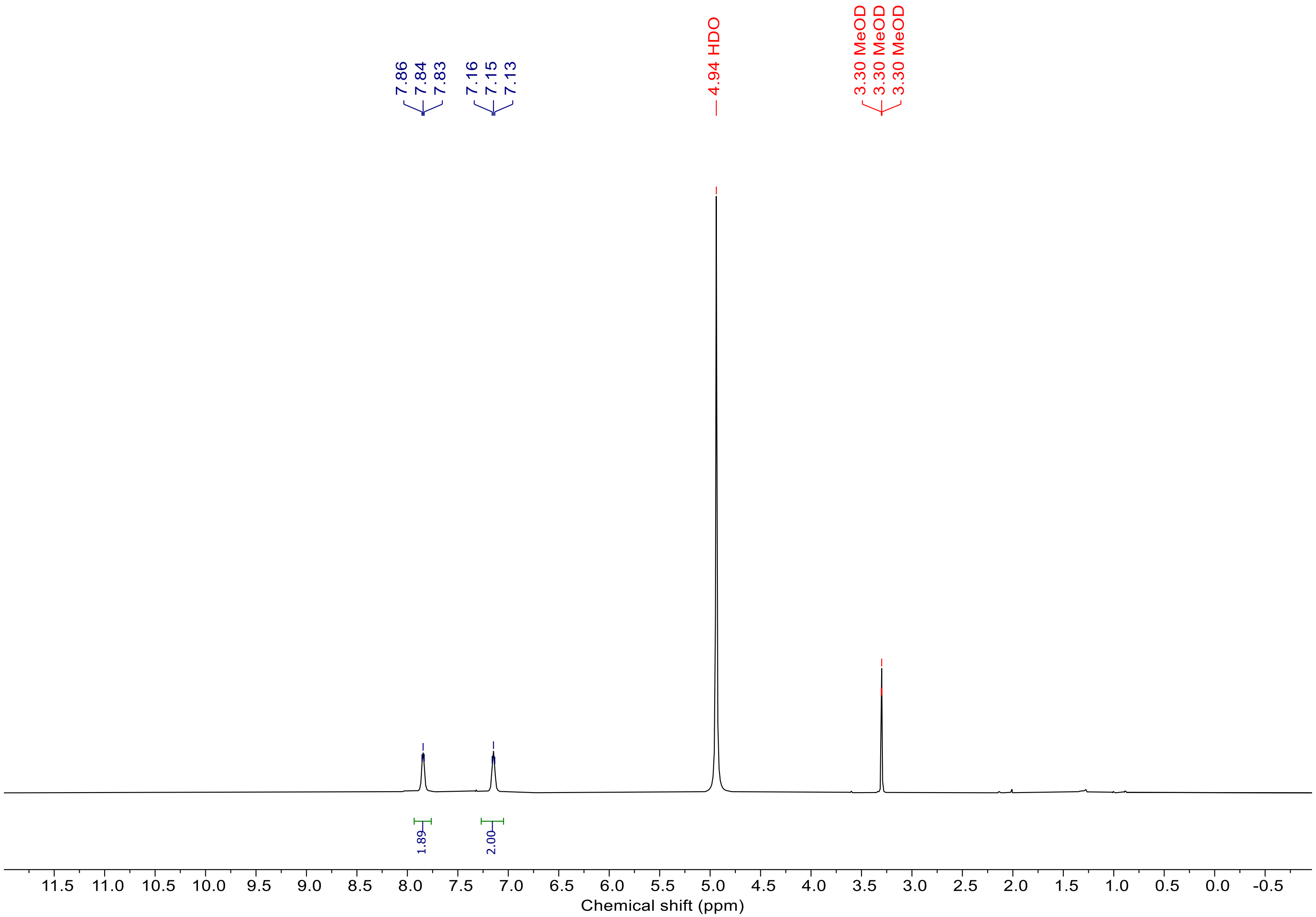

^{13}C NMR spectrum of 4-fluorobenzenesulfonic acid (46)

CD_3OD, 23°C

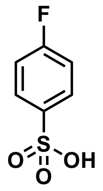

46

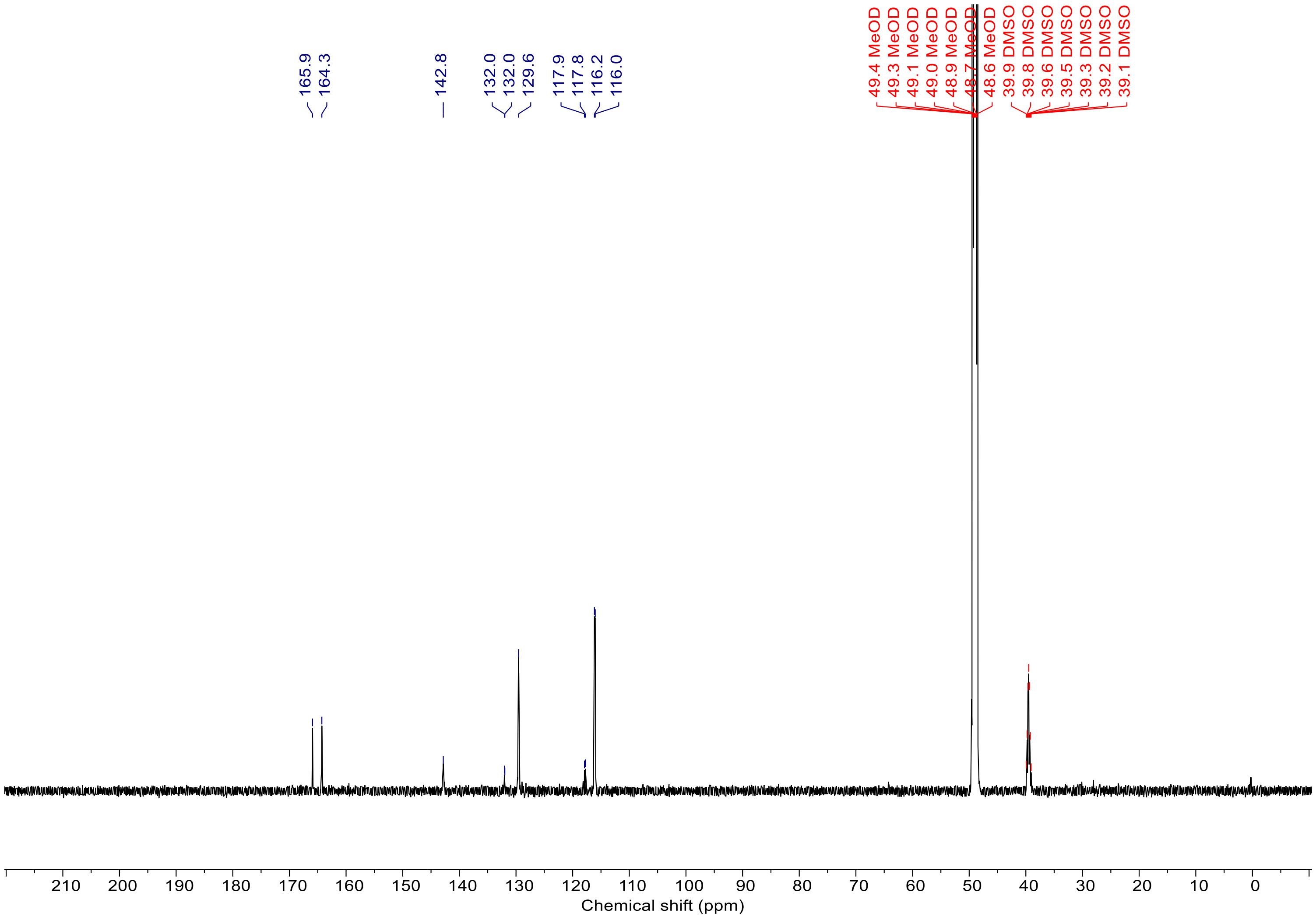

^{19}F NMR spectrum of 4-fluorobenzenesulfonic acid (46)

CD_3OD, 23°C

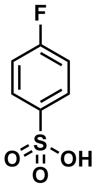

46

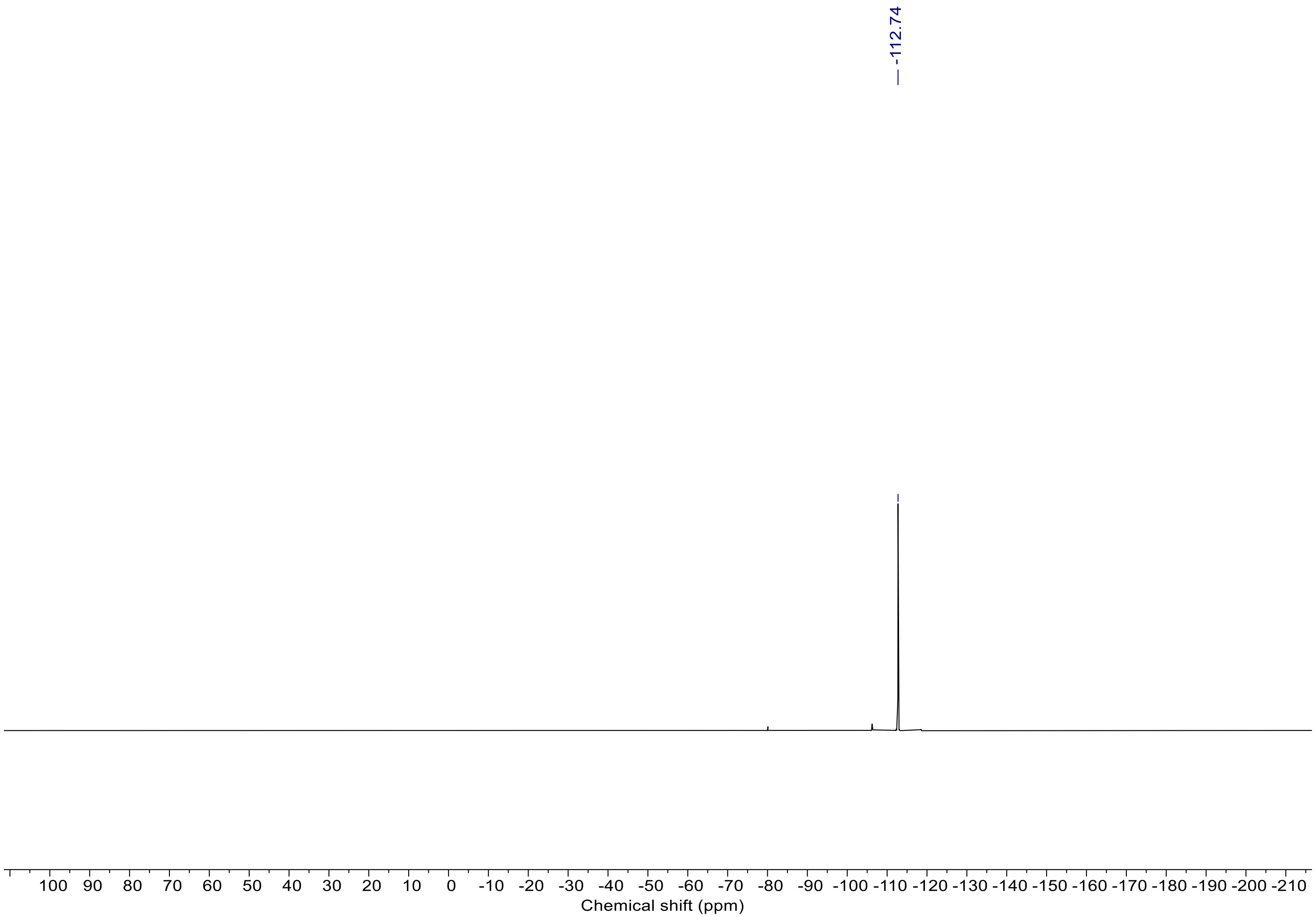

^{1}H NMR spectrum of Aminoglutethimide-derived sulfonic acid *rac*-47

CD3OD, 23°C

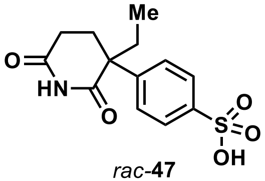

rac-**47**

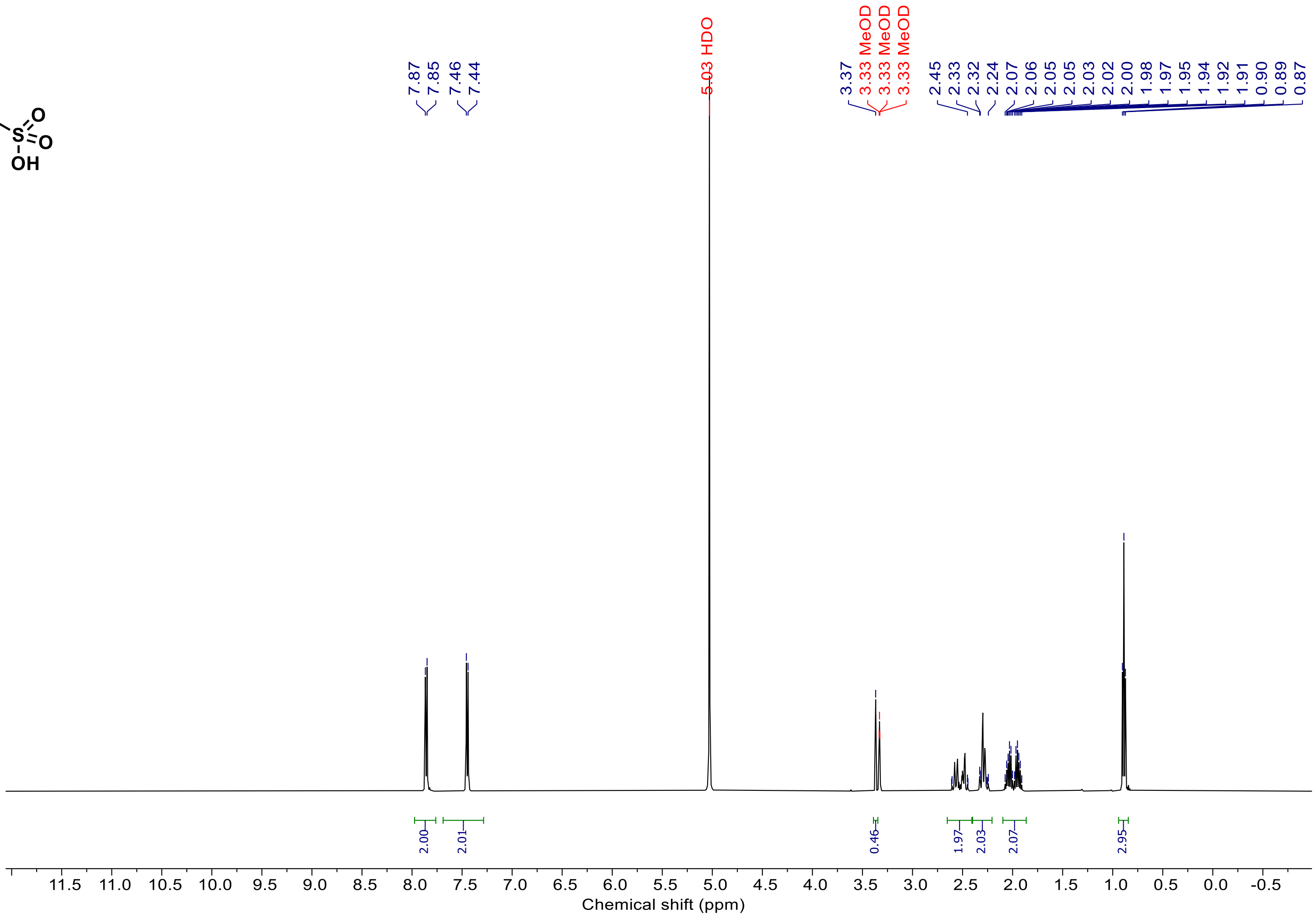

^{13}C NMR spectrum of Aminoglutethimide-derived sulfonic acid *rac*-47

CD_3OD, 23°C

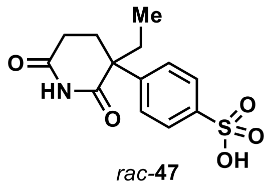

rac-**47**

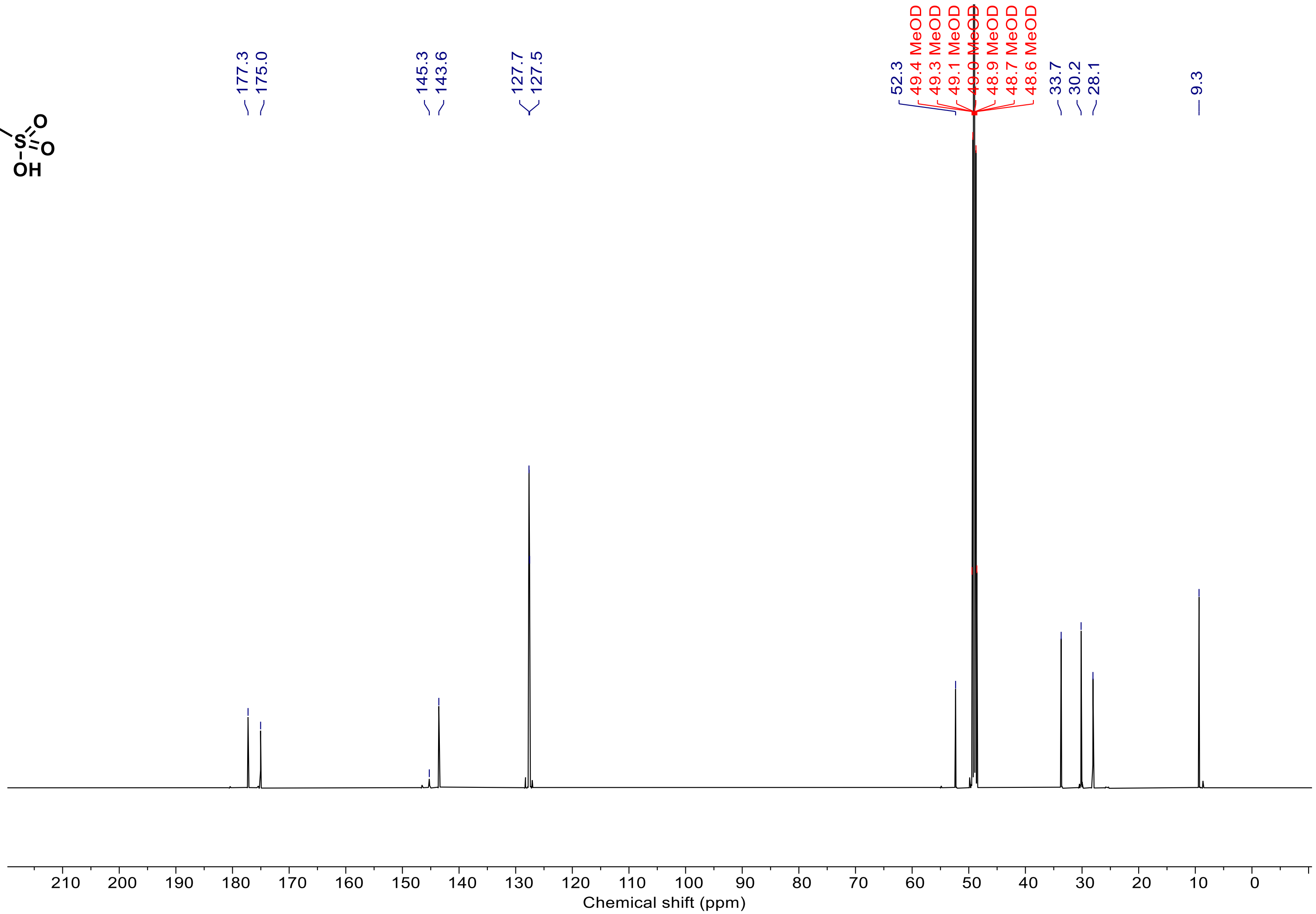

^{1}H NMR spectrum of 2-methyl-3-nitrobenzenesulfonic acid (48)

CD_3OD, 23°C

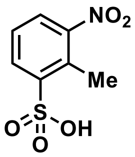

48

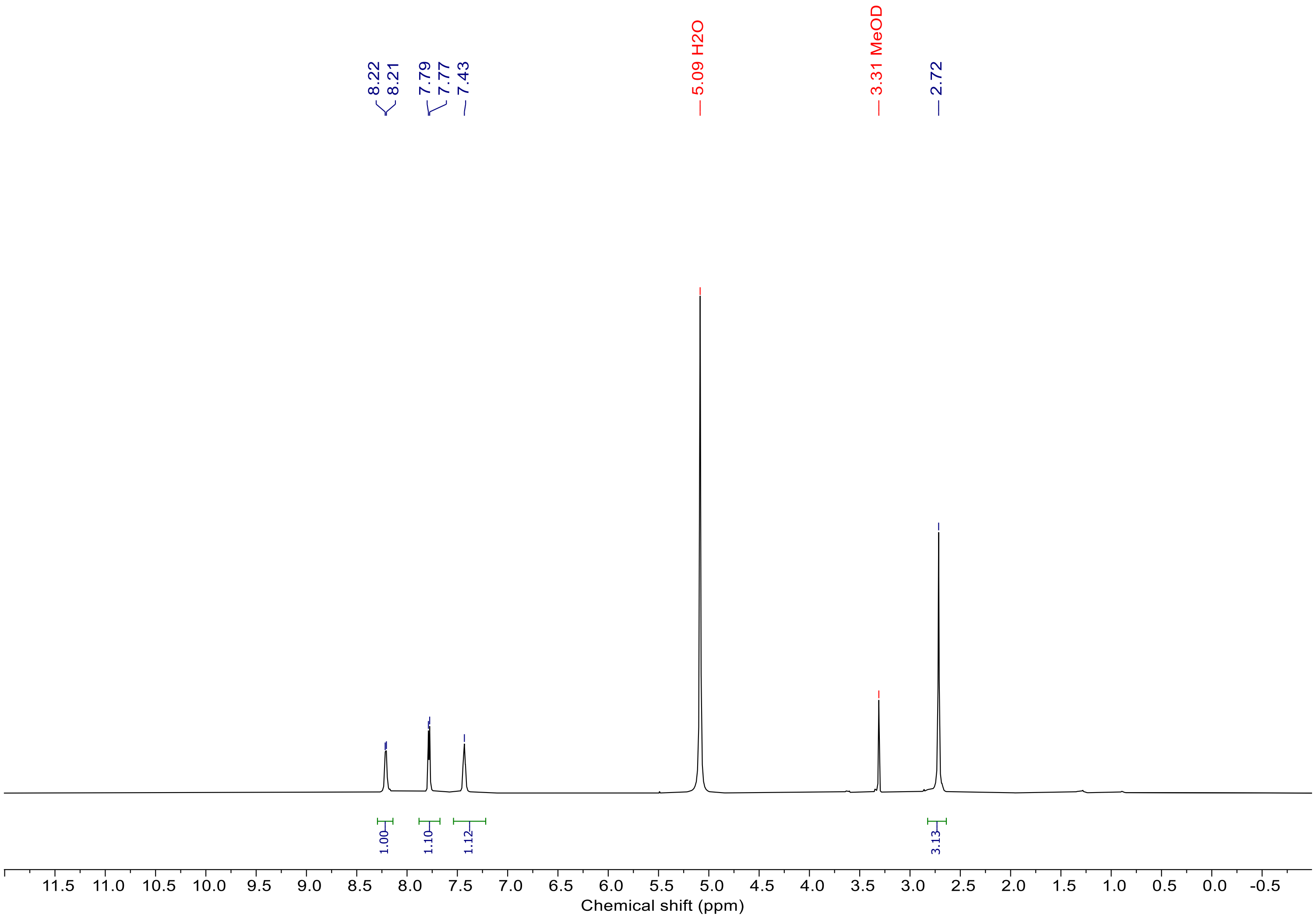

^{13}C NMR spectrum of 2-methyl-3-nitrobenzenesulfonic acid (48)

CD_3OD, 23°C

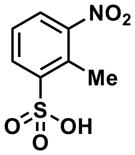

48

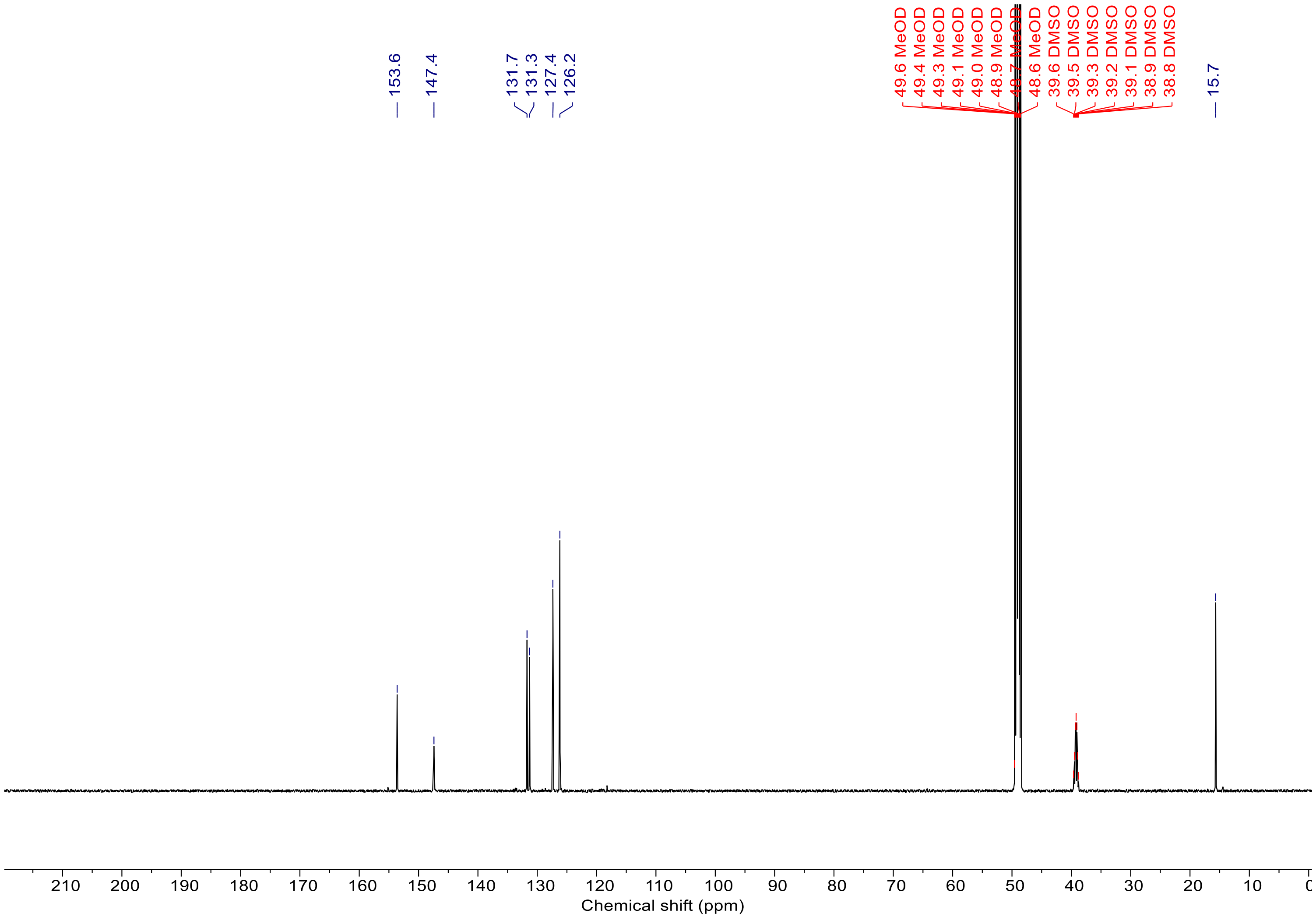

^{1}H NMR spectrum of Procainamide-derived sulfonic acid 49

D_2O, 23°C

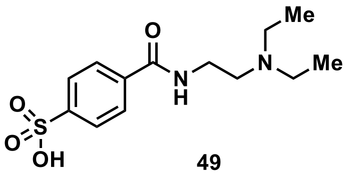

49

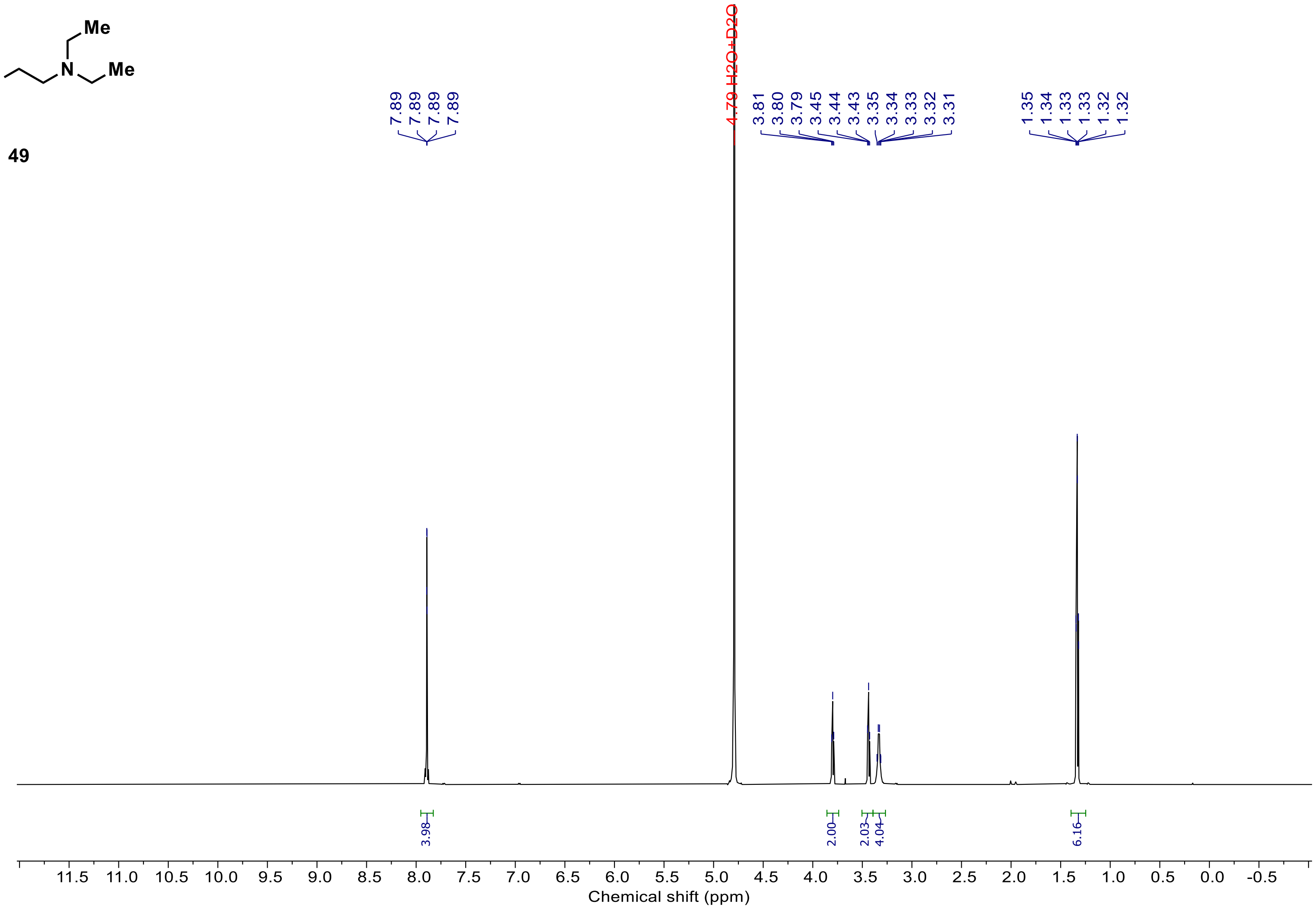

^{13}C NMR spectrum of Procainamide-derived sulfonic acid 49

D_2O, 23°C

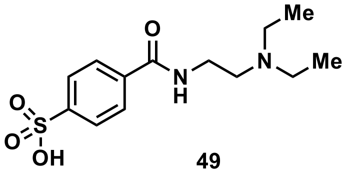

49

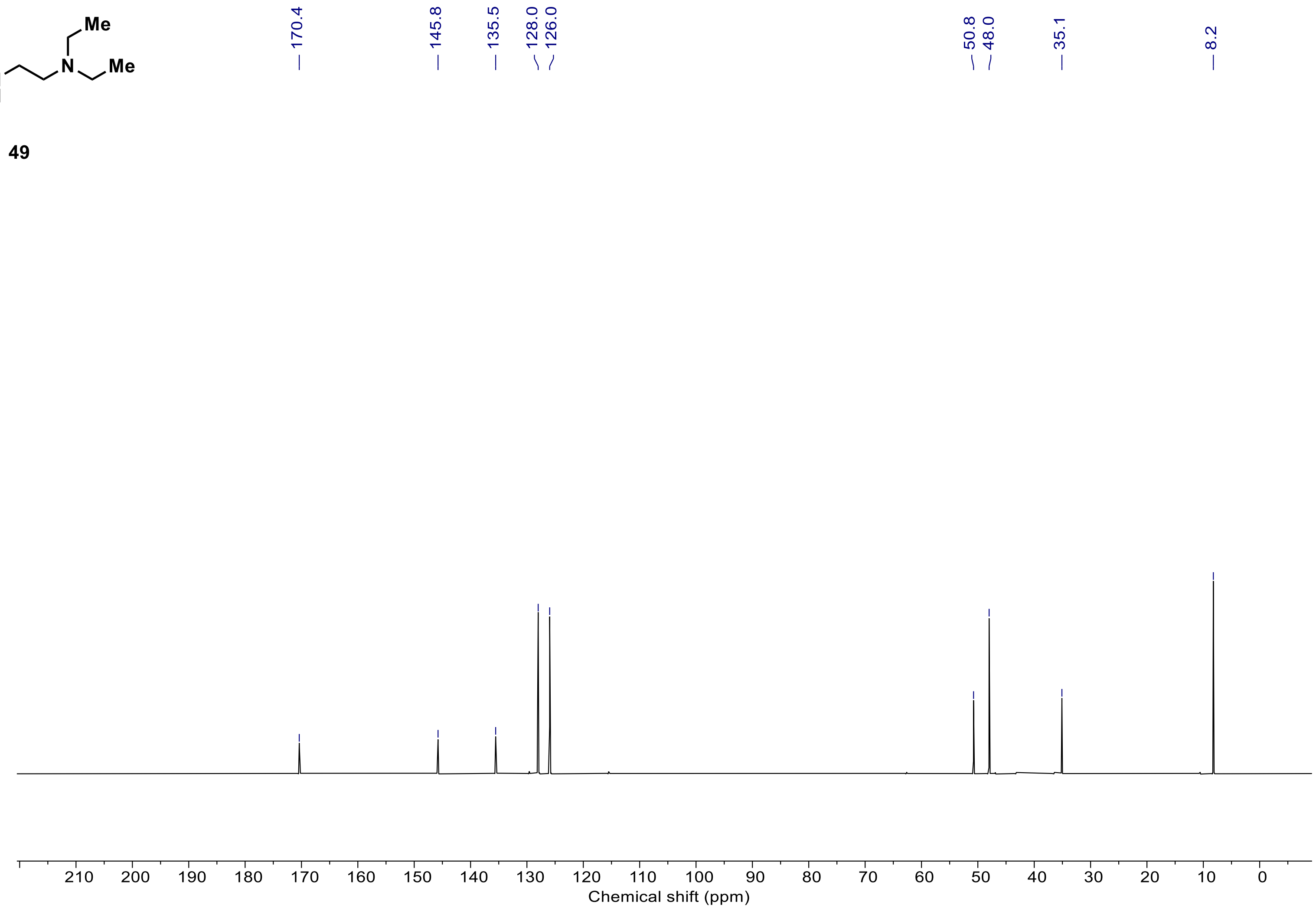

^{1}H NMR spectrum of Sulfapyridine-derived sulfonic acid 50

DMSO-d_6, 23°C

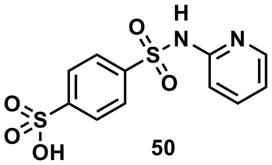

50

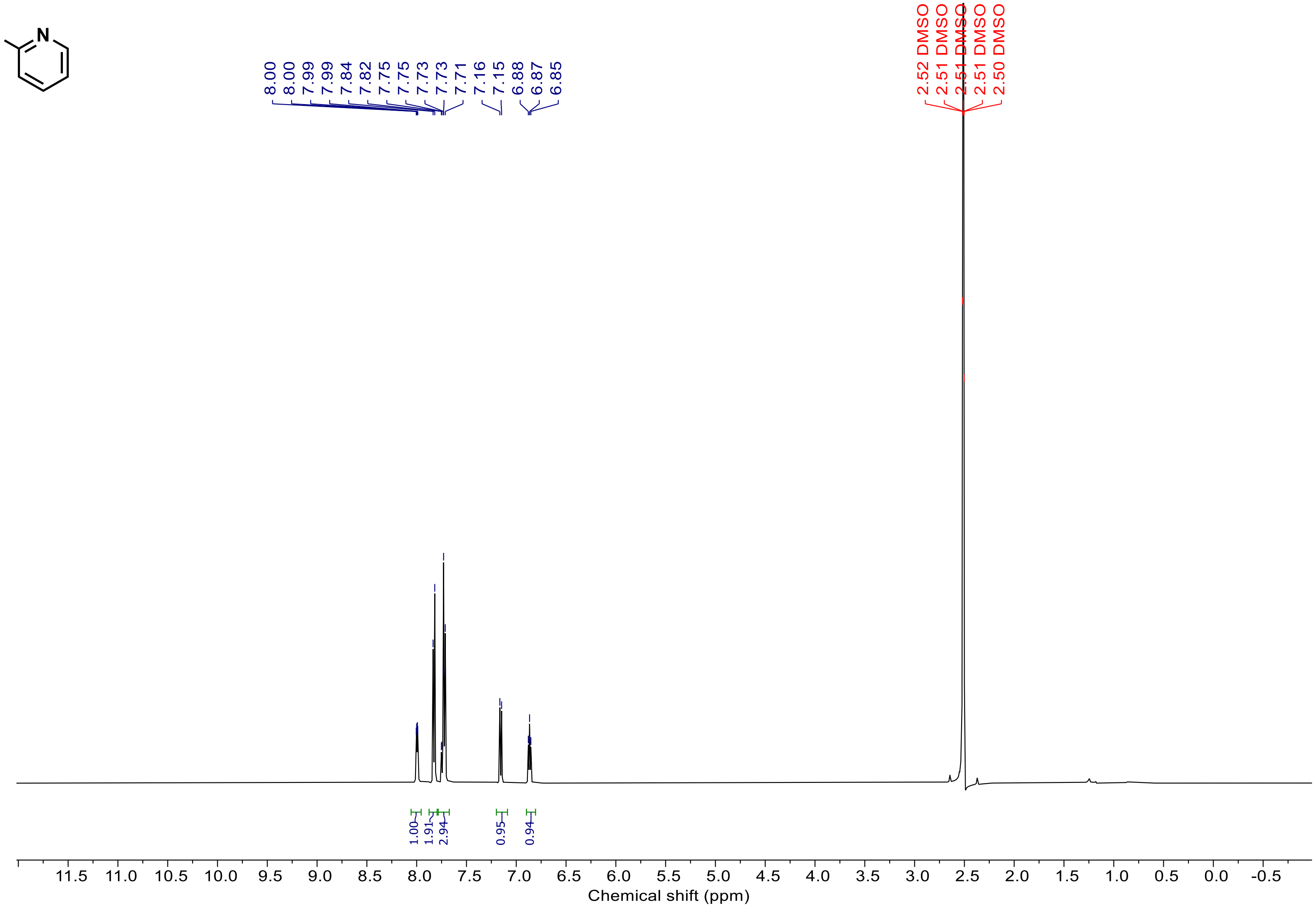

^{13}C NMR spectrum of Sulfapyridine-derived sulfonic acid 50

DMSO-d_6, 23°C

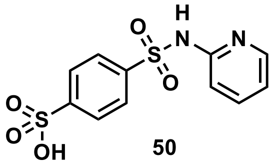

50

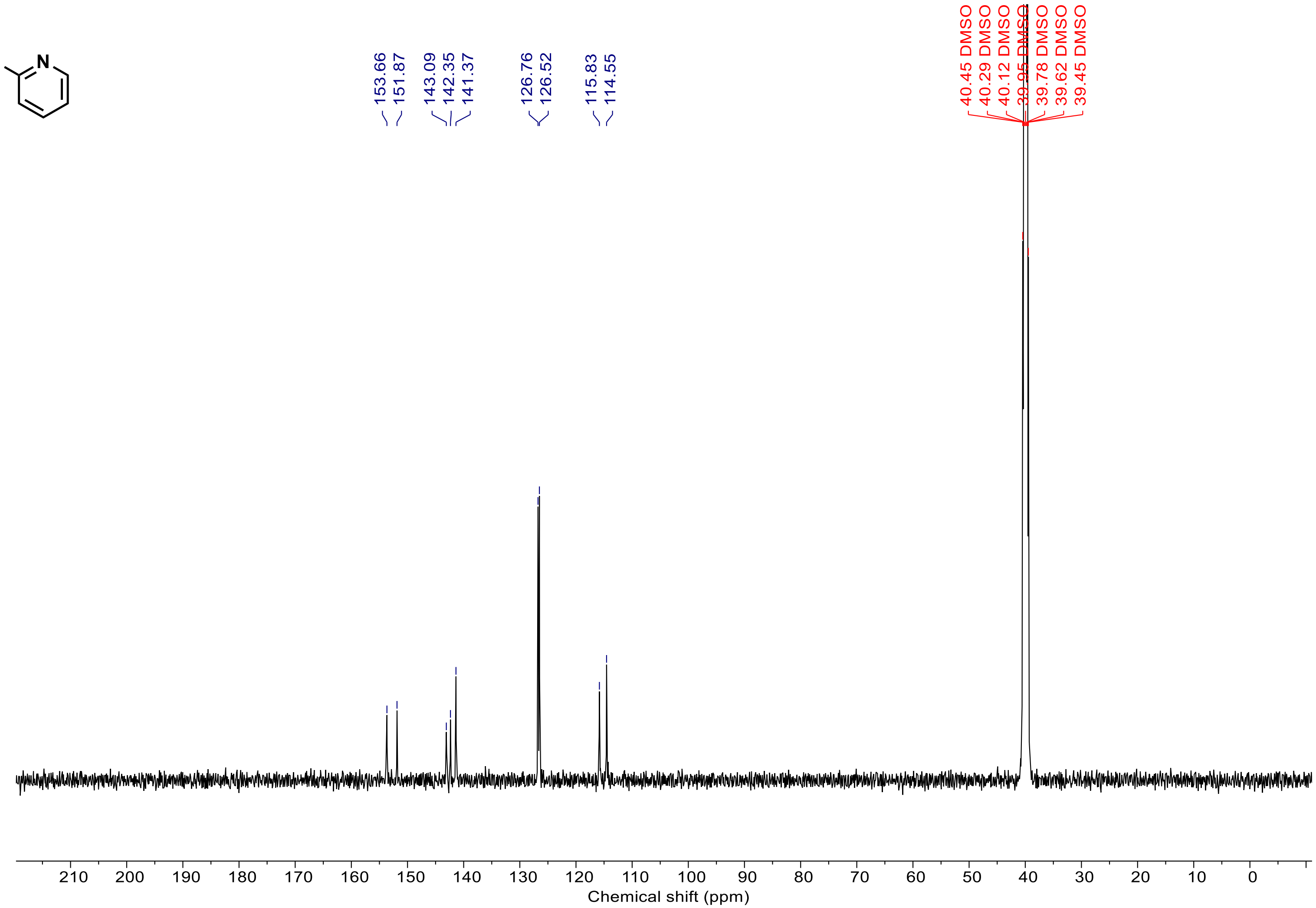

REFERENCES

1. Neese, F., The ORCA program system. *Wiley interdiscip. Rev. Comput. Mol. Sci.* **2012,** *2* (1), 73–78.
2. Neese, F., Software update: the ORCA program system, version 4.0. *Wiley interdiscip. Rev. Comput. Mol. Sci.* **2018,** *8* (1), e1327.
3. Zhao, Y.; Truhlar, D. G., The M06 suite of density functionals for main group thermochemistry, thermochemical kinetics, noncovalent interactions, excited states, and transition elements: two new functionals and systematic testing of four M06-class functionals and 12 other functionals. *Theoretical Chemistry Accounts* **2008,** *120* (1), 215–241.
4. Weigend, F., Accurate Coulomb-fitting basis sets for H to Rn. *Phys. Chem. Chem. Phys.* **2006,** *8* (9), 1057−1065.
5. Weigend, F.; Ahlrichs, R., Balanced basis sets of split valence, triple zeta valence and quadruple zeta valence quality for H to Rn: design and assessment of accuracy. *Phys. Chem. Chem. Phys.* **2005,** *7* (18), 3297−3305.
6. Marenich, A. V.; Cramer, C. J.; Truhlar, D. G., Universal solvation model based on solute electron density and on a continuum model of the solvent defined by the bulk dielectric constant and atomic surface tensions. *J. Phys. Chem. B* **2009,** *113* (18), 6378−6396.
7. *Prudent practices in the laboratory: handling and management of chemical hazards, updated version*. National Academies Press: Washington, DC, 2011.
8. Shigeno, M.; Nakaji, K.; Nozawa-Kumada, K.; Kondo, Y., Catalytic amide–base system of TMAF and N(TMS)3 for deprotonative coupling of benzylic C(sp3)–H bonds with carbonyls. *Org. Lett.* **2019,** *21* (8), 2588–2592.
